# The Preventive Effects of GLP-1 Receptor Agonists and SGLT2 Inhibitors on Cancer Metastasis: A Network Meta-Analysis of 67 Randomized Controlled Trials

**DOI:** 10.3390/ijms26178202

**Published:** 2025-08-23

**Authors:** Chih-Wei Hsu, Bing-Syuan Zeng, Chih-Sung Liang, Bing-Yan Zeng, Chao-Ming Hung, Brendon Stubbs, Yen-Wen Chen, Wei-Te Lei, Jiann-Jy Chen, Po-Huang Chen, Kuan-Pin Su, Tien-Yu Chen, Ping-Tao Tseng

**Affiliations:** 1 Department of Psychiatry, Kaohsiung Chang Gung Memorial Hospital, Chang Gung University College of Medicine, Kaohsiung 833, Taiwan; harwicacademia@gmail.com; 2 Department of Internal Medicine, E-Da Cancer Hospital, I-Shou University, Kaohsiung 824, Taiwan; b95401072@ntu.edu.tw; 3 Department of Psychiatry, Beitou Branch, Tri-Service General Hospital, School of Medicine, National Defense Medical Center, Taipei 112, Taiwan; lcsyfw@gmail.com; 4 Department of Psychiatry, National Defense Medical Center, Taipei 114, Taiwan; 5 Institute of Biomedical Sciences, National Sun Yat-sen University, Kaohsiung 804, Taiwan; holdinggreat@yahoo.com.tw; 6 Department of Internal Medicine, E-Da Dachang Hospital, I-Shou University, Kaohsiung 807, Taiwan; 7 Division of General Surgery, Department of Surgery, E-Da Cancer Hospital, I-Shou University, Kaohsiung 824, Taiwan; ed100647@edah.org.tw; 8 School of Medicine, College of Medicine, I-Shou University, Kaohsiung 824, Taiwan; 9 Department of Psychological Medicine, Institute of Psychiatry, Psychology and Neuroscience, King’s College London, London WC2R 2LS, UK; brendon.stubbs@kcl.ac.uk (B.S.); cobolsu@gmail.com (K.-P.S.); 10 Department of Sport, University of Vienna, 1010 Vienna, Austria; 11 Prospect Clinic for Otorhinolaryngology & Neurology, Kaohsiung 811, Taiwan; kevinachen0527@gmail.com (Y.-W.C.); jiannjy@yahoo.com.tw (J.-J.C.); 12 Section of Immunology, Rheumatology, and Allergy Department of Pediatrics, Hsinchu Munipical MacKay Children’s Hospital, Hsinchu 300, Taiwan; lazyleisure@gmail.com; 13 Center for Molecular and Clinical Immunology, Chang Gung University, Taoyuan 333, Taiwan; 14 Department of Otorhinolaryngology, E-Da Cancer Hospital, I-Shou University, Kaohsiung 824, Taiwan; 15 Division of Hematology and Oncology, Department of Internal Medicine, Tri-Service General Hospital, School of Medicine, National Defense Medical University, Taipei 114, Taiwan; chenpohuang@hotmail.com; 16 Mind-Body Interface Research Center (MBI-Lab), China Medical University Hospital, Taichung 404, Taiwan; 17 College of Medicine, China Medical University, Taichung 404, Taiwan; 18 An-Nan Hospital, China Medical University, Tainan 709, Taiwan; 19 Department of Psychiatry, Tri-Service General Hospital, Taipei 114, Taiwan; 20 Department of Psychiatry, College of Medicine, National Defense Medical University, Taipei 114, Taiwan; 21 Institute of Precision Medicine, National Sun Yat-sen University, Kaohsiung 804, Taiwan; 22 Department of Psychology, College of Medical and Health Science, Asia University, Taichung 413, Taiwan

**Keywords:** network meta-analysis, GLP-1 receptor agonist, SGLT2 inhibitor, metastasis, cancer, malignancy

## Abstract

Metastatic cancer, characterized by poor survival outcomes and grim prognosis, represents the final stage of malignancy. The current evidence regarding the prophylactic effects of glucagon-like peptide-1 (GLP-1) receptor agonists and sodium–glucose cotransporter 2 (SGLT2) inhibitors on metastatic cancer remains largely unexamined. With a confirmatory approach based on the Cochrane recommendation, we conducted a frequentist-based network meta-analysis (NMA) of randomized controlled trials (RCTs) evaluating such medications. The primary outcome was the incidence of metastatic cancer, while secondary outcomes included safety profiles assessed through dropout rates. The findings were reaffirmed by sensitivity analysis with a Bayesian-based NMA. This NMA of 207,606 participants from 67 RCTs revealed that only efpeglenatide demonstrated a statistically significant reduction in metastatic cancer events compared to controls (odds ratio = 0.26, 95% confidence intervals = 0.09 to 0.70, *p* = 0.010, number needed to treat = 188.4). Efpeglenatide’s efficacy was not confined to specific cancer types. Safety profiles were comparable across all treatments. These findings indicate that efpeglenatide may possess a broad, systemic preventive effect against metastatic cancers, potentially operating through mechanisms that are not restricted to individual organ systems. Further research is warranted to elucidate the molecular pathways underlying its anti-metastatic properties and to explore its role in preventive oncology.

## 1. Introduction

Glucagon-like peptide-1 (GLP-1) receptor agonists and sodium–glucose cotransporter 2 (SGLT2) inhibitors have recently emerged as innovative glucose-lowering therapies with mechanisms that differ significantly from traditional treatments [1]. Beyond their primary role in glycemic control, these agents have demonstrated additional therapeutic benefits, including cardiovascular and renal protection, which have been increasingly recognized in recent years.

Recent studies have explored the potential risks and benefits of GLP-1 receptor agonists and SGLT2 inhibitors concerning cancer development. Traditional pairwise meta-analyses suggest that SGLT2 inhibitors might be associated with an increased risk of primary tumors across various contexts [2,3]. Similarly, GLP-1 receptor agonists have also been linked to a heightened risk of primary tumors in some studies [4]. However, other research indicates a neutral effect of GLP-1 receptor agonists [5,6] and SGLT2 inhibitors [7] on primary tumor risk. Despite this growing body of evidence, no research has specifically examined the impact of these agents on metastatic cancers.

Metastatic cancers, representing the final and most severe stage of malignancy, are associated with extremely poor survival rates and prognosis across tumor types [8]. Theoretically, the administration of GLP-1 receptor agonists or SGLT2 inhibitors may influence the expression of matrix metalloproteinases (MMPs) and adhesion molecules, such as intercellular adhesion molecules (ICAM) and vascular cell adhesion molecules (VCAM). These factors play critical roles in cancer initiation and metastasis, suggesting potential for these medications to reduce the incidence of metastatic cancers.

A well-designed network meta-analysis (NMA) provides a valuable methodology for comparing the relative efficacy of different medications, enabling indirect comparisons and the assessment of multiple interventions at varying dosages, for which merit could not be provided by traditional pairwise meta-analyses [9]. To date, no NMA has specifically investigated the preventive potential of GLP-1 receptor agonists and SGLT2 inhibitors in metastatic cancer. This NMA builds on our prior studies regarding the adverse events of neurodegenerative disease [10] and audiology function [11] related to GLP-1 receptor agonists and SGLT2 inhibitors use. Specifically, this NMA aims to (1) compare the preventive efficacy of GLP-1 receptor agonists and SGLT2 inhibitors against metastatic cancers; (2) identify the most effective agents for prevention; (3) evaluate their relative safety profiles in preventive applications; and (4) provide evidence-based recommendations for future preventive strategies.

## 2. Results

### 2.1. Eligibility of the Studies

**Figure 1:**
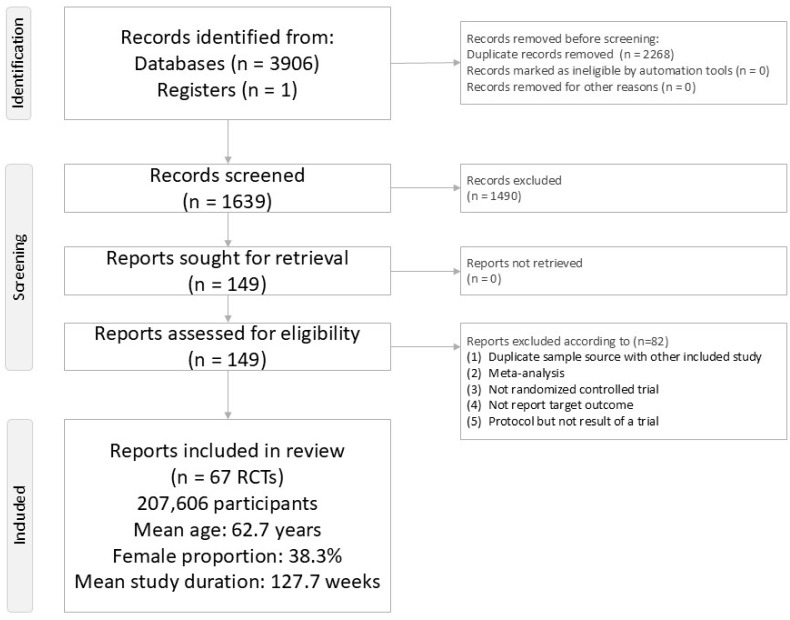
PRISMA2020 Flowchart of current network meta-analysis.

Figure 1 presents the literature screening process for this NMA. After excluding 82 articles (Table S3) [12,13,14,15,16,17,18,19,20,21,22,23,24,25,26,27,28,29,30,31,32,33,34,35,36,37,38,39,40,41,42,43,44,45,46,47,48,49,50,51,52,53,54,55,56,57,58,59,60,61,62,63,64,65,66,67,68,69,70,71,72,73,74,75,76,77,78,79,80,81,82,83,84,85,86,87,88,89,90,91,92,93], we included 59 articles encompassing 67 randomized controlled trials (RCTs) (Table S4) [47,94,95,96,97,98,99,100,101,102,103,104,105,106,107,108,109,110,111,112,113,114,115,116,117,118,119,120,121,122,123,124,125,126,127,128,129,130,131,132,133,134,135,136,137,138,139,140,141,142,143,144,145,146,147,148,149,150,151,152,153,154]. The analysis comprised 207,606 participants (mean age: 62.7 years [range: 41.2–71.9]; 38.3% female [range: 23.4–81.6%]) with a mean study duration of 127.7 weeks (range: 13–281). The network included 16 treatment arms: one placebo/control and 15 varying doses of GLP-1 receptor agonists (tirzepatide, liraglutide, albiglutide, dulaglutide, efpeglenatide, exenatide, semaglutide, and lixisenatide) and SGLT2 inhibitors (bexagliflozin, canagliflozin, empagliflozin, ertugliflozin, dapagliflozin, and sotagliflozin).

### 2.2. Primary Outcome: Overall Events of Metastatic Cancers

**Figure 2:**
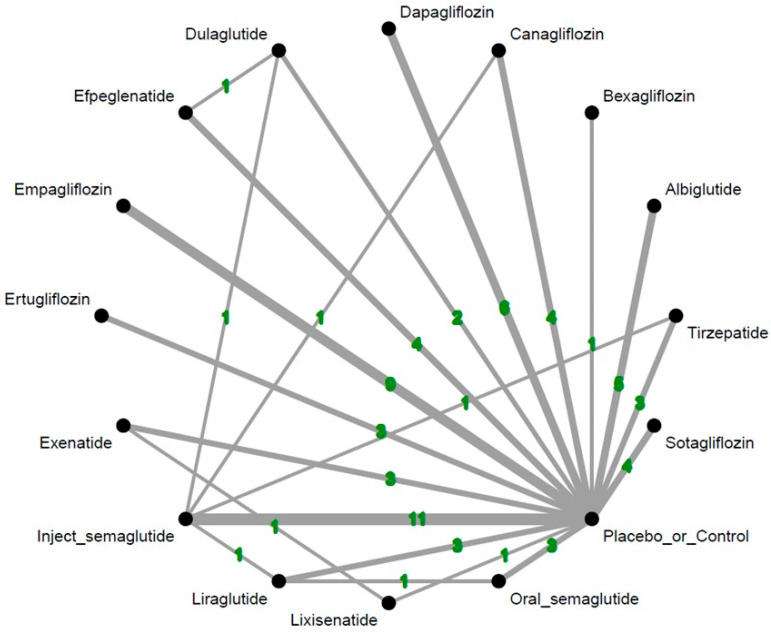
Network structure of the primary outcome: overall events of metastatic cancers. The overall structure of the network meta-analysis. The lines between nodes represent direct comparisons from various trials, with the numbers over the lines indicating the number of trials providing these comparisons for each specific treatment. The thickness of the lines corresponds to the number of trials linked to the network.

**Figure 3:**
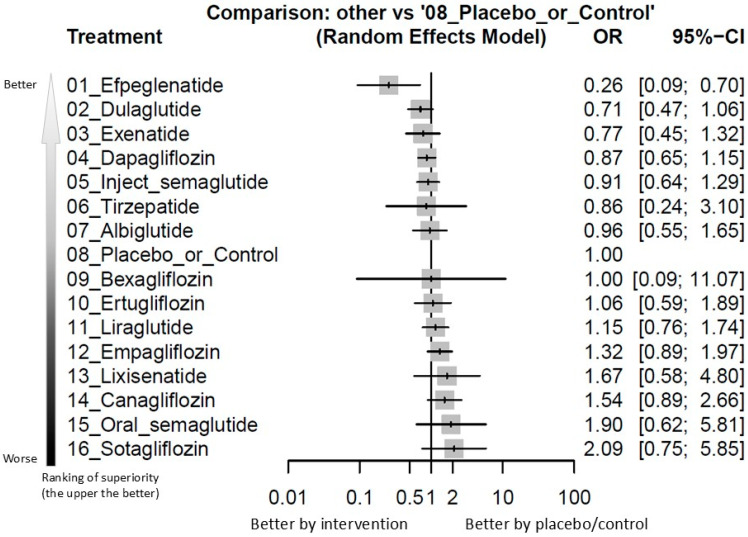
Forest plot of primary outcome: overall events of metastatic cancers. When the effect size (expressed as odds ratio) is less than 1, the specified treatment is associated with fewer events of metastatic cancers than the placebo/controls. Abbreviation: 95%CIs: 95% confidence intervals; GLP-1 agonist: glucagon-like peptide-1 agonist; NMA: network meta-analysis; OR: odds ratio; RCT: randomized controlled trial; SGLT2 inhibitor: sodium–glucose cotransporter 2 inhibitor.

Analysis of overall metastatic cancer events revealed that efpeglenatide, one of the GLP-1 receptor agonists, was associated with significantly fewer events of overall metastatic cancers than the controls [odds ratio (OR) = 0.26, 95% confidence intervals (95%CIs) = 0.09 to 0.70, incidence in efpeglenatide group and control group = 0.145% and 0.676%, respectively, number needed to treat (NNT) = 188.4]. Since there were not any RCTs specifically addressing the incidence rate of metastatic cancers related to efpeglenatide prescription, we calculated the incidence rate of metastatic cancers related to efpeglenatide prescription based on the incidence rate of metastatic cancers from the experimental arms among the included RCTs with efpeglenatide prescription. None of the experimental arms investigated were associated with a significantly higher risk of metastatic cancers than the controls. Among these interventions, the efpeglenatide ranked the best and the dulaglutide ranked second (OR = 0.71, 95%CIs = 0.47 to 1.06 in comparison with controls) (Figure 2 and Figure 3 and Figure S3A, and Table 1).

### 2.3. Subgroup Analyses of the Origin of Metastatic Cancers Based on the ICD-10 Classification System

For metastatic cancers of specific origins, our NMA revealed no significant preventive effects across all investigated interventions (Figures S1A–J, S2A–J, S3B–K, and Table S5A–J).

The evidence base for hematologic cancer origins was limited to a single RCT [103], which prevented meaningful NMA for the subgroup analysis. This paucity of data highlights an important gap in current research regarding the preventive potential of these medications for less common origins of metastatic cancers.

### 2.4. Safety Profile: Drop-Out Rate

The tirzepatide (OR = 0.61, 95%CIs = 0.48 to 0.79), canagliflozin (OR = 0.67, 95%CIs = 0.53 to 0.85), injected form of semaglutide (OR = 0.80, 95%CIs = 0.68 to 0.94), and empagliflozin (OR = 0.83, 95%CIs = 0.72 to 0.96) were associated with significantly lower drop-out rates than the control group. Among the investigated interventions, tirzepatide ranked the best (Figures S1K, S2K and S3L, and Table S5K).

### 2.5. Sensitivity Analysis with Bayesian-Based NMA

Generally, the main result of this study did not differ between the frequentist-based NMA and the Bayesian-based NMA (Figure S4). The Bayesian-based SUCRA list had been depicted in Table S6 and Figure S5A,B. The deviation-model assessment did not demonstrate significant deviation among the current NMA (Figure S6A–C). The residual deviance plot shows that the majority of data points have posterior mean residual deviance values below 2, indicating an overall satisfactory model fit and the absence of strongly misfitting or influential data points. The alignment of points along the diagonal in the dev–dev plot indicates little to no evidence of inconsistency between the consistency and UME models. All trace plots appeared satisfactory, indicating adequate model convergence.

### 2.6. Publication Bias, Risk of Bias and Inconsistency

Small-study effects were not detected through visual inspection of comparison-adjusted funnel plots (Figure S7A). Further, publication bias was not significantly based on statistically insignificant Egger’s regression test results (Figure S7B). We identified that 75.7% (355/469 items), 17.7% (83/469 items), and 6.6% (31/469 items) of the included studies had low, unclear, and high risks of bias, respectively (Figure S8A,B). The inconsistency test, evaluating the assumption of consistency, showed no significant inconsistencies in the present NMA based on loop-specific methods, node-splitting approaches, and design-by-treatment interaction models (Table S7A,B). Assessment of heterogeneity using τ^2^ statistics did not reveal substantial between-study variability across networks (Table S8). The overall certainty of evidence of this NMA falls within moderate-high according to GRADE ratings (Table S9).

## 3. Discussion

### 3.1. Prophylactic Potential of Efpeglenatide

This NMA reveals efpeglenatide’s unique prophylactic benefit against metastatic cancers (OR = 0.26, 95% CI: 0.09-0.70, NNT = 188.4), representing a potentially significant advance in preventing the spread of cancer cells, which served as a key favorable flag in preventive oncology. Among all evaluated GLP-1 receptor agonists and SGLT2 inhibitors, only efpeglenatide demonstrated significant preventive effects. The agent’s efficacy across multiple cancer types suggests a broad, systemic mechanism of action, rather than organ-specific effects.

### 3.2. Comparison with Previous Studies

To the best of our knowledge, this study is the first NMA to assess and compare the prophylactic benefits of individual GLP-1 receptor agonists and SGLT2 inhibitors on metastatic cancers with various origins. Unlike previous traditional pairwise meta-analyses, which either found a higher risk [2,3,4] or no significant risk difference [5,6,7] for primary cancers, our NMA focused on metastatic cancers and observed a significantly prophylactic effect on the incidence of metastatic cancers by efpeglenatide, but no other GLP-1 receptor agonists or SGLT2 inhibitors. The divergence between our findings and prior analyses primarily results from the methodological advantage of NMAs and different target outcomes. Traditional pairwise meta-analyses tend to pool varying regimens into one single comparison group and would potentially obscure significant benefits of individual drugs due to the heterogeneity within pooled treatments. In contrast, NMAs allow for multiple comparisons across different regimens so that it would enhance the power to detect the efficacy of individual drugs and provide a clearer view of the relative benefits of different dosages [9]. This methodological strength enables us to identify specific agents, such as efpeglenatide, which may hold promise for the prevention of metastatic disease, while also highlighting areas where the current evidence remains insufficient. In addition, metastatic cancers relied on different mechanisms from the primary tumor. To be specific, the cancer metastasis depended on the interaction between MMPs family, an adhesion molecule family, and numerous environmental factors within the human body [155,156], which factors played less role in the formation of primary tumors.

### 3.3. Mechanistic Insights

A key finding from this NMA was that only the efpeglenatide, one of the GLP-1 receptor agonists, was associated with significantly fewer events of overall metastatic cancers than the control group. Further, this preventive effect was not limited to any specific systems of metastatic cancer origin. These findings suggest that the mechanisms of preventive effects on metastatic cancers by efpeglenatide may rely on a more general and extensive way but are not limited to a confined mechanism specified to a specific organ system. To be specific, the GLP-1 receptor agonists had been found to suppress MMP-1 expression by inhibiting the extracellular regulated protein kinase 1/2 (ERK1/2) and nuclear factor kappa-B (NF-κB) pathways [157]. In the animal model, the overt expression of MMP-1 was associated with the empowerment of the primary tumor to spontaneously metastasize to the other organ [156]. Further, the prescription of GLP-1 receptor agonists could modulate the expression of an adhesion molecules family (i.e., ICAM and VCAM) [158], in which molecule stability played an important role in cancer metastatic mechanism [155]. As we know, the formation and metastasis of metastatic tumors was a sub-chronic process. The efpeglenatide, which had a longer half-life (from weeks to months) [159], might hypothetically provide steady and sustained suppression of metastasis-related molecules, thereby potentially preventing the incidence of metastatic cancers. Supporting this hypothesis, the dulaglutide, another GLP-1 receptor agonist with a long half-life [159], had a trend of prophylactic effects on metastatic cancers (OR = 0.71, 95%Cis = 0.47 to 1.06 in comparison with controls) and was ranked the second in overall metastatic cancer prevention. Therefore, based on the aforementioned evidence, the GLP-1 receptor agonists with a longer half-life might hypothetically exert prophylactic benefits against metastatic cancers. Finally, the null findings of subgroup analysis focusing on specific origins of metastatic cancers might reflect another potential issue, if any, that the protective effect of efpeglenatide on specific origins of metastatic cancers might not achieve significance due to insufficient statistical power. This issue is more relevant in the subgroup of digestive organ origins, the subgroup of prostate/male genital organ origins, and the subgroup of neuron/nerve/neuroendocrine origins, among which the subgroup analysis remained a trend of protection by efpeglenatide prescription.

### 3.4. Clinical Implications of NNT

The value of NNT of efpeglenatide (NNT = 188.4) for metastatic cancer prevention represents a clinically meaningful value. While NNT thresholds varied by condition and context, values between 100 and 200 are generally considered acceptable for life-threatening conditions [160]. For comparison, established preventive interventions like statins demonstrate NNTs of 154 for men and 1075 for women in preventing coronary death, supporting efpeglenatide’s clinical relevance in metastatic cancer prevention.

Further, in other aspects of the prevention medicine field, the low-dose computed tomography scanning prevents one death from any cause with an NNT of 219 compared to a conventional chest X-ray screening [161], while aspirin for primary prevention requires treating 241 patients to prevent one composite cardiovascular outcome (NNT = 241) [162]. As we know, we did not have a satisfactory treatment strategy for metastatic cancers, which are a major disease and lead to high mortality. The finding of NNT = 188.4 in our NMA gained particular significance when compared to the NNT values of other well-established preventive interventions in major diseases. Given the aforementioned evidence, the fact that efpeglenatide’s NNT for preventing cancer metastasis fell within a comparable range to these proven preventive measures was a remarkable clue for the future regimen development. Given the current limitations in metastasis prevention options, which are largely restricted to anti-cancer treatments, this represents a significant advancement in oncological care.

### 3.5. Gaps in Evidence

Although the dose- and duration-exposure would be clinically relevant variables in oncology studies, we could not perform further subgroup analysis focusing on different dosages or treatment durations due to insufficient numbers of RCTs. In addition, there had been no pharmacokinetics studies focusing on efpeglenatide’s pharmacokinetic properties and metastatic cancer prevention. Finally, the evidence bases for less common origins of metastatic cancers, specifically those arising from hematologic cancers, proved insufficient for definitive conclusions, highlighting critical gaps in current research.

### 3.6. Strengths and Limitations

This NMA offers key methodological advantages. The NMA design enables a comprehensive comparison of GLP-1 receptor agonists and SGLT2 inhibitors, surpassing traditional pairwise meta-analyses. Our methodology strengthens evidence quality through the exclusive inclusion of randomized controlled trials and the careful exclusion of participants with pre-existing metastatic cancers, ensuring true prophylactic effects. The organ-specific subgroup analyses provide clinicians with detailed evidence for tailoring preventive strategies. Finally, to enhance reliability, we also arranged sensitivity analysis with a Bayesian-based NMA to re-affirm the main result of the current study, to which sensitivity analysis revealed similar results.

Some limitations warrant consideration. First, insufficient evidence for rare cancers (e.g., hematologic malignancies) limits generalizability for these populations. Second, our exclusive focus on RCTs excluded potentially valuable long-term observational data. Third, varying diagnostic approaches across multi-country trials may have introduced case-identification heterogeneity, affecting the estimate’s precision. Although there was no significant publication bias detected in our NMA, adverse effects data are often handled with less rigor than the primary beneficial outcomes of a study. Therefore, incomplete reporting might still exist when synthesizing data. Although meta-analyses may raise concerns about “inconsistencies in diagnostic tools,” the nature of these studies are rooted in large databases or numerous clinical studies, making them more reflective of clinical experience. Therefore, many meta-analyses on drug side effects [163,164] have become a benchmark for guiding future research [165,166]. Finally, since the primary outcome of this NMA counted event occurrences rather than the number of affected patients, some patients with multiple metastasis might be counted multiple times. These limitations underscore the need for standardized, prospective studies specifically designed to evaluate metastatic cancer prevention.

## 4. Methods and Materials

In this NMA, we used the confirmatory approach to specifically focus on particular adverse effects of interest (i.e., incidence of tumor metastasis here) based on the Cochrane recommendation [167]. This NMA adhered to the guidelines outlined in the Preferred Reporting Items for Systematic Reviews and Meta-Analyses extension for network meta-analyses (PRISMA NMA) [168] (Table S1). The study protocol was registered with PROSPERO under registration number CRD42024611311 on 7 November 2024. The study protocol was approved by the Institutional Review Board of the Tri-Service General Hospital, National Defense Medical Center (TSGHIRB E202416043) on 13 November 2024.

### 4.1. Database Searches and Study Identification

Comprehensive searches were conducted across PubMed, Embase, ClinicalKey, Cochrane CENTRAL, ProQuest, ScienceDirect, Web of Science, and ClinicalTrials.gov (Table S2) to identify studies published up to 13 November 2024. Two independent reviewers (PT Tseng and BS Zeng) screened titles and abstracts, resolving any disagreements through consensus. Additionally, manual searches of reference lists from relevant reviews and meta-analyses were performed to ensure thorough inclusion [2,3,4,5,6,7,12,13,14,15,16]. No language restrictions were applied during the search.

### 4.2. Inclusion and Exclusion Criteria

The primary focus of this NMA was the prophylactic impact of GLP-1 receptor agonists and SGLT2 inhibitors, excluding RCTs specifically designed to involve patients with pre-existing metastatic cancers. The study selection followed the PICOS framework (Population, Intervention, Comparison, Outcome, and Study design): (1) Population: Human participants without definitely pre-existing metastatic cancers; (2) Intervention: GLP-1 receptor agonists or SGLT2 inhibitors; (3) Comparison: Control groups receiving standard care or placebo; (4) Outcome: As outlined in primary and secondary outcomes; and (5) Study design: RCTs.

This NMA focused on assessing prophylactic effects so that we did not include those RCTs specifically designed to recruit pre-existing metastatic cancers at baseline. To limit heterogeneity, we included only studies comparing GLP-1 receptor agonists or SGLT2 inhibitors. Additionally, to enhance reliability and reduce selective reporting bias [169], we only included RCTs with systematic screening for adverse events or those directly assessing our target outcomes. Therefore, the inclusion criteria were as follows: (1) RCTs with participants free of pre-existing metastatic cancers at baseline; (2) RCTs involving GLP-1 receptor agonists or SGLT2 inhibitors; (3) Studies on human participants; and (4) RCTs that systematically screened for adverse events or specifically targeted these outcomes.

Exclusion criteria included: (1) studies that were not RCTs; (2) RCTs involving participants with pre-existing metastatic cancers; (3) RCTs not directly comparing GLP-1 receptor agonists or SGLT2 inhibitors; (4) RCTs lacking information on target outcomes; and (5) animal studies.

### 4.3. Methodological Quality Appraisal

Two reviewers independently evaluated the risk of bias using the Cochrane Risk of Bias Tool 1.0 [170], achieving inter-rater reliability of 0.86. Discrepancies were resolved by a third reviewer.

### 4.4. Outcome Definition

The primary outcome was the total number of overall metastatic cancer events recorded in registry systems. In situations of multi-metastasis, we count individuals but not events. The safety profile was assessed through drop-out rates (i.e., participants who withdrew from the study before completion for any reason).

### 4.5. Data Extraction, Management and Conversion

The literature and studies screen process included two steps. At the initial screening step of the “Title-Abstract Screening”, we excluded the literature and studies that were not related to topics of metastatic cancers, not related to GLP-1 receptor agonists or SGLT2 inhibitors prescription, and not human trials. After passing the initial screening step, the remaining literature and study registries entered the “Detailed Information Screening, either Full-Text or Full Information in Registry System” step. At this step, we screened word by word within the full text or registry system of the remaining literature and study registries. Data extraction was independently performed by two authors (PT Tseng and BS Zeng), recording demographic data, study design, treatment details, primary outcomes, and safety information. If essential data were missing, we reached out to corresponding authors. The data extraction adhered to protocols from the Cochrane Handbook for Systematic Reviews of Interventions and other pertinent medical literature [171].

### 4.6. Statistical Analyses

Given the multiple treatment arms, we used a random-effects model for the NMA [172], utilizing MetaInsight (version 4.0.2, Complex Reviews Support Unit, National Institute for Health Research, London, UK) within a frequentist framework. MetaInsight is a web-based platform for conducting NMAs using the *netmeta* package in R software (https://crsu.shinyapps.io/MetaInsight/, accessed on 13 December 2024) for frequentist statistical analysis [173].

For categorical data, a continuity correction of single-zero-event studies was applied in the meta-analytical procedure. However, for studies with zero events in both the intervention and the control arms, such a correction was not applied to avoid increasing the bias. Rather, we excluded that comparison instead [174,175]. We first generated forest plots to display ORs with 95%CIs for categorical outcomes (i.e., event numbers in registry systems) [176]. Treatment rankings and effect sizes for both direct and indirect comparisons were then tabulated. To assess the consistency between direct and indirect evidence, we applied the “node-splitting” method, which separates direct and indirect components of a particular comparison (node) and is particularly useful in an NMA when trial-level data are available [173,177]. Further, we also arranged a loop-specific method and a design-by-treatment interaction model to evaluate potential inconsistency [178]. Statistical significance was determined by a two-tailed *p*-value of less than 0.05. Publication bias was assessed by a visual inspection of comparison-adjusted funnel plots with a placebo as the comparator. Additionally, we conducted the Egger test to examine the asymmetry of the funnel plot. Between-study heterogeneity was estimated and reported using the posterior distribution of the heterogeneity parameter (tau).

### 4.7. Sensitivity Analyses

To assess the robustness of our findings, we conducted a subgroup analysis by subgrouping RCTs by the origin of metastatic cancers. To be specific, based on the International Classification of Diseases, Tenth Edition (ICD-10) classification system, the origin of the metastatic cancers was defined to include: (1) head, eyes, ears, nose, and throat (HEENT), (2) digestive organs, (3) respiratory and intrathoracic organs, (4) bone, (5) skin, mesothelium, soft tissue, and cartilage, (6) breast and female genital organs, (7) prostate and male genital organs, (8) kidney and urinary tract, (9) neuron, nerve, and neuroendocrine, (10) thyroid and other endocrine glands, and (11) hematologic cancers. Additional subgroup analyses were carried out within these categories.

Further, to re-affirm the reliability and the convergence of the investigated treatment estimates, we arranged a sensitivity analysis with a Bayesian-based NMA to re-run the analytic process of the main result of primary outcome. Specifically, for all Bayesian-based NMAs, we used weakly informative priors to ensure minimal influence on posterior estimates while facilitating model convergence. All priors were centered on the null value (no effect) and were wide enough to include all plausible effect sizes. The inconsistency assumption was examined using an Unrelated Mean Effects (UME) model. Deviance–deviance plots comparing the deviance residuals from the consistency and UME (inconsistency) models were generated to assess model fit and detect potential inconsistency within the network. Further, we arranged a Bayesian-based surface under the cumulative ranking (SUCRA) evaluations by Litmus Rank-O-Gram and radial SUCRA plots [179], to evaluate the rank of superiority of individual regimen. We arranged a deviation model to evaluate the deviation of the treatment estimates [180]. Finally, we evaluated the overall quality of evidence in our NMA with the GRADE [181].

### 4.8. General Declaration

This study complies with the principles outlined in the Declaration of Helsinki.

## 5. Conclusions

This comprehensive NMA reveals a potentially important breakthrough in metastatic cancer prevention, demonstrating that the long half-life GLP-1 receptor agonist, the efpeglenatide, significantly reduces the risk of metastatic cancer (OR = 0.26, 95%CIs = 0.09 to 0.70) with an acceptable NNT value (188.4). Further, this preventive effect was not limited to any specific systems of metastatic cancer origin. These findings suggest that the mechanisms of preventive effects on metastatic cancers by efpeglenatide may rely on a more general and extensive way, such as an adhesion molecules family and an MMP family, but not limited to a confined mechanism specified to a specific organ system. The specificity of efpeglenatide’s preventive effect suggests distinct metastasis preventive mechanisms that warrant further investigation. Future prospective RCTs should focus on the determination of efpeglenatide’s optimal preventive dosage. In addition, future mechanistic studies should be warranted to elucidate the underlying mechanisms of efpeglenatide’s metastasis preventive effects.

## Figures and Tables

**Table 1:** League table of the primary outcome: overall events of metastatic cancers.

**Efpeglenatide**	1.50 [0.06; 36.96]	**.**	**.**	**.**	**.**	**.**	* 0.22 [0.08; 0.63]	**.**	**.**	**.**	**.**	**.**	**.**	**.**	**.**
0.36 [0.12; 1.05]	Dulaglutide	.	.	0.33 [0.01; 8.23]	.	.	0.73 [0.49; 1.10]	.	.	.	.	.	.	.	.
0.33 [0.11; 1.05]	0.92 [0.47; 1.81]	Exenatide	.	.	.	.	0.78 [0.45; 1.34]	.	.	.	.	0.33 [0.01; 8.24]	.	.	.
* 0.29 [0.10; 0.84]	0.82 [0.50; 1.33]	0.88 [0.48; 1.63]	Dapagliflozin	.	.	.	0.87 [0.65; 1.15]	.	.	.	.	.	.	.	.
* 0.28 [0.10; 0.82]	0.78 [0.46; 1.33]	0.85 [0.44; 1.62]	0.96 [0.61; 1.51]	Inject_ semaglutide	1.00 [0.04; 24.59]	.	0.91 [0.63; 1.31]	.	.	0.43 [0.02; 10.69]	.	.	0.33 [0.01; 8.23]	.	.
0.30 [0.06; 1.53]	0.83 [0.21; 3.18]	0.90 [0.22; 3.63]	1.01 [0.27; 3.79]	1.06 [0.28; 3.97]	Tirzepatide	.	0.85 [0.21; 3.45]	.	.	.	.	.	.	.	.
* 0.27 [0.08; 0.84]	0.74 [0.38; 1.45]	0.80 [0.37; 1.73]	0.91 [0.49; 1.68]	0.95 [0.49; 1.82]	0.89 [0.22; 3.62]	Albiglutide	0.96 [0.55; 1.65]	.	.	.	.	.	.	.	.
* 0.26 [0.09; 0.70]	0.71 [0.47; 1.06]	0.77 [0.45; 1.32]	0.87 [0.65; 1.15]	0.91 [0.64; 1.29]	0.86 [0.24; 3.10]	0.96 [0.55; 1.65]	Placebo_ or_Control	1.00 [0.09; 11.03]	0.95 [0.53; 1.69]	0.92 [0.60; 1.40]	0.76 [0.51; 1.13]	0.62 [0.20; 1.91]	0.66 [0.38; 1.15]	0.38 [0.11; 1.26]	0.48 [0.17; 1.34]
0.26 [0.02; 3.45]	0.71 [0.06; 8.07]	0.77 [0.07; 9.00]	0.87 [0.08; 9.74]	0.91 [0.08; 10.28]	0.86 [0.06; 13.06]	0.96 [0.08; 11.23]	1.00 [0.09; 11.03]	Bexagliflozin	.	.	.	.	.	.	.
* 0.24 [0.08; 0.77]	0.67 [0.33; 1.35]	0.73 [0.33; 1.61]	0.82 [0.43; 1.56]	0.86 [0.44; 1.69]	0.81 [0.20; 3.33]	0.91 [0.41; 2.01]	0.95 [0.53; 1.69]	0.95 [0.08; 11.22]	Ertugliflozin	.	.	.	.	.	.
* 0.22 [0.07; 0.66]	0.62 [0.35; 1.10]	0.67 [0.34; 1.32]	0.76 [0.46; 1.25]	0.79 [0.46; 1.36]	0.75 [0.19; 2.88]	0.83 [0.42; 1.65]	0.87 [0.57; 1.32]	0.87 [0.08; 9.98]	0.92 [0.45; 1.87]	Liraglutide	.	.	.	5.05 [0.24; 105.72]	.
* 0.19 [0.07; 0.57]	* 0.54 [0.30; 0.94]	0.58 [0.30; 1.14]	0.66 [0.40; 1.07]	0.69 [0.40; 1.17]	0.65 [0.17; 2.49]	0.72 [0.37; 1.42]	0.76 [0.51; 1.13]	0.76 [0.07; 8.65]	0.80 [0.40; 1.61]	0.87 [0.49; 1.54]	Empagliflozin	.	.	.	.
* 0.15 [0.04; 0.66]	0.42 [0.14; 1.32]	0.46 [0.14; 1.47]	0.52 [0.17; 1.56]	0.54 [0.18; 1.66]	0.51 [0.10; 2.72]	0.57 [0.17; 1.89]	0.60 [0.21; 1.73]	0.60 [0.04; 8.30]	0.63 [0.19; 2.12]	0.69 [0.22; 2.15]	0.79 [0.26; 2.46]	Lixisenatide	.	.	.
* 0.17 [0.05; 0.52]	* 0.46 [0.23; 0.90]	0.50 [0.23; 1.07]	0.56 [0.30; 1.04]	0.59 [0.31; 1.12]	0.56 [0.14; 2.25]	0.62 [0.29; 1.34]	0.65 [0.38; 1.12]	0.65 [0.06; 7.63]	0.69 [0.31; 1.52]	0.75 [0.38; 1.48]	0.86 [0.44; 1.68]	1.08 [0.33; 3.55]	Canagliflozin	.	.
* 0.13 [0.03; 0.61]	0.37 [0.11; 1.22]	0.40 [0.12; 1.40]	0.46 [0.14; 1.45]	0.48 [0.15; 1.54]	0.45 [0.08; 2.48]	0.50 [0.15; 1.75]	0.53 [0.17; 1.61]	0.53 [0.04; 7.46]	0.56 [0.16; 1.96]	0.60 [0.19; 1.95]	0.70 [0.21; 2.28]	0.88 [0.19; 4.08]	0.81 [0.23; 2.81]	Oral_ semaglutide	.
* 0.12 [0.03; 0.52]	0.34 [0.11; 1.02]	0.37 [0.11; 1.18]	0.42 [0.14; 1.21]	0.43 [0.15; 1.29]	0.41 [0.08; 2.13]	0.46 [0.14; 1.47]	0.48 [0.17; 1.34]	0.48 [0.04; 6.54]	0.51 [0.16; 1.65]	0.55 [0.18; 1.67]	0.63 [0.21; 1.91]	0.80 [0.18; 3.48]	0.74 [0.23; 2.36]	0.91 [0.20; 4.15]	Sotagliflozin

Data presents as OR [95%CIs]. Pairwise (upper-right portion) and network (lower-left portion) meta-analysis results are presented as estimate effect sizes for the outcome of overall events of metastatic cancers. Interventions are reported in order of mean ranking of beneficially prophylactic effect on overall events of metastatic cancers, and outcomes are expressed as odds ratio (OR) (95% confidence intervals) (95%CIs). For the pairwise meta-analyses, OR of less than 1 indicates that the treatment specified in the row achieved a more beneficial effect than that specified in the column. For the network meta-analysis (NMA), OR of less than 1 indicates that the treatment specified in the column achieved a more beneficial effect than that specified in the row. Bold results marked with * indicate statistical significance. Abbreviation: 95%CIs: 95% confidence intervals; GLP-1 agonist: glucagon-like peptide-1 agonist; NMA: network meta-analysis; OR: odds ratio; RCT: randomized controlled trial; SGLT2 inhibitor: sodium–glucose cotransporter 2 inhibitor.

## Data Availability

All the data of the current study were available at reasonable request to the corresponding authors.

## Supplementary Materials

The following supporting information can be downloaded at: https://www.mdpi.com/article/10.3390/ijms26178202/s1. References included below [182].
