# Supplementary material for: The Preventive Effects of GLP-1 Receptor Agonists and SGLT2 Inhibitors on Cancer Metastasis: A Network Meta-Analysis of 67 Randomized Controlled Trials
Source: Int J Mol Sci. 2025 Aug 23;26(17):8202. doi: 10.3390/ijms26178202 (PMC12428495; doi:10.3390/ijms26178202)
Supplement: Supplementary file 1 [file ijms-26-08202-s001.zip › ijms-3793936-supplementary.pdf]

List of content in supplement materials

The Preventive Effects of GLP-1 Receptor Agonists and SGLT2 Inhibitors on Cancer Metastasis

A Network Meta-analysis of 67 Randomized Controlled Trials

Chih-Wei Hsu, et al.

Figure S1	(A) Network structure of NMA of primary outcome: subgroup analysis of head, eyes, ears, nose, and throat origin
	(B) Network structure of NMA of primary outcome: subgroup digestive organ origin
	(C) Network structure of NMA of primary outcome: subgroup analysis of respiratory and intrathoracic organ origin
	(D) Network structure of NMA of primary outcome: subgroup analysis of bone origin
	(E) Network structure of NMA of primary outcome: subgroup analysis of skin, mesothelium, soft tissue, and cartilage origin
	(F) Network structure of NMA of primary outcome: subgroup analysis of breast and female genital organ origin
	(G) Network structure of NMA of primary outcome: subgroup analysis of prostate and male genital organ origin
	(H) Network structure of NMA of primary outcome: subgroup analysis of kidney and urinary tract origin
	(I) Network structure of NMA of primary outcome: subgroup analysis of neuron, nerve, and neuroendocrine origin
	(J) Network structure of NMA of primary outcome: subgroup analysis of thyroid and other endocrine gland origin
	(K) Network structure of NMA of safety profile: drop-out rate
Figure S2	(A) Forest plot of NMA of primary outcome: subgroup analysis of head, eyes, ears, nose, and throat origin
	(B) Forest plot of NMA of primary outcome: subgroup analysis of digestive organ origin
	(C) Forest plot of NMA of primary outcome: subgroup analysis of respiratory and intrathoracic organ origin
	(D) Forest plot of NMA of primary outcome: subgroup analysis of bone origin
	(E) Forest plot of NMA of primary outcome: subgroup analysis of skin, mesothelium, soft tissue, and cartilage origin
	(F) Forest plot of NMA of primary outcome: subgroup analysis of breast and female genital organ origin
	(G) Forest plot of NMA of primary outcome: subgroup analysis of prostate and male genital organ origin
	(H) Forest plot of NMA of primary outcome: subgroup analysis of kidney and urinary tract origin
	(I) Forest plot of NMA of primary outcome: subgroup analysis of neuron, nerve, and neuroendocrine origin
	(J) Forest plot of NMA of primary outcome: subgroup analysis of thyroid and other endocrine gland origin
	(K) Forest plot of NMA of safety profile: drop-out rate
Figure S3	(A) Individual study result of primary outcome: overall events of metastatic cancers
	(B) Individual study result of primary outcome: subgroup analysis of head, eyes, ears, nose, and throat origin

	(C) Individual study result of primary outcome: subgroup analysis of digestive organ origin
	(D) Individual study result of primary outcome: subgroup analysis of respiratory and intrathoracic organ origin
	(E) Individual study result of primary outcome: subgroup analysis of bone origin
	(F) Individual study result of primary outcome: subgroup analysis of skin, mesothelium, soft tissue, and cartilage origin
	(G) Individual study result of primary outcome: subgroup analysis of breast and female genital organ origin
	(H) Individual study result of primary outcome: subgroup analysis of prostate and male genital organ origin
	(I) Individual study result of primary outcome: subgroup analysis of kidney and urinary tract origin
	(J) Individual study result of primary outcome: subgroup analysis of neuron, nerve, and neuroendocrine origin
	(K) Individual study result of primary outcome: subgroup analysis of thyroid and other endocrine gland origin
	(L) Individual study result of safety profile: drop-out rate
Figure S4	Bayesian-based forest plot of NMA of primary outcome: overall events of metastatic cancers
Figure S5	(A) Bayesian-based Litmus Rank-O-Gram rank plot of primary outcome: overall events of metastatic cancers
	(B) Bayesian-based radial surface under the cumulative ranking of primary outcome: overall events of metastatic cancers
Figure S6	(A) Bayesian-based residual deviance NMA/UME model of primary outcome: overall events of metastatic cancers
	(B) Bayesian-based per-arm residual deviance of primary outcome: overall events of metastatic cancers
	(C) Bayesian-based leverage plot of primary outcome: overall events of metastatic cancers
Figure S7	(A) Funnel plot of primary outcome: overall events of metastatic cancers
	(B) Egger test of primary outcome: overall events of metastatic cancers
Figure S8	(A) Overview of risk of bias
	(B) Detailed risk of bias in each study
Table S1	PRISMA 2020 checklist of the current network meta-analysis
Table S2	Keyword used in each database and search results
Table S3	Excluded studies and reason
Table S4	Characteristics of the included studies
Table S5	(A): League table of NMA of primary outcome: subgroup of head, eyes, ears, nose, and throat origin
	(B): League table of NMA of primary outcome: subgroup of digestive organ origin
	(C): League table of NMA of primary outcome: subgroup of respiratory and intrathoracic organ origin
	(D): League table of NMA of primary outcome: subgroup of bone origin
	(E): League table of NMA of primary outcome: subgroup of skin, mesothelium, soft tissue, and cartilage origin
	(F): League table of NMA of primary outcome: subgroup of breast and female genital organ origin
	(G): League table of NMA of primary outcome: subgroup of prostate and male genital organ origin
	(H): League table of NMA of primary outcome: subgroup of kidney and urinary tract origin
	(I): League table of NMA of primary outcome: subgroup of neuron, nerve, and neuroendocrine origin
	(J): League table of NMA of primary outcome: subgroup of thyroid and other endocrine gland origin

	(K): League table of NMA of safety profile: drop-out rate
Table S6	SUCRA (Surface under the cumulative ranking) of primary outcome: overall events of metastatic cancers
Table S7	(A) Side-splitting model inconsistency of primary outcome: overall events of metastatic cancers
	(B) Design-by-treatment model and loop inconsistency of primary outcome: overall events of metastatic cancers
Table S8	Heterogeneity of primary outcome: overall events of metastatic cancers
Table S9	GRADE of primary outcome: overall events of metastatic cancers

Figure S1A network structure of NMA of primary outcome: subgroup analysis of head, eyes, ears, nose, and throat origin

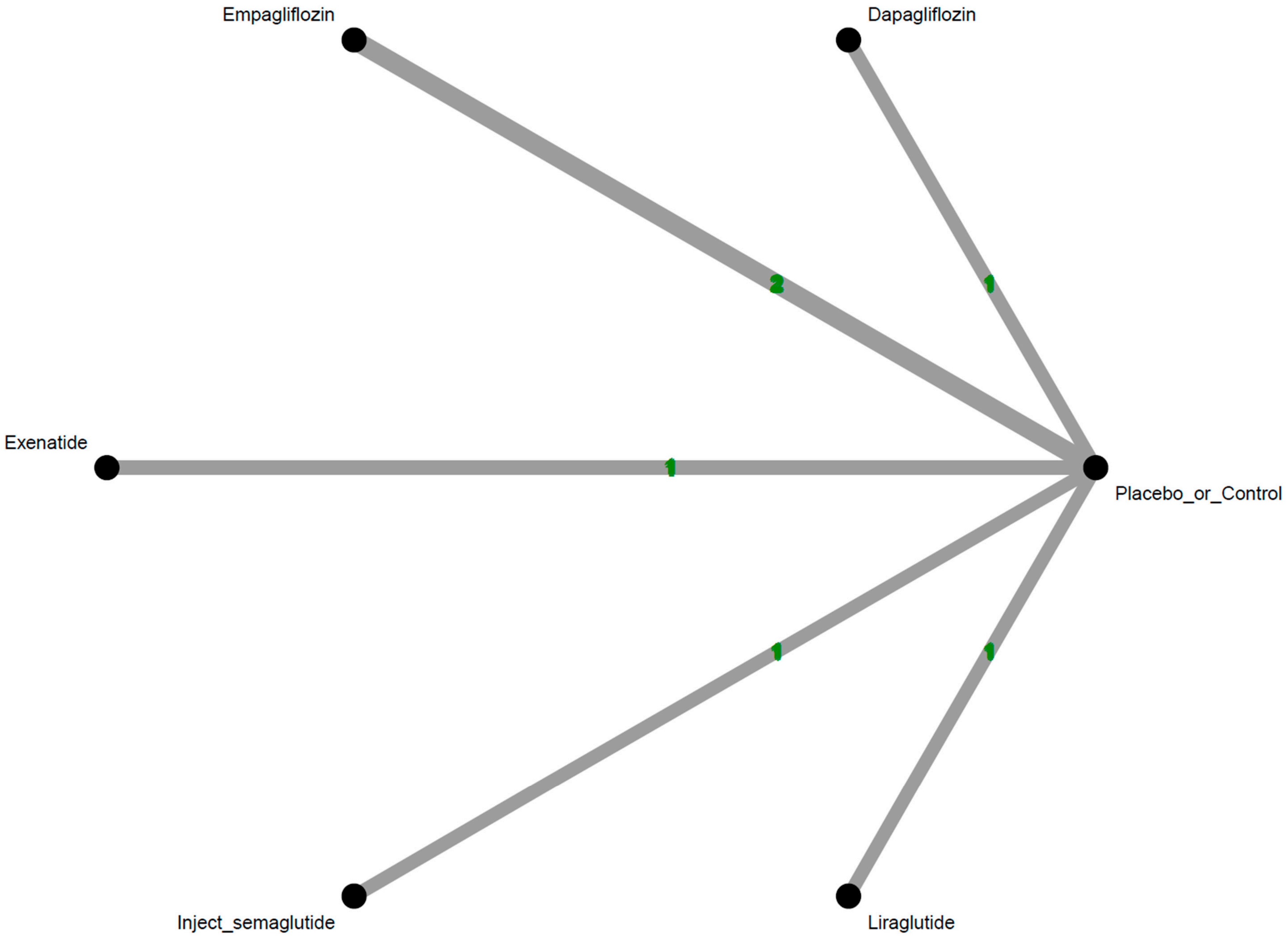

Figure S1B network structure of NMA of primary outcome: subgroup digestive organ origin

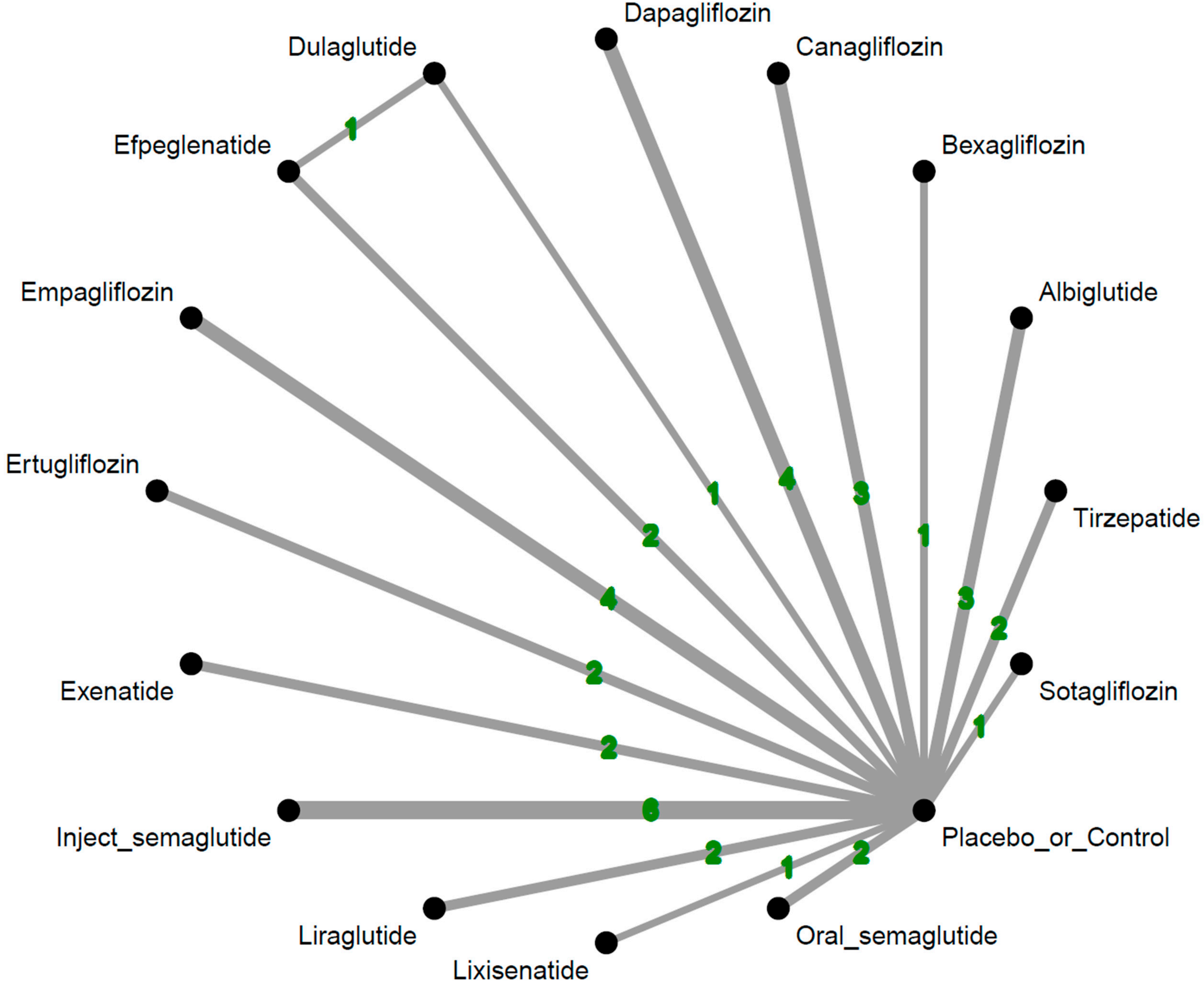

Figure S1C network structure of NMA of primary outcome: subgroup analysis of respiratory and intrathoracic organ origin

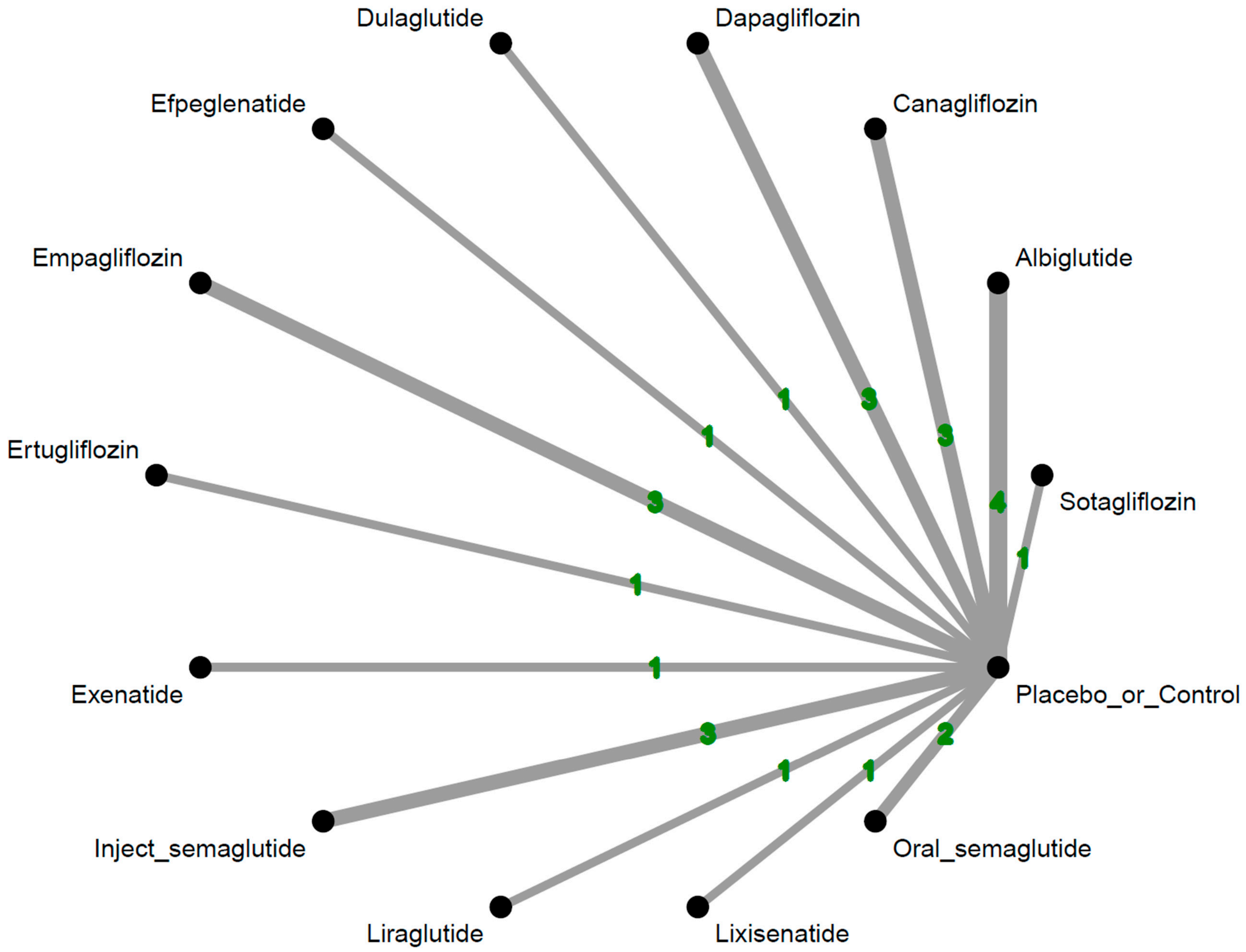

Figure S1D network structure of NMA of primary outcome: subgroup analysis of bone origin

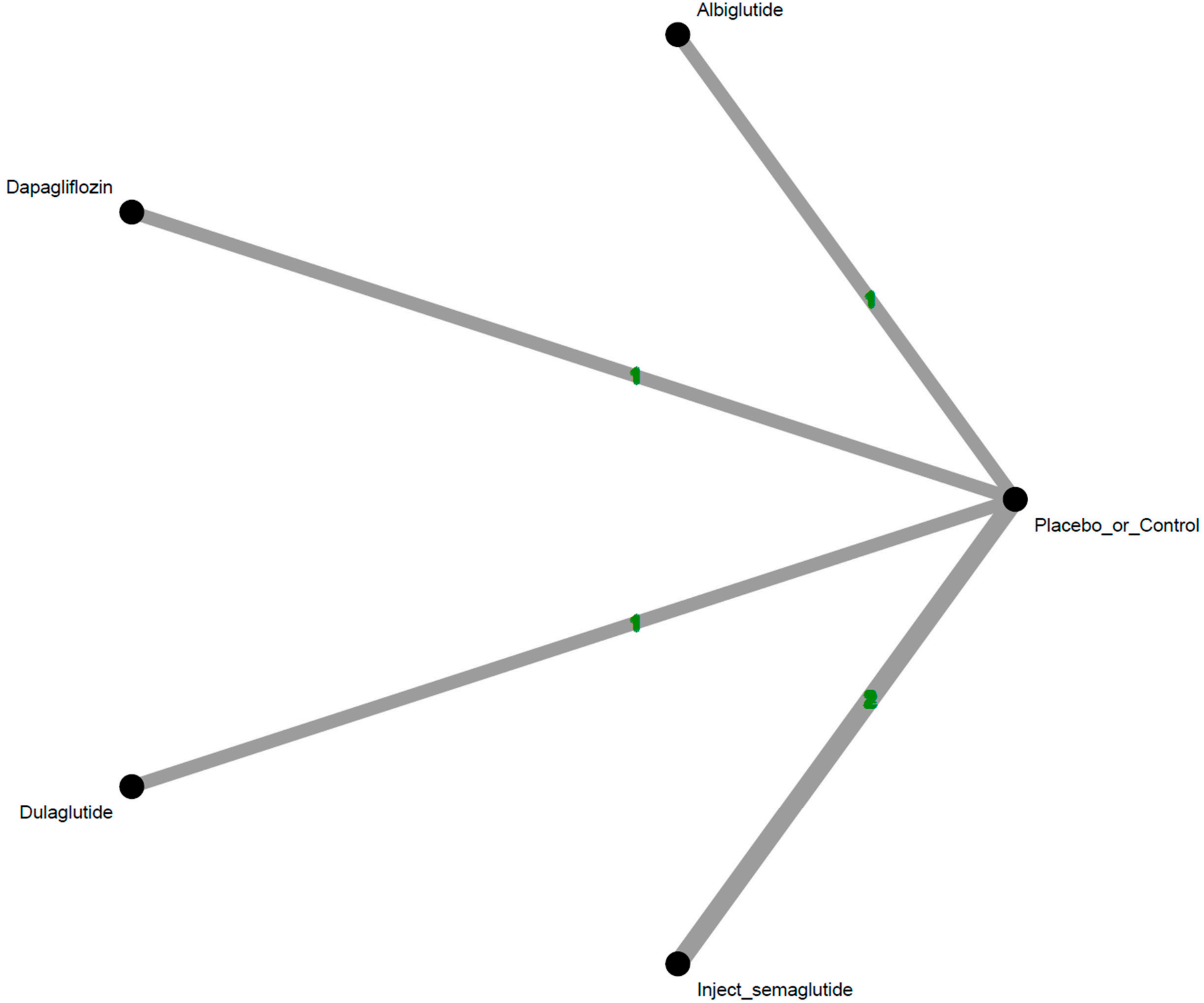

Figure S1E network structure of NMA of primary outcome: subgroup analysis of skin, mesothelium, soft tissue, and cartilage origin

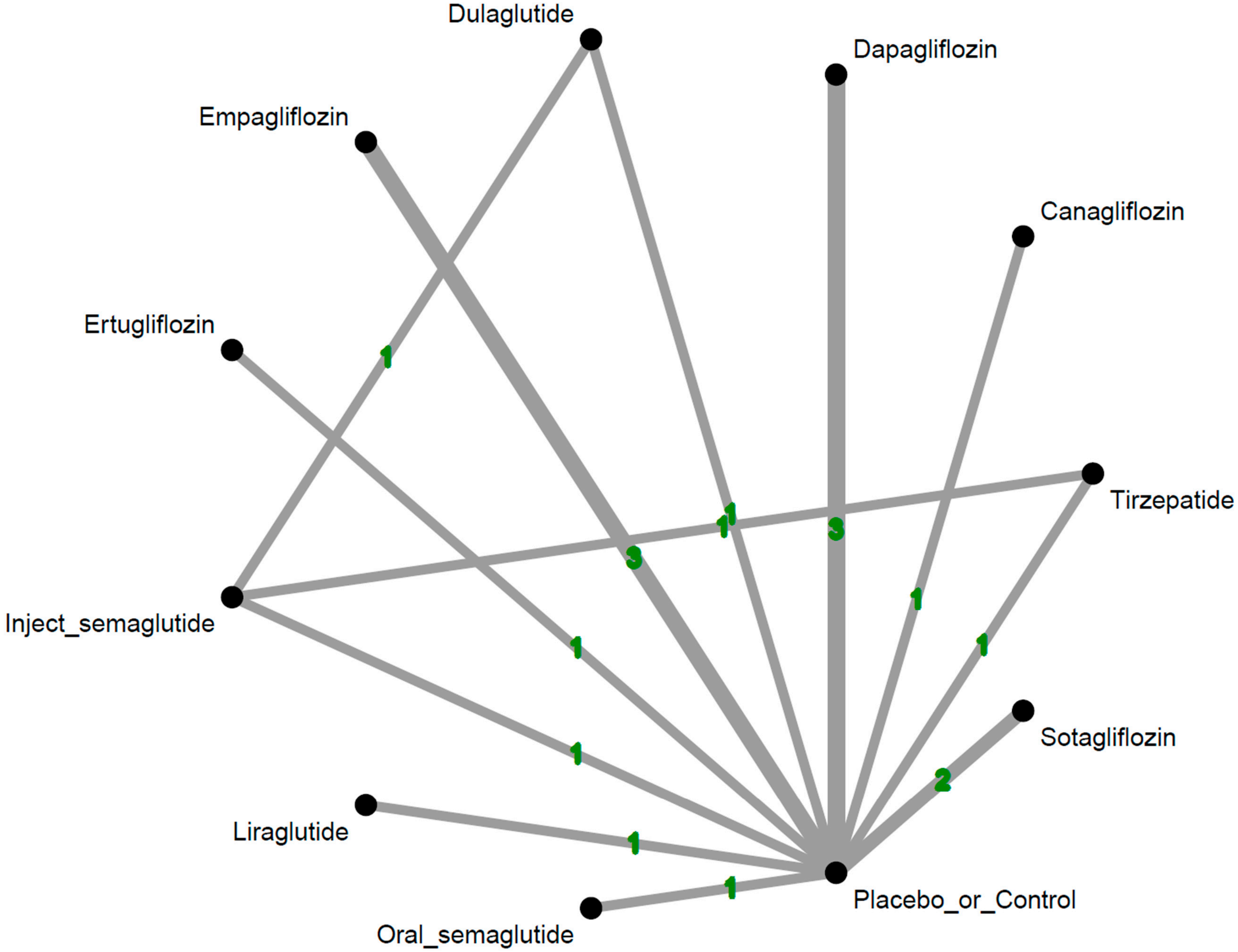

Figure S1F network structure of NMA of primary outcome: subgroup analysis of breast and female genital organ origin

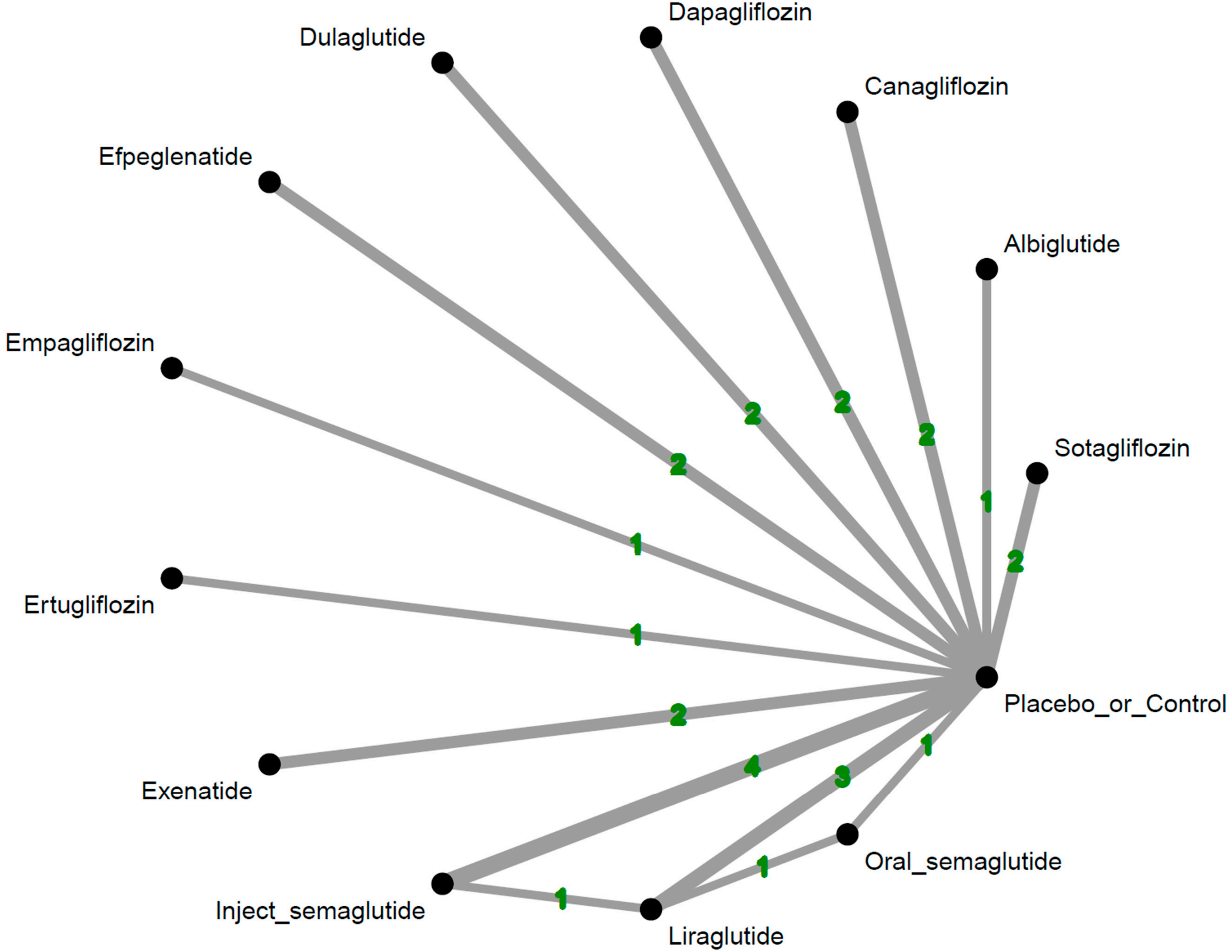

Figure S1G network structure of NMA of primary outcome: subgroup analysis of prostate and male genital organ origin

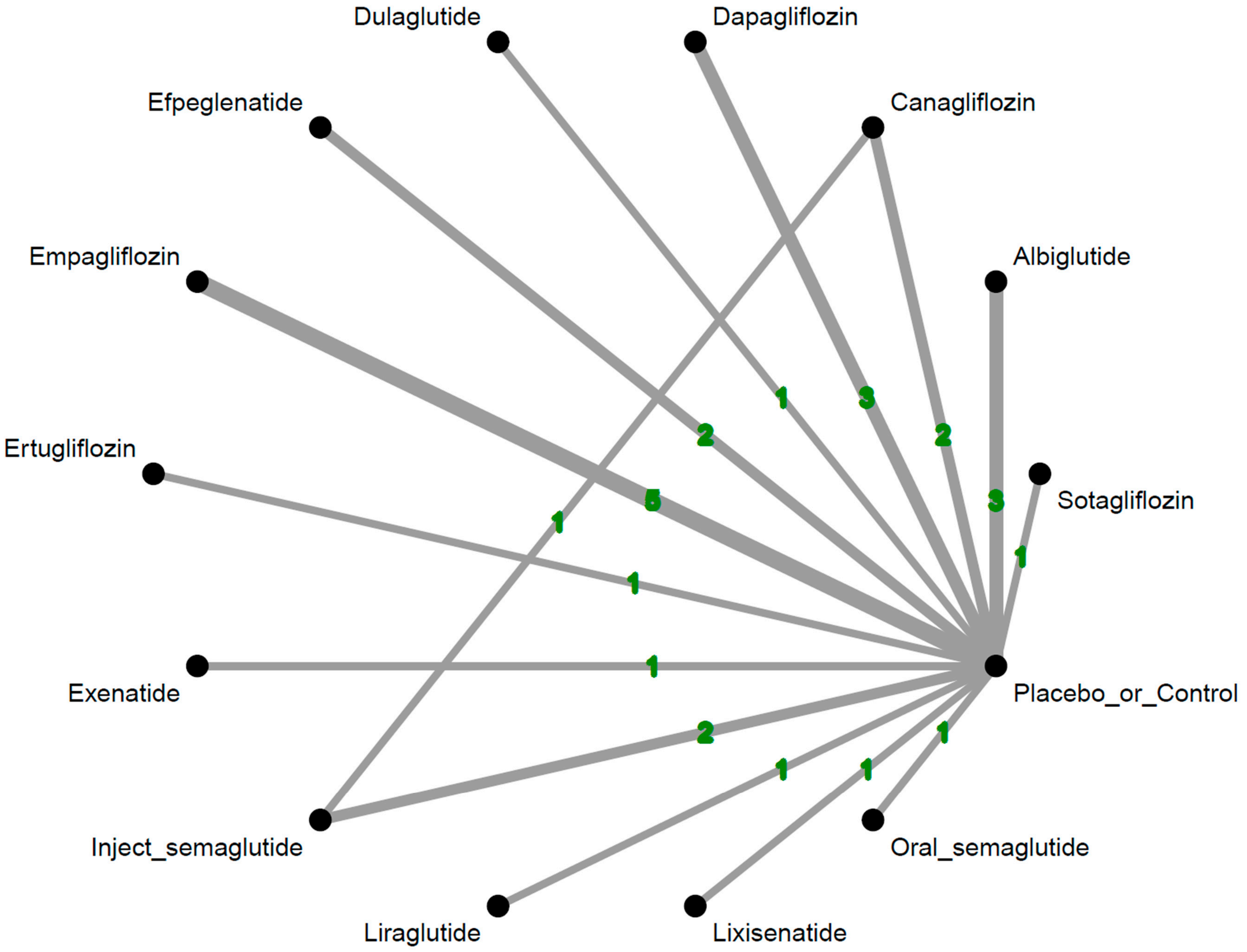

Figure S1H network structure of NMA of primary outcome: subgroup analysis of kidney and urinary tract origin

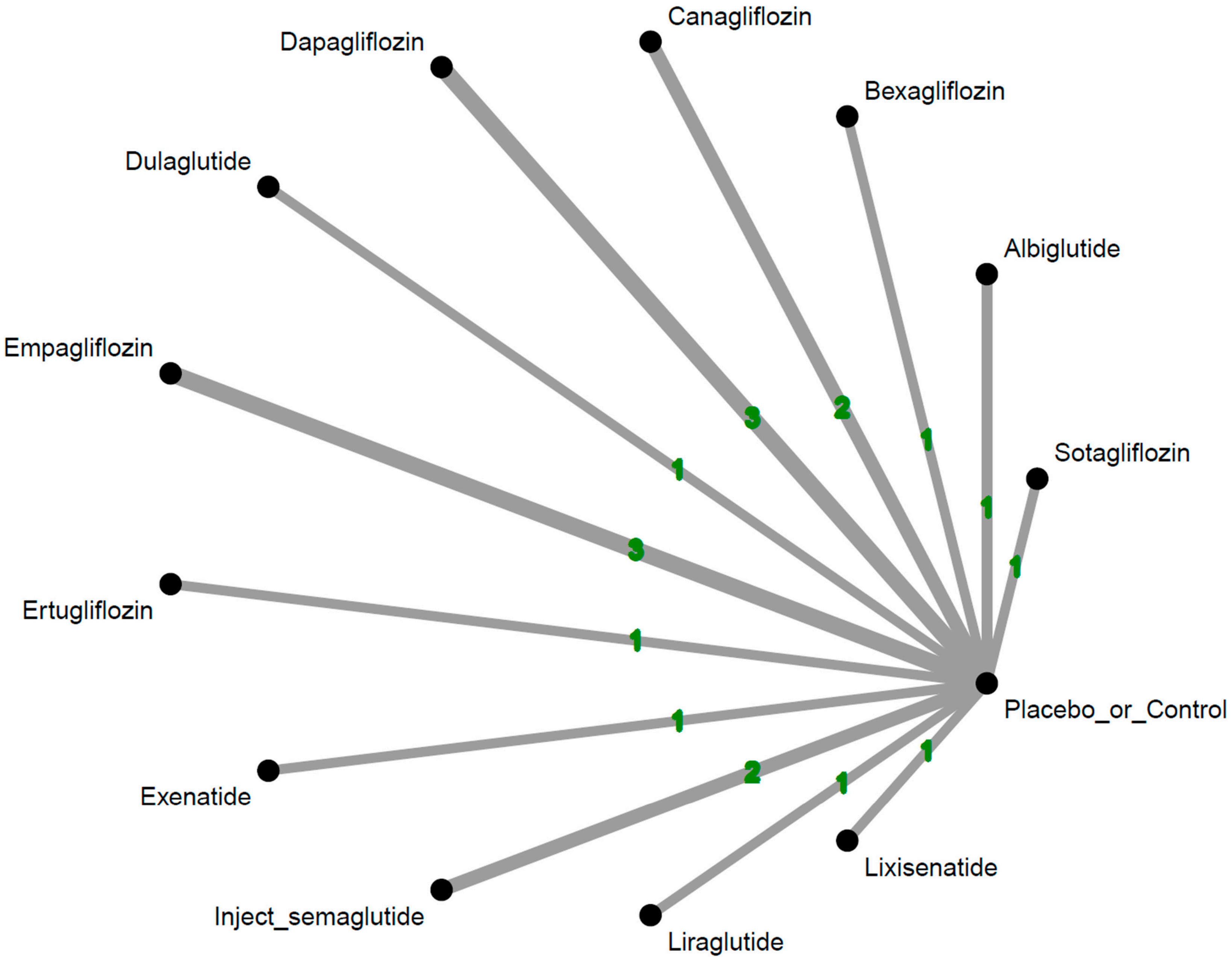

Figure S1I network structure of NMA of primary outcome: subgroup analysis of neuron, nerve, and neuroendocrine origin

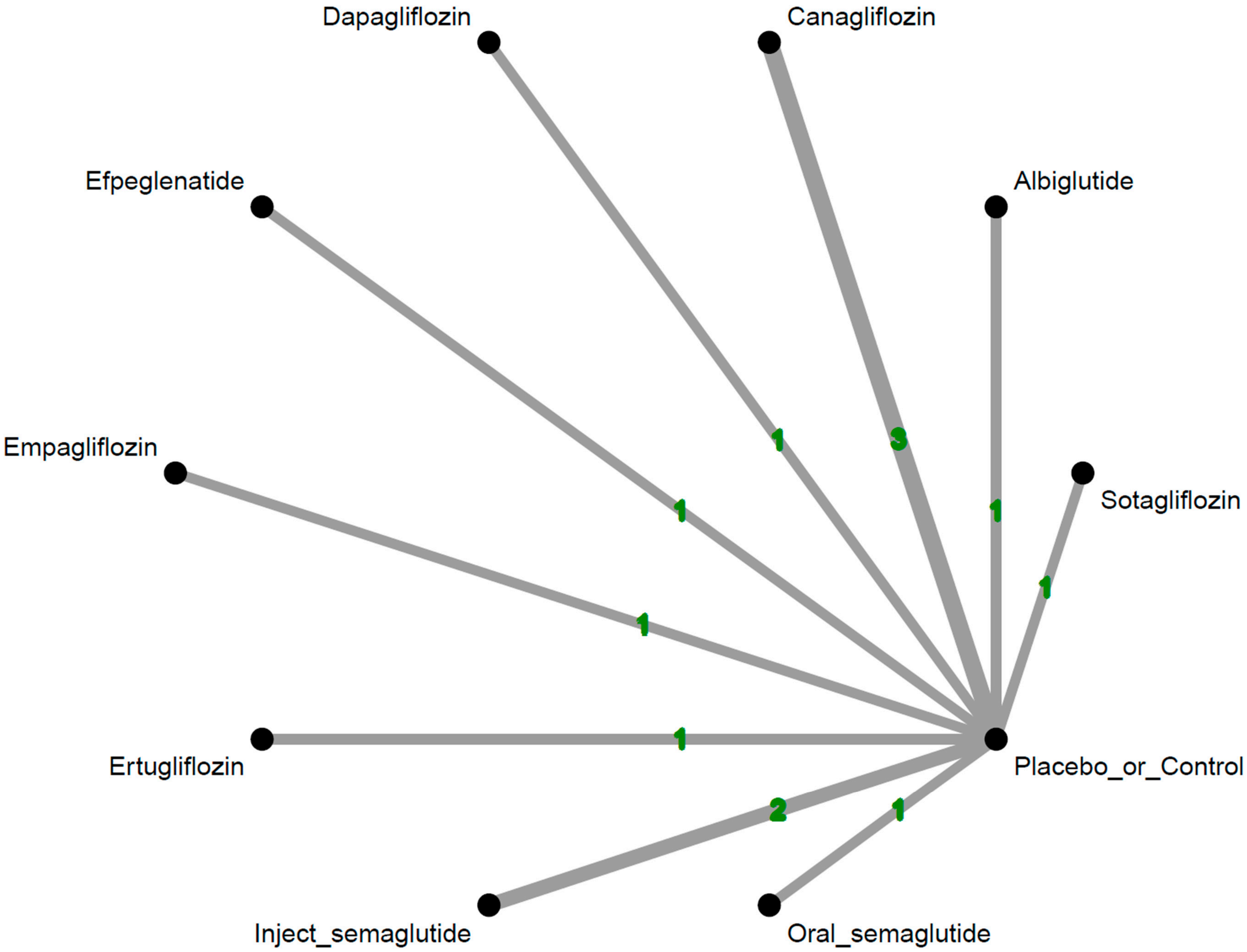

Figure S1J network structure of NMA of primary outcome: subgroup analysis of thyroid and other endocrine gland origin

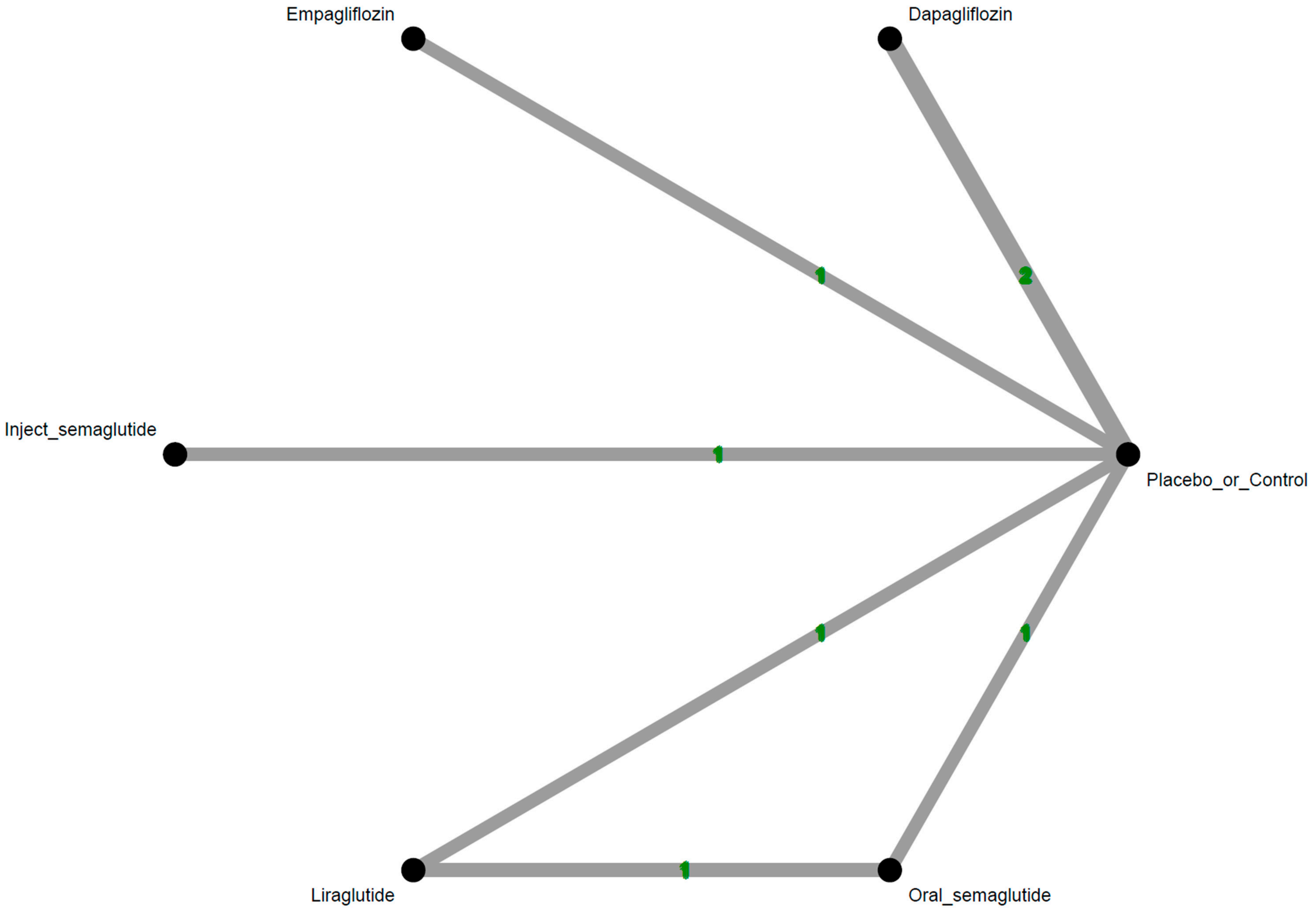

Figure S1K network structure of NMA of safety profile: drop-out rate

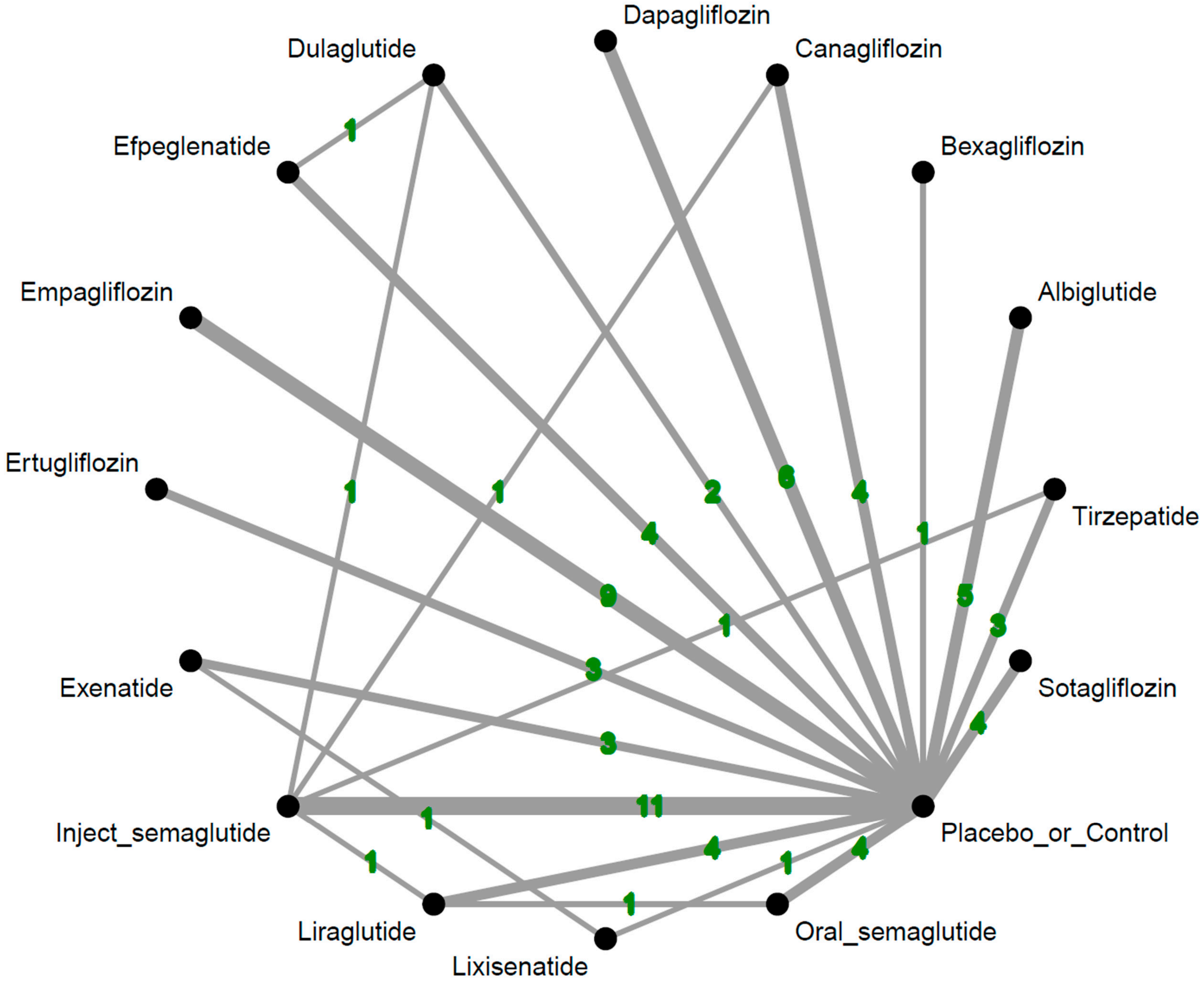

Figure legend of Figure S1A-1K:

The structure of the network meta-analysis. The lines between nodes represent direct comparisons from various trials, with the numbers over the lines indicating the number of trials providing these comparisons for each specific treatment. The thickness of the lines corresponds to the number of trials linked to the network.

Abbreviation for Figure S1A-1K:

95%CIs: 95% confidence intervals; GLP-1 agonist: glucagon-like peptide-1 agonist; NMA: network meta-analysis; OR: odds ratio; RCT: randomized controlled trial; SGLT2 inhibitor: sodium–glucose cotransporter 2 inhibitor

Figure S2A forest plot of NMA of primary outcome: subgroup analysis of head, eyes, ears, nose, and throat origin

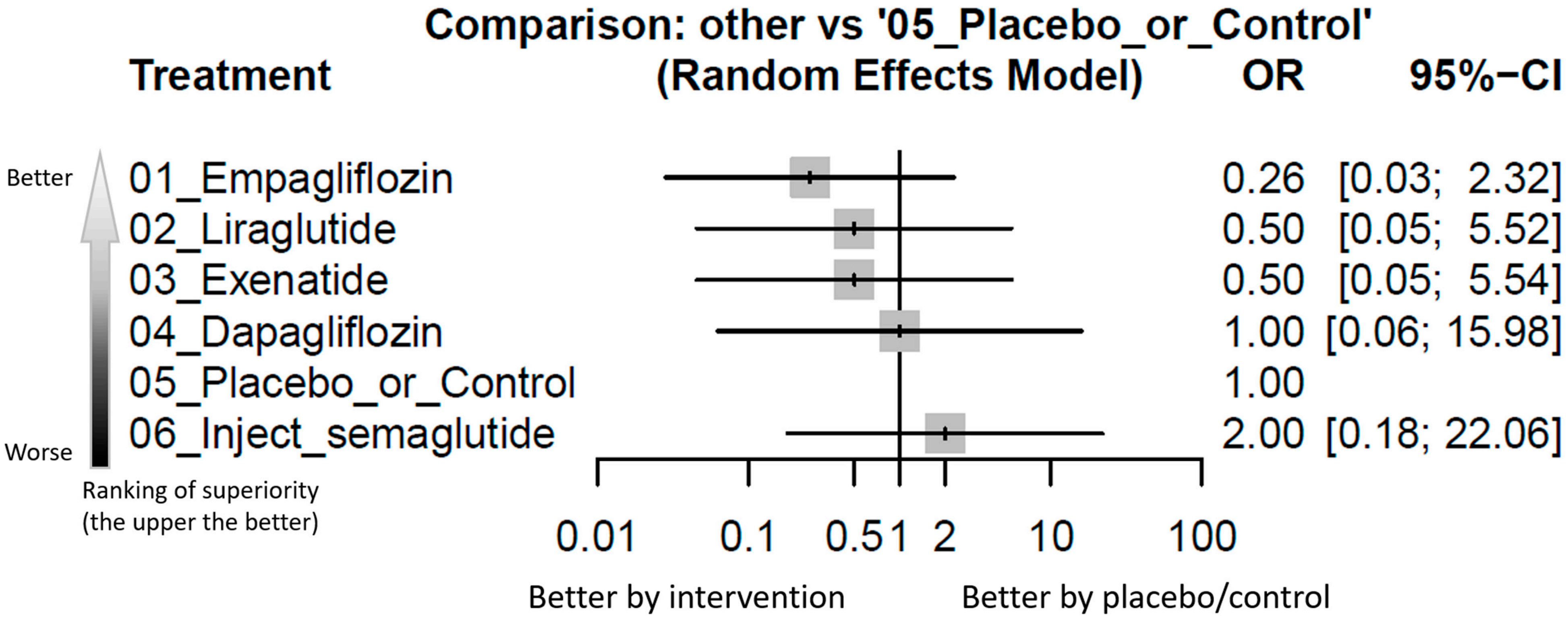

Figure S2B forest plot of NMA of primary outcome: subgroup digestive organ origin

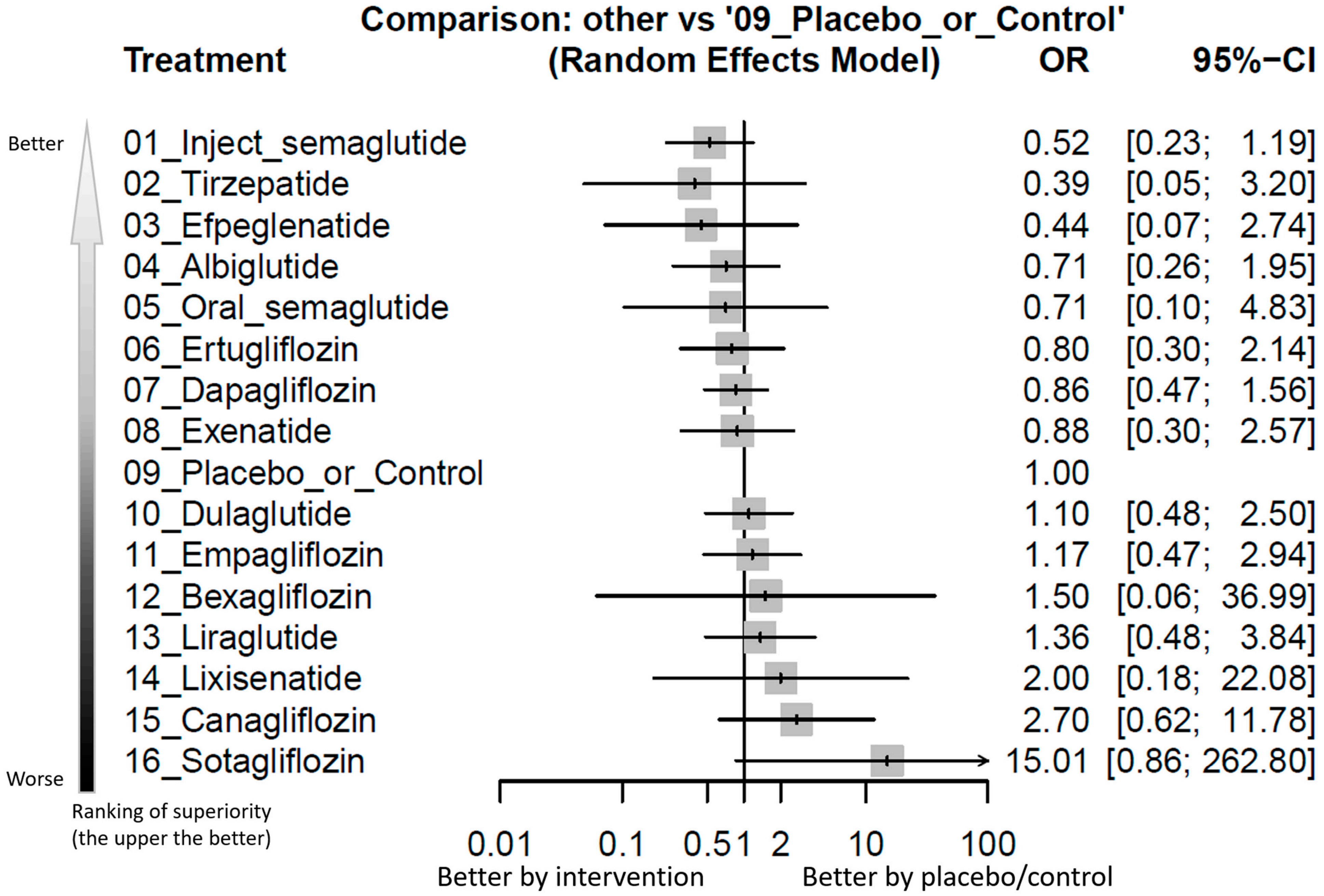

Figure S2C forest plot of NMA of primary outcome: subgroup analysis of respiratory and intrathoracic organ origin

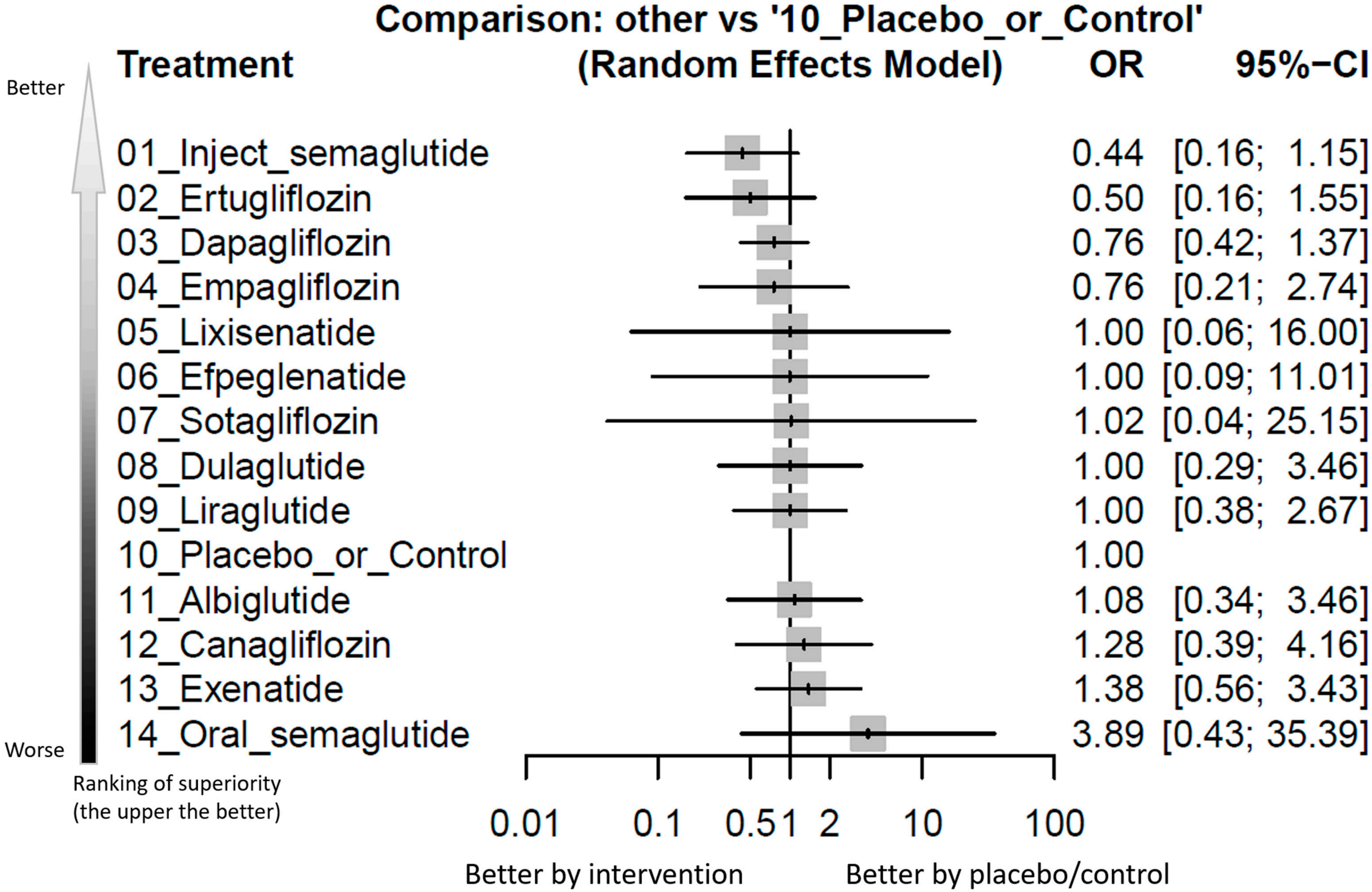

Figure S2D forest plot of NMA of primary outcome: subgroup analysis of bone origin

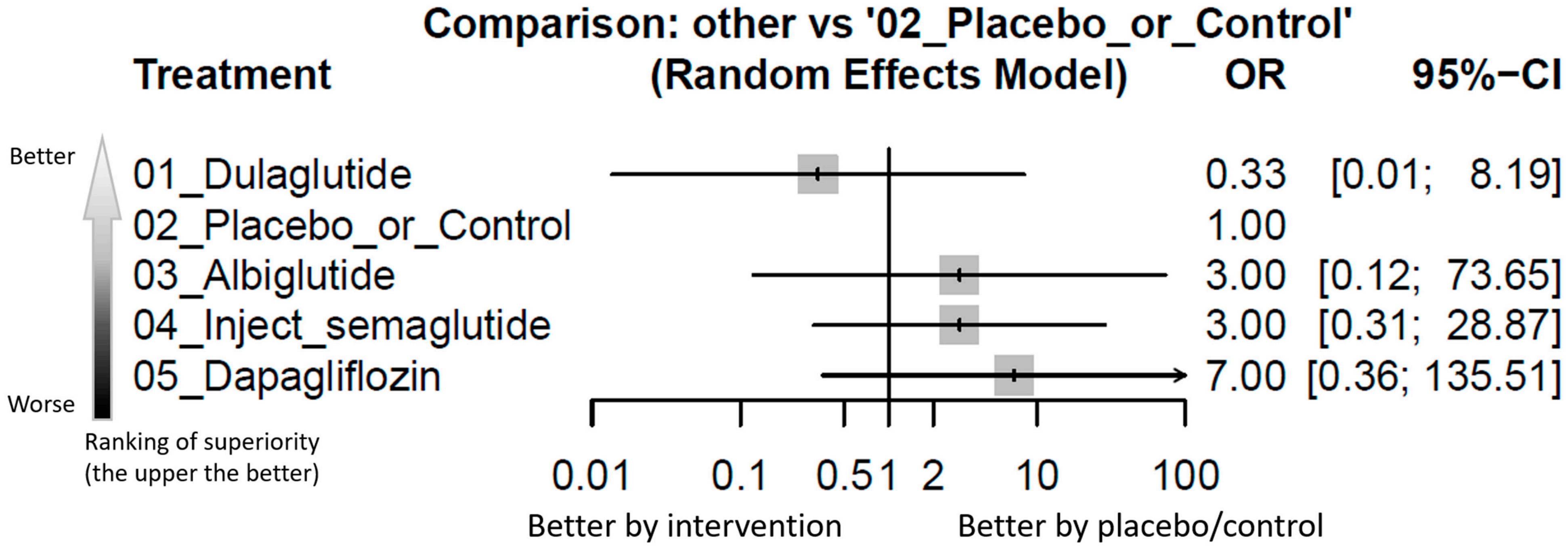

Figure S2E forest plot of NMA of primary outcome: subgroup analysis of skin, mesothelium, soft tissue, and cartilage origin

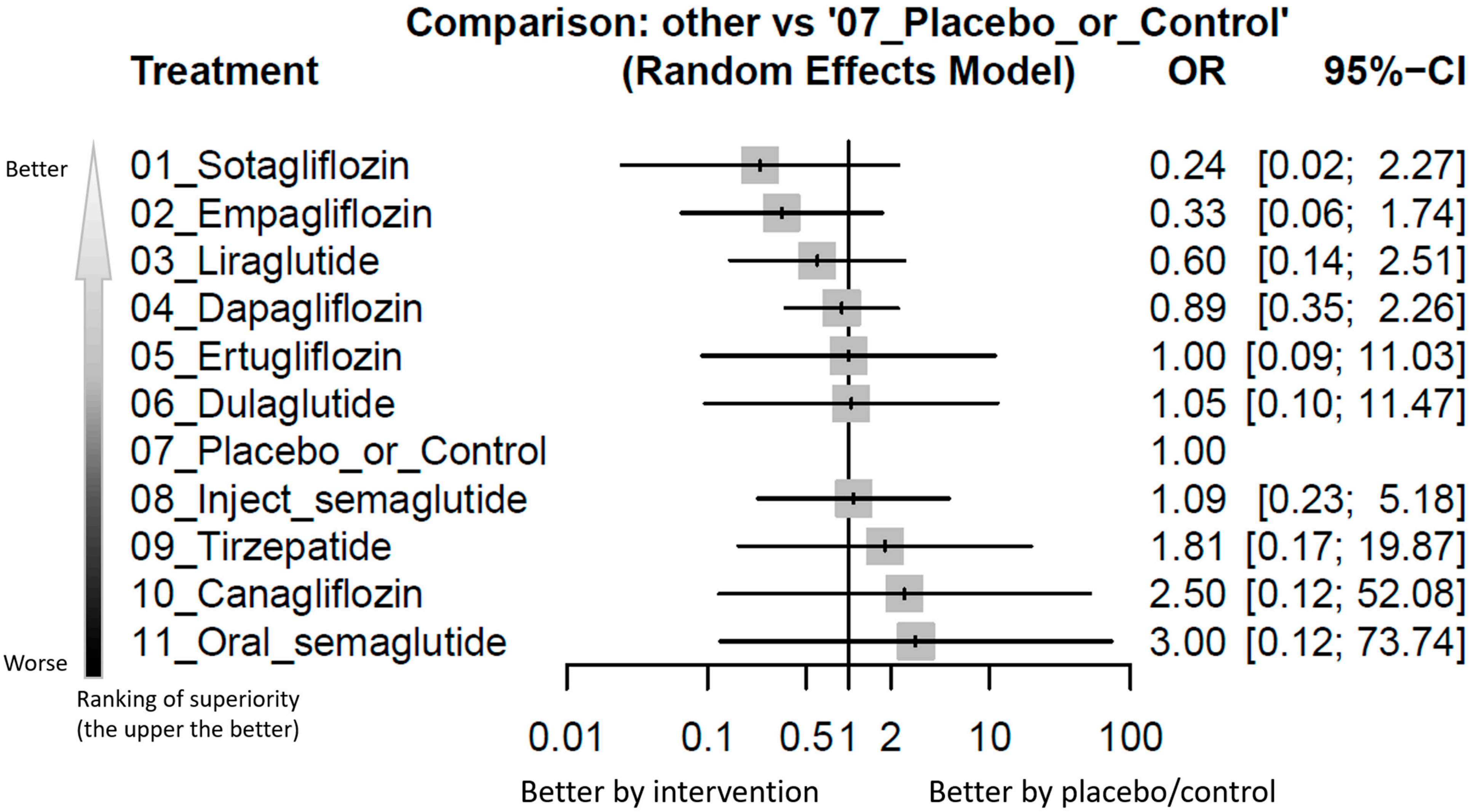

Figure S2F forest plot of NMA of primary outcome: subgroup analysis of breast and female genital organ origin

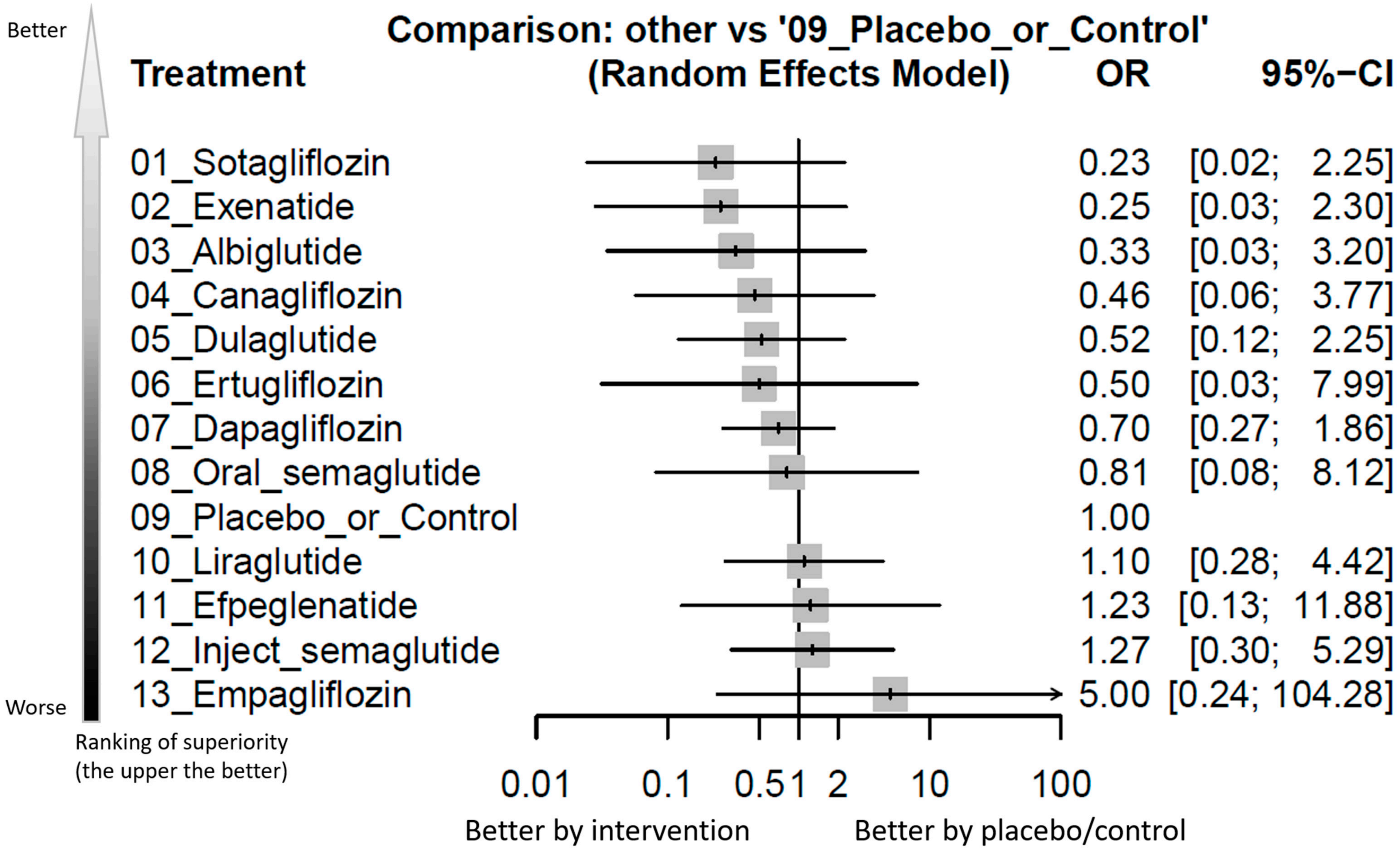

Figure S2G forest plot of NMA of primary outcome: subgroup analysis of prostate and male genital organ origin

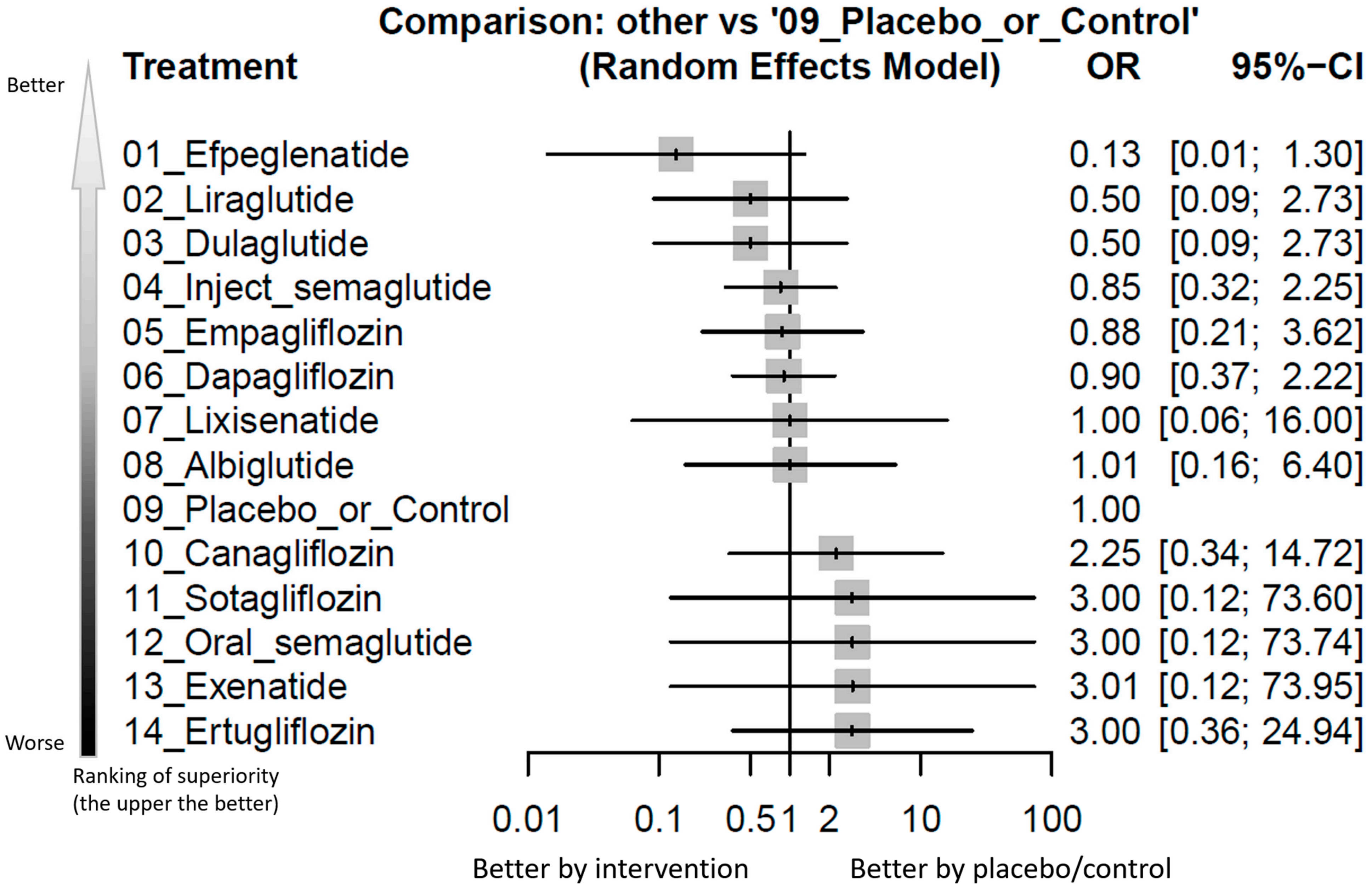

Figure S2H forest plot of NMA of primary outcome: subgroup analysis of kidney and urinary tract origin

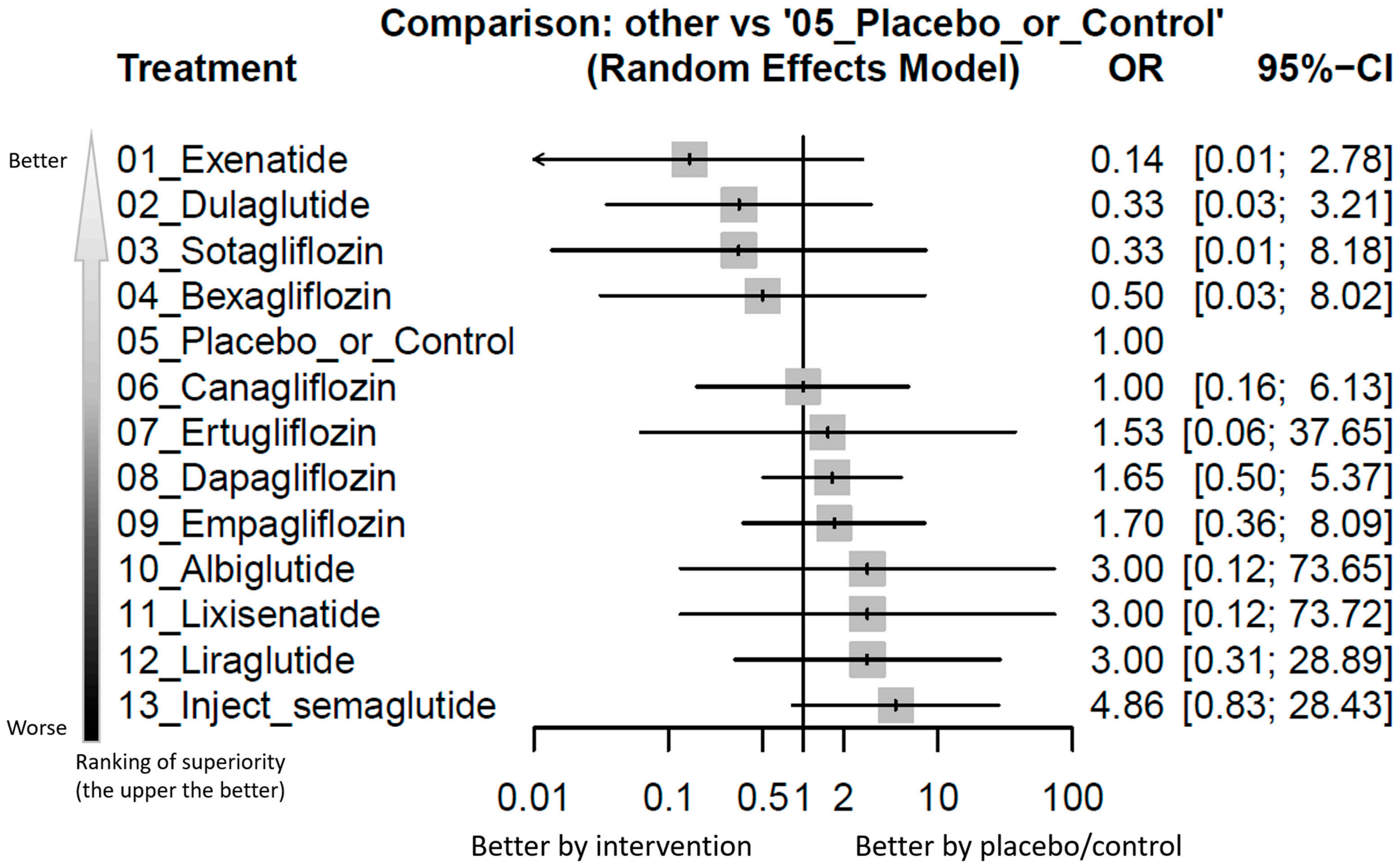

Figure S2I forest plot of NMA of primary outcome: subgroup analysis of neuron, nerve, and neuroendocrine origin

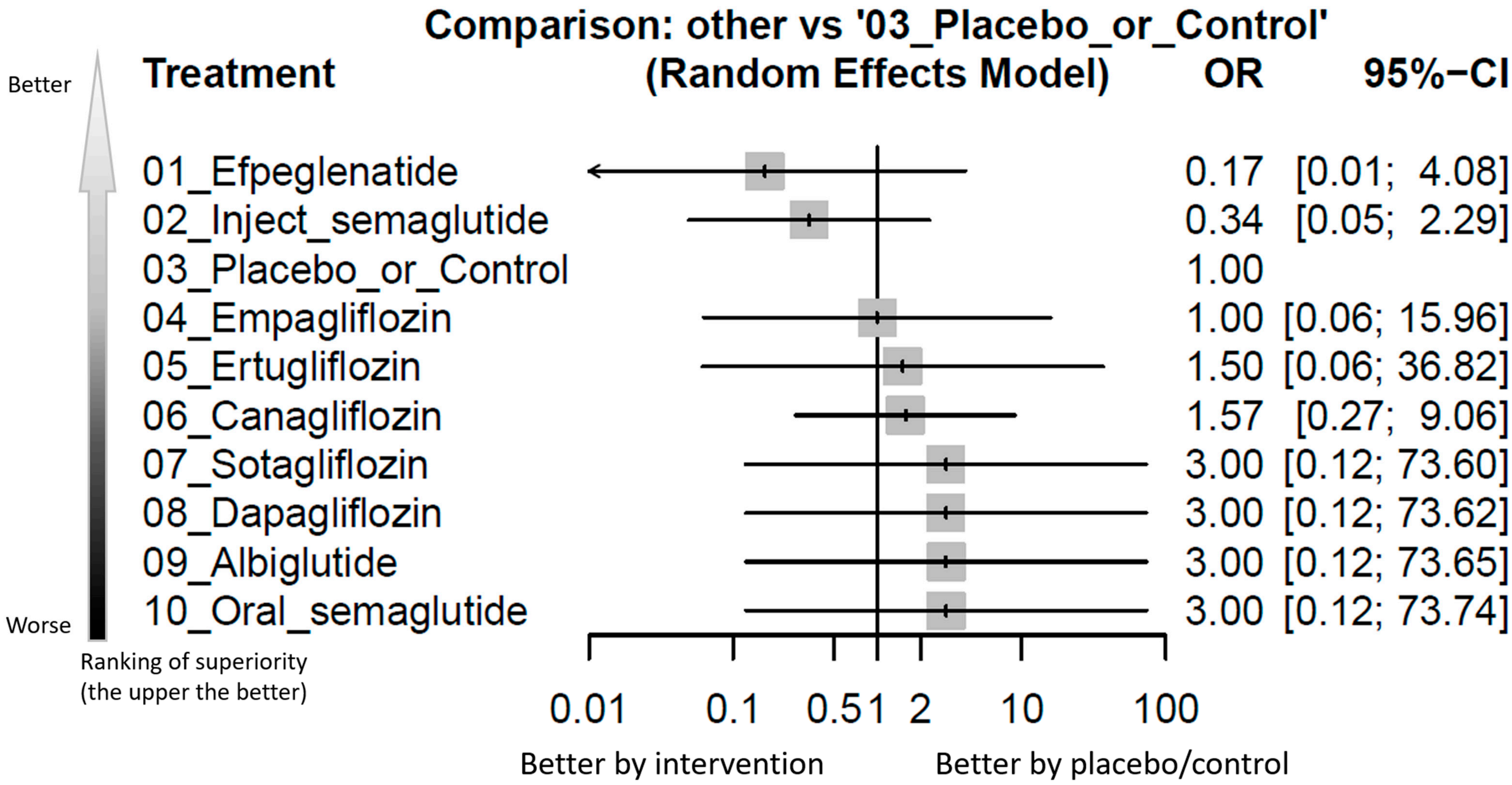

Figure S2J forest plot of NMA of primary outcome: subgroup analysis of thyroid and other endocrine gland origin

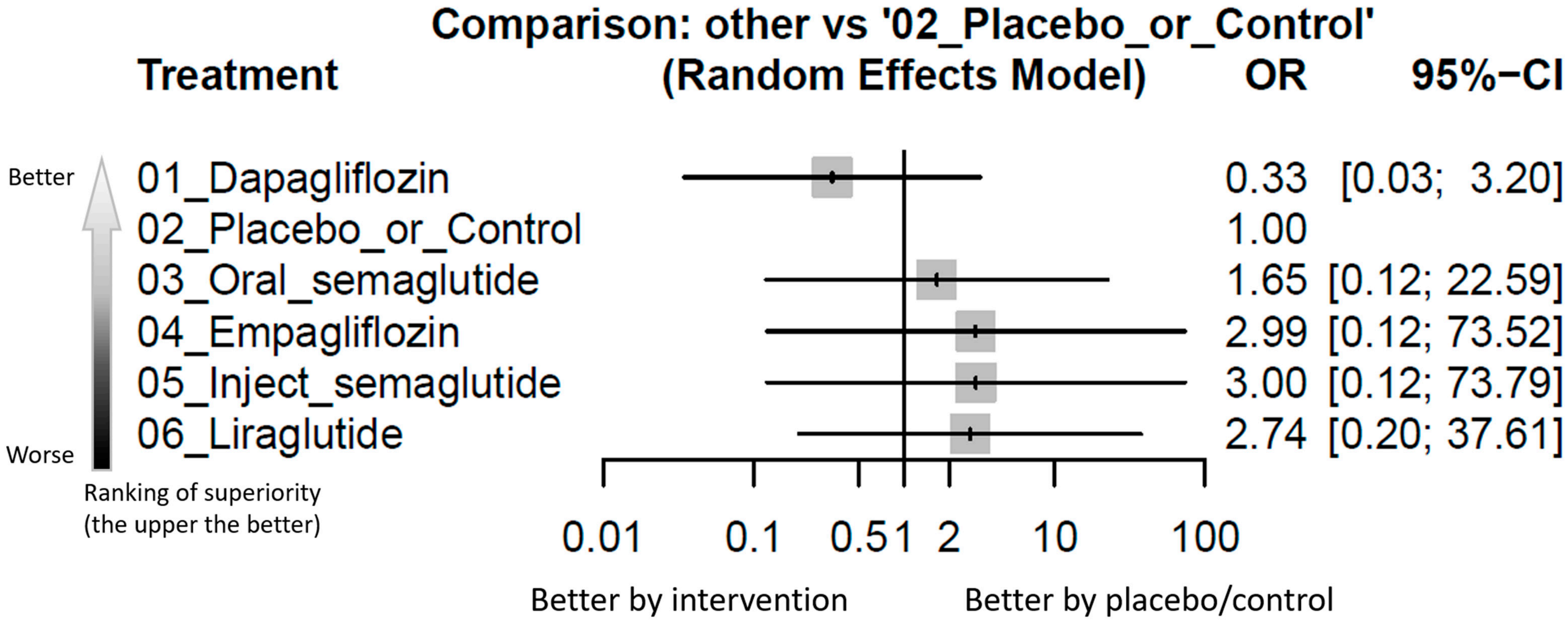

Figure S2K forest plot of NMA of safety profile: drop-out rate

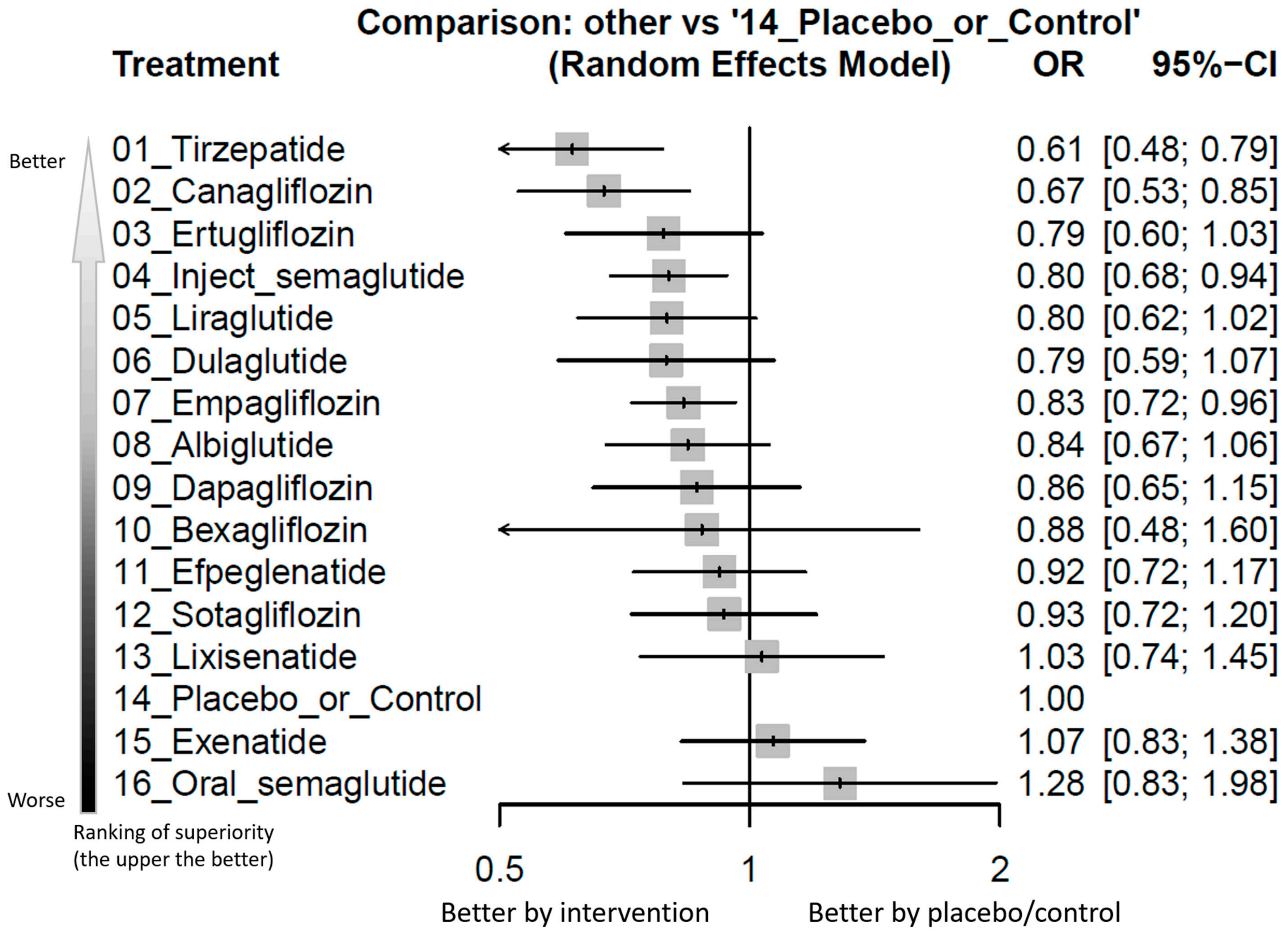

Abbreviation for Figure S2A-2K:

95%CIs: 95% confidence intervals; GLP-1 agonist: glucagon-like peptide-1 agonist; NMA: network meta-analysis; OR: odds ratio; RCT: randomized controlled trial; SGLT2 inhibitor: sodium–glucose cotransporter 2 inhibitor

Figure S3A Individual study result of primary outcome: overall events of metastatic cancers

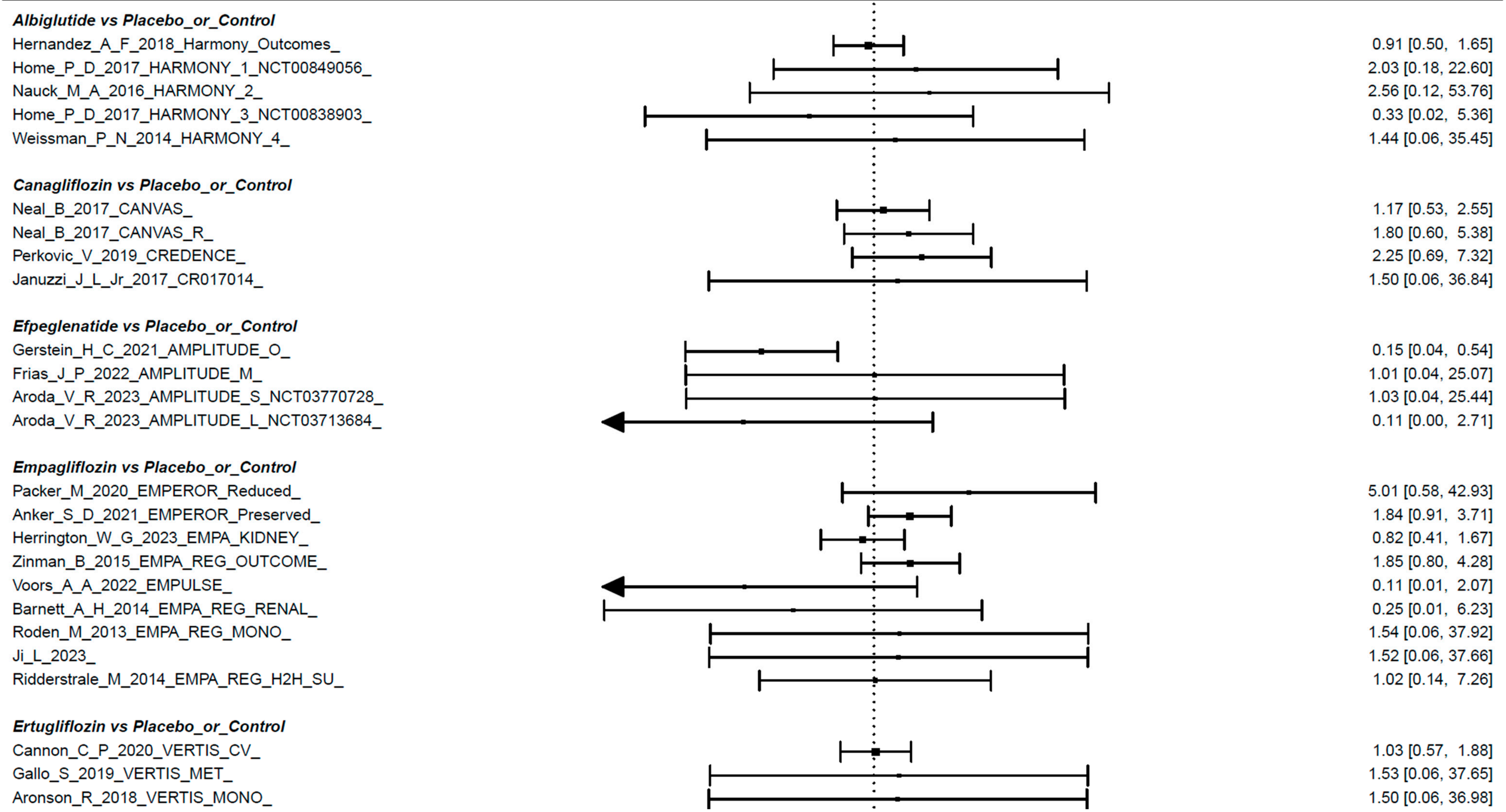

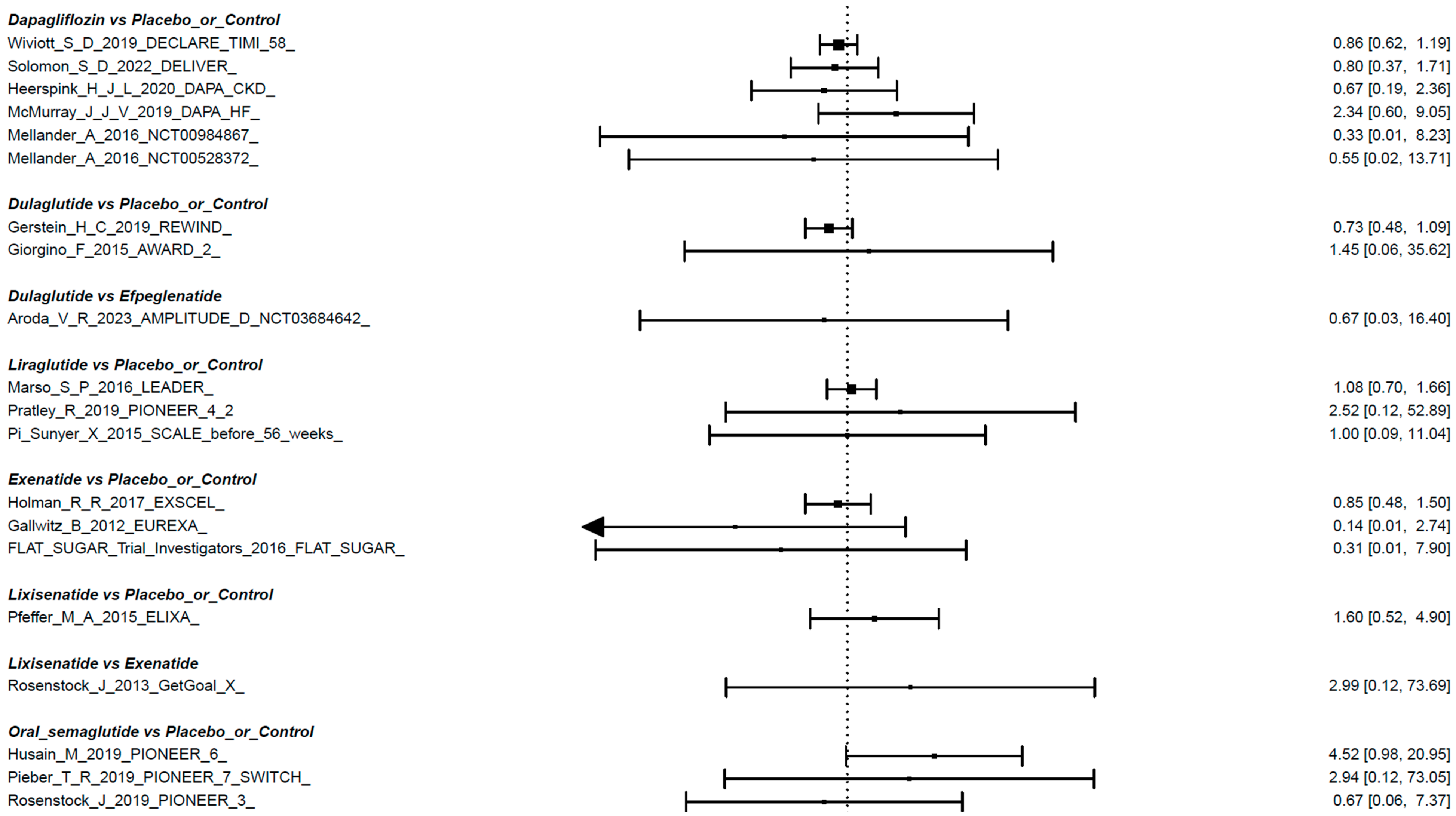
Dapagliflozin vs Placebo_or_Control
Wiviott_S_D_2019_DECLARE_TIMI_58_ 0.86 [0.62, 1.19]
Solomon_S_D_2022_DELIVER_ 0.80 [0.37, 1.71]
Heerspink_H_J_L_2020_DAPA_CKD_ 0.67 [0.19, 2.36]
McMurray_J_J_V_2019_DAPA_HF_ 2.34 [0.60, 9.05]
Mellander_A_2016_NCT00984867_ 0.33 [0.01, 8.23]
Mellander_A_2016_NCT00528372_ 0.55 [0.02, 13.71]
Dulaglutide vs Placebo_or_Control
Gerstein_H_C_2019_REWIND_ 0.73 [0.48, 1.09]
Giorgino_F_2015_AWARD_2_ 1.45 [0.06, 35.62]
Dulaglutide vs Efpeglenatide
Aroda_V_R_2023_AMPLITUDE_D_NCT03684642_ 0.67 [0.03, 16.40]
Liraglutide vs Placebo_or_Control
Marso_S_P_2016_LEADER_ 1.08 [0.70, 1.66]
Pratley_R_2019_PIONEER_4_2 2.52 [0.12, 52.89]
Pi_Sunyer_X_2015_SCALE_before_56_weeks_ 1.00 [0.09, 11.04]
Exenatide vs Placebo_or_Control
Holman_R_R_2017_EXSCEL_ 0.85 [0.48, 1.50]
Gallwitz_B_2012_EUREXA_ 0.14 [0.01, 2.74]
FLAT_SUGAR_Trial_Investigators_2016_FLAT_SUGAR_ 0.31 [0.01, 7.90]
Lixisenatide vs Placebo_or_Control
Pfeffer_M_A_2015_ELIXA_ 1.60 [0.52, 4.90]
Lixisenatide vs Exenatide
Rosenstock_J_2013_GetGoal_X_ 2.99 [0.12, 73.69]
Oral_semaglutide vs Placebo_or_Control
Husain_M_2019_PIONEER_6_ 4.52 [0.98, 20.95]
Pieber_T_R_2019_PIONEER_7_SWITCH_ 2.94 [0.12, 73.05]
Rosenstock_J_2019_PIONEER_3_ 0.67 [0.06, 7.37]

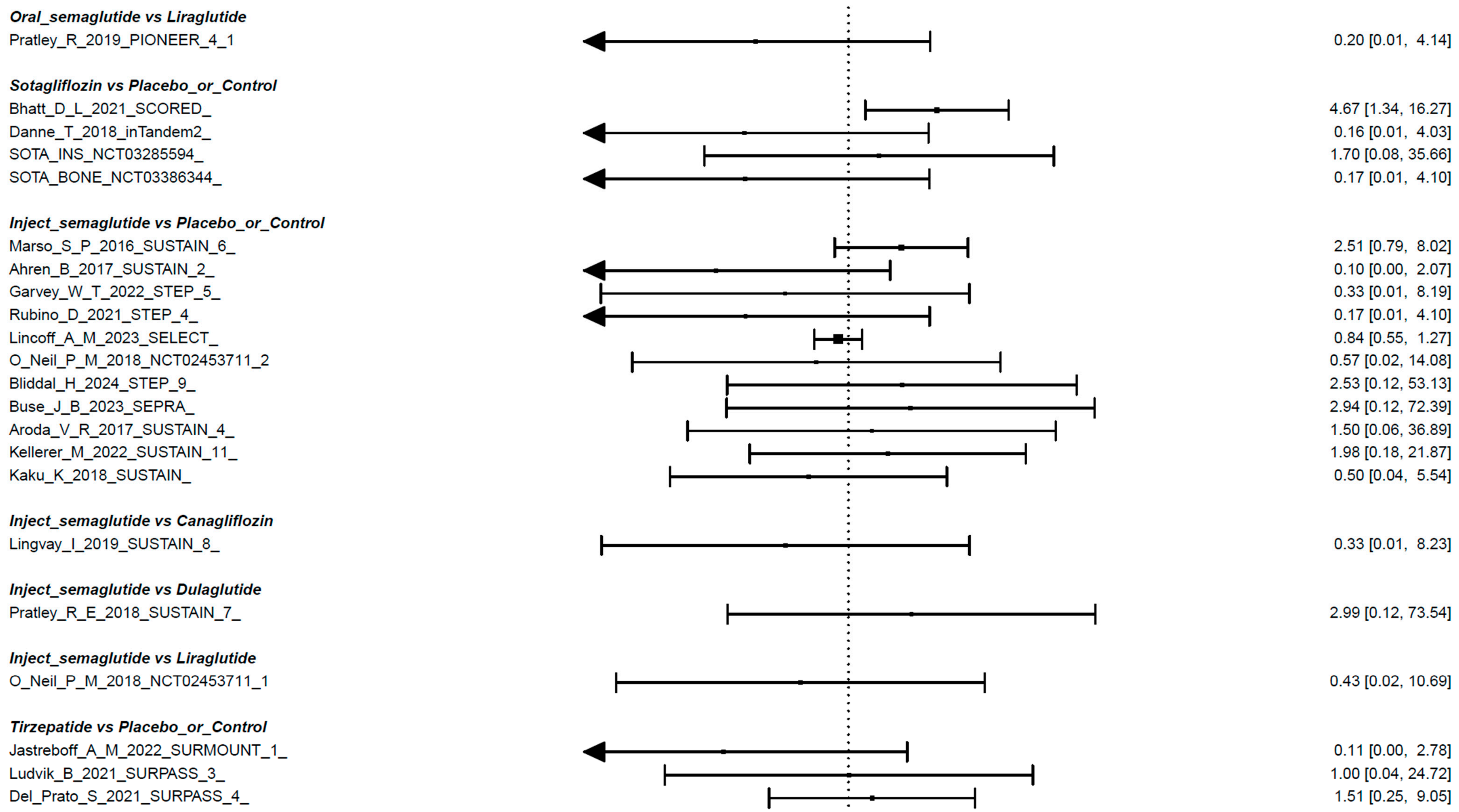

Oral_semaglutide vs Liraglutide
Pratley_R_2019_PIONEER_4_1
0.20 [0.01, 4.14]
Sotagliflozin vs Placebo_or_Control
Bhatt_D_L_2021_SCORED_
4.67 [1.34, 16.27]
Danne_T_2018_inTandem2_
0.16 [0.01, 4.03]
SOTA_INS_NCT03285594_
1.70 [0.08, 35.66]
SOTA_BONE_NCT03386344_
0.17 [0.01, 4.10]
Inject_semaglutide vs Placebo_or_Control
Marso_S_P_2016_SUSTAIN_6_
2.51 [0.79, 8.02]
Ahren_B_2017_SUSTAIN_2_
0.10 [0.00, 2.07]
Garvey_W_T_2022_STEP_5_
0.33 [0.01, 8.19]
Rubino_D_2021_STEP_4_
0.17 [0.01, 4.10]
Lincoff_A_M_2023_SELECT_
0.84 [0.55, 1.27]
O_Neil_P_M_2018_NCT02453711_2
0.57 [0.02, 14.08]
Bliddal_H_2024_STEP_9_
2.53 [0.12, 53.13]
Buse_J_B_2023_SEPRA_
2.94 [0.12, 72.39]
Aroda_V_R_2017_SUSTAIN_4_
1.50 [0.06, 36.89]
Kellerer_M_2022_SUSTAIN_11_
1.98 [0.18, 21.87]
Kaku_K_2018_SUSTAIN_
0.50 [0.04, 5.54]
Inject_semaglutide vs Canagliflozin
Lingvay_I_2019_SUSTAIN_8_
0.33 [0.01, 8.23]
Inject_semaglutide vs Dulaglutide
Pratley_R_E_2018_SUSTAIN_7_
2.99 [0.12, 73.54]
Inject_semaglutide vs Liraglutide
O_Neil_P_M_2018_NCT02453711_1
0.43 [0.02, 10.69]
Tirzepatide vs Placebo_or_Control
Jastreboff_A_M_2022_SURMOUNT_1_
0.11 [0.00, 2.78]
Ludvik_B_2021_SURPASS_3_
1.00 [0.04, 24.72]
Del_Prato_S_2021_SURPASS_4_
1.51 [0.25, 9.05]

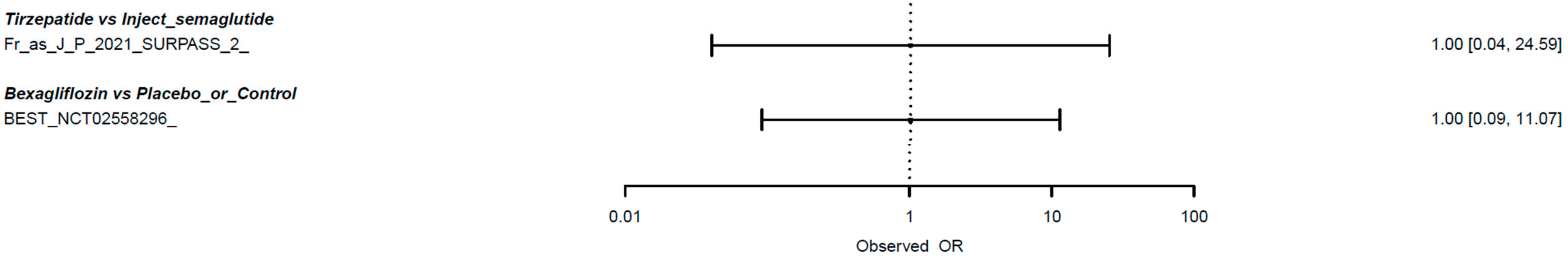
Tirzepatide vs Inject_semaglutide
Fr_as_J_P_2021_SURPASS_2_
1.00 [0.04, 24.59]
Bexagliflozin vs Placebo_or_Control
BEST_NCT02558296_
1.00 [0.09, 11.07]
0.01
1
10
100
Observed OR

Figure S3B Individual study result of primary outcome: subgroup analysis of head, eyes, ears, nose, and throat origin

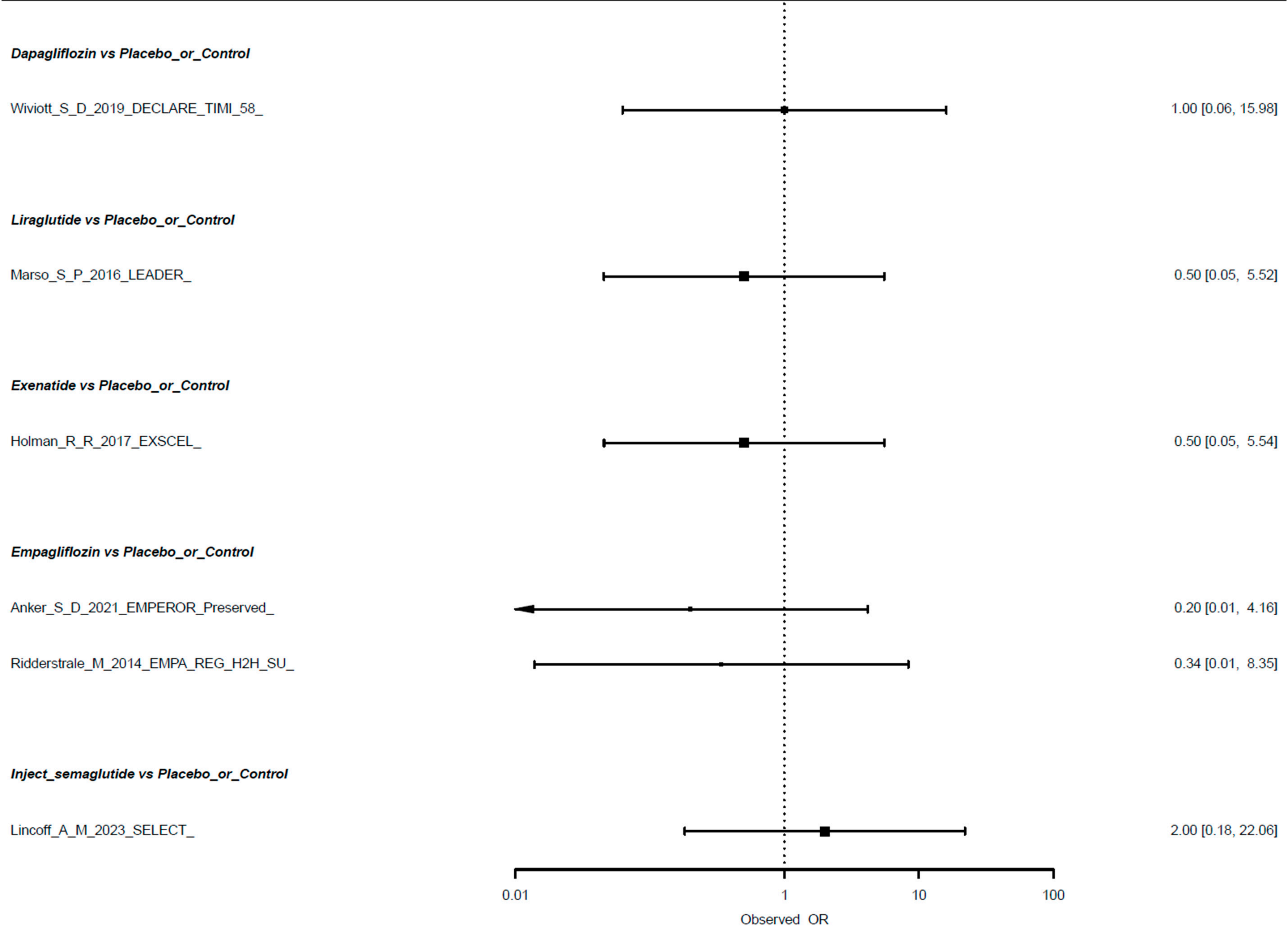

Figure S3C Individual study result of primary outcome: subgroup digestive organ origin

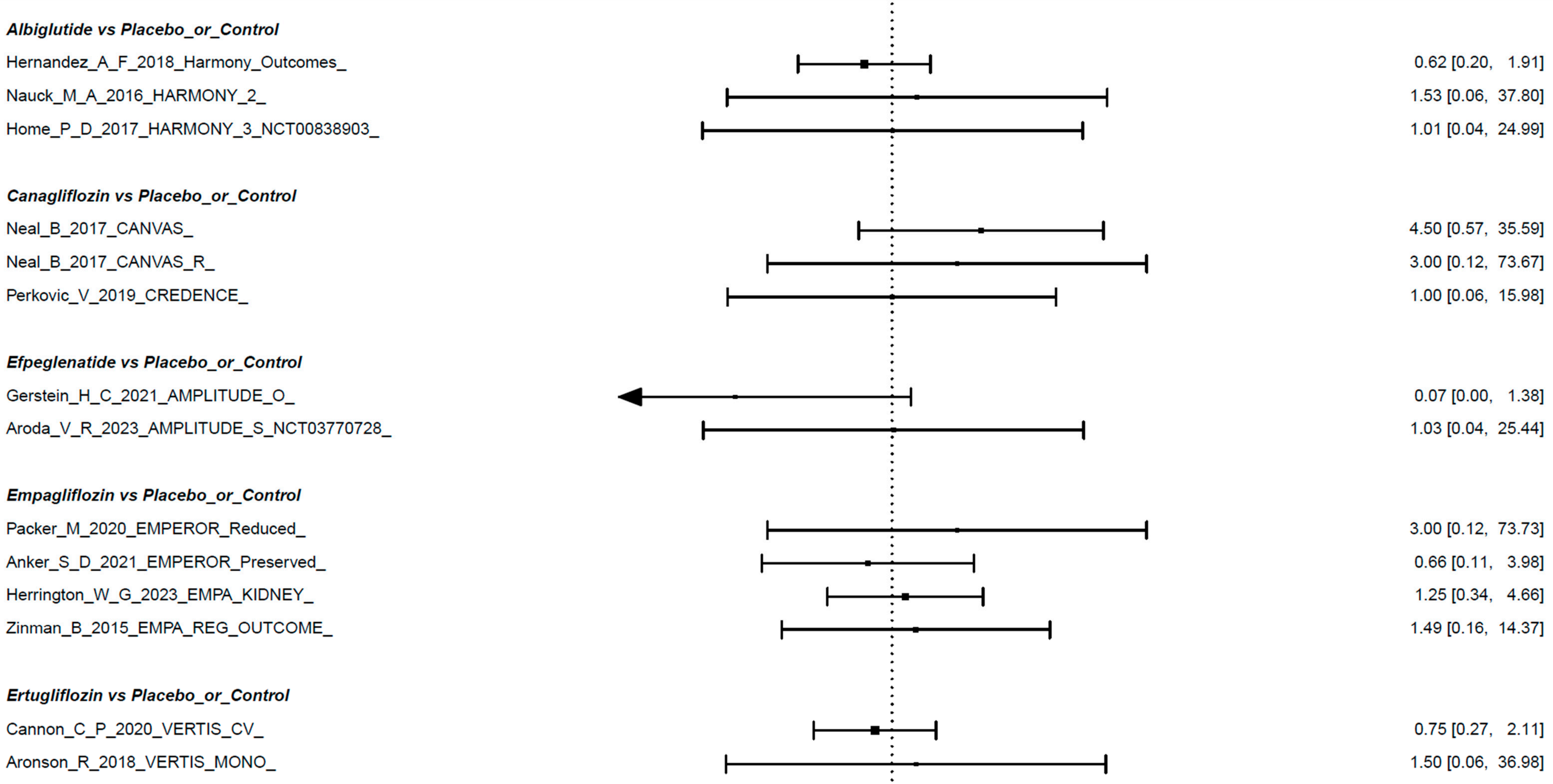

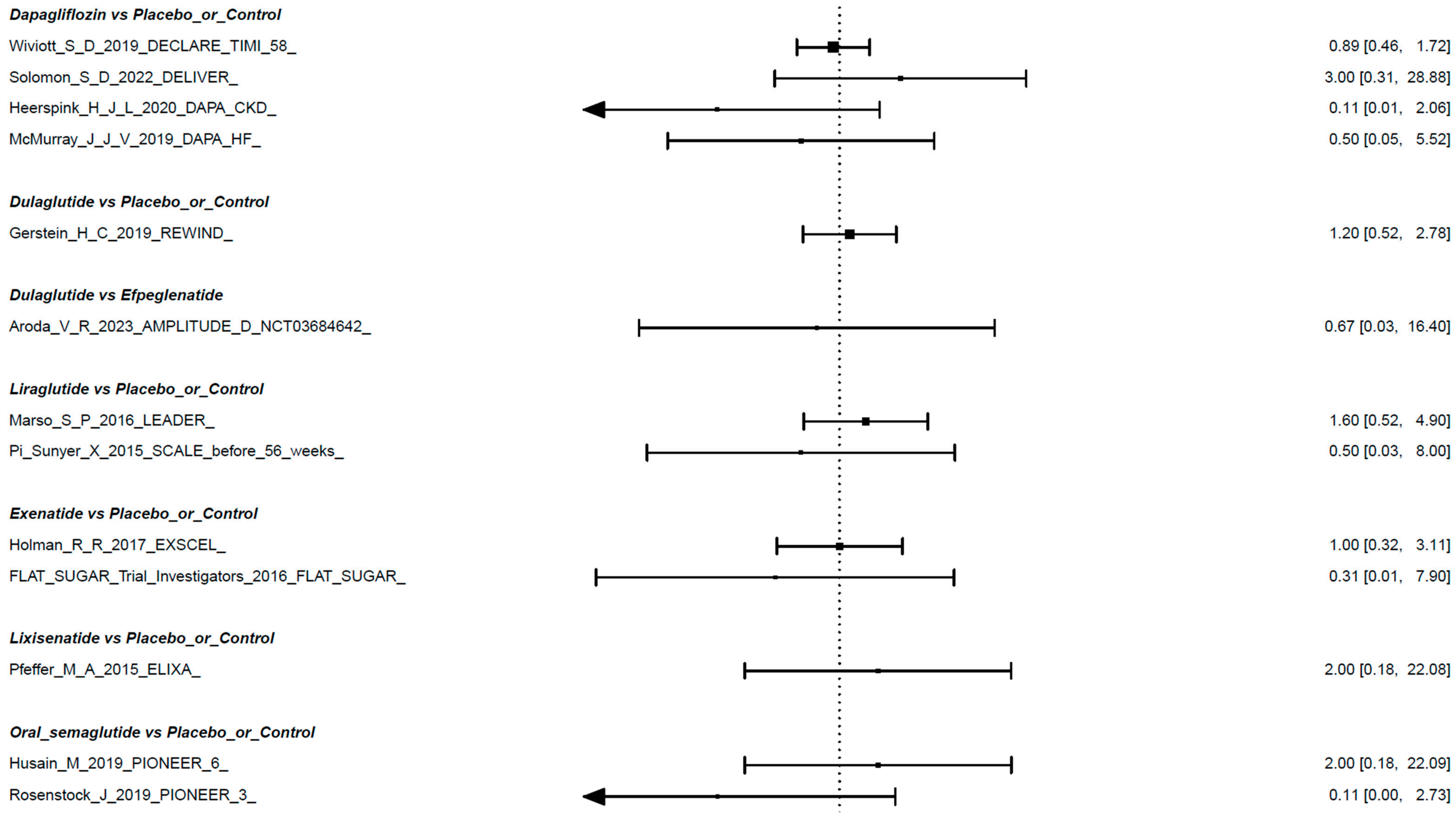
Dapagliflozin vs Placebo_or_Control
Wiviott_S_D_2019_DECLARE_TIMI_58_ 0.89 [0.46, 1.72]
Solomon_S_D_2022_DELIVER_ 3.00 [0.31, 28.88]
Heerspink_H_J_L_2020_DAPA_CKD_ 0.11 [0.01, 2.06]
McMurray_J_J_V_2019_DAPA_HF_ 0.50 [0.05, 5.52]
Dulaglutide vs Placebo_or_Control
Gerstein_H_C_2019_REWIND_ 1.20 [0.52, 2.78]
Dulaglutide vs Efpeglenatide
Aroda_V_R_2023_AMPLITUDE_D_NCT03684642_ 0.67 [0.03, 16.40]
Liraglutide vs Placebo_or_Control
Marso_S_P_2016_LEADER_ 1.60 [0.52, 4.90]
Pi_Sunyer_X_2015_SCALE_before_56_weeks_ 0.50 [0.03, 8.00]
Exenatide vs Placebo_or_Control
Holman_R_R_2017_EXSCEL_ 1.00 [0.32, 3.11]
FLAT_SUGAR_Trial_Investigators_2016_FLAT_SUGAR_ 0.31 [0.01, 7.90]
Lixisenatide vs Placebo_or_Control
Pfeffer_M_A_2015_ELIXA_ 2.00 [0.18, 22.08]
Oral_semaglutide vs Placebo_or_Control
Husain_M_2019_PIONEER_6_ 2.00 [0.18, 22.09]
Rosenstock_J_2019_PIONEER_3_ 0.11 [0.00, 2.73]

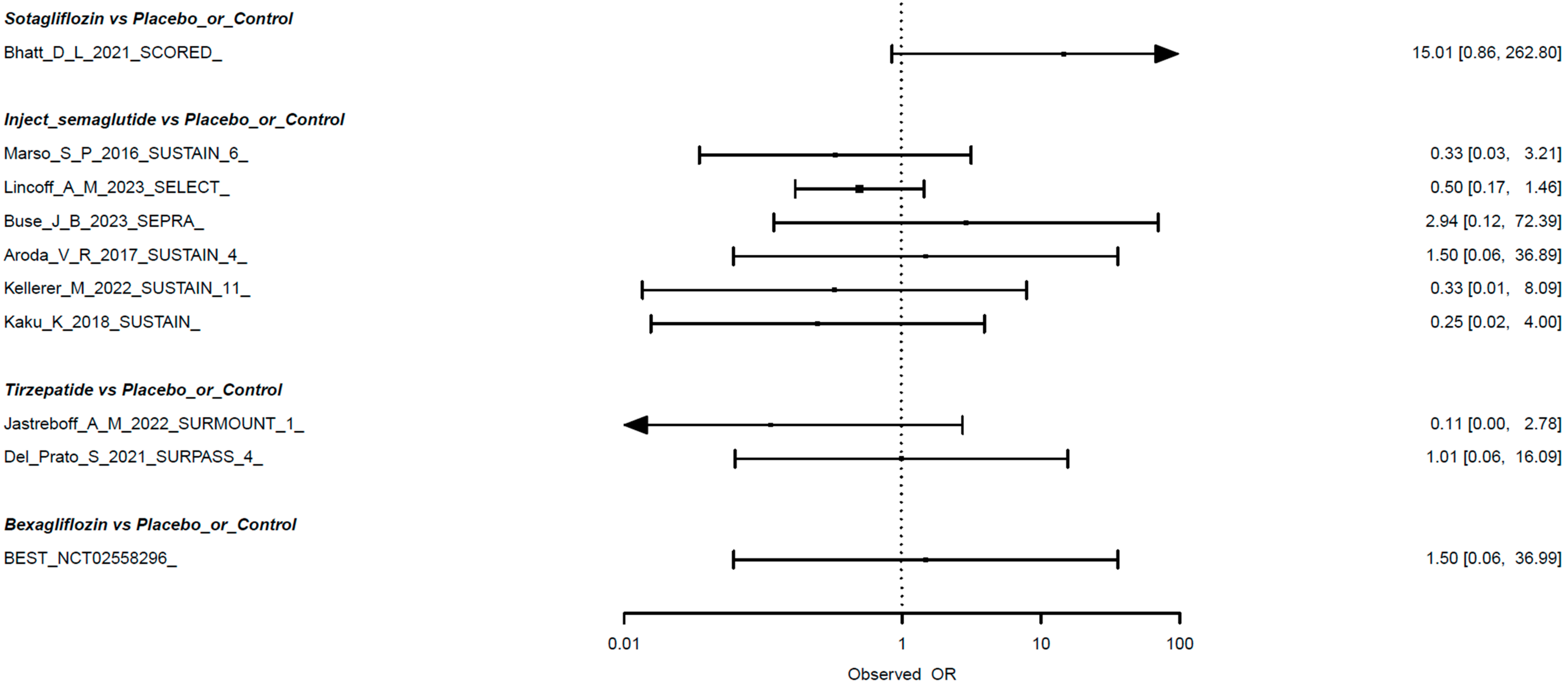
Sotagliflozin vs Placebo_or_Control
Bhatt_D_L_2021_SCORED_
15.01 [0.86, 262.80]
Inject_semaglutide vs Placebo_or_Control
Marso_S_P_2016_SUSTAIN_6_
0.33 [0.03, 3.21]
Lincoff_A_M_2023_SELECT_
0.50 [0.17, 1.46]
Buse_J_B_2023_SEPRA_
2.94 [0.12, 72.39]
Aroda_V_R_2017_SUSTAIN_4_
1.50 [0.06, 36.89]
Kellerer_M_2022_SUSTAIN_11_
0.33 [0.01, 8.09]
Kaku_K_2018_SUSTAIN_
0.25 [0.02, 4.00]
Tirzepatide vs Placebo_or_Control
Jastreboff_A_M_2022_SURMOUNT_1_
0.11 [0.00, 2.78]
Del_Prato_S_2021_SURPASS_4_
1.01 [0.06, 16.09]
Bexagliflozin vs Placebo_or_Control
BEST_NCT02558296_
1.50 [0.06, 36.99]
0.01
1
10
100
Observed OR

Figure S3D Individual study result of primary outcome: subgroup analysis of respiratory and intrathoracic organ origin

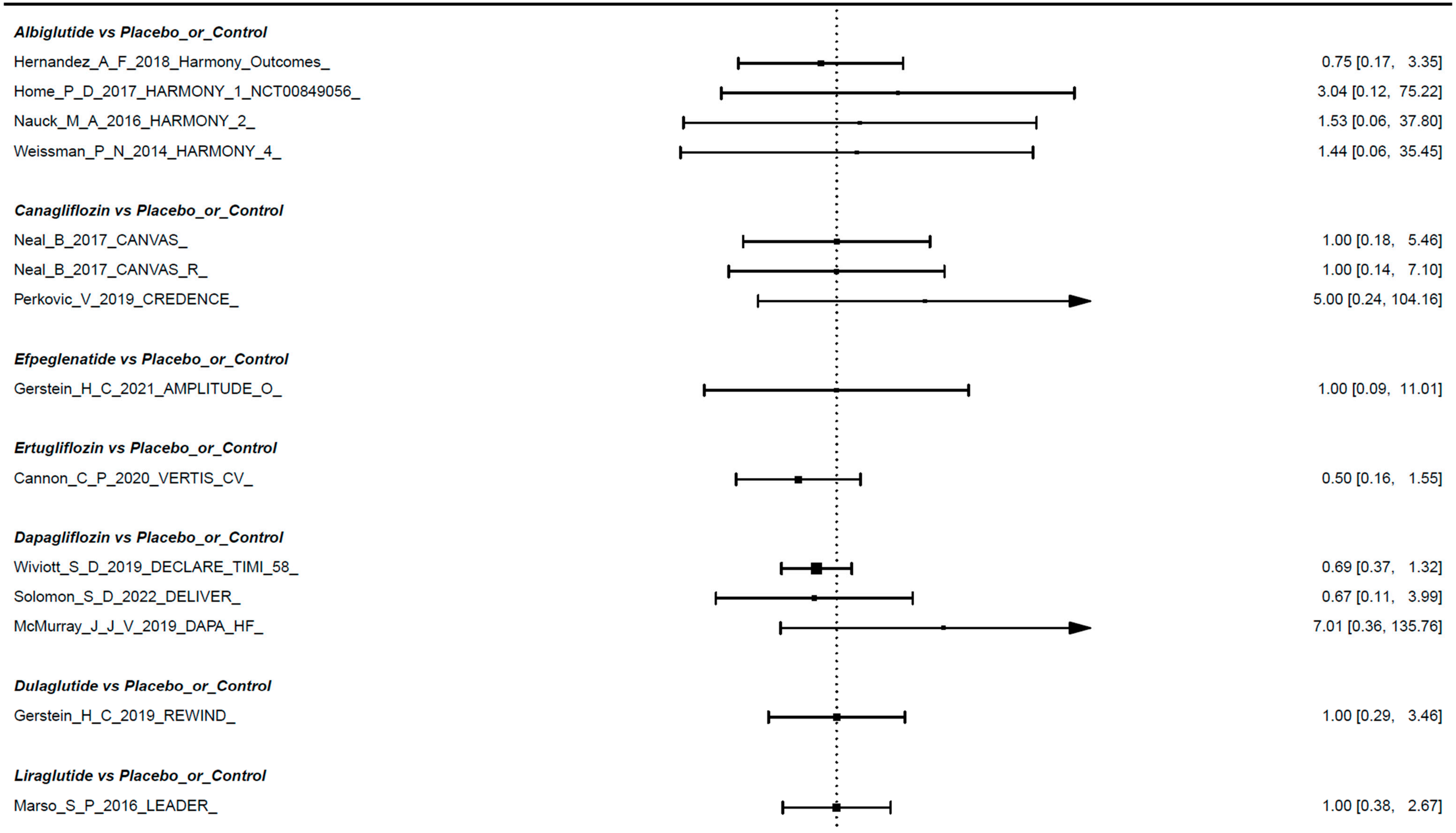

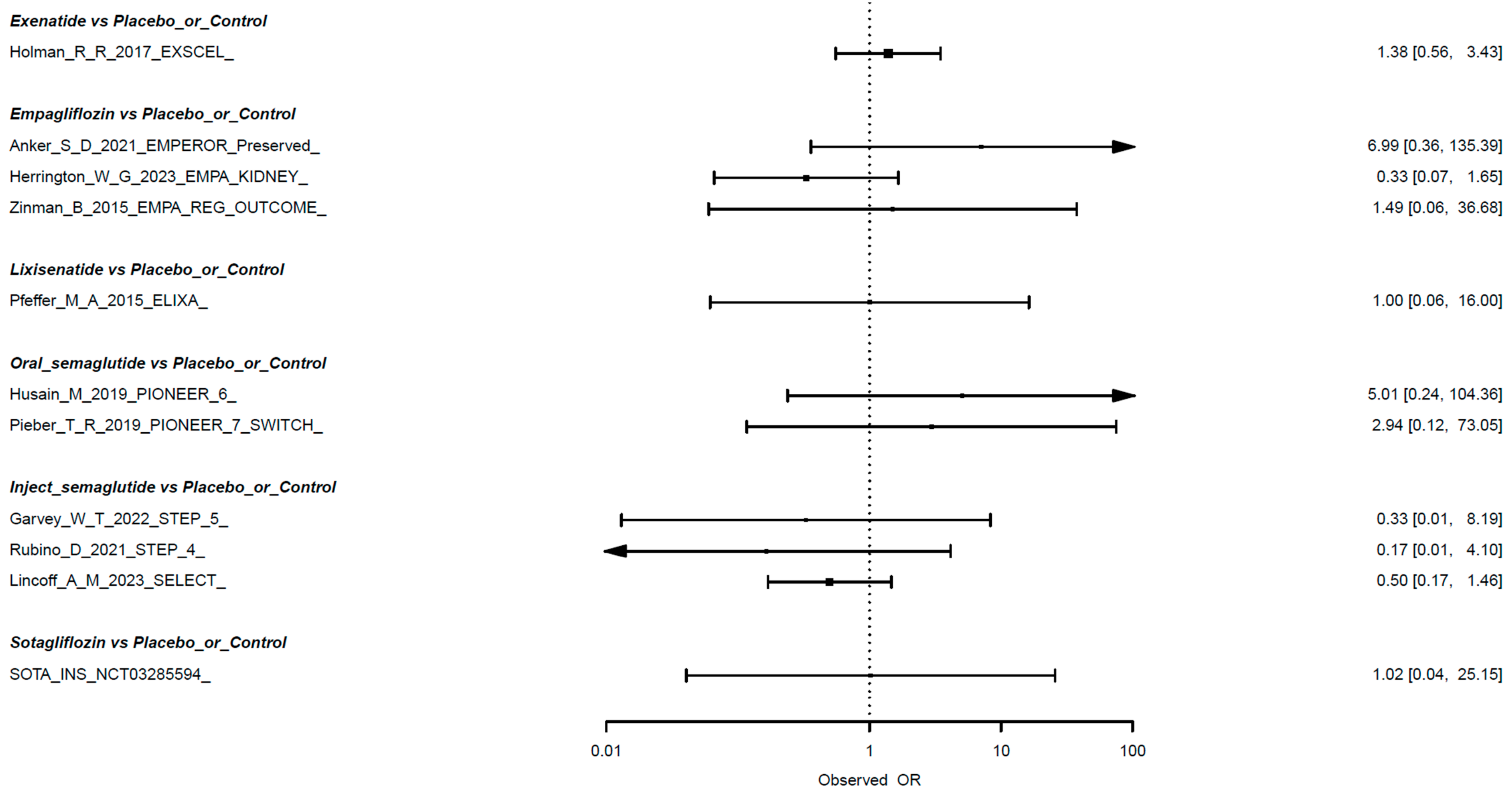

Exenatide vs Placebo_or_Control
Holman_R_R_2017_EXSCEL_
1.38 [0.56, 3.43]
Empagliflozin vs Placebo_or_Control
Anker_S_D_2021_EMPEROR_Preserved_
6.99 [0.36, 135.39]
Herrington_W_G_2023_EMPA_KIDNEY_
0.33 [0.07, 1.65]
Zinman_B_2015_EMPA_REG_OUTCOME_
1.49 [0.06, 36.68]
Lixisenatide vs Placebo_or_Control
Pfeffer_M_A_2015_ELIXA_
1.00 [0.06, 16.00]
Oral_semaglutide vs Placebo_or_Control
Husain_M_2019_PIONEER_6_
5.01 [0.24, 104.36]
Pieber_T_R_2019_PIONEER_7_SWITCH_
2.94 [0.12, 73.05]
Inject_semaglutide vs Placebo_or_Control
Garvey_W_T_2022_STEP_5_
0.33 [0.01, 8.19]
Rubino_D_2021_STEP_4_
0.17 [0.01, 4.10]
Lincoff_A_M_2023_SELECT_
0.50 [0.17, 1.46]
Sotagliflozin vs Placebo_or_Control
SOTA_INS_NCT03285594_
1.02 [0.04, 25.15]
0.01
1
10
100
Observed OR

Figure S3E Individual study result of primary outcome: subgroup analysis of bone origin

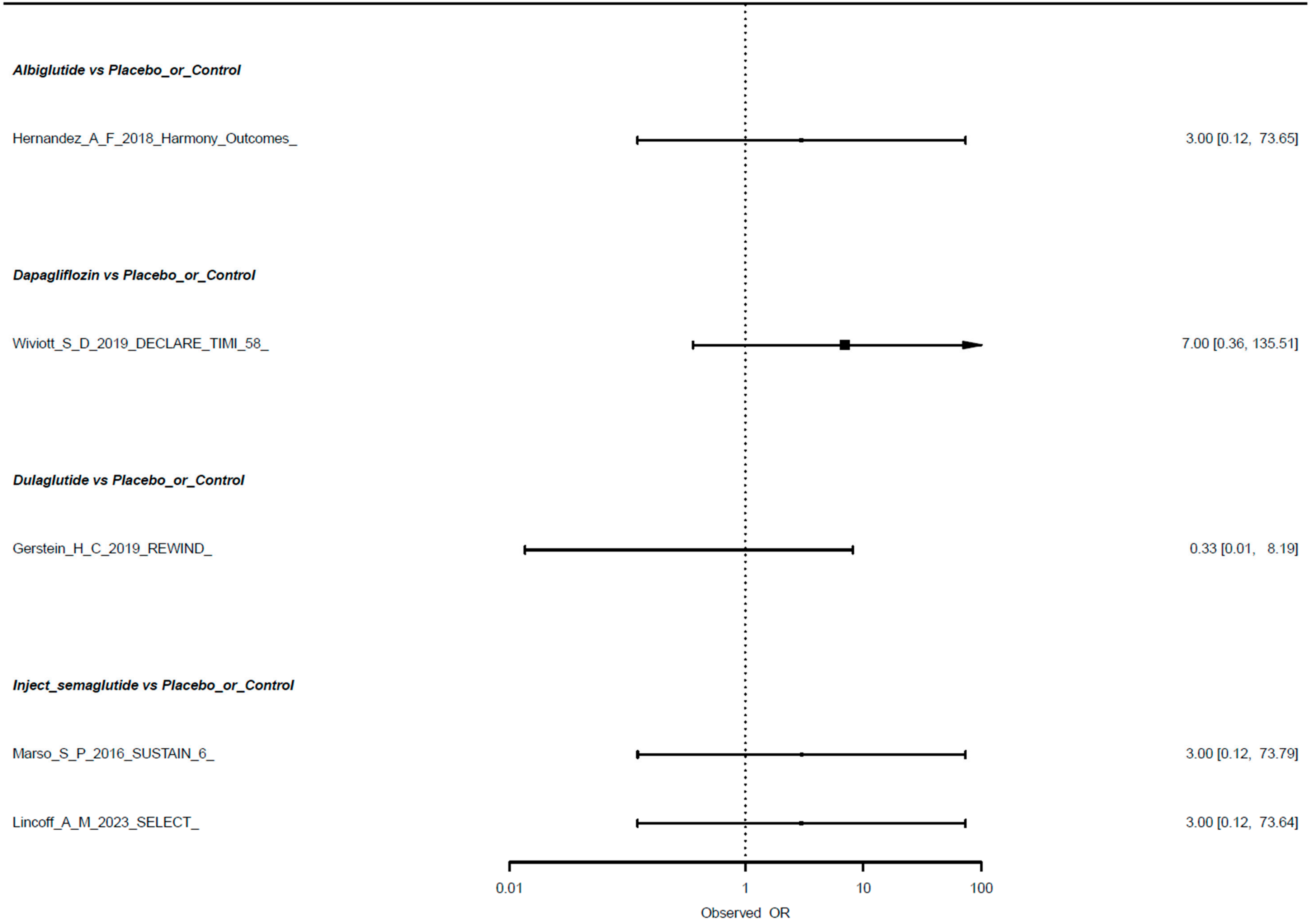

Figure S3F Individual study result of primary outcome: subgroup analysis of skin, mesothelium, soft tissue, and cartilage origin

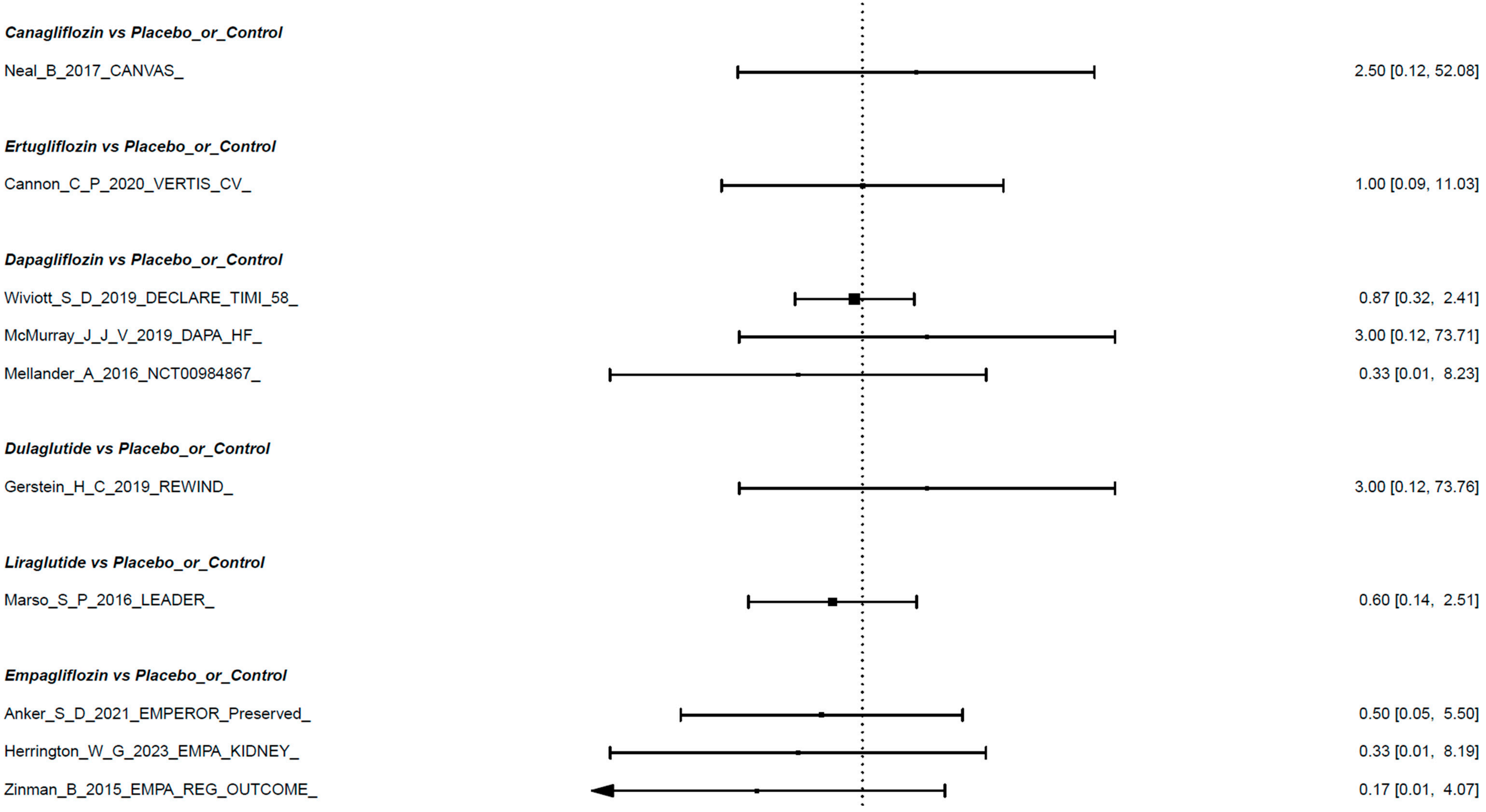

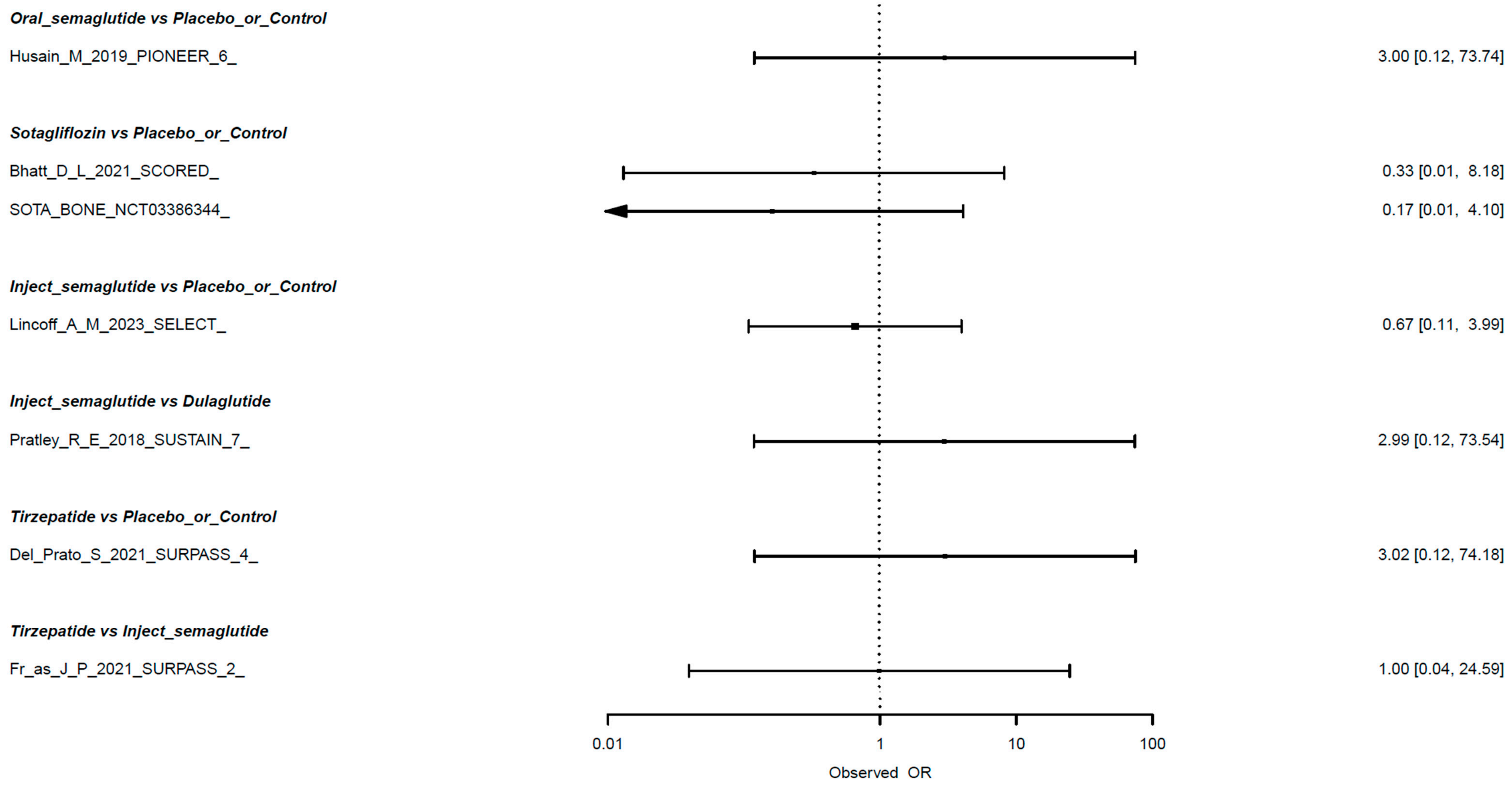
Oral_semaglutide vs Placebo_or_Control
Husain_M_2019_PIONEER_6_
3.00 [0.12, 73.74]
Sotagliflozin vs Placebo_or_Control
Bhatt_D_L_2021_SCORED_
0.33 [0.01, 8.18]
SOTA_BONE_NCT03386344_
0.17 [0.01, 4.10]
Inject_semaglutide vs Placebo_or_Control
Lincoff_A_M_2023_SELECT_
0.67 [0.11, 3.99]
Inject_semaglutide vs Dulaglutide
Pratley_R_E_2018_SUSTAIN_7_
2.99 [0.12, 73.54]
Tirzepatide vs Placebo_or_Control
Del_Prato_S_2021_SURPASS_4_
3.02 [0.12, 74.18]
Tirzepatide vs Inject_semaglutide
Fr_as_J_P_2021_SURPASS_2_
1.00 [0.04, 24.59]
0.01
1
10
100
Observed OR

Figure S3G Individual study result of primary outcome: subgroup analysis of breast and female genital organ origin

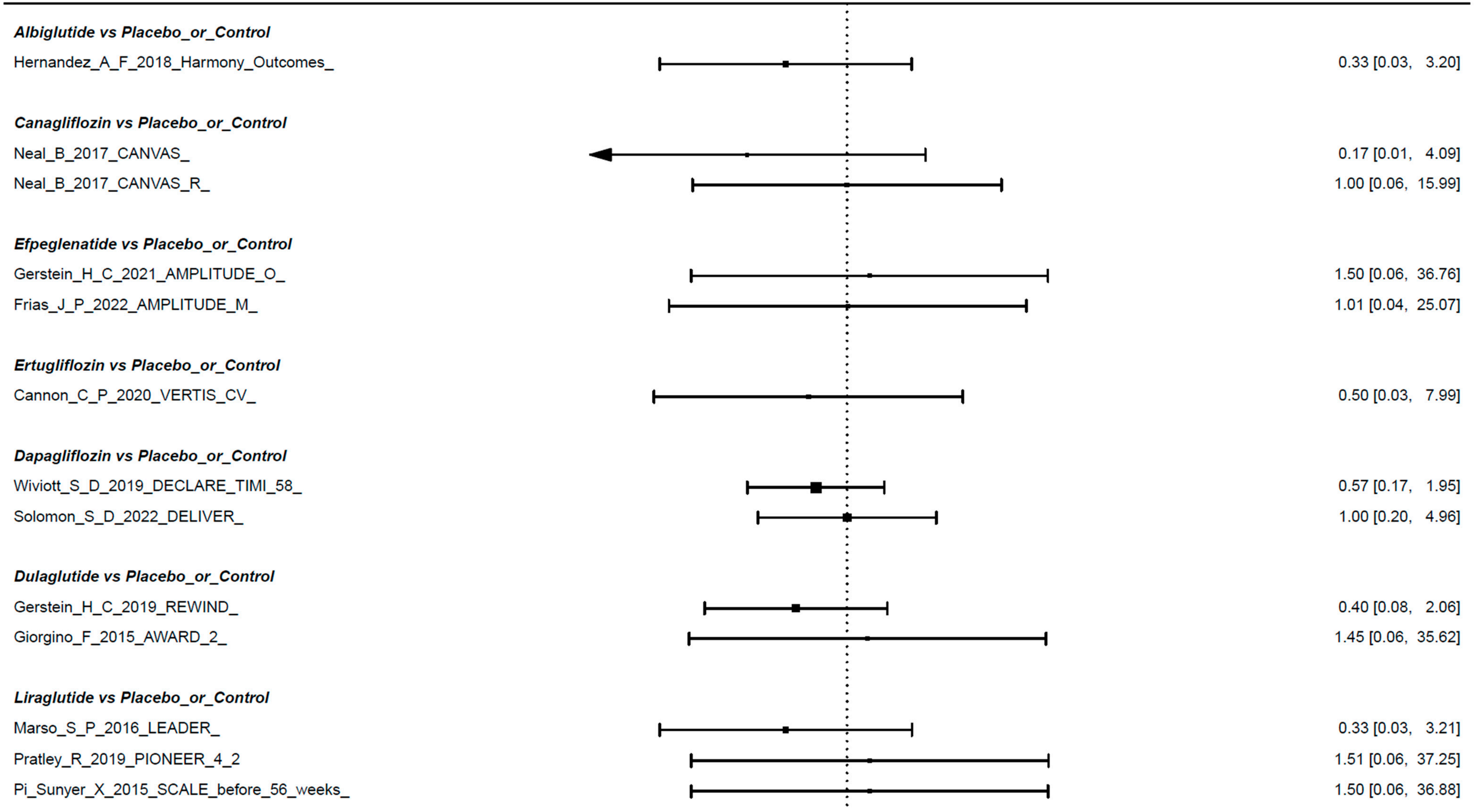

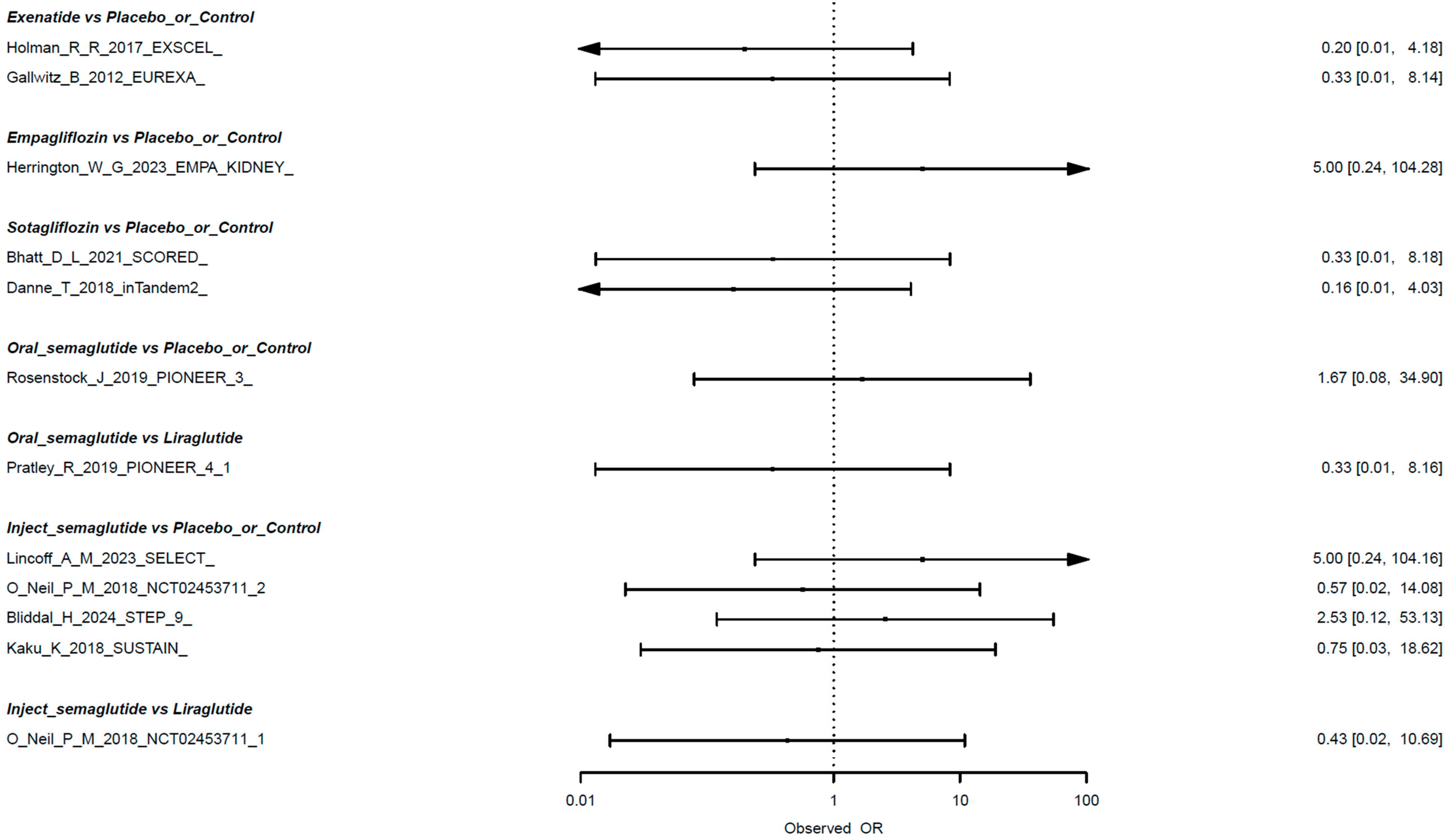
Exenatide vs Placebo_or_Control
Holman_R_R_2017_EXSCEL_ 0.20 [0.01, 4.18]
Gallwitz_B_2012_EUREXA_ 0.33 [0.01, 8.14]
Empagliflozin vs Placebo_or_Control
Herrington_W_G_2023_EMPA_KIDNEY_ 5.00 [0.24, 104.28]
Sotagliflozin vs Placebo_or_Control
Bhatt_D_L_2021_SCORED_ 0.33 [0.01, 8.18]
Danne_T_2018_inTandem2_ 0.16 [0.01, 4.03]
Oral_semaglutide vs Placebo_or_Control
Rosenstock_J_2019_PIONEER_3_ 1.67 [0.08, 34.90]
Oral_semaglutide vs Liraglutide
Pratley_R_2019_PIONEER_4_1 0.33 [0.01, 8.16]
Inject_semaglutide vs Placebo_or_Control
Lincoff_A_M_2023_SELECT_ 5.00 [0.24, 104.16]
O_Neil_P_M_2018_NCT02453711_2 0.57 [0.02, 14.08]
Bliddal_H_2024_STEP_9_ 2.53 [0.12, 53.13]
Kaku_K_2018_SUSTAIN_ 0.75 [0.03, 18.62]
Inject_semaglutide vs Liraglutide
O_Neil_P_M_2018_NCT02453711_1 0.43 [0.02, 10.69]
0.01
1
10
100
Observed OR

Figure S3H Individual study result of primary outcome: subgroup analysis of prostate and male genital organ origin

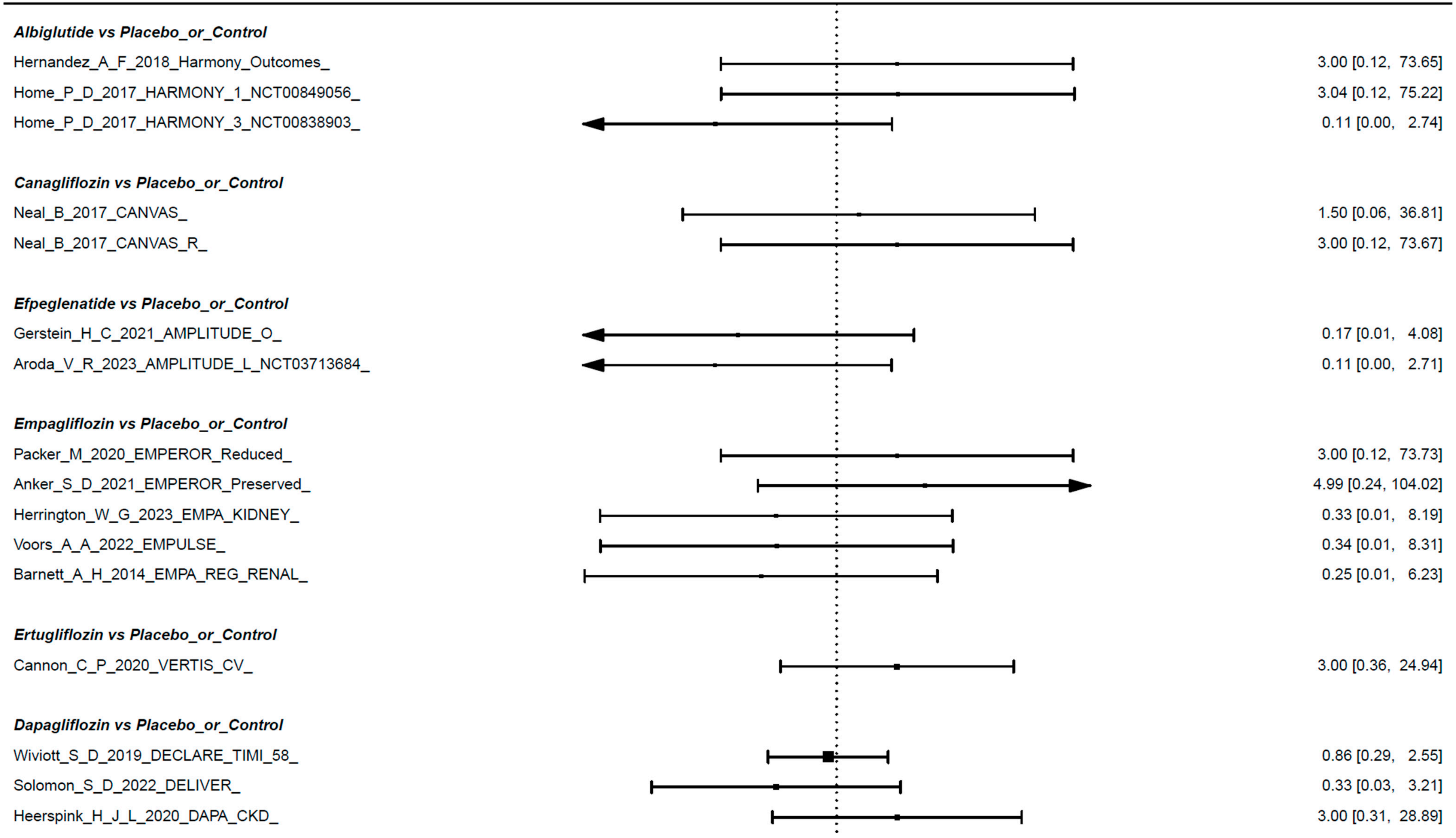

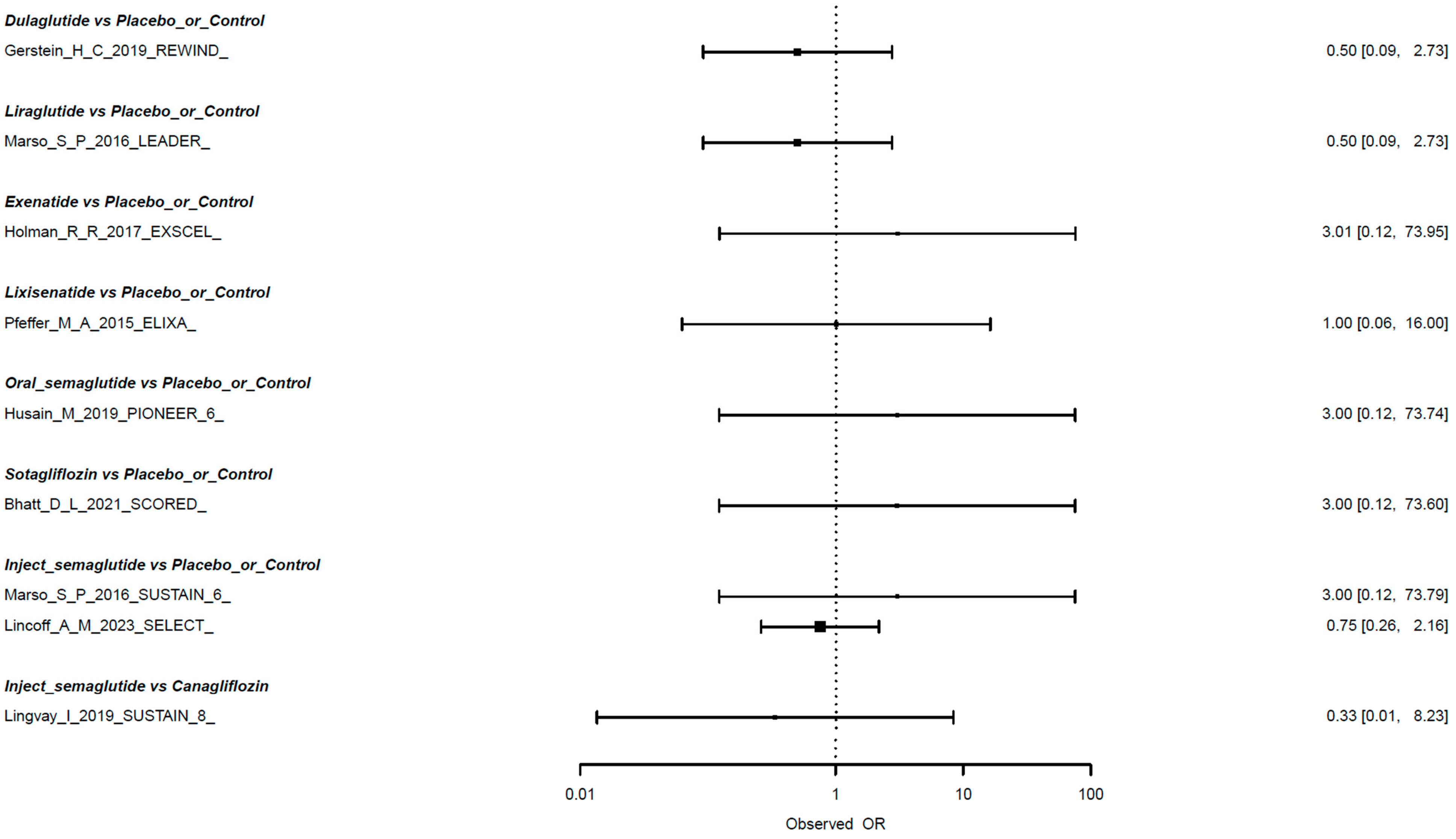
Dulaglutide vs Placebo_or_Control
Gerstein_H_C_2019_REWIND_
0.50 [0.09, 2.73]
Liraglutide vs Placebo_or_Control
Marso_S_P_2016_LEADER_
0.50 [0.09, 2.73]
Exenatide vs Placebo_or_Control
Holman_R_R_2017_EXSCEL_
3.01 [0.12, 73.95]
Lixisenatide vs Placebo_or_Control
Pfeffer_M_A_2015_ELIXA_
1.00 [0.06, 16.00]
Oral_semaglutide vs Placebo_or_Control
Husain_M_2019_PIONEER_6_
3.00 [0.12, 73.74]
Sotagliflozin vs Placebo_or_Control
Bhatt_D_L_2021_SCORED_
3.00 [0.12, 73.60]
Inject_semaglutide vs Placebo_or_Control
Marso_S_P_2016_SUSTAIN_6_
3.00 [0.12, 73.79]
Lincoff_A_M_2023_SELECT_
0.75 [0.26, 2.16]
Inject_semaglutide vs Canagliflozin
Lingvay_I_2019_SUSTAIN_8_
0.33 [0.01, 8.23]
0.01
1
10
100
Observed OR

Figure S3I Individual study result of primary outcome: subgroup analysis of kidney and urinary tract origin

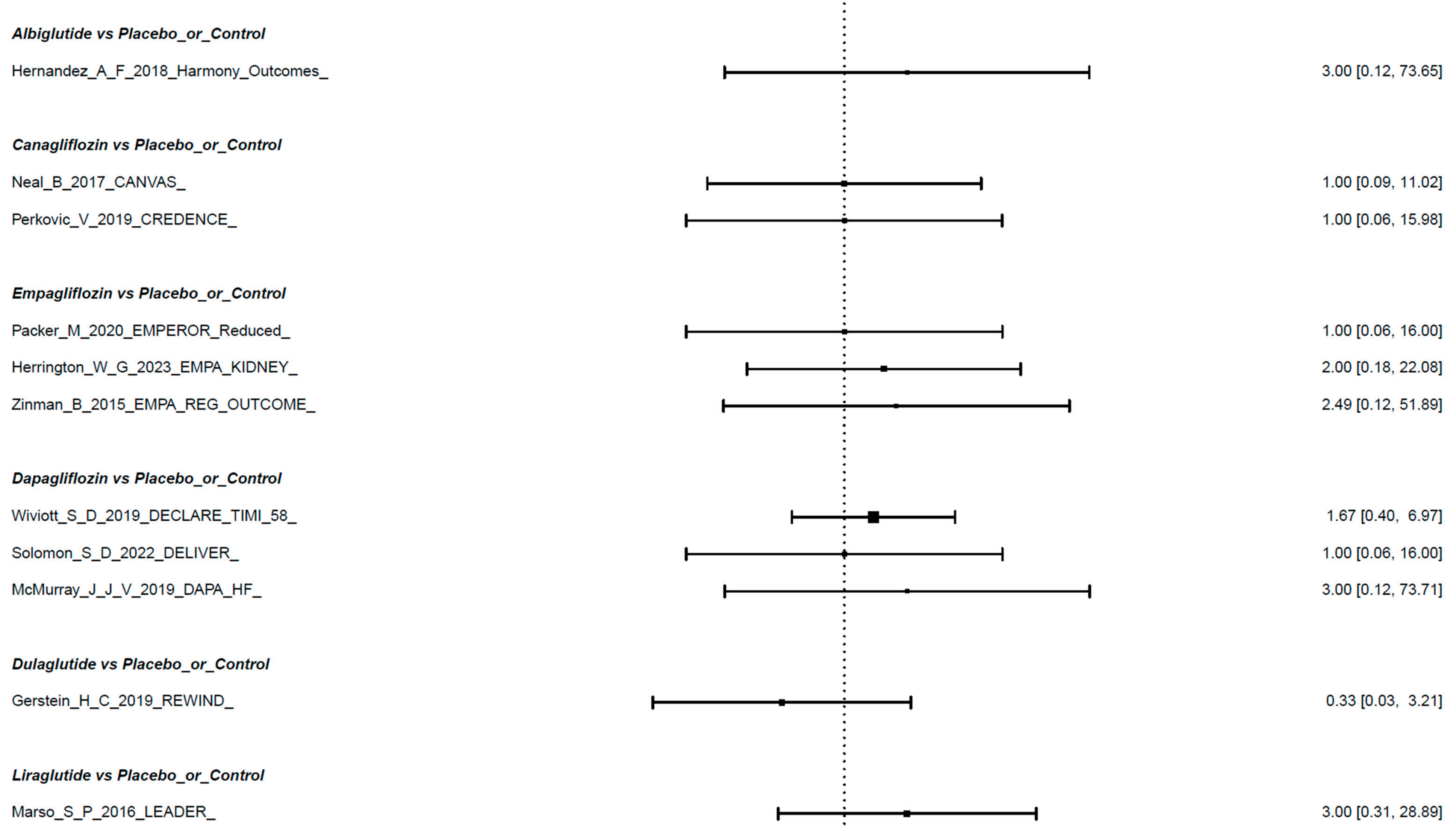

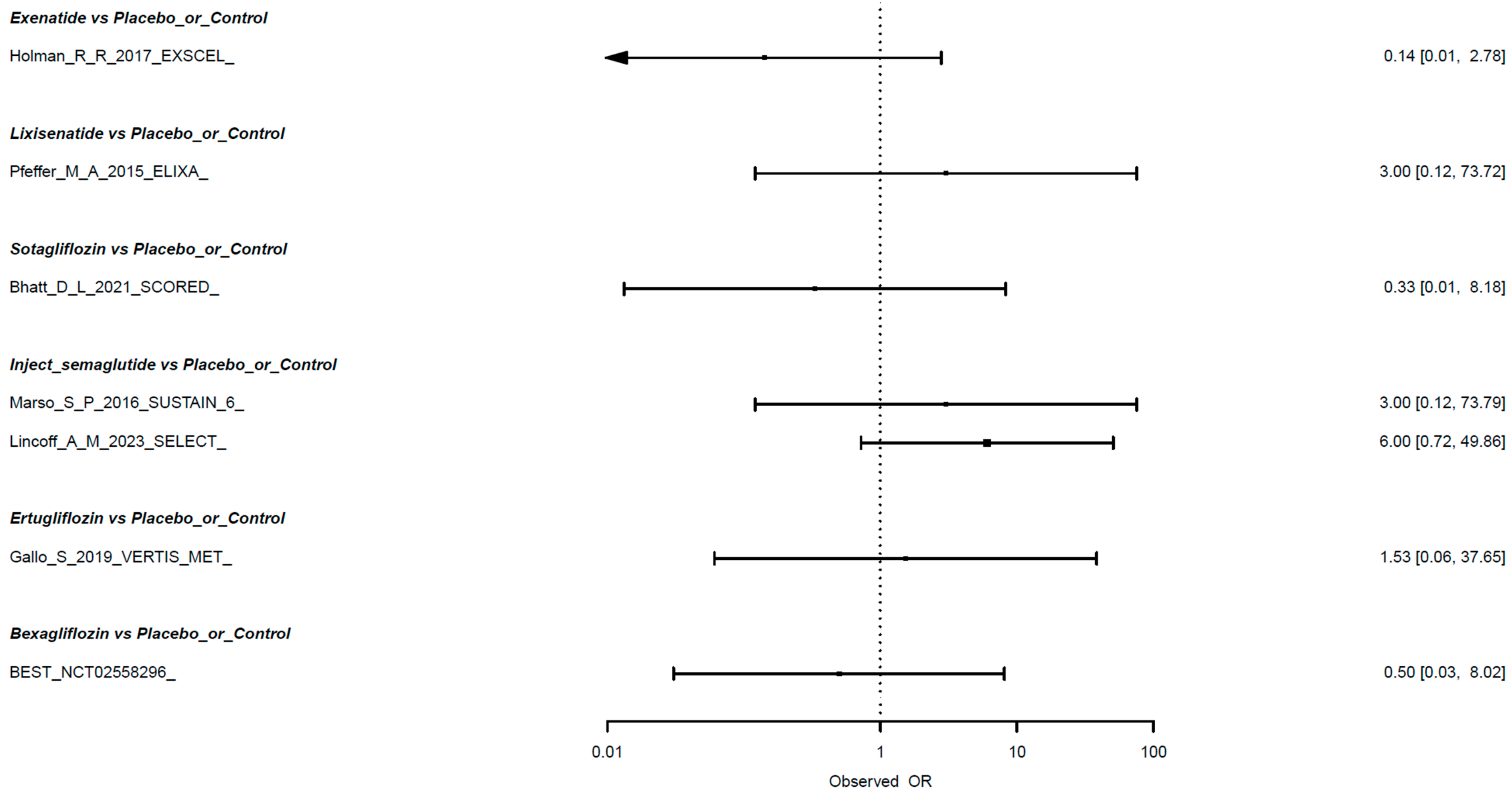
Exenatide vs Placebo_or_Control
Holman_R_R_2017_EXSCEL_
0.14 [0.01, 2.78]
Lixisenatide vs Placebo_or_Control
Pfeffer_M_A_2015_ELIXA_
3.00 [0.12, 73.72]
Sotagliflozin vs Placebo_or_Control
Bhatt_D_L_2021_SCORED_
0.33 [0.01, 8.18]
Inject_semaglutide vs Placebo_or_Control
Marso_S_P_2016_SUSTAIN_6_
3.00 [0.12, 73.79]
Lincoff_A_M_2023_SELECT_
6.00 [0.72, 49.86]
Ertugliflozin vs Placebo_or_Control
Gallo_S_2019_VERTIS_MET_
1.53 [0.06, 37.65]
Bexagliflozin vs Placebo_or_Control
BEST_NCT02558296_
0.50 [0.03, 8.02]
0.01
1
10
100
Observed OR

Figure S3J Individual study result of primary outcome: subgroup analysis of neuron, nerve, and neuroendocrine origin

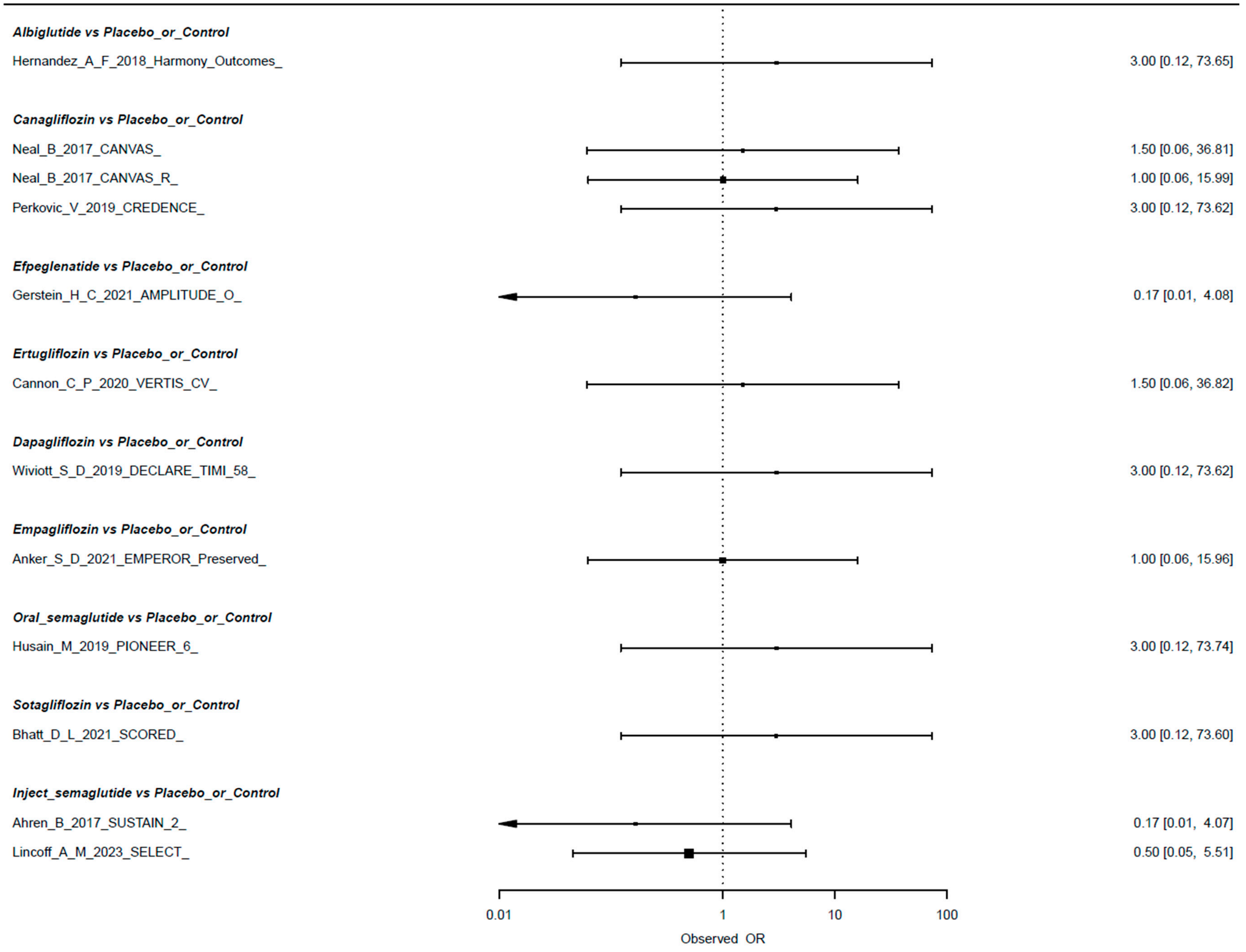

Figure S3K Individual study result of primary outcome: subgroup analysis of thyroid and other endocrine gland origin

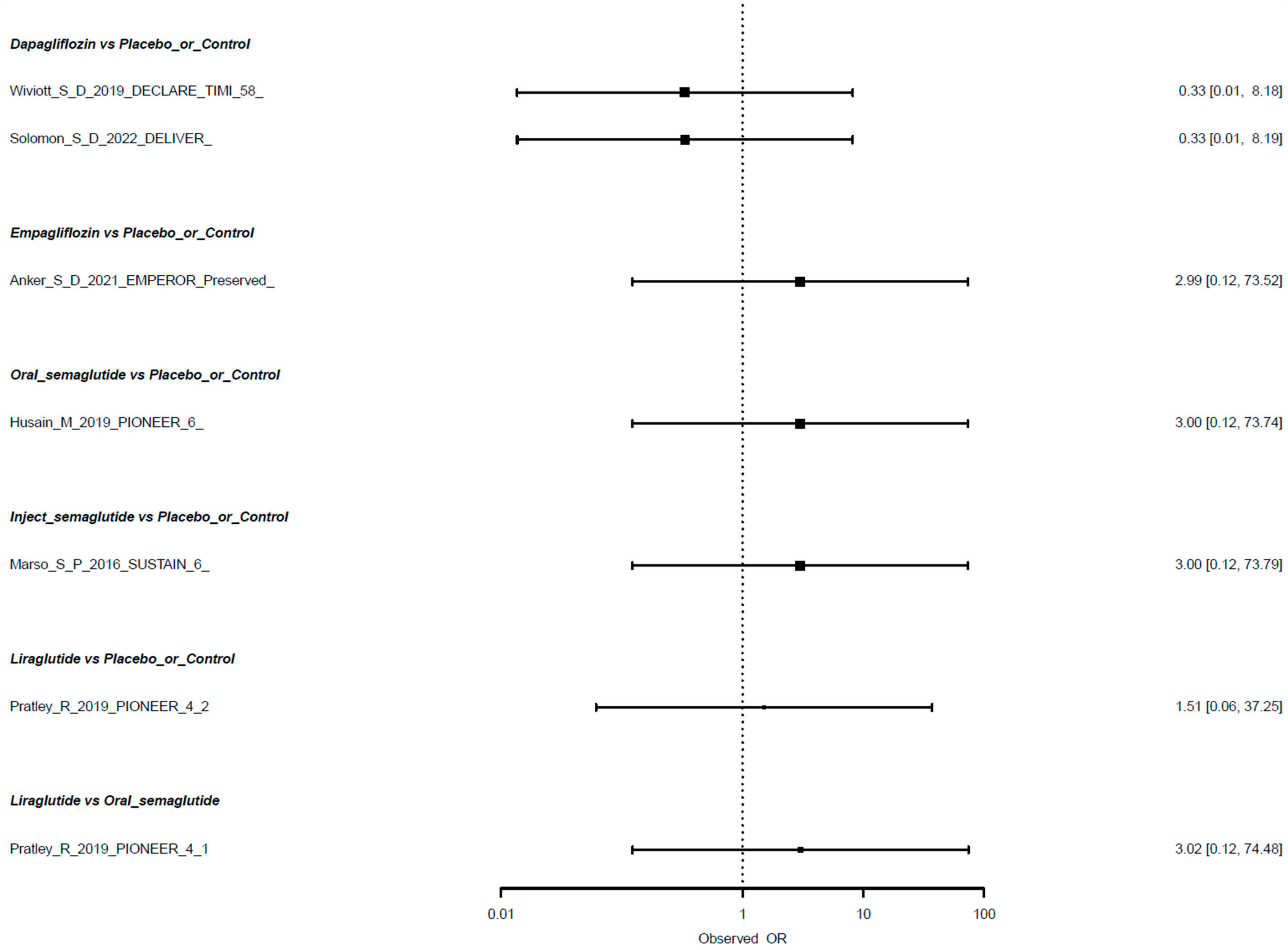

Figure S3L Individual study result of safety profile: drop-out rate

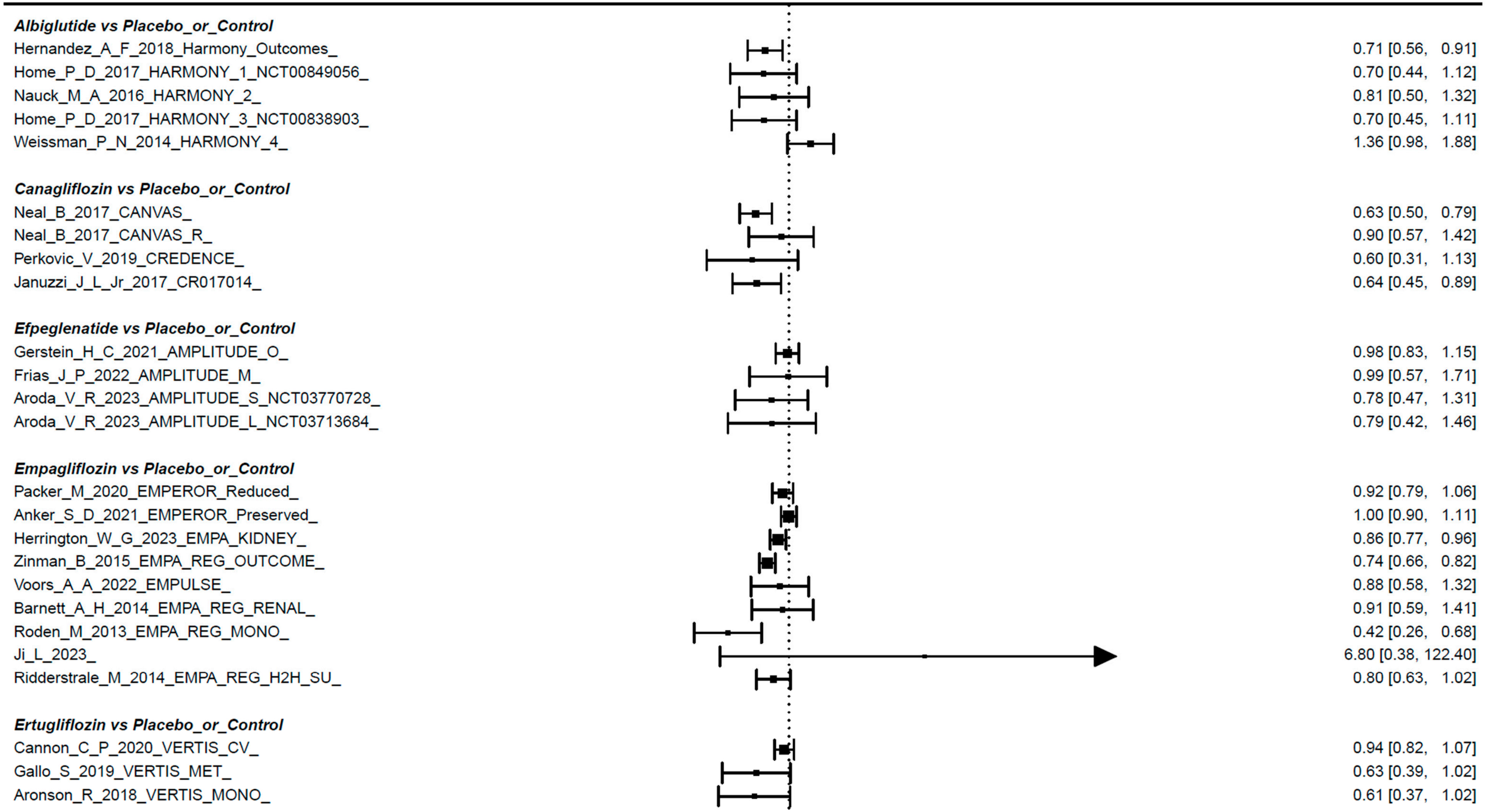

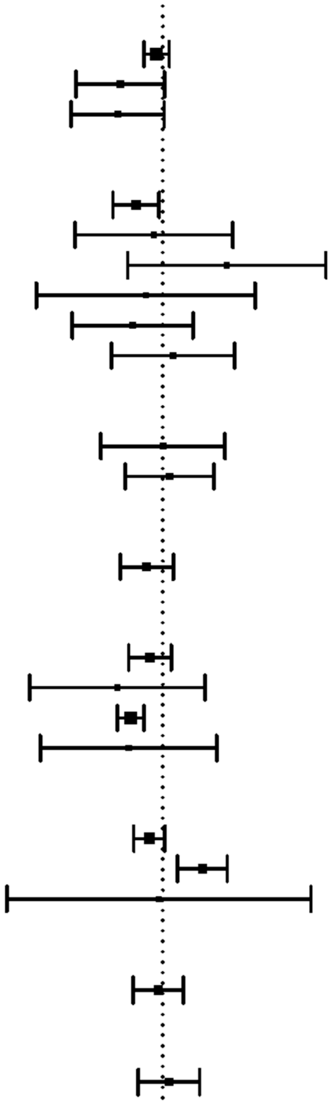

Comparison / Study	Estimate [95% CI]
Ertugliflozin vs Placebo_or_Control	
Cannon_C_P_2020_VERTIS_CV_	0.94 [0.82, 1.07]
Gallo_S_2019_VERTIS_MET_	0.63 [0.39, 1.02]
Aronson_R_2018_VERTIS_MONO_	0.61 [0.37, 1.02]
Dapagliflozin vs Placebo_or_Control	
Wiviott_S_D_2019_DECLARE_TIMI_58_	0.75 [0.58, 0.96]
Solomon_S_D_2022_DELIVER_	0.91 [0.39, 2.14]
Heerspink_H_J_L_2020_DAPA_CKD_	2.00 [0.68, 5.87]
McMurray_J_J_V_2019_DAPA_HF_	0.83 [0.25, 2.73]
Mellander_A_2016_NCT00984867_	0.72 [0.37, 1.39]
Mellander_A_2016_NCT00528372_	1.12 [0.57, 2.18]
Dulaglutide vs Placebo_or_Control	
Gerstein_H_C_2019_REWIND_	1.00 [0.51, 1.96]
Giorgino_F_2015_AWARD_2_	1.08 [0.67, 1.74]
Dulaglutide vs Efpeglenatide	
Aroda_V_R_2023_AMPLITUDE_D_NCT03684642_	0.84 [0.63, 1.12]
Liraglutide vs Placebo_or_Control	
Marso_S_P_2016_LEADER_	0.87 [0.69, 1.10]
Pratley_R_2019_PIONEER_4_	0.61 [0.24, 1.58]
Pi_Sunyer_X_2015_SCALE_before_56_weeks_	0.71 [0.61, 0.82]
O_Neil_P_M_2018_NCT02453711_	0.69 [0.26, 1.80]
Exenatide vs Placebo_or_Control	
Holman_R_R_2017_EXSCEL_	0.86 [0.73, 1.02]
Gallwitz_B_2012_EUREXA_	1.54 [1.17, 2.02]
FLAT_SUGAR_Trial_Investigators_2016_FLAT_SUGAR_	0.96 [0.18, 4.99]
Lixisenatide vs Placebo_or_Control	
Pfeffer_M_A_2015_ELIXA_	0.95 [0.73, 1.25]
Lixisenatide vs Exenatide	
Rosenstock_J_2013_GetGoal_X_	1.07 [0.77, 1.49]

Study		Estimate [95% CI]
Oral_semaglutide vs Placebo_or_Control		
Husain_M_2019_PIONEER_6_		0.83 [0.25, 2.74]
Pieber_T_R_2019_PIONEER_7_SWITCH_		1.74 [0.67, 4.48]
Rosenstock_J_2019_PIONEER_3_		1.94 [1.13, 3.34]
Pratley_R_2019_PIONEER_4_		0.48 [0.18, 1.32]
Oral_semaglutide vs Liraglutide		
Pratley_R_2019_PIONEER_4_		0.79 [0.31, 2.04]
Sotagliflozin vs Placebo_or_Control		
Bhatt_D_L_2021_SCORED_		0.89 [0.79, 1.01]
Danne_T_2018_inTandem2_		1.07 [0.69, 1.66]
SOTA_INS_NCT03285594_		1.09 [0.59, 2.02]
SOTA_BONE_NCT03386344_		0.72 [0.37, 1.42]
Inject_semaglutide vs Placebo_or_Control		
Marso_S_P_2016_SUSTAIN_6_		0.62 [0.37, 1.03]
Ahren_B_2017_SUSTAIN_2_		1.13 [0.65, 1.97]
Garvey_W_T_2022_STEP_5_		0.20 [0.07, 0.61]
Rubino_D_2021_STEP_4_		0.47 [0.28, 0.79]
Lincoff_A_M_2023_SELECT_		0.91 [0.77, 1.08]
O_Neil_P_M_2018_NCT02453711_		0.63 [0.33, 1.21]
Bliddal_H_2024_STEP_9_		0.89 [0.44, 1.76]
Buse_J_B_2023_SEPRA_		0.93 [0.73, 1.19]
Aroda_V_R_2017_SUSTAIN_4_		1.29 [0.74, 2.26]
Kellerer_M_2022_SUSTAIN_11_		0.55 [0.33, 0.91]
Kaku_K_2018_SUSTAIN_		0.66 [0.25, 1.73]
Inject_semaglutide vs Canagliflozin		
Lingvay_I_2019_SUSTAIN_8_		1.24 [0.70, 2.22]
Inject_semaglutide vs Dulaglutide		
Pratley_R_E_2018_SUSTAIN_7_		1.51 [0.93, 2.46]
Inject_semaglutide vs Liraglutide		
O_Neil_P_M_2018_NCT02453711_		0.92 [0.40, 2.09]

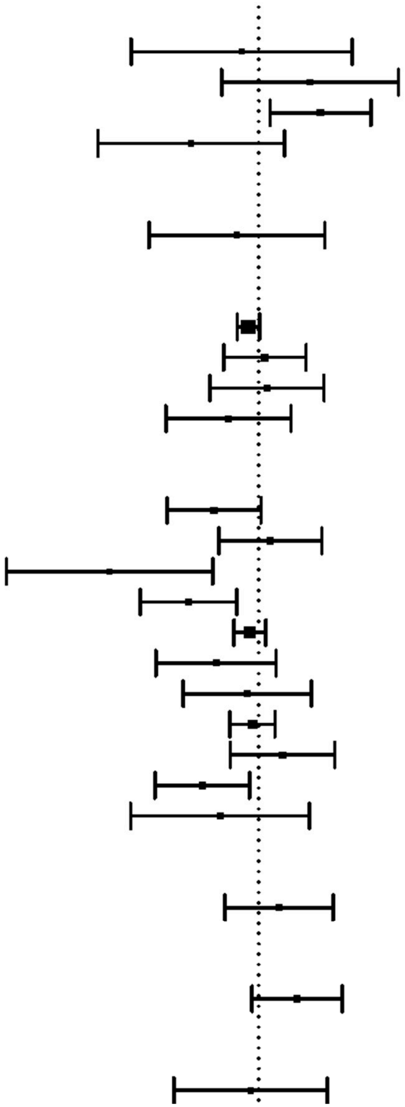

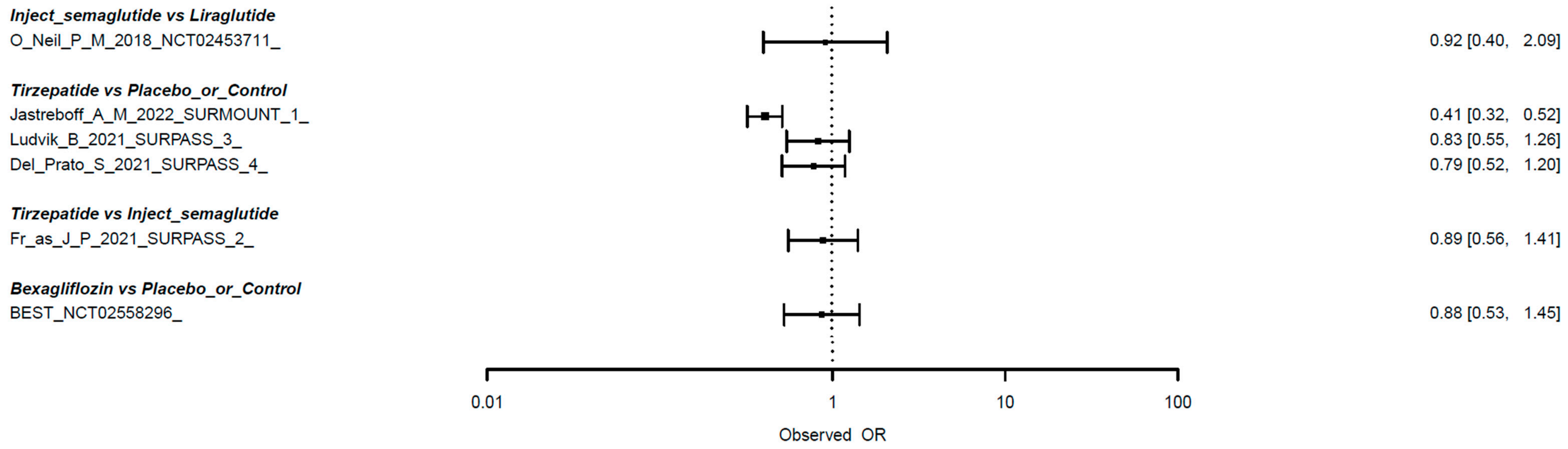
Inject_semaglutide vs Liraglutide
O_Neil_P_M_2018_NCT02453711_
0.92 [0.40, 2.09]
Tirzepatide vs Placebo_or_Control
Jastreboff_A_M_2022_SURMOUNT_1_
0.41 [0.32, 0.52]
Ludvik_B_2021_SURPASS_3_
0.83 [0.55, 1.26]
Del_Prato_S_2021_SURPASS_4_
0.79 [0.52, 1.20]
Tirzepatide vs Inject_semaglutide
Fr_as_J_P_2021_SURPASS_2_
0.89 [0.56, 1.41]
Bexagliflozin vs Placebo_or_Control
BEST_NCT02558296_
0.88 [0.53, 1.45]
0.01
1
10
100
Observed OR

Abbreviation for Figure S3A-3L:

95%CIs: 95% confidence intervals; GLP-1 agonist: glucagon-like peptide-1 agonist; NMA: network meta-analysis; OR: odds ratio; RCT: randomized controlled trial; SGLT2 inhibitor: sodium–glucose cotransporter 2 inhibitor

Figure S4 Bayesian-based forest plot of NMA of primary outcome: overall events of metastatic cancers

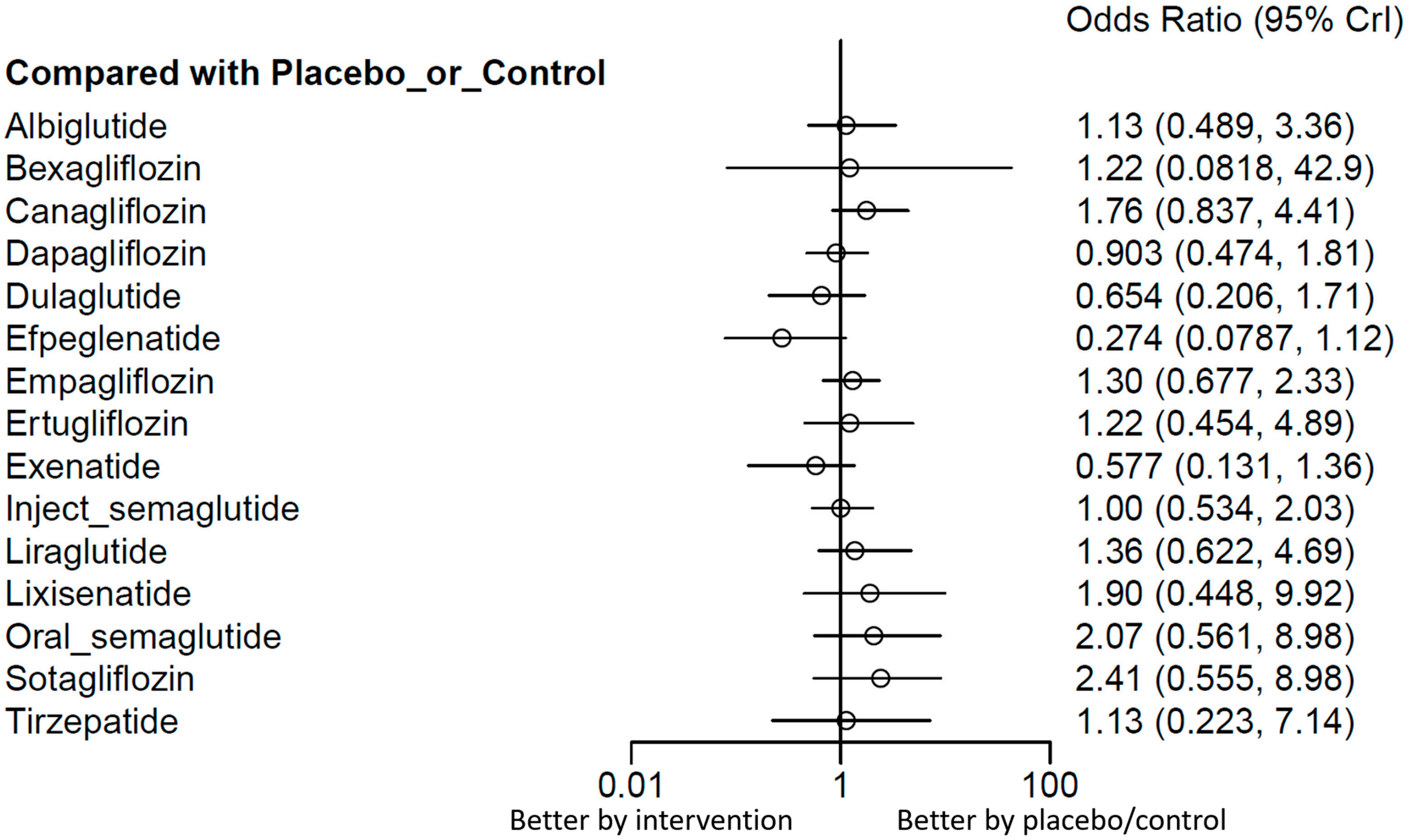

Abbreviation for Figure S4:

95%CIs: 95% confidence intervals; GLP-1 agonist: glucagon-like peptide-1 agonist; NMA: network meta-analysis; OR: odds ratio; RCT: randomized controlled trial; SGLT2 inhibitor: sodium–glucose cotransporter 2 inhibitor

Figure S5A Bayesian-based Litmus Rank-O-Gram rank plot of primary outcome: overall events of metastatic cancers

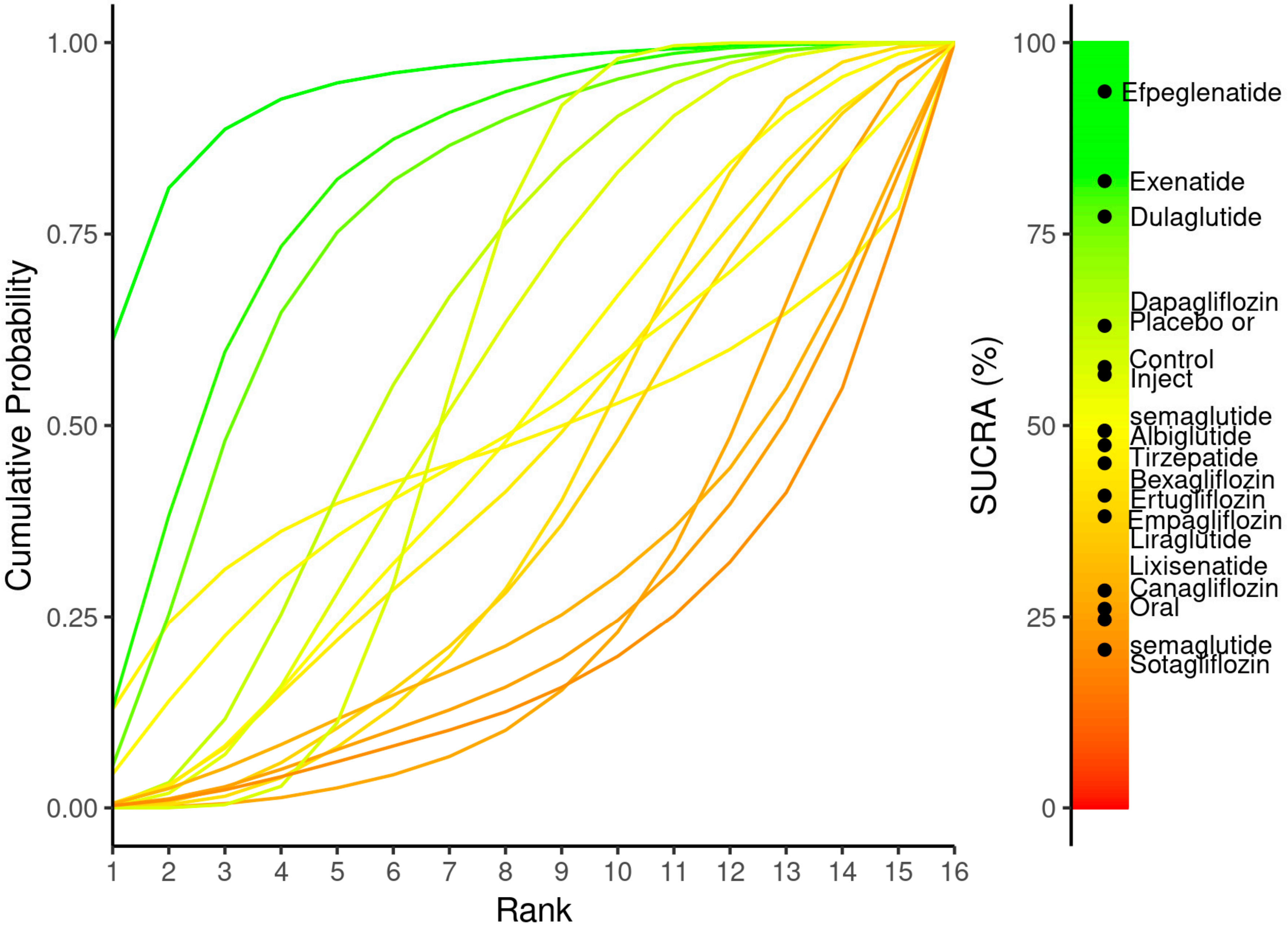

Figure S5B Bayesian-based radial surface under the cumulative ranking of primary outcome: overall events of metastatic cancers

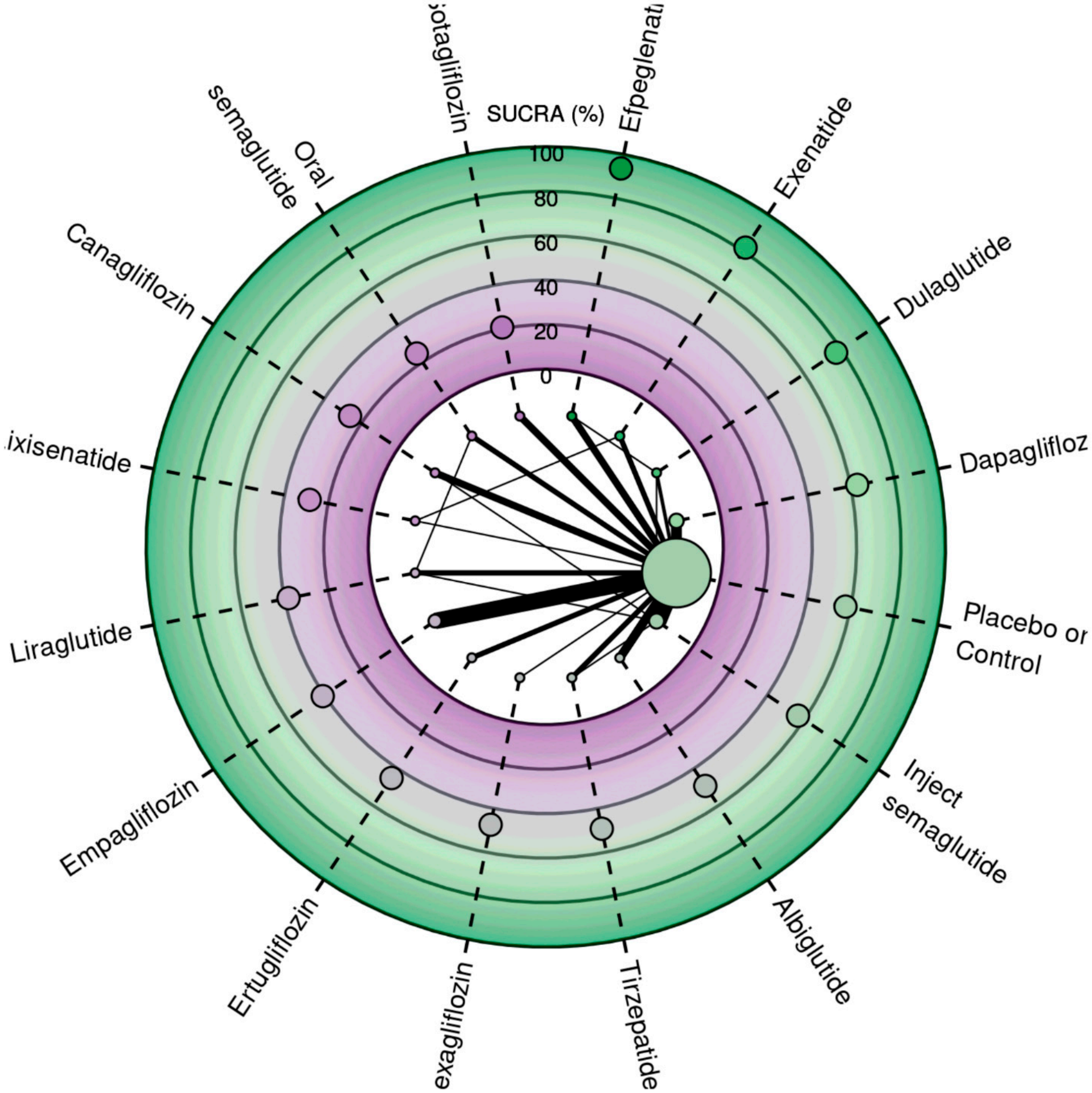

Abbreviation for Figure S5A-5B:

95%CIs: 95% confidence intervals; GLP-1 agonist: glucagon-like peptide-1 agonist; NMA: network meta-analysis; OR: odds ratio; RCT: randomized controlled trial; SGLT2 inhibitor: sodium–glucose cotransporter 2 inhibitor

Figure S6A Bayesian-based residual deviance NMA/UME model of primary outcome: overall events of metastatic cancers

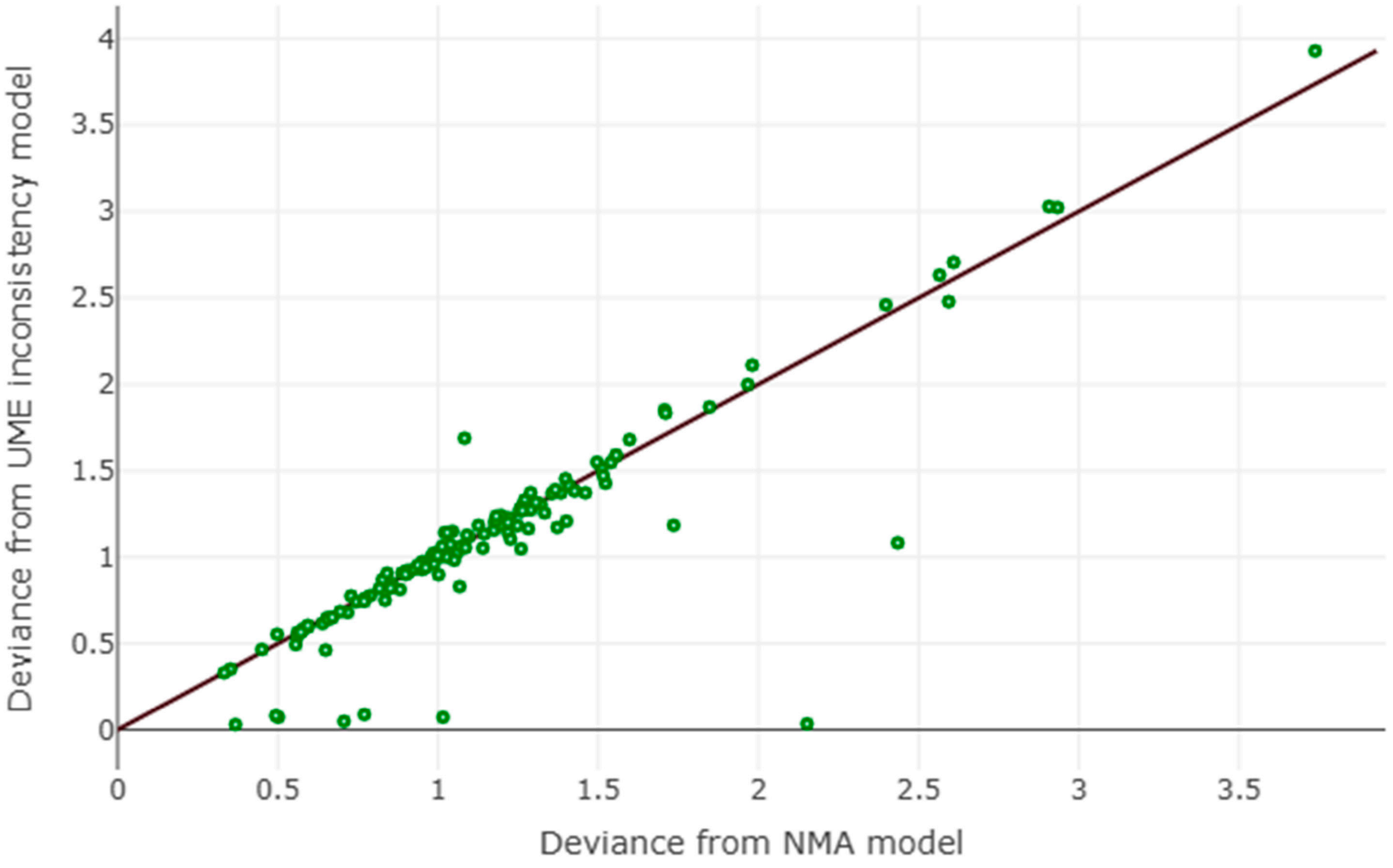

Figure S6B Bayesian-based per-arm residual deviance of primary outcome: overall events of metastatic cancers

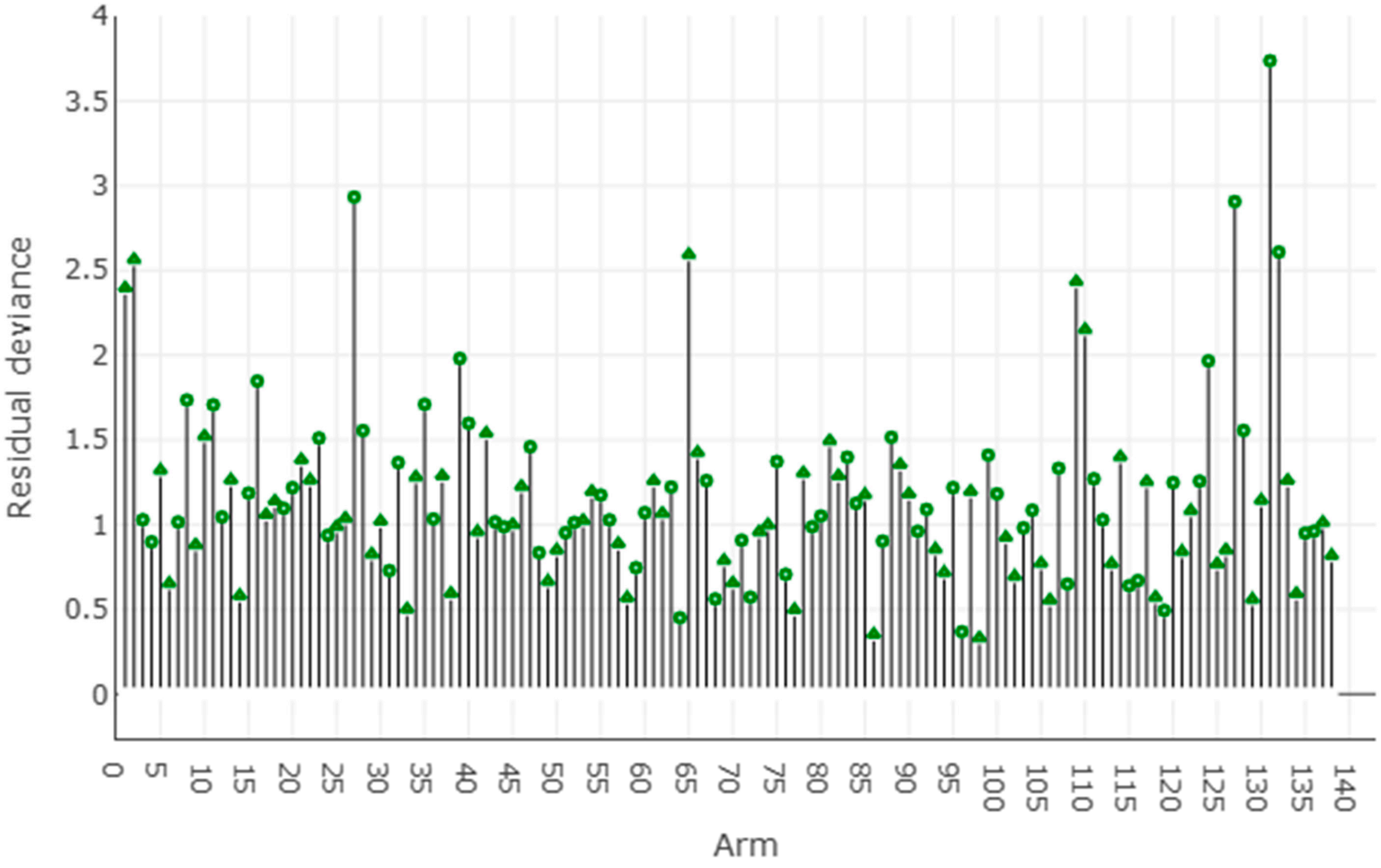

Figure S6C Bayesian-based leverage plot of primary outcome: overall events of metastatic cancers

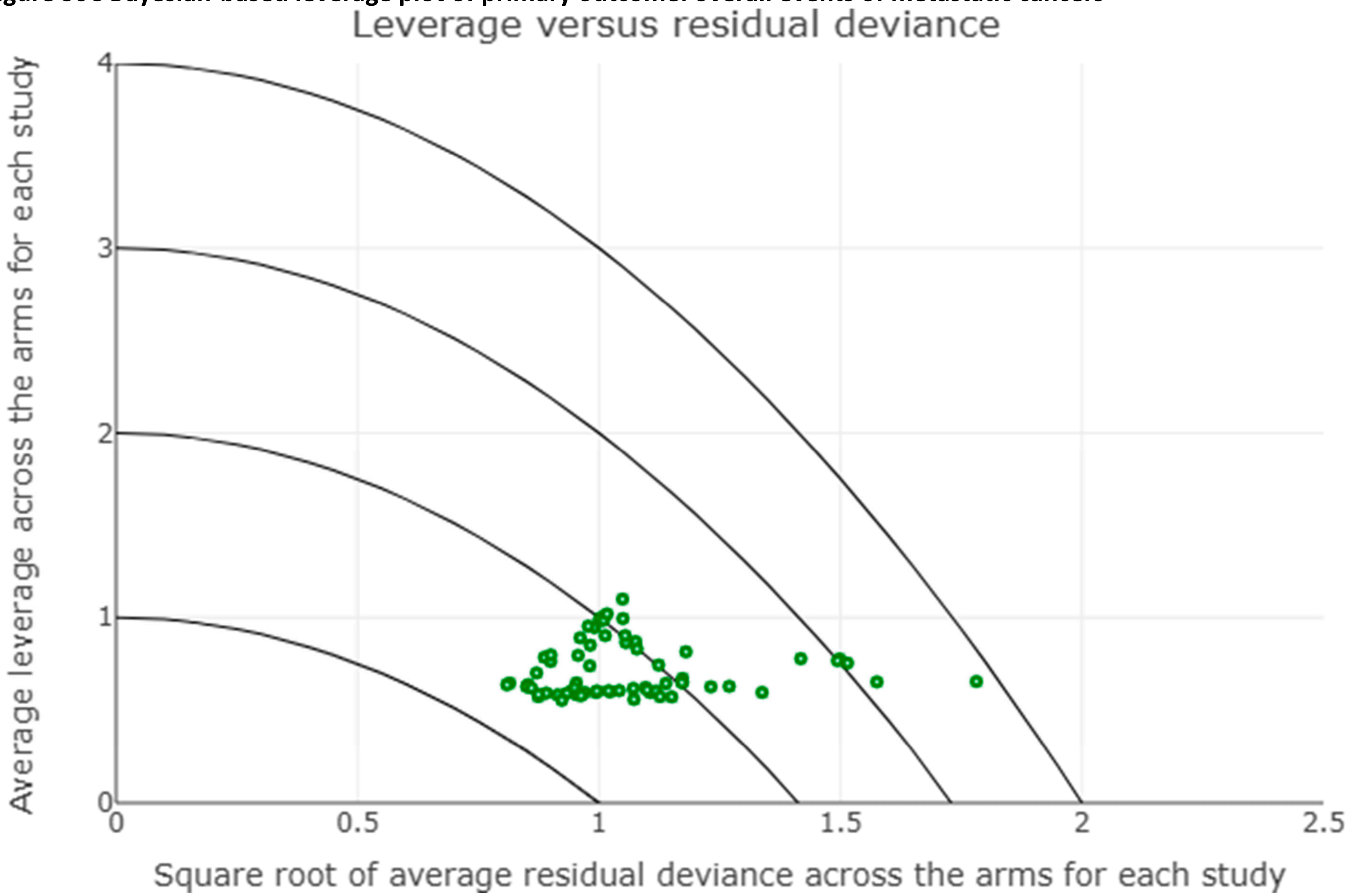

Abbreviation for Figure S6A-6C:

95%CIs: 95% confidence intervals; GLP-1 agonist: glucagon-like peptide-1 agonist; NMA: network meta-analysis; OR: odds ratio; RCT: randomized controlled trial; SGLT2 inhibitor: sodium–glucose cotransporter 2 inhibitor

Figure S7A Funnel plot of primary outcome: overall events of metastatic cancers

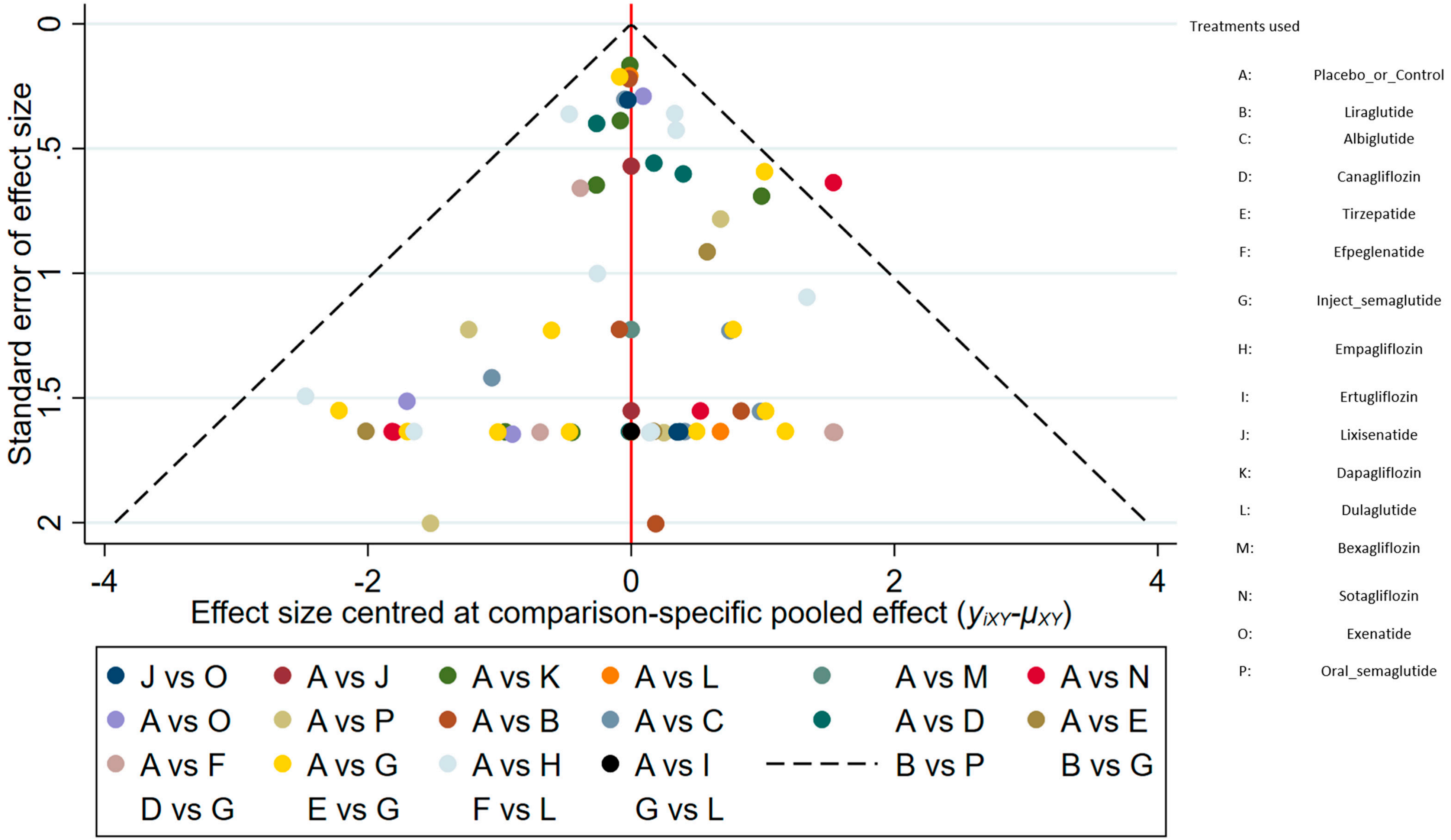

Figure S7B Egger test of primary outcome: overall events of metastatic cancers

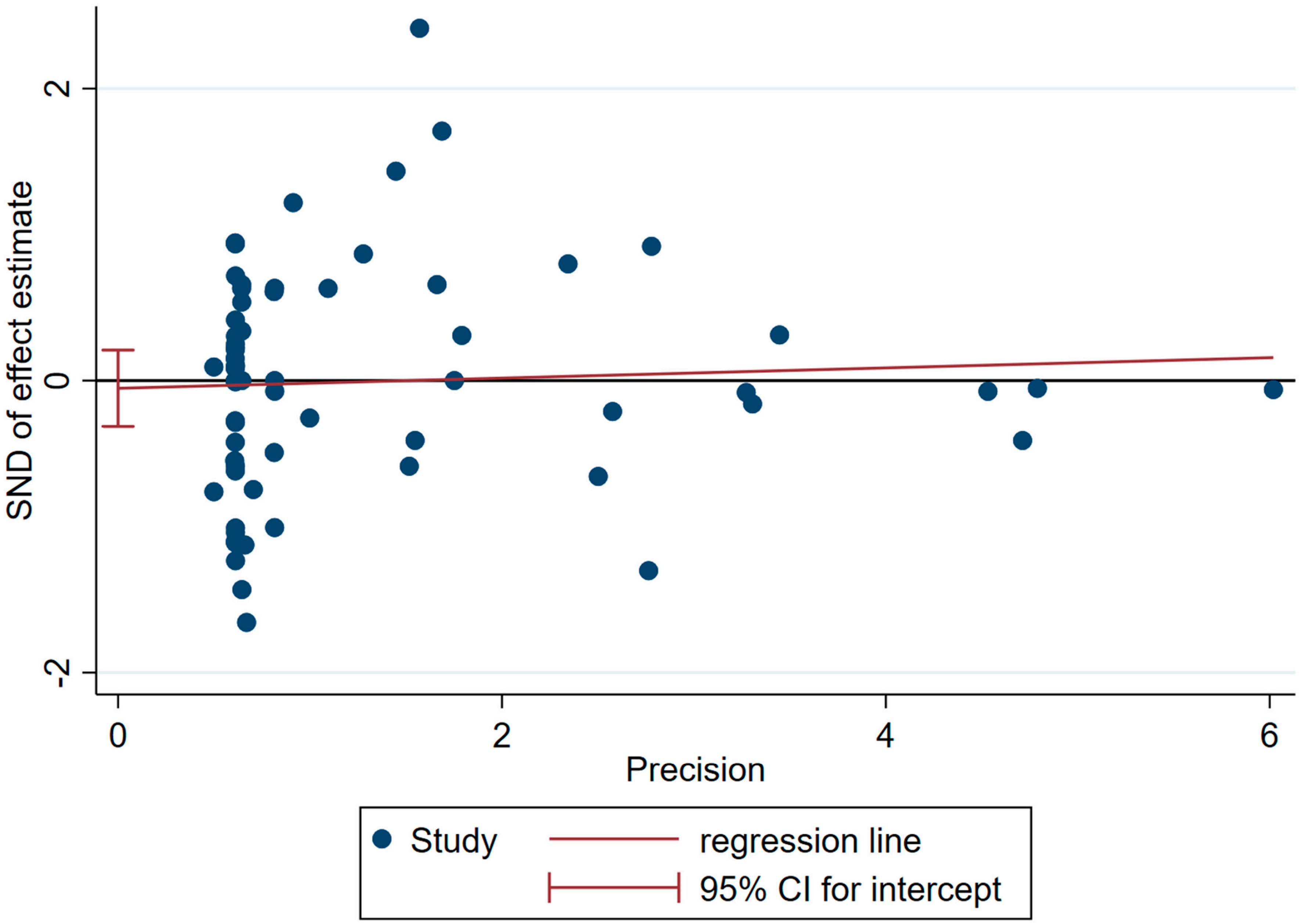

Abbreviation for Figure S7A-7B:

95%CIs: 95% confidence intervals; GLP-1 agonist: glucagon-like peptide-1 agonist; NMA: network meta-analysis; OR: odds ratio; RCT: randomized controlled trial; SGLT2 inhibitor: sodium–glucose cotransporter 2 inhibitor

Figure S8A overview of risk of bias

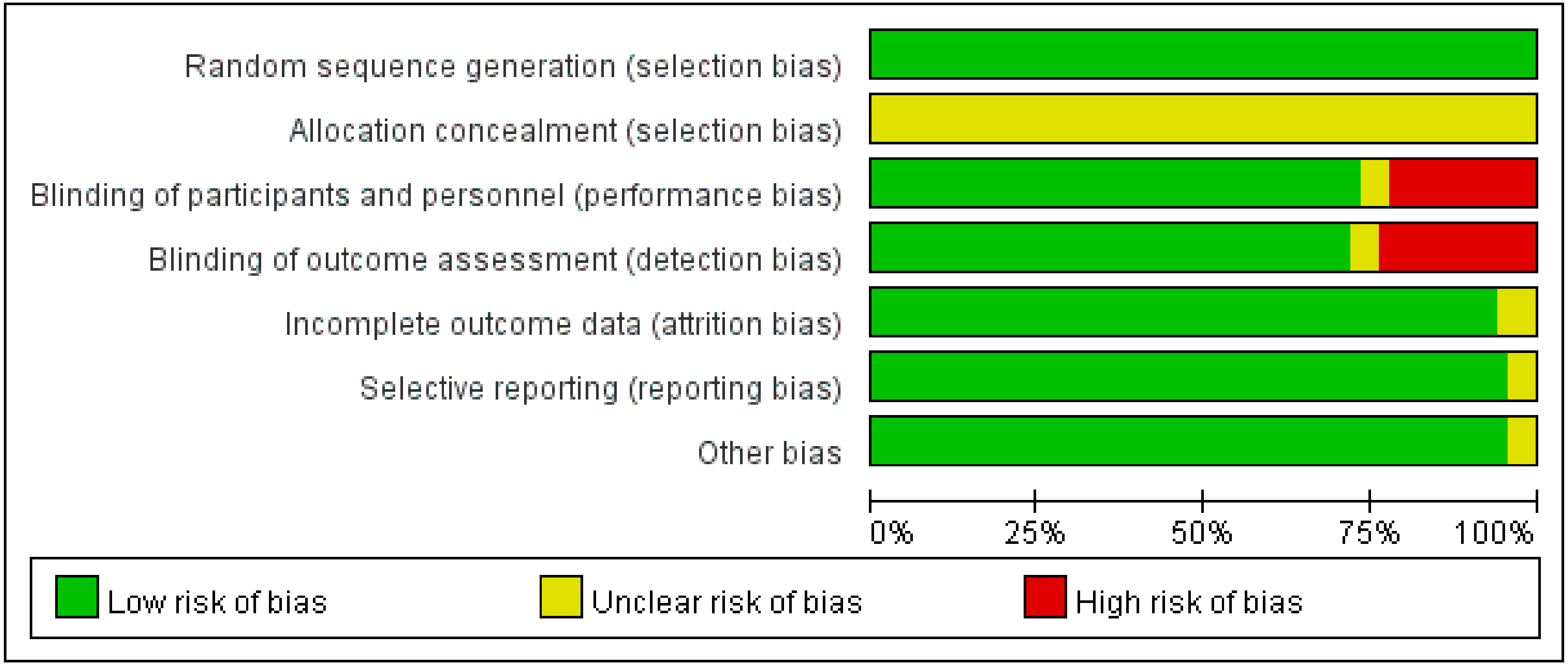

Figure S8B detailed risk of bias in each study

	Random sequence generation (selection bias)	Allocation concealment (selection bias)	Blinding of participants and personnel (performance bias)	Blinding of outcome assessment (detection bias)	Incomplete outcome data (attrition bias)	Selective reporting (reporting bias)	Other bias
Ahren, B. (2017) (SUSTAIN 2)	+	?	+	+	+	+	+
Anker, S.D. (2021) (EMPEROR-Preserved)	+	?	+	+	+	+	+
Aroda, V.R. (2017) (SUSTAIN 4)	+	?	–	–	+	+	+
Aroda, V.R. (2023) (AMPLITUDE-D, NCT03684642)	+	?	–	–	+	+	+
Aroda, V.R. (2023) (AMPLITUDE-L, NCT03713684)	+	?	+	+	+	+	+
Aroda, V.R. (2023) (AMPLITUDE-S, NCT03770728)	+	?	+	+	+	+	+
Aronson, R. (2018) (VERTIS MONO)	+	?	+	+	+	+	+
Barnett, A.H. (2014) (EMPA-REG RENAL)	+	?	+	+	+	+	+
Bhatt, D.L. (2021) (SCORED)	+	?	+	+	+	+	+
Bliddal, H. (2024) (STEP 9)	+	?	+	+	+	+	+
Buse, J.B. (2023) (SEPRA)	+	?	–	–	+	+	+
Cannon, C.P. (2020) (VERTIS CV)	+	?	+	+	+	+	+
Danne, T. (2018) (inTandem2)	+	?	+	+	+	+	+
Del Prato, S. (2021) (SURPASS-4)	+	?	–	–	+	+	+
FLAT-SUGAR Trial Investigators (2016) (FLAT-SUGAR)	+	?	–	–	+	+	+
Frías, J.P. (2021) (SURPASS-2)	+	?	+	+	+	+	+
Frias, J.P. (2022) (AMPLITUDE-M)	+	?	–	–	+	+	+

Gallo, S. (2019) (VERTIS MET)	+	?	+	+	+	+	+
Gallwitz, B. (2012) (EUREXA)	+	?	−	−	+	+	+
Garvey, W.T. (2022) (STEP-5)	+	?	+	+	+	+	+
Gerstein, H.C. (2019) (REWIND)	+	?	+	+	+	+	+
Gerstein, H.C. (2021) (AMPLITUDE-O)	+	?	+	+	+	+	+
Giorgino, F. (2015) (AWARD-2)	+	?	−	−	+	+	+
Heerspink, H.J.L. (2020) (DAPA-CKD)	+	?	+	+	+	+	+
Hernandez, A.F. (2018) (Harmony Outcomes)	+	?	+	+	+	+	+
Herrington, W.G. (2023) (EMPA-KIDNEY)	+	?	+	+	+	+	+
Holman, R.R. (2017) (EXSCEL)	+	?	+	+	+	+	+
Home, P.D. (2017) (HARMONY 1-NCT00849056)	+	?	+	+	?	+	+
Home, P.D. (2017) (HARMONY 3-NCT00838903)	+	?	+	+	+	+	+
Husain, M. (2019) (PIONEER 6)	+	?	+	+	+	+	+
Januzzi, J.L. Jr. (2017) (CR017014)	+	?	+	+	+	+	+
Jastreboff, A.M. (2022) (SURMOUNT-1)	+	?	+	+	+	+	+
Ji, L. (2023)	+	?	+	+	+	+	+
Kaku, K. (2018) (SUSTAIN)	+	?	−	−	+	+	+
Kellerer, M. (2022) (SUSTAIN 11)	+	?	−	−	+	+	+
Lincoff, A.M. (2023) (SELECT)	+	?	+	+	+	+	+
Lingvay, I. (2019) (SUSTAIN 8)	+	?	+	+	+	+	+
Lock, J.P. (2021) (BEST, NCT02558296)	+	?	?	?	?	?	?
Ludvik, B. (2021) (SURPASS-3)	+	?	−	−	+	+	+
Marso, S.P. (2016) (LEADER)	+	?	+	+	+	+	+
Marso, S.P. (2016) (SUSTAIN-6)	+	?	+	+	+	+	+
McMurray, J.J.V. (2019) (DAPA-HF)	+	?	+	+	+	+	+
Mellander, A. (2016) (NCT00528372)	+	?	+	+	+	+	+
Mellander, A. (2016) (NCT00984867)	+	?	+	+	+	+	+
Nauck, M.A. (2016) (HARMONY 2)	+	?	+	+	+	+	+
Neal, B. (2017) (CANVAS)	+	?	+	+	+	+	+
Neal, B. (2017) (CANVAS-R)	+	?	+	+	+	+	+
O'Neil, P.M. (2018) (NCT02453711)	+	?	+	+	+	+	+
Packer, M. (2020) (EMPEROR-Reduced)	+	?	+	+	+	+	+

Perkovic, V. (2019) (CREDENCE)	+	?	+	-	+	+	+
Pfeffer, M.A. (2015) (ELIXA)	+	?	+	+	+	+	+
Pieber, T.R. (2019) (PIONEER 7)	+	?	-	-	+	+	+
Pi-Sunyer, X. (2015) (SCALE)	+	?	+	+	+	+	+
Pratley, R. (2019) (PIONEER 4)	+	?	+	+	+	+	+
Pratley, R.E. (2018) (SUSTAIN 7)	+	?	-	-	+	+	+
Ridderstrale, M. (2014) (EMPA-REG H2H-SU)	+	?	+	+	+	+	+
Roden, M. (2013) (EMPA-REG MONO)	+	?	+	+	+	+	+
Rosenstock, J. (2013) (GetGoal-X)	+	?	-	-	+	+	+
Rosenstock, J. (2019) (PIONEER 3)	+	?	+	+	+	+	+
Rubino, D. (2021) (STEP 4)	+	?	+	+	+	+	+
Solomon, S.D. (2022) (DELIVER)	+	?	+	+	+	+	+
Voors, A.A. (2022) (EMPULSE)	+	?	+	+	+	+	+
Wason, S. (2021) (SOTA-BONE, NCT03386344)	+	?	?	?	?	?	?
Wason, S. (2021) (SOTA-INS, NCT03285594)	+	?	?	?	?	?	?
Weissman, P.N. (2014) (HARMONY 4)	+	?	-	-	+	+	+
Wiviott, S.D. (2019) (DECLARE-TIMI 58)	+	?	+	+	+	+	+
Zinman, B. (2015) (EMPA-REG OUTCOME)	+	?	+	+	+	+	+

Table S1: PRISMA 2020 checklist of the current network meta-analysis

Section and Topic	Item #	Checklist item	Page where item is reported
**TITLE**			
Title	1	Identify the report as a systematic review.	1
**ABSTRACT**			
Abstract	2	See the PRISMA 2020 for Abstracts checklist.	7-8
**INTRODUCTION**			
Rationale	3	Describe the rationale for the review in the context of existing knowledge.	9-10
Objectives	4	Provide an explicit statement of the objective(s) or question(s) the review addresses.	9-10
**METHODS**			
Eligibility criteria	5	Specify the inclusion and exclusion criteria for the review and how studies were grouped for the syntheses.	11-12
Information sources	6	Specify all databases, registers, websites, organisations, reference lists and other sources searched or consulted to identify studies. Specify the date when each source was last searched or consulted.	11-12
Search strategy	7	Present the full search strategies for all databases, registers and websites, including any filters and limits used.	11-12
Selection process	8	Specify the methods used to decide whether a study met the inclusion criteria of the review, including how many reviewers screened each record and each report retrieved, whether they worked independently, and if applicable, details of automation tools used in the process.	11-12
Data collection process	9	Specify the methods used to collect data from reports, including how many reviewers collected data from each report, whether they worked independently, any processes for obtaining or confirming data from study investigators, and if applicable, details of automation tools used in the process.	11-12
Data items	10a	List and define all outcomes for which data were sought. Specify whether all results that were compatible with each outcome domain in each study were sought (e.g. for all measures, time points, analyses), and if not, the methods used to decide which results to collect.	12-13
	10b	List and define all other variables for which data were sought (e.g. participant and intervention characteristics, funding sources). Describe any assumptions made about any missing or unclear information.	12-13
Study risk of bias assessment	11	Specify the methods used to assess risk of bias in the included studies, including details of the tool(s) used, how many reviewers assessed each study and whether they worked independently, and if applicable, details of automation tools used in the process.	12-13
Effect measures	12	Specify for each outcome the effect measure(s) (e.g. risk ratio, mean difference) used in the synthesis or presentation of results.	12-13
Synthesis methods	13a	Describe the processes used to decide which studies were eligible for each synthesis (e.g. tabulating the study intervention characteristics and comparing against the planned groups for each synthesis (item #5)).	12-13
	13b	Describe any methods required to prepare the data for presentation or synthesis, such as handling of missing summary statistics, or data conversions.	13-15
	13c	Describe any methods used to tabulate or visually display results of individual studies and syntheses.	13-15
	13d	Describe any methods used to synthesize results and provide a rationale for the choice(s). If meta-analysis was performed, describe the model(s), method(s) to identify the presence and extent of statistical heterogeneity, and software package(s) used.	13-15
	13e	Describe any methods used to explore possible causes of heterogeneity among study results (e.g. subgroup analysis, meta-regression).	13-15
	13f	Describe any sensitivity analyses conducted to assess robustness of the synthesized results.	13-15
Reporting bias assessment	14	Describe any methods used to assess risk of bias due to missing results in a synthesis (arising from reporting biases).	13-15

Section and Topic	Item #	Checklist item	Page where item is reported
Certainty assessment	15	Describe any methods used to assess certainty (or confidence) in the body of evidence for an outcome.	13-15
**RESULTS**			
Study selection	16a	Describe the results of the search and selection process, from the number of records identified in the search to the number of studies included in the review, ideally using a flow diagram.	16-17, Fig 1, eTab 2
	16b	Cite studies that might appear to meet the inclusion criteria, but which were excluded, and explain why they were excluded.	16-17, eTab 3
Study characteristics	17	Cite each included study and present its characteristics.	16-17, eTab 4
Risk of bias in studies	18	Present assessments of risk of bias for each included study.	16-17, eFig 8
Results of individual studies	19	For all outcomes, present, for each study: (a) summary statistics for each group (where appropriate) and (b) an effect estimate and its precision (e.g. confidence/credible interval), ideally using structured tables or plots.	16-17, eFig 3
Results of syntheses	20a	For each synthesis, briefly summarise the characteristics and risk of bias among contributing studies.	17-18, Fig 2
	20b	Present results of all statistical syntheses conducted. If meta-analysis was done, present for each the summary estimate and its precision (e.g. confidence/credible interval) and measures of statistical heterogeneity. If comparing groups, describe the direction of the effect.	17-18, Fig 3
	20c	Present results of all investigations of possible causes of heterogeneity among study results.	17-18, eTab 7
	20d	Present results of all sensitivity analyses conducted to assess the robustness of the synthesized results.	17-18
Reporting biases	21	Present assessments of risk of bias due to missing results (arising from reporting biases) for each synthesis assessed.	17-18, eFig 8
Certainty of evidence	22	Present assessments of certainty (or confidence) in the body of evidence for each outcome assessed.	17-18
**DISCUSSION**			
Discussion	23a	Provide a general interpretation of the results in the context of other evidence.	19-21
	23b	Discuss any limitations of the evidence included in the review.	21-22
	23c	Discuss any limitations of the review processes used.	21-22
	23d	Discuss implications of the results for practice, policy, and future research.	23
**OTHER INFORMATION**			
Registration and protocol	24a	Provide registration information for the review, including register name and registration number, or state that the review was not registered.	8
	24b	Indicate where the review protocol can be accessed, or state that a protocol was not prepared.	8
	24c	Describe and explain any amendments to information provided at registration or in the protocol.	8
Support	25	Describe sources of financial or non-financial support for the review, and the role of the funders or sponsors in the review.	24
Competing interests	26	Declare any competing interests of review authors.	24
Availability of data, code and other materials	27	Report which of the following are publicly available and where they can be found: template data collection forms; data extracted from included studies; data used for all analyses; analytic code; any other materials used in the review.	24

The current checklist followed the latest PRISMA 2020 guideline [1].

Table S2: Keyword used in each database and search results

Database	Keyword	Filter	Date	Result
PubMed	(metastatic OR metastasis) AND (cancer OR tumor) AND (glucagon-like peptide-1 receptor agonist OR Sodium Glucose Cotransporter 2 Inhibitor OR lixisenatide OR orforglipron OR exenatide OR semaglutide OR liraglutide OR albiglutide OR dulaglutide OR tirzepatide OR bexagliflozin OR canagliflozin OR dapagliflozin OR empagliflozin OR ertugliflozin OR ipragliflozin OR luseogliflozin OR remogliflozin OR sergliflozin OR sotagliflozin OR tofogliflozin OR henagliflozin OR janagliflozin OR mizagliflozin OR velagliflozin OR enavogliflozin OR licogliflozin OR rongliflozin) AND (random OR randomized OR randomised)	N/A	2024/11/13	49
ClinicalKey	(metastatic OR metastasis) AND (cancer OR tumor) AND (glucagon-like peptide-1 receptor agonist OR Sodium Glucose Cotransporter 2 Inhibitor OR lixisenatide OR orforglipron OR exenatide OR semaglutide OR liraglutide OR albiglutide OR dulaglutide OR tirzepatide OR bexagliflozin OR canagliflozin OR dapagliflozin OR empagliflozin OR ertugliflozin OR ipragliflozin OR luseogliflozin OR remogliflozin OR sergliflozin OR sotagliflozin OR tofogliflozin OR henagliflozin OR janagliflozin OR mizagliflozin OR velagliflozin OR enavogliflozin OR licogliflozin OR rongliflozin) AND (random OR randomized OR randomised)	N/A	2024/11/13	860
Embase	(metastatic OR metastasis) AND (cancer OR tumor) AND (glucagon-like peptide-1 receptor agonist OR Sodium Glucose Cotransporter 2 Inhibitor) AND (random OR randomized OR	N/A	2024/11/13	46

	randomised)			
Cochrane CENTRAL	(metastatic OR metastasis) AND (cancer OR tumor) AND (glucagon-like peptide-1 receptor agonist OR Sodium Glucose Cotransporter 2 Inhibitor OR lixisenatide OR orforglipron OR exenatide OR semaglutide OR liraglutide OR albiglutide OR dulaglutide OR tirzepatide OR bexagliflozin OR canagliflozin OR dapagliflozin OR empagliflozin OR ertugliflozin OR ipragliflozin OR luseogliflozin OR remogliflozin OR sergliflozin OR sotagliflozin OR tofogliflozin OR henagliflozin OR janagliflozin OR mizagliflozin OR velagliflozin OR enavogliflozin OR licogliflozin OR rongliflozin) AND (random OR randomized OR randomised)	N/A	2024/11/13	3
ProQuest	(metastatic OR metastasis) AND (cancer OR tumor) AND (glucagon-like peptide-1 receptor agonist OR Sodium Glucose Cotransporter 2 Inhibitor) AND (random OR randomized OR randomised)	N/A	2024/11/13	1412
ScienceDirect	(metastatic OR metastasis) AND (cancer OR tumor) AND (glucagon-like peptide-1 receptor agonist OR Sodium Glucose Cotransporter 2 Inhibitor) AND (random OR randomized OR randomised)	N/A	2024/11/13	1535
Web of Science	(metastatic OR metastasis) AND (cancer OR tumor) AND (glucagon-like peptide-1 receptor agonist OR Sodium Glucose Cotransporter 2 Inhibitor) AND (random OR randomized OR randomised)	N/A	2024/11/13	1
ClinicalTrials.gov	(metastatic OR metastasis) AND (cancer OR tumor) AND (glucagon-like peptide-1 receptor agonist OR Sodium Glucose Cotransporter 2 Inhibitor OR lixisenatide OR orforglipron OR exenatide OR semaglutide OR liraglutide OR albiglutide OR dulaglutide OR tirzepatide OR bexagliflozin OR canagliflozin OR dapagliflozin OR empagliflozin OR ertugliflozin OR	N/A	2024/11/13	1

	ipragliflozin OR luseogliflozin OR remogliflozin OR sergliflozin OR sotagliflozin OR tofogliflozin OR henagliflozin OR janagliflozin OR mizagliflozin OR velagliflozin OR enavogliflozin OR licogliflozin OR rongliflozin) AND (random OR randomized OR randomised)

Abbreviation: N/A: not applied

Table S3: Excluded studies and reason

Reason	Numbers	References
Duplicate sample source with other included trial	1	[2]
Meta-analysis	5	[3-7]
Not randomized controlled trial	1	[8]
Not report target outcome	74	[9-82]
Protocol but not result of a trial	1	[83]

Table S4: Characteristics of the included studies

Study name	Baseline illness	Comparison	Subjects	Mean age (year)	Female (%)	Treatment duration	Category	ClinicalTrials.gov	Country
Bliddal, H. (2024) (STEP 9)[84]	patients with obesity and knee osteoarthritis	Inject semaglutide 2.4 mg/week<br>Placebo	271<br>136	56.0±10.0<br>56.0±10.0	84.1<br>76.5	68 weeks	GLP-1 agonist	NCT05064735	Multiple countries
Aroda, V.R. (2023) (AMPLITUDE-D, NCT03684642)[85]	patients with type 2 diabetes mellitus	Efpeglenatide 4 mg<br>Efpeglenatide 6 mg<br>Dulaglutide 1.5 mg	303<br>302<br>303	60.3±9.6<br>60.0±10.1<br>59.4±10.1	46.9<br>52.0<br>50.5	56 weeks	GLP-1 agonist	NCT03684642	Multiple countries
Aroda, V.R. (2023) (AMPLITUDE-L, NCT03713684)[85]	patients with type 2 diabetes mellitus	Efpeglenatide 2 mg<br>Efpeglenatide 4 mg<br>Efpeglenatide 6 mg<br>Placebo	92<br>93<br>93<br>92	59.1±10.7<br>60.6±11.5<br>61.6±10.3<br>58.9±10.7	45.7<br>45.2<br>43.0<br>53.3	56 weeks	GLP-1 agonist	NCT03713684	Multiple countries
Aroda, V.R. (2023) (AMPLITUDE-S, NCT03770728)[85]	patients with type 2 diabetes mellitus	Efpeglenatide 2 mg<br>Efpeglenatide 4 mg<br>Efpeglenatide 6 mg<br>Placebo	78<br>77<br>78<br>79	60.1±10.9<br>57.9±10.5<br>58.8±11.5<br>58.9±10.6	43.6<br>42.9<br>50.0<br>43.0	30 weeks	GLP-1 agonist	NCT03770728	Multiple countries
Buse, J.B. (2023) (SEPRA)[86]	patients with type 2 diabetes mellitus	Inject semaglutide<br>Control with standard care	644<br>634	57.5±11.3<br>57.2±11.0	48.1<br>43.5	104 weeks	GLP-1 agonist	NCT03596450	Multiple countries
Herrington, W.G. (2023) (EMPA-KIDNEY)[87]	patients with renal failure	Empagliflozin 10mg/day<br>Placebo	3304<br>3305	63.9±13.9<br>63.8±13.9	33.2<br>33.1	104 weeks	SGLT2 inhibitor	NCT03594110	Multiple countries
Ji, L. (2023)[88]	patients with type 2 diabetes mellitus	Empagliflozin 10mg/day<br>Empagliflozin 25mg/day<br>Placebo	73<br>73<br>73	59.9±7.7<br>60.7±9.1<br>60.1±8.0	41.1<br>45.2<br>50.7	24 weeks	SGLT2 inhibitor	NCT04233801	China
Lincoff, A.M. (2023) (SELECT)[89]	patients with obesity	Inject Semaglutide 2.4mg<br>Placebo	8803<br>8801	61.6±8.9<br>61.6±8.8	27.8<br>27.5	104 weeks	GLP-1 agonist	NCT03574597	Multiple countries
Frias, J.P. (2022) (AMPLITUDE-M)[90]	patients with type 2 diabetes mellitus	Efpeglenatide 2 mg<br>Efpeglenatide 4 mg<br>Efpeglenatide 6 mg<br>Placebo	100<br>101<br>103<br>102	58.6±10.5<br>56.3±11.5<br>59.6±10.7<br>59.5±11.7	55.0<br>51.5<br>59.2<br>50.0	62 weeks	GLP-1 agonist	NCT03353350	Multiple countries

Garvey, W.T. (2022) (STEP-5)[91]	patients with obesity	Inject semaglutide 2.4 mg/week<br>Placebo	152<br>152	47.3±11.7<br>47.4±10.3	80.9<br>74.3	104 weeks	GLP-1 agonist	NCT03693430	Multiple countries
Jastreboff, A.M. (2022) (SURMOUNT-1)[92]	patients with obesity	Tirzepatide 5mg<br>Tirzepatide 10mg<br>Tirzepatide 15mg<br>Placebo	630<br>636<br>630<br>643	45.6±12.7<br>44.7±12.4<br>44.9±12.3<br>44.4±12.5	67.6<br>67.1<br>67.5<br>67.8	72 weeks	GLP-1 agonist	NCT04184622	Multiple countries
Kellerer, M. (2022) (SUSTAIN 11)[93]	patients with type 2 diabetes mellitus	Inject semaglutide 1.0 mg<br>Control with insulin glargine and aspart	874<br>874	60.8±9.4<br>61.5±9.5	49.1<br>48.6	52 weeks	GLP-1 agonist	NCT03689374	Multiple countries
Solomon, S.D. (2022) (DELIVER)[94]	patients with stabilized heart failure	Dapagliflozin 10mg/day<br>Placebo	3131<br>3132	71.8±9.6<br>71.5±9.5	43.6<br>44.2	120 weeks	SGLT2 inhibitor	NCT03619213	Multiple countries
Voors, A.A. (2022) (EMPULSE)[95]	patients with acute heart failure and dyspnea	Empagliflozin 10mg/day<br>Placebo	265<br>265	71.0<br>70.0	32.5<br>35.1	13 weeks	SGLT2 inhibitor	NCT04157751	Multiple countries
Anker, S.D. (2021) (EMPEROR-Preserved)[96]	patients with heart failure with preserved ejection fraction	Empagliflozin 10mg/day<br>Placebo	2997<br>2991	71.8±9.3<br>71.9±9.6	44.6<br>44.7	156 weeks	SGLT2 inhibitor	NCT03057951	Multiple countries
Bhatt, D.L. (2021) (SCORED)[97]	patients with type 2 diabetes mellitus and chronic kidney disease	Sotagliflozin 200-400mg/day<br>Placebo	5292<br>5292	68.4±8.4<br>68.2±8.4	44.3<br>45.5	116 weeks	SGLT2 inhibitor	NCT03315143	Multiple countries
Del Prato, S. (2021) (SURPASS-4)[98]	patients with type 2 diabetes mellitus	Tirzepatide 5mg<br>Tirzepatide 10mg<br>Tirzepatide 15mg<br>Controls with insulin glargine	329<br>328<br>338<br>1000	62.9±8.6<br>63.7±8.7<br>63.7±8.6<br>63.8±8.5	39.8<br>36.3<br>39.9<br>36.4	108 weeks	GLP-1 agonist	NCT03730662	Multiple countries
Frías, J.P. (2021) (SURPASS-2)[99]	patients with type 2 diabetes mellitus	Tirzepatide 5mg<br>Tirzepatide 10mg<br>Tirzepatide 15mg<br>Inject semaglutide 1.0 mg	470<br>469<br>470<br>469	56.3±10.0<br>57.2±10.5<br>55.9±10.4<br>56.9±10.8	56.4<br>49.3<br>54.5<br>52.0	40 weeks	GLP-1 agonist	NCT03987919	Multiple countries

Gerstein, H.C. (2021) (AMPLITUDE-O)[100]	patients with type 2 diabetes mellitus	Efpeglenatide 4 mg<br>Efpeglenatide 6 mg<br>Placebo	1359<br>1358<br>1359	64.6±8.2<br>64.7±8.2<br>64.4±8.3	32.5<br>35.6<br>30.8	104 weeks	GLP-1 agonist	NCT03496298	Multiple countries
Lock, J.P. (2021) (BEST, NCT02558296)[101]	patients with type 2 diabetes mellitus	Bexagliflozin 20mg<br>Placebo	1132<br>567	64.4±7.9<br>64.6±8.0	30.1<br>31.2	52 weeks	SGLT2 inhibitor	NCT02558296	Multiple countries
Ludvik, B. (2021) (SURPASS-3)[102]	patients with type 2 diabetes mellitus	Tirzepatide 5mg<br>Tirzepatide 10mg<br>Tirzepatide 15mg<br>Controls with insulin degludec	358<br>360<br>359<br>360	57.2±10.1<br>57.4±9.7<br>57.5±10.2<br>57.5±10.1	44.1<br>45.8<br>46.0<br>40.8	67 weeks	GLP-1 agonist	NCT03882970	Multiple countries
Rubino, D. (2021) (STEP 4)[103]	patients with overweight or obesity	Inject semaglutide 2.4 mg/week<br>Placebo	535<br>268	47.0±12.0<br>46.0±12.0	80.2<br>76.5	68 weeks	GLP-1 agonist	NCT03548987	Multiple countries
Wason, S. (2021) (SOTA-BONE, NCT03386344)[104]	patients with type 2 diabetes mellitus	Sotagliflozin 200-400mg/day<br>Placebo	250<br>126	66.3±6.8<br>66.3±5.7	44.4<br>44.4	110 weeks	SGLT2 inhibitor	NCT03386344	Multiple countries
Wason, S. (2021) (SOTA-INS, NCT03285594)[105]	patients with type 2 diabetes mellitus	Sotagliflozin 200-400mg/day<br>Placebo	427<br>144	62.5±9.5<br>62.2±8.9	46.2<br>40.3	52 weeks	SGLT2 inhibitor	NCT03285594	Multiple countries
Cannon, C.P. (2020) (VERTIS CV)[106]	patients with type 2 diabetes mellitus	Ertugliflozin 5 mg/day<br>Ertugliflozin 15 mg/day<br>Placebo	2752<br>2747<br>2747	64.3±8.2<br>64.4±8.0<br>64.4±8.0	29.1<br>30.3<br>30.7	182 weeks	SGLT2 inhibitor	NCT01986881	Multiple countries
Heerspink, H.J.L. (2020) (DAPA-CKD)[107]	patients with renal failure	Dapagliflozin 10mg/day<br>Placebo	2152<br>2152	61.8±12.1<br>61.9±12.1	32.9<br>33.3	125 weeks	SGLT2 inhibitor	NCT03036150	Multiple countries
Packer, M. (2020) (EMPEROR-Reduced)[108]	patients with chronic heart failure	Empagliflozin 10mg/day<br>Placebo	1863<br>1867	67.2±10.8<br>66.5±11.2	23.5<br>24.4	64 weeks	SGLT2 inhibitor	NCT03057977	Multiple countries
Gallo, S. (2019) (VERTIS MET)[109]	patients with type 2 diabetes mellitus	Ertugliflozin 5 mg/day<br>Ertugliflozin 15 mg/day<br>Placebo with glimepiride	207<br>205<br>209	56.6±8.2<br>56.9±9.4<br>56.5±8.7	53.1<br>54.6<br>53.1	104 weeks	SGLT2 inhibitor	NCT02033889	Multiple countries
Gerstein, H.C. (2019) (REWIND)[110]	patients with type 2 diabetes mellitus	Dulaglutide 1.5 mg<br>Placebo	4949<br>4952	66.2±6.5<br>66.2±6.5	46.6<br>46.1	281 weeks	GLP-1 agonist	NCT01394952	Multiple countries
Husain, M. (2019) (PIONEER 6)[111]	patients with cardiovascular disease	Semaglutide 14mg<br>Placebo	1591<br>1592	66.0±7.0<br>66.0±7.0	31.9<br>31.4	64 weeks	GLP-1 agonist	NCT02692716	Multiple countries

	or chronic kidney disease								
Lingvay, I. (2019) (SUSTAIN 8)[112]	patients with type 2 diabetes mellitus	Inject semaglutide 1.0 mg<br>Canagliflozin 300 mg	394<br>394	55.7±11.1<br>57.5±10.7	43.4<br>49.0	52 weeks	GLP-1 agonist/SGLT2 inhibitor	NCT03136484	Multiple countries
McMurray, J.J.V. (2019) (DAPA-HF)[113]	patients with stabilized heart failure	Dapagliflozin 10mg/day<br>Placebo	2373<br>2371	66.2±11.0<br>66.5±10.8	23.8<br>23.0	73 weeks	SGLT2 inhibitor	NCT03036124	Multiple countries
Perkovic, V. (2019) (CREDENCE)[114]	patients with type 2 diabetes mellitus	Canagliflozin 100 mg<br>Placebo	2202<br>2199	62.9±9.2<br>63.2±9.2	34.6<br>33.3	130 weeks	SGLT2 inhibitor	NCT02065791	Multiple countries
Pieber, T.R. (2019) (PIONEER 7)[115]	patients with type 2 diabetes mellitus	Semaglutide 3-14mg<br>Control with sitagliptin	253<br>251	56.9±9.7<br>57.9±10.1	42.7<br>44.2	52 weeks	GLP-1 agonist	NCT02849080	Multiple countries
Pratley, R. (2019) (PIONEER 4)[116]	patients with type 2 diabetes mellitus	Semaglutide 14mg<br>Liraglutide 1.8mg<br>Placebo	285<br>284<br>142	56.0±10.0<br>56.0±10.0<br>57.0±10.0	51.6<br>52.5<br>52.1	52 weeks	GLP-1 agonist	NCT02863419	Multiple countries
Rosenstock, J. (2019) (PIONEER 3)[117]	patients with type 2 diabetes mellitus	Semaglutide 3-14mg<br>Control with sitagliptin	1396<br>466	57.7±10.0<br>58.0±10.0	46.6<br>49.0	78 weeks	GLP-1 agonist	NCT02607865	Multiple countries
Wiviott, S.D. (2019) (DECLARE-TIMI 58)[118]	patients with atherosclerotic vascular disease	Dapagliflozin 10mg/day<br>Placebo	8582<br>8578	63.9±6.8<br>64.0±6.8	36.9<br>37.9	206 weeks	SGLT2 inhibitor	NCT01730534	Multiple countries
Aronson, R. (2018) (VERTIS MONO)[119]	patients with type 2 diabetes mellitus	Ertugliflozin 5 mg/day<br>Ertugliflozin 15 mg/day<br>Placebo	156<br>152<br>153	56.8±11.4<br>56.2±10.8<br>56.1±10.9	42.9<br>40.8<br>46.4	52 weeks	SGLT2 inhibitor	NCT01958671	Multiple countries
Danne, T. (2018) (inTandem2)[120]	patients with type 1 diabetes mellitus	Sotagliflozin 200-400mg/day<br>Placebo	524<br>258	42.0±13.4<br>39.7±13.4	48.1<br>48.1	52 weeks	SGLT2 inhibitor	NCT02421510	Multiple countries
Hernandez, A.F. (2018) (Harmony Outcomes)[121]	patients with type 2 diabetes mellitus	Albiglutide initial 30-50 mg/day<br>Placebo	4731<br>4732	64.1±8.7<br>64.2±8.7	30.2<br>31.0	86 weeks	GLP-1 agonist	NCT02465515	Multiple countries
Kaku, K. (2018) (SUSTAIN)[122]	patients with type 2 diabetes mellitus	Inject semaglutide 0.5 mg<br>Inject semaglutide 1.0 mg	239<br>241<br>121	58.0±10.6<br>58.7±10.2<br>59.2±10.1	30.5<br>27.8<br>25.8	61 weeks	GLP-1 agonist	NCT02207374	Multiple countries

		Control with standard care							
O'Neil, P.M. (2018) (NCT02453711)[123]	patients with obesity	Inject semaglutide 0.05-0.4 mg<br>Liraglutide 3.0mg<br>Placebo	718<br>103<br>136	46.4±12.6<br>48.5±11.2<br>46.4±12.8	64.6<br>65.0<br>64.7	52 weeks	GLP-1 agonist	NCT02453711	Multiple countries
Pratley, R.E. (2018) (SUSTAIN 7)[124]	patients with type 2 diabetes mellitus	Inject semaglutide 0.5 mg<br>Inject semaglutide 1.0 mg<br>Dulaglutide 0.75-1.5 mg	301<br>300<br>598	56.0±10.9<br>55.0±10.6<br>55.5±10.5	43.9<br>46.0<br>44.7	40 weeks	GLP-1 agonist	NCT02648204	Multiple countries
Ahren, B. (2017) (SUSTAIN 2)[125]	patients with type 2 diabetes mellitus	Inject semaglutide 1.0 mg<br>Inject semaglutide 0.5 mg<br>Placebo	409<br>409<br>407	56.0±9.4<br>54.8±10.2<br>54.6±10.4	49.9<br>50.6<br>51.1	56 weeks	GLP-1 agonist	NCT01930188	Multiple countries
Aroda, V.R. (2017) (SUSTAIN 4)[126]	patients with type 2 diabetes mellitus	Inject semaglutide 0.5 mg<br>Inject semaglutide 1.0 mg<br>Control with insulin glargine	362<br>360<br>360	56.5±10.3<br>56.7±10.4<br>56.2±10.6	45.6<br>49.4<br>45.8	30 weeks	GLP-1 agonist	NCT02128932	Multiple countries
Holman, R.R. (2017) (EXSCEL)[127]	patients with type 2 diabetes mellitus	Exenatide 2mg<br>Placebo	7356<br>7396	61.8±9.4<br>61.9±9.4	38.0<br>38.0	166 weeks	GLP-1 agonist	NCT01144338	Multiple countries
Home, P.D. (2017) (HARMONY 1-NCT00849056)[31]	patients with type 2 diabetes mellitus	Albiglutide 30 mg<br>Placebo	150<br>151	55.2±10.0<br>54.9±9.4	38.7<br>41.7	52 weeks	GLP-1 agonist	NCT00849056	Multiple countries
Home, P.D. (2017) (HARMONY 3-NCT00838903)[31]	patients with type 2 diabetes mellitus	Albiglutide 30 mg<br>Placebo	302<br>101	54.3±10.1<br>56.1±10.0	55.3<br>50.5	104 weeks	GLP-1 agonist	NCT00838903	Multiple countries
Januzzi, J.L. Jr. (2017) (CR017014)[128]	patients with type 2 diabetes mellitus	Canagliflozin 100 mg<br>Canagliflozin 300 mg<br>Placebo	241<br>236<br>237	64.3±6.5<br>63.4±6.0<br>63.2±6.2	48.5<br>45.3<br>39.7	104 weeks	SGLT2 inhibitor	NCT01106651	Multiple countries

Neal, B. (2017) (CANVAS)[129]	patients with type 2 diabetes mellitus	Canagliflozin 100 mg<br>Canagliflozin 300 mg<br>Placebo	1445<br>1443<br>1442	62.2±8.0<br>62.8±8.1<br>62.3±7.9	33.5<br>34.6<br>33.7	126 weeks	SGLT2 inhibitor	NCT01032629	Multiple countries
Neal, B. (2017) (CANVAS-R)[129]	patients with type 2 diabetes mellitus	Canagliflozin 300 mg<br>Placebo	2907<br>2905	63.9±8.4<br>64.0±8.3	36.2<br>38.2	126 weeks	SGLT2 inhibitor	NCT01989754	Multiple countries
FLAT-SUGAR Trial Investigators (2016) (FLAT-SUGAR)[130]	patients with type 2 diabetes mellitus	Exenatide<br>Control with insulin glargine	52<br>50	62.0±8.0<br>63.0±7.0	30.8<br>44.0	26 weeks	GLP-1 agonist	NCT01524705	Multiple countries
Marso, S.P. (2016) (LEADER)[131]	patients with type 2 diabetes mellitus	Liraglutide 1.8mg<br>Placebo	4668<br>4672	64.2±7.2<br>64.4±7.2	35.5<br>36.0	198 weeks	GLP-1 agonist	NCT01179048	Multiple countries
Marso, S.P. (2016) (SUSTAIN-6)[132]	patients with type 2 diabetes mellitus	Inject semaglutide 0.5 mg<br>Inject semaglutide 1.0 mg<br>Placebo	826<br>822<br>1649	NA	40.1<br>37.0<br>40.0	109 weeks	GLP-1 agonist	NCT01720446	Multiple countries
Mellander, A. (2016) (NCT00528372)[133]	patients with type 2 diabetes mellitus	Dapagliflozin 2.5 mg<br>Dapagliflozin 5 mg<br>Dapagliflozin 10 mg<br>Placebo	132<br>132<br>146<br>75	NA	50.7<br>54.6<br>50.0<br>58.7	102 weeks	SGLT2 inhibitor	NCT00528372	Multiple countries
Mellander, A. (2016) (NCT00984867)[133]	patients with type 2 diabetes mellitus	Dapagliflozin 10mg/day<br>Placebo	223<br>224	54.8±10.4<br>55.0±10.2	43.0<br>47.3	48 weeks	SGLT2 inhibitor	NCT00984867	Multiple countries
Nauck, M.A. (2016) (HARMONY 2)[134]	patients with type 2 diabetes mellitus	Albiglutide 30-50 mg<br>Placebo	200<br>101	52.8±11.4<br>53.1±11.7	46.0<br>42.6	52 weeks	GLP-1 agonist	NCT00849017	Multiple countries
Giorgino, F. (2015) (AWARD-2)[135]	patients with type 2 diabetes mellitus	Dulaglutide 0.75-1.5 mg<br>Control with insulin glargine	545<br>262	56.5±9.5<br>57.0±9.0	48.6<br>48.9	78 weeks	GLP-1 agonist	NCT01075282	Multiple countries
Pfeffer, M.A. (2015) (ELIXA)[136]	patients with type 2 diabetes mellitus and recent acute coronary syndrome	Lixisenatide 20ug<br>Placebo	3034<br>3034	59.9±9.7<br>60.6±9.6	30.4<br>30.9	100 weeks	GLP-1 agonist	NCT01147250	Multiple countries
Pi-Sunyer, X. (2015) (SCALE) (before 56 weeks)[137]	patients with obesity	Liraglutide 3.0mg<br>Placebo	2487<br>1244	45.2±12.1<br>45.0±12.0	78.7<br>78.1	56 weeks	GLP-1 agonist	NCT01272219	Multiple countries

Zinman, B. (2015) (EMPA-REG OUTCOME)[138]	patients with type 2 diabetes mellitus	Empagliflozin 10mg/day<br>Empagliflozin 25mg/day<br>Placebo	2345<br>2342<br>2333	63.0±8.6<br>63.2±8.6<br>63.2±8.8	29.5<br>28.1<br>28.0	135 weeks	SGLT2 inhibitor	NCT01131676	Multiple countries
Barnett, A.H. (2014) (EMPA-REG RENAL)[139]	patients with type 2 diabetes mellitus and kidney disease	Empagliflozin 10mg/day<br>Empagliflozin 25mg/day<br>Placebo	98<br>97<br>95	63.2±8.5<br>62.0±8.4<br>62.6±8.1	38.8<br>37.1<br>41.1	24 weeks	SGLT2 inhibitor	NCT01164501	Multiple countries
Ridderstrale, M. (2014) (EMPA-REG H2H-SU)[140]	patients with type 2 diabetes mellitus and moderate-to-severe chronic kidney disease	Empagliflozin 25mg/day<br>Control with glimepiride	765<br>780	56.2±10.3<br>55.7±10.4	43.5<br>46.0	104 weeks	SGLT2 inhibitor	NCT01167881	Multiple countries
Weissman, P.N. (2014) (HARMONY 4)[141]	patients with type 2 diabetes mellitus	Albiglutide 30 mg<br>Control with insulin glargine	504<br>241	55.8±9.3<br>54.7±9.8	43.3<br>45.2	52 weeks	GLP-1 agonist	NCT00838916	Multiple countries
Roden, M. (2013) (EMPA-REG MONO)[142]	patients with type 2 diabetes mellitus	Empagliflozin 10mg/day<br>Empagliflozin 25mg/day<br>Placebo	224<br>224<br>228	56.2±11.6<br>53.8±11.6<br>54.9±10.9	36.6<br>35.3<br>46.1	24 weeks	SGLT2 inhibitor	NCT01177813	Multiple countries
Rosenstock, J. (2013) (GetGoal-X)[143]	patients with type 2 diabetes mellitus	Lixisenatide 20ug<br>Exenatide	318<br>316	57.3±9.2<br>57.6±10.7	52.5<br>40.8	24 weeks	GLP-1 agonist	NCT00707031	Multiple countries
Gallwitz, B. (2012) (EUREXA)[144]	patients with type 2 diabetes mellitus	Exenatide<br>Control with glimepiride	490<br>487	56.0±10.0<br>56.0±9.1	44.5<br>48.3	104 weeks	GLP-1 agonist	NCT00359762	Multiple countries

Abbreviations: GLP-1 agonist: glucagon-like peptide-1 agonist; NA: not available; SGLT2 inhibitor: sodium–glucose cotransporter 2 inhibitor

Table S5A: League table of the primary outcome: subgroup analysis of head, eyes, ears, nose, and throat origin

Empagliflozin	.	.	.	0.26 [0.03; 2.32]	.
0.51 [0.02; 13.34]	Liraglutide	.	.	0.50 [0.05; 5.52]	.
0.51 [0.02; 13.30]	1.00 [0.03; 29.73]	Exenatide	.	0.50 [0.05; 5.54]	.
0.26 [0.01; 8.86]	0.50 [0.01; 19.59]	0.50 [0.01; 19.65]	Dapagliflozin	1.00 [0.06; 15.98]	.
0.26 [0.03; 2.32]	0.50 [0.05; 5.52]	0.50 [0.05; 5.54]	1.00 [0.06; 15.98]	Placebo_or_Control	0.50 [0.05; 5.52]
0.13 [0.00; 3.34]	0.25 [0.01; 7.46]	0.25 [0.01; 7.48]	0.50 [0.01; 19.56]	0.50 [0.05; 5.52]	Inject_semaglutide

Data present as OR [95%CIs]. Pairwise (upper-right portion) and network (lower-left portion) meta-analysis results are presented as estimate effect sizes for the outcome of events of head, eyes, ears, nose, and throat origin metastatic cancers. Interventions are reported in order of mean ranking of beneficially prophylactic effect on events of head, eyes, ears, nose, and throat origin metastatic cancers, and outcomes are expressed as odds ratio (OR) (95% confidence intervals) (95%CIs). For the pairwise meta-analyses, OR of less than 1 indicate that the treatment specified in the row got more beneficial effect than that specified in the column. For the network meta-analysis (NMA), OR of less than 1 indicate that the treatment specified in the column got more beneficial effect than that specified in the row. Bold results marked with * indicate statistical significance.

Table S5B: League table of the primary outcome: subgroup analysis of digestive organ origin

Inject_ semaglutide	.	.	.	.	.	.	.	0.52 [0.23; 1.19]	.	.	.	.	.	.	.
1.33 [0.14; 12.63]	Tirzepatide	.	.	.	.	.	.	0.39 [0.05; 3.20]	.	.	.	.	.	.	.
1.18 [0.16; 8.67]	0.89 [0.06; 14.21]	Efpeglenatide	.	.	.	.	.	0.24 [0.03; 2.14]	1.50 [0.06; 36.96]	.	.	.	.	.	.
0.73 [0.20; 2.68]	0.55 [0.05; 5.63]	0.62 [0.08; 4.97]	Albiglutide	.	.	.	.	0.71 [0.26; 1.95]	.	.	.	.	.	.	.
0.74 [0.09; 5.98]	0.56 [0.03; 9.57]	0.63 [0.04; 8.87]	1.01 [0.12; 8.82]	Oral_ semaglutide	.	.	.	0.71 [0.10; 4.83]	.	.	.	.	.	.	.
0.65 [0.18; 2.36]	0.49 [0.05; 4.99]	0.56 [0.07; 4.40]	0.89 [0.22; 3.64]	0.88 [0.10; 7.65]	Ertugliflozin	.	.	0.80 [0.30; 2.14]	.	.	.	.	.	.	.
0.61 [0.22; 1.68]	0.46 [0.05; 4.05]	0.52 [0.08; 3.51]	0.83 [0.26; 2.67]	0.82 [0.11; 6.14]	0.93 [0.29; 2.94]	Dapagliflozin	.	0.86 [0.47; 1.56]	.	.	.	.	.	.	.
0.59 [0.15; 2.28]	0.45 [0.04; 4.68]	0.50 [0.06; 4.15]	0.81 [0.19; 3.50]	0.80 [0.09; 7.21]	0.90 [0.21; 3.87]	0.97 [0.29; 3.31]	Exenatide	0.88 [0.30; 2.57]	.	.	.	.	.	.	.
0.52 [0.23; 1.19]	0.39 [0.05; 3.20]	0.44 [0.07; 2.74]	0.71 [0.26; 1.95]	0.71 [0.10; 4.83]	0.80 [0.30; 2.14]	0.86 [0.47; 1.56]	0.88 [0.30; 2.57]	Placebo_ or_Control	0.83 [0.36; 1.93]	0.85 [0.34; 2.14]	0.66 [0.03; 16.34]	0.73 [0.26; 2.07]	0.50 [0.05; 5.51]	0.37 [0.08; 1.61]	0.07 [0.00; 1.17]
0.48 [0.15; 1.52]	0.36 [0.04; 3.41]	0.40 [0.06; 2.67]	0.65 [0.18; 2.38]	0.64 [0.08; 5.20]	0.73 [0.20; 2.62]	0.78 [0.28; 2.16]	0.80 [0.21; 3.09]	0.91 [0.40; 2.07]	Dulaglutide	.	.	.	.	.	.
0.45 [0.13; 1.53]	0.34 [0.03; 3.32]	0.38 [0.05; 2.92]	0.61 [0.16; 2.38]	0.60 [0.07; 5.08]	0.68 [0.18; 2.62]	0.73 [0.25; 2.20]	0.75 [0.18; 3.09]	0.85 [0.34; 2.14]	0.94 [0.27; 3.22]	Empagliflozin	.	.	.	.	.
0.35 [0.01; 9.49]	0.26 [0.01; 12.02]	0.30 [0.01; 11.76]	0.47 [0.02; 13.60]	0.47 [0.01; 19.67]	0.53 [0.02; 15.15]	0.57 [0.02; 14.85]	0.59 [0.02; 17.17]	0.66 [0.03; 16.34]	0.73 [0.03; 19.91]	0.78 [0.03; 21.79]	Bexagliflozin	.	.	.	.
0.38 [0.10; 1.44]	0.29 [0.03; 3.00]	0.33 [0.04; 2.65]	0.52 [0.12; 2.22]	0.52 [0.06; 4.61]	0.59 [0.14; 2.45]	0.63 [0.19; 2.09]	0.65 [0.15; 2.88]	0.73 [0.26; 2.07]	0.81 [0.21; 3.03]	0.86 [0.22; 3.44]	1.11 [0.04; 32.00]	Liraglutide	.	.	.
0.26 [0.02; 3.31]	0.20 [0.01; 4.77]	0.22 [0.01; 4.52]	0.36 [0.03; 4.81]	0.35 [0.02; 7.65]	0.40 [0.03; 5.35]	0.43 [0.04; 5.10]	0.44 [0.03; 6.11]	0.50 [0.05; 5.51]	0.55 [0.04; 6.94]	0.59 [0.04; 7.66]	0.75 [0.01; 41.14]	0.68 [0.05; 9.30]	Lixisenatide	.	.
0.19 [0.04; 1.04]	0.15 [0.01; 1.89]	0.16 [0.02; 1.71]	0.26 [0.04; 1.57]	0.26 [0.02; 2.94]	0.30 [0.05; 1.74]	0.32 [0.07; 1.56]	0.33 [0.05; 2.01]	0.37 [0.08; 1.61]	0.41 [0.08; 2.19]	0.43 [0.08; 2.46]	0.56 [0.02; 18.88]	0.50 [0.08; 3.05]	0.74 [0.04; 12.37]	Canagliflozin	.
***0.03 [0.00; 0.69]**	***0.03 [0.00; 0.91]**	***0.03 [0.00; 0.88]**	***0.05 [0.00; 0.99]**	0.05 [0.00; 1.48]	0.05 [0.00; 1.10]	0.06 [0.00; 1.07]	0.06 [0.00; 1.25]	0.07 [0.00; 1.17]	0.07 [0.00; 1.44]	0.08 [0.00; 1.58]	0.10 [0.00; 7.36]	0.09 [0.00; 1.91]	0.13 [0.00; 5.59]	0.18 [0.01; 4.51]	Sotagliflozin

Data present as OR [95%CIs]. Pairwise (upper-right portion) and network (lower-left portion) meta-analysis results are presented as estimate effect sizes for the outcome of events of digestive organ origin metastatic cancers. Interventions are reported in order of mean ranking of beneficially prophylactic effect on events of digestive organ origin metastatic cancers, and outcomes are expressed as odds ratio (OR) (95% confidence intervals) (95%CIs). For the pairwise meta-analyses, OR of less than 1 indicate that the treatment specified in the row got more beneficial effect than that specified in the column. For the network meta-analysis (NMA), OR of less than 1 indicate that the treatment specified in the column got more beneficial effect than that specified in the row. Bold results marked with * indicate statistical significance.

Table S5C: League table of the primary outcome: subgroup analysis of respiratory and intrathoracic organ origin

Inject_ semaglutide	.	.	.	.	.	.	.	.	0.44 [0.16; 1.15]	.	.	.	.
0.87 [0.20; 3.87]	Ertugliflozin	.	.	.	.	.	.	.	0.50 [0.16; 1.55]	.	.	.	.
0.57 [0.18; 1.79]	0.66 [0.18; 2.36]	Dapagliflozin	.	.	.	.	.	.	0.76 [0.42; 1.37]	.	.	.	.
0.58 [0.11; 2.89]	0.66 [0.12; 3.67]	1.00 [0.24; 4.14]	Empagliflozin	.	.	.	.	.	0.76 [0.21; 2.74]	.	.	.	.
0.43 [0.02; 8.20]	0.50 [0.02; 9.97]	0.76 [0.04; 12.89]	0.76 [0.04; 16.07]	Lixisenatide	.	.	.	.	1.00 [0.06; 16.00]	.	.	.	.
0.44 [0.03; 5.82]	0.50 [0.04; 7.12]	0.76 [0.06; 9.01]	0.76 [0.05; 11.57]	1.00 [0.03; 39.29]	Efpeglenatide	.	.	.	1.00 [0.09; 11.01]	.	.	.	.
0.43 [0.01; 12.17]	0.49 [0.02; 14.69]	0.74 [0.03; 19.38]	0.74 [0.02; 23.50]	0.98 [0.01; 68.05]	0.98 [0.02; 53.74]	Sotagliflozin	.	.	1.02 [0.04; 25.15]	.	.	.	.
0.43 [0.09; 2.10]	0.50 [0.09; 2.67]	0.76 [0.19; 2.99]	0.75 [0.13; 4.52]	1.00 [0.05; 20.82]	1.00 [0.07; 14.86]	1.02 [0.03; 31.66]	Dulaglutide	.	1.00 [0.29; 3.46]	.	.	.	.
0.43 [0.11; 1.73]	0.50 [0.11; 2.23]	0.76 [0.24; 2.38]	0.76 [0.15; 3.82]	1.00 [0.05; 18.92]	1.00 [0.07; 13.33]	1.02 [0.04; 29.10]	1.00 [0.21; 4.86]	Liraglutide	1.00 [0.38; 2.67]	.	.	.	.
0.44 [0.16; 1.15]	0.50 [0.16; 1.55]	0.76 [0.42; 1.37]	0.76 [0.21; 2.74]	1.00 [0.06; 16.00]	1.00 [0.09; 11.01]	1.02 [0.04; 25.15]	1.00 [0.29; 3.46]	1.00 [0.38; 2.67]	Placebo_ or_Control	0.93 [0.29; 2.97]	0.78 [0.24; 2.56]	0.72 [0.29; 1.80]	0.26 [0.03; 2.33]
0.40 [0.09; 1.84]	0.46 [0.09; 2.35]	0.70 [0.19; 2.59]	0.70 [0.12; 3.98]	0.93 [0.05; 18.75]	0.92 [0.06; 13.33]	0.94 [0.03; 28.61]	0.93 [0.17; 5.09]	0.93 [0.20; 4.25]	0.93 [0.29; 2.97]	Albiglutide	.	.	.
0.34 [0.07; 1.57]	0.39 [0.08; 2.01]	0.59 [0.16; 2.23]	0.59 [0.10; 3.41]	0.78 [0.04; 15.98]	0.78 [0.05; 11.37]	0.80 [0.03; 24.36]	0.79 [0.14; 4.36]	0.78 [0.17; 3.65]	0.78 [0.24; 2.56]	0.85 [0.16; 4.45]	Canagliflozin	.	.
0.32 [0.08; 1.19]	0.36 [0.08; 1.55]	0.55 [0.19; 1.62]	0.55 [0.11; 2.65]	0.72 [0.04; 13.41]	0.72 [0.06; 9.42]	0.74 [0.03; 20.68]	0.73 [0.16; 3.38]	0.72 [0.19; 2.76]	0.72 [0.29; 1.80]	0.78 [0.18; 3.43]	0.92 [0.21; 4.11]	Exenatide	.
0.11 [0.01; 1.25]	0.13 [0.01; 1.53]	0.19 [0.02; 1.91]	0.19 [0.02; 2.50]	0.26 [0.01; 8.89]	0.26 [0.01; 6.68]	0.26 [0.01; 12.83]	0.26 [0.02; 3.23]	0.26 [0.02; 2.88]	0.26 [0.03; 2.33]	0.28 [0.02; 3.36]	0.33 [0.03; 4.01]	0.35 [0.03; 3.86]	Oral_ semaglutide

Data present as OR [95%CIs]. Pairwise (upper-right portion) and network (lower-left portion) meta-analysis results are presented as estimate effect sizes for the outcome of events of respiratory and intrathoracic organ origin metastatic cancers. Interventions are reported in order of mean ranking of beneficially prophylactic effect on events of respiratory and intrathoracic organ origin metastatic cancers, and outcomes are expressed as odds ratio (OR) (95% confidence intervals) (95%CIs). For the pairwise meta-analyses, OR of less than 1 indicate that the treatment

specified in the row got more beneficial effect than that specified in the column. For the network meta-analysis (NMA), OR of less than 1 indicate that the treatment specified in the column got more beneficial effect than that specified in the row. Bold results marked with * indicate statistical significance.

Table S5D: League table of the primary outcome: subgroup analysis of bone origin

Dulaglutide	0.33 [0.01; 8.19]	.	.	.
0.33 [0.01; 8.19]	Placebo_or_Control	0.33 [0.01; 8.19]	0.33 [0.03; 3.20]	0.14 [0.01; 2.77]
0.11 [0.00; 10.29]	0.33 [0.01; 8.19]	Albiglutide	.	.
0.11 [0.00; 5.60]	0.33 [0.03; 3.20]	1.00 [0.02; 50.38]	Inject_semaglutide	.
0.05 [0.00; 3.74]	0.14 [0.01; 2.77]	0.43 [0.01; 33.61]	0.43 [0.01; 17.86]	Dapagliflozin

Data present as OR [95%CIs]. Pairwise (upper-right portion) and network (lower-left portion) meta-analysis results are presented as estimate effect sizes for the outcome of events of bone origin metastatic cancers. Interventions are reported in order of mean ranking of beneficially prophylactic effect on events of bone origin metastatic cancers, and outcomes are expressed as odds ratio (OR) (95% confidence intervals) (95%CIs). For the pairwise meta-analyses, OR of less than 1 indicate that the treatment specified in the row got more beneficial effect than that specified in the column. For the network meta-analysis (NMA), OR of less than 1 indicate that the treatment specified in the column got more beneficial effect than that specified in the row. Bold results marked with * indicate statistical significance.

Table S5E: League table of the primary outcome: subgroup analysis of skin, mesothelium, soft tissue, and cartilage origin

Sotagliflozin	.	.	.	.	.	0.24 [0.02; 2.27]	.	.	.	.
0.70 [0.04; 11.55]	Empagliflozin	.	.	.	.	0.33 [0.06; 1.74]	.	.	.	.
0.39 [0.03; 5.71]	0.56 [0.06; 4.95]	Liraglutide	.	.	.	0.60 [0.14; 2.51]	.	.	.	.
0.26 [0.02; 3.04]	0.37 [0.06; 2.48]	0.67 [0.12; 3.69]	Dapagliflozin	.	.	0.89 [0.35; 2.26]	.	.	.	.
0.24 [0.01; 6.38]	0.34 [0.02; 6.16]	0.60 [0.04; 9.83]	0.89 [0.07; 11.73]	Ertugliflozin	.	1.00 [0.09; 11.03]	.	.	.	.
0.22 [0.01; 6.07]	0.32 [0.02; 5.86]	0.57 [0.04; 9.35]	0.86 [0.07; 11.15]	0.96 [0.03; 28.38]	Dulaglutide	3.00 [0.12; 73.76]	0.33 [0.01; 8.23]	.	.	.
0.24 [0.02; 2.27]	0.33 [0.06; 1.74]	0.60 [0.14; 2.51]	0.89 [0.35; 2.26]	1.00 [0.09; 11.03]	1.05 [0.10; 11.47]	Placebo_ or_Control	1.50 [0.25; 8.98]	0.33 [0.01; 8.14]	0.40 [0.02; 8.34]	0.33 [0.01; 8.18]
0.22 [0.01; 3.39]	0.31 [0.03; 2.98]	0.55 [0.07; 4.60]	0.82 [0.13; 5.06]	0.92 [0.05; 16.12]	0.96 [0.09; 10.55]	0.92 [0.19; 4.39]	Inject_ semaglutide	1.00 [0.04; 24.59]	.	.
0.13 [0.00; 3.51]	0.18 [0.01; 3.38]	0.33 [0.02; 5.40]	0.49 [0.04; 6.44]	0.55 [0.02; 16.39]	0.58 [0.02; 14.19]	0.55 [0.05; 6.06]	0.60 [0.05; 6.58]	Tirzepatide	.	.
0.09 [0.00; 4.16]	0.13 [0.00; 4.24]	0.24 [0.01; 6.90]	0.36 [0.01; 8.57]	0.40 [0.01; 19.20]	0.42 [0.01; 20.02]	0.40 [0.02; 8.34]	0.44 [0.01; 13.24]	0.72 [0.02; 34.68]	Canagliflozin	.
0.08 [0.00; 3.95]	0.11 [0.00; 4.08]	0.20 [0.01; 6.67]	0.30 [0.01; 8.35]	0.33 [0.01; 18.21]	0.35 [0.01; 18.98]	0.33 [0.01; 8.18]	0.36 [0.01; 12.76]	0.60 [0.01; 32.89]	0.83 [0.01; 68.67]	Oral_ semaglutide

Data present as OR [95%CIs]. Pairwise (upper-right portion) and network (lower-left portion) meta-analysis results are presented as estimate effect sizes for the outcome of events of skin, mesothelium, soft tissue, and cartilage origin metastatic cancers. Interventions are reported in order of mean ranking of beneficially prophylactic effect on events of skin, mesothelium, soft tissue, and cartilage origin metastatic cancers, and outcomes are expressed as odds ratio (OR) (95% confidence intervals) (95%CIs). For the pairwise meta-analyses, OR of less than 1 indicate that the treatment specified in the row got more beneficial effect than that specified in the column. For the network meta-analysis (NMA), OR of less than 1 indicate that the treatment specified in the column got more beneficial effect than that specified in the row. Bold results marked with * indicate statistical significance.

Table S5F: League table of the primary outcome: subgroup analysis of breast and female genital organ origin

Sotagliflozin	.	.	.	.	.	.	.	0.23 [0.02; 2.25]	.	.	.	.
0.92 [0.04; 21.64]	Exenatide	.	.	.	.	.	.	0.25 [0.03; 2.30]	.	.	.	.
0.70 [0.03; 17.23]	0.76 [0.03; 17.98]	Albiglutide	.	.	.	.	.	0.33 [0.03; 3.20]	.	.	.	.
0.50 [0.02; 11.02]	0.55 [0.03; 11.48]	0.72 [0.03; 15.71]	Canagliflozin	.	.	.	.	0.46 [0.06; 3.77]	.	.	.	.
0.45 [0.03; 6.61]	0.49 [0.03; 6.84]	0.64 [0.04; 9.42]	0.89 [0.07; 11.41]	Dulaglutide	.	.	.	0.52 [0.12; 2.25]	.	.	.	.
0.47 [0.01; 16.76]	0.51 [0.01; 17.56]	0.67 [0.02; 23.89]	0.93 [0.03; 29.97]	1.05 [0.05; 24.00]	Ertugliflozin	.	.	0.50 [0.03; 7.99]	.	.	.	.
0.33 [0.03; 3.91]	0.36 [0.03; 4.03]	0.47 [0.04; 5.57]	0.66 [0.07; 6.65]	0.74 [0.13; 4.30]	0.71 [0.04; 13.43]	Dapagliflozin	.	0.70 [0.27; 1.86]	.	.	.	.
0.29 [0.01; 7.24]	0.31 [0.01; 7.55]	0.41 [0.02; 10.32]	0.57 [0.03; 12.79]	0.64 [0.04; 9.79]	0.61 [0.02; 22.52]	0.86 [0.07; 10.50]	Oral_ semaglutide	1.67 [0.08; 34.90]	0.33 [0.01; 8.16]	.	.	.
0.23 [0.02; 2.25]	0.25 [0.03; 2.30]	0.33 [0.03; 3.20]	0.46 [0.06; 3.77]	0.52 [0.12; 2.25]	0.50 [0.03; 7.99]	0.70 [0.27; 1.86]	0.81 [0.08; 8.12]	Placebo_ or_Control	1.41 [0.28; 7.01]	0.81 [0.08; 7.83]	0.63 [0.13; 2.98]	0.20 [0.01; 4.16]
0.21 [0.01; 3.01]	0.23 [0.02; 3.11]	0.30 [0.02; 4.29]	0.42 [0.03; 5.19]	0.47 [0.06; 3.55]	0.45 [0.02; 10.05]	0.64 [0.12; 3.47]	0.74 [0.07; 7.53]	0.91 [0.23; 3.63]	Liraglutide	.	2.31 [0.09; 57.10]	.
0.19 [0.01; 4.67]	0.21 [0.01; 4.87]	0.27 [0.01; 6.65]	0.38 [0.02; 8.24]	0.42 [0.03; 6.29]	0.41 [0.01; 14.56]	0.57 [0.05; 6.72]	0.66 [0.03; 16.69]	0.81 [0.08; 7.83]	0.90 [0.06; 12.76]	Efpeglenatide	.	.
0.18 [0.01; 2.68]	0.20 [0.01; 2.77]	0.26 [0.02; 3.81]	0.37 [0.03; 4.61]	0.41 [0.05; 3.17]	0.39 [0.02; 8.90]	0.55 [0.10; 3.12]	0.64 [0.05; 9.01]	0.79 [0.19; 3.29]	0.87 [0.14; 5.24]	0.97 [0.07; 14.14]	Inject_ semaglutide	.
0.05 [0.00; 2.06]	0.05 [0.00; 2.16]	0.07 [0.00; 2.94]	0.09 [0.00; 3.71]	0.10 [0.00; 3.04]	0.10 [0.00; 6.10]	0.14 [0.01; 3.41]	0.16 [0.00; 7.34]	0.20 [0.01; 4.16]	0.22 [0.01; 6.21]	0.25 [0.01; 10.88]	0.25 [0.01; 7.27]	Empagliflozin

Data present as OR [95%CIs]. Pairwise (upper-right portion) and network (lower-left portion) meta-analysis results are presented as estimate effect sizes for the outcome of events of breast and female genital organ origin metastatic cancers. Interventions are reported in order of mean ranking of beneficially prophylactic effect on events of breast and female genital organ origin metastatic cancers, and outcomes are expressed as odds ratio (OR) (95% confidence intervals) (95%CIs). For the pairwise meta-analyses, OR of less than 1 indicate that the treatment specified in the row got more beneficial effect than that specified in the column. For the network meta-analysis (NMA), OR of less than 1 indicate that the treatment specified in the column got more beneficial effect than that specified in the row. Bold results marked with * indicate statistical significance.

Table S5G: League table of the primary outcome: subgroup analysis of prostate and male genital organ origin

Efpeglenatide	.	.	.	.	.	.	.	0.13 [0.01; 1.30]	.	.	.	.	.
0.27 [0.02; 4.58]	Liraglutide	.	.	.	.	.	.	0.50 [0.09; 2.73]	.	.	.	.	.
0.27 [0.02; 4.58]	1.00 [0.09; 11.03]	Dulaglutide	.	.	.	.	.	0.50 [0.09; 2.73]	.	.	.	.	.
0.16 [0.01; 1.87]	0.59 [0.08; 4.17]	0.59 [0.08; 4.17]	Inject_ semaglutide	.	.	.	.	0.86 [0.31; 2.35]	0.33 [0.01; 8.23]	.	.	.	.
0.15 [0.01; 2.23]	0.57 [0.06; 5.20]	0.57 [0.06; 5.21]	0.97 [0.17; 5.40]	Empagliflozin	.	.	.	0.88 [0.21; 3.62]	.	.	.	.	.
0.15 [0.01; 1.72]	0.56 [0.08; 3.80]	0.56 [0.08; 3.80]	0.94 [0.25; 3.56]	0.97 [0.18; 5.23]	Dapagliflozin	.	.	0.90 [0.37; 2.22]	.	.	.	.	.
0.13 [0.00; 4.84]	0.50 [0.02; 12.91]	0.50 [0.02; 12.91]	0.85 [0.04; 16.04]	0.88 [0.04; 19.72]	0.90 [0.05; 16.60]	Lixisenatide	.	1.00 [0.06; 16.00]	.	.	.	.	.
0.13 [0.01; 2.51]	0.50 [0.04; 6.14]	0.50 [0.04; 6.14]	0.85 [0.10; 6.84]	0.87 [0.08; 8.98]	0.90 [0.11; 7.02]	1.00 [0.04; 27.90]	Albiglutide	1.01 [0.16; 6.40]	.	.	.	.	.
0.13 [0.01; 1.30]	0.50 [0.09; 2.73]	0.50 [0.09; 2.73]	0.85 [0.32; 2.25]	0.88 [0.21; 3.62]	0.90 [0.37; 2.22]	1.00 [0.06; 16.00]	1.01 [0.16; 6.40]	Placebo_ or_Control	0.47 [0.05; 4.54]	0.33 [0.01; 8.19]	0.33 [0.01; 8.18]	0.33 [0.01; 8.15]	0.33 [0.04; 2.77]
0.06 [0.00; 1.14]	0.22 [0.02; 2.79]	0.22 [0.02; 2.79]	0.38 [0.05; 2.68]	0.39 [0.04; 4.09]	0.40 [0.05; 3.20]	0.44 [0.02; 12.63]	0.45 [0.03; 6.23]	0.44 [0.07; 2.90]	Canagliflozin	.	.	.	.
0.05 [0.00; 2.27]	0.17 [0.00; 6.25]	0.17 [0.00; 6.25]	0.28 [0.01; 8.04]	0.29 [0.01; 9.69]	0.30 [0.01; 8.35]	0.33 [0.00; 23.03]	0.34 [0.01; 13.53]	0.33 [0.01; 8.19]	0.75 [0.02; 30.71]	Sotagliflozin	.	.	.
0.04 [0.00; 2.27]	0.17 [0.00; 6.24]	0.17 [0.00; 6.25]	0.28 [0.01; 8.04]	0.29 [0.01; 9.68]	0.30 [0.01; 8.34]	0.33 [0.00; 23.01]	0.33 [0.01; 13.51]	0.33 [0.01; 8.18]	0.75 [0.02; 30.68]	1.00 [0.01; 92.36]	Oral_ semaglutide	.	.
0.04 [0.00; 2.26]	0.17 [0.00; 6.22]	0.17 [0.00; 6.22]	0.28 [0.01; 8.00]	0.29 [0.01; 9.65]	0.30 [0.01; 8.31]	0.33 [0.00; 22.92]	0.33 [0.01; 13.46]	0.33 [0.01; 8.15]	0.75 [0.02; 30.57]	1.00 [0.01; 92.01]	1.00 [0.01; 92.17]	Exenatide	.
0.04 [0.00; 1.00]	0.17 [0.01; 2.52]	0.17 [0.01; 2.52]	0.28 [0.03; 2.91]	0.29 [0.02; 3.74]	0.30 [0.03; 2.99]	0.33 [0.01; 10.91]	0.33 [0.02; 5.58]	0.33 [0.04; 2.77]	0.75 [0.04; 12.71]	1.00 [0.02; 46.38]	1.00 [0.02; 46.47]	1.00 [0.02; 46.60]	Ertugliflozin

Data present as OR [95%CIs]. Pairwise (upper-right portion) and network (lower-left portion) meta-analysis results are presented as estimate effect sizes for the outcome of events of prostate and male genital organ origin metastatic cancers. Interventions are reported in order of mean ranking of beneficially prophylactic effect on events of prostate and male genital organ origin metastatic cancers, and outcomes are expressed as odds ratio (OR) (95% confidence intervals) (95%CIs). For the pairwise meta-analyses, OR of less than 1 indicate that the treatment specified in the row got more beneficial effect than that specified in the column. For the network meta-analysis (NMA), OR of less than 1 indicate that the treatment specified in the column got more beneficial effect than that specified in the row. Bold results marked with * indicate statistical significance.

Table S5H: League table of the primary outcome: subgroup analysis of kidney and urinary tract origin

Exenatide	.	.	.	0.14 [0.01; 2.78]	.	.	.	.	.	.	.	.
0.43 [0.01; 17.89]	Dulaglutide	.	.	0.33 [0.03; 3.21]	.	.	.	.	.	.	.	.
0.43 [0.01; 33.76]	1.00 [0.02; 50.51]	Sotagliflozin	.	0.33 [0.01; 8.18]	.	.	.	.	.	.	.	.
0.29 [0.00; 16.59]	0.67 [0.02; 23.91]	0.67 [0.01; 45.97]	Bexagliflozin	0.50 [0.03; 8.02]	.	.	.	.	.	.	.	.
0.14 [0.01; 2.78]	0.33 [0.03; 3.21]	0.33 [0.01; 8.18]	0.50 [0.03; 8.02]	Placebo_ or_Control	1.00 [0.16; 6.15]	0.65 [0.03; 16.14]	0.61 [0.19; 1.98]	0.59 [0.12; 2.79]	0.33 [0.01; 8.19]	0.33 [0.01; 8.18]	0.33 [0.03; 3.20]	0.21 [0.04; 1.20]
0.14 [0.00; 4.64]	0.33 [0.02; 6.08]	0.33 [0.01; 13.21]	0.50 [0.02; 13.79]	1.00 [0.16; 6.15]	Canagliflozin	.	.	.	.	.	.	.
0.09 [0.00; 7.38]	0.22 [0.00; 11.05]	0.22 [0.00; 20.21]	0.33 [0.00; 22.71]	0.65 [0.03; 16.14]	0.65 [0.02; 26.01]	Ertugliflozin	.	.	.	.	.	.
0.09 [0.00; 2.12]	0.20 [0.02; 2.61]	0.20 [0.01; 6.14]	0.30 [0.01; 6.20]	0.61 [0.19; 1.98]	0.61 [0.07; 5.30]	0.93 [0.03; 28.26]	Dapagliflozin	.	.	.	.	.
0.08 [0.00; 2.39]	0.20 [0.01; 3.06]	0.20 [0.01; 6.87]	0.29 [0.01; 7.08]	0.59 [0.12; 2.79]	0.59 [0.05; 6.41]	0.90 [0.03; 31.65]	0.97 [0.14; 6.83]	Empagliflozin	.	.	.	.
0.05 [0.00; 3.75]	0.11 [0.00; 5.61]	0.11 [0.00; 10.26]	0.17 [0.00; 11.53]	0.33 [0.01; 8.19]	0.33 [0.01; 13.20]	0.51 [0.01; 47.21]	0.55 [0.02; 16.64]	0.57 [0.02; 19.96]	Albiglutide	.	.	.
0.05 [0.00; 3.74]	0.11 [0.00; 5.60]	0.11 [0.00; 10.25]	0.17 [0.00; 11.52]	0.33 [0.01; 8.18]	0.33 [0.01; 13.19]	0.51 [0.01; 47.18]	0.55 [0.02; 16.63]	0.57 [0.02; 19.94]	1.00 [0.01; 92.38]	Lixisenatide	.	.
0.05 [0.00; 1.99]	0.11 [0.00; 2.73]	0.11 [0.00; 5.59]	0.17 [0.00; 5.98]	0.33 [0.03; 3.20]	0.33 [0.02; 6.05]	0.51 [0.01; 25.72]	0.55 [0.04; 7.04]	0.57 [0.04; 8.85]	1.00 [0.02; 50.34]	1.00 [0.02; 50.39]	Liraglutide	.
***0.03 [0.00; 0.93]**	0.07 [0.00; 1.21]	0.07 [0.00; 2.65]	0.10 [0.00; 2.76]	0.21 [0.04; 1.20]	0.21 [0.02; 2.58]	0.31 [0.01; 12.20]	0.34 [0.04; 2.83]	0.35 [0.03; 3.69]	0.62 [0.02; 23.87]	0.62 [0.02; 23.89]	0.62 [0.03; 10.91]	Inject_ semaglutide

Data present as OR [95%CIs]. Pairwise (upper-right portion) and network (lower-left portion) meta-analysis results are presented as estimate effect sizes for the outcome of events of kidney and urinary tract origin metastatic cancers. Interventions are reported in order of mean ranking of beneficially prophylactic effect on events of kidney and urinary tract origin metastatic cancers, and outcomes are expressed as odds ratio (OR) (95% confidence intervals) (95%CIs). For the pairwise meta-analyses, OR of less than 1 indicate that the treatment specified in the row got more beneficial effect than that specified in the column. For the network meta-analysis (NMA), OR of less than 1 indicate that the treatment specified in the column got more beneficial effect than that specified in the row. Bold results marked with * indicate statistical significance.

Table S5I: League table of the primary outcome: subgroup analysis of neuron, nerve, and neuroendocrine origin

Efpeglenatide	.	0.17 [0.01; 4.08]	.	.	.	.	.	.	.
0.49 [0.01; 20.68]	Inject_semaglutide	0.34 [0.05; 2.29]	.	.	.	.	.	.	.
0.17 [0.01; 4.08]	0.34 [0.05; 2.29]	Placebo_or_Control	1.00 [0.06; 16.03]	0.67 [0.03; 16.37]	0.64 [0.11; 3.68]	0.33 [0.01; 8.19]	0.33 [0.01; 8.19]	0.33 [0.01; 8.19]	0.33 [0.01; 8.18]
0.17 [0.00; 11.49]	0.34 [0.01; 9.82]	1.00 [0.06; 16.03]	Empagliflozin	.	.	.	.	.	.
0.11 [0.00; 10.24]	0.22 [0.01; 9.36]	0.67 [0.03; 16.37]	0.67 [0.01; 45.93]	Ertugliflozin	.	.	.	.	.
0.11 [0.00; 4.07]	0.21 [0.02; 2.88]	0.64 [0.11; 3.68]	0.64 [0.02; 16.90]	0.96 [0.02; 36.76]	Canagliflozin	.	.	.	.
0.06 [0.00; 5.12]	0.11 [0.00; 4.68]	0.33 [0.01; 8.19]	0.33 [0.00; 22.97]	0.50 [0.01; 46.25]	0.52 [0.01; 20.13]	Sotagliflozin	.	.	.
0.06 [0.00; 5.12]	0.11 [0.00; 4.68]	0.33 [0.01; 8.19]	0.33 [0.00; 22.96]	0.50 [0.01; 46.23]	0.52 [0.01; 20.12]	1.00 [0.01; 92.42]	Dapagliflozin	.	.
0.06 [0.00; 5.12]	0.11 [0.00; 4.68]	0.33 [0.01; 8.19]	0.33 [0.00; 22.96]	0.50 [0.01; 46.23]	0.52 [0.01; 20.12]	1.00 [0.01; 92.40]	1.00 [0.01; 92.42]	Albiglutide	.
0.06 [0.00; 5.12]	0.11 [0.00; 4.68]	0.33 [0.01; 8.18]	0.33 [0.00; 22.95]	0.50 [0.01; 46.20]	0.52 [0.01; 20.11]	1.00 [0.01; 92.36]	1.00 [0.01; 92.38]	1.00 [0.01; 92.41]	Oral_semaglutide

Data present as OR [95%CIs]. Pairwise (upper-right portion) and network (lower-left portion) meta-analysis results are presented as estimate effect sizes for the outcome of events of neuron, nerve, and neuroendocrine origin metastatic cancers. Interventions are reported in order of mean ranking of beneficially prophylactic effect on events of neuron, nerve, and neuroendocrine origin metastatic cancers, and outcomes are expressed as odds ratio (OR) (95% confidence intervals) (95%CIs). For the pairwise meta-analyses, OR of less than 1 indicate that the treatment specified in the row got more beneficial effect than that specified in the column. For the network meta-analysis (NMA), OR of less than 1 indicate that the treatment specified in the column got more beneficial effect than that specified in the row. Bold results marked with * indicate statistical significance.

Table S5J: League table of the primary outcome: subgroup analysis of thyroid and other endocrine gland origin

Dapagliflozin	0.33 [0.03; 3.20]	.	.	.	.
0.33 [0.03; 3.20]	Placebo_or_Control	0.33 [0.01; 8.18]	0.33 [0.01; 8.20]	0.33 [0.01; 8.18]	0.66 [0.03; 16.38]
0.20 [0.01; 6.41]	0.61 [0.04; 8.27]	Oral_semaglutide	.	.	0.33 [0.01; 8.16]
0.11 [0.00; 5.61]	0.33 [0.01; 8.20]	0.55 [0.01; 34.44]	Empagliflozin	.	.
0.11 [0.00; 5.59]	0.33 [0.01; 8.18]	0.55 [0.01; 34.34]	1.00 [0.01; 92.20]	Inject_semaglutide	.
0.12 [0.00; 3.86]	0.36 [0.03; 4.99]	0.60 [0.04; 8.24]	1.09 [0.02; 68.15]	1.09 [0.02; 68.39]	Liraglutide

Data present as OR [95%CIs]. Pairwise (upper-right portion) and network (lower-left portion) meta-analysis results are presented as estimate effect sizes for the outcome of events of thyroid and other endocrine gland origin metastatic cancers. Interventions are reported in order of mean ranking of beneficially prophylactic effect on events of thyroid and other endocrine gland origin metastatic cancers, and outcomes are expressed as odds ratio (OR) (95% confidence intervals) (95%CIs). For the pairwise meta-analyses, OR of less than 1 indicate that the treatment specified in the row got more beneficial effect than that specified in the column. For the network meta-analysis (NMA), OR of less than 1 indicate that the treatment specified in the column got more beneficial effect than that specified in the row. Bold results marked with * indicate statistical significance.

Table S5K: League table of NMA of safety profile: drop-out rate

Tirzepatide	.	.	0.89 [0.50; 1.57]	.	.	.	.	.	.	.	.	.	***0.59 [0.45; 0.78]**	.	.
0.92 [0.65; 1.29]	Canagliflozin	.	0.80 [0.41; 1.57]	.	.	.	.	.	.	.	.	.	***0.67 [0.52; 0.86]**	.	.
0.78 [0.54; 1.12]	0.85 [0.59; 1.22]	Ertugliflozin	.	.	.	.	.	.	.	.	.	.	0.79 [0.60; 1.03]	.	.
0.77 [0.58; 1.01]	0.84 [0.63; 1.10]	0.99 [0.72; 1.35]	Inject_ semaglutide	0.92 [0.38; 2.23]	1.51 [0.84; 2.72]	.	.	.	.	.	.	.	***0.78 [0.65; 0.93]**	.	.
0.77 [0.54; 1.09]	0.84 [0.60; 1.18]	0.99 [0.69; 1.43]	1.00 [0.75; 1.34]	Liraglutide	.	.	.	.	.	.	.	.	***0.76 [0.59; 0.98]**	.	1.26 [0.46; 3.44]
0.77 [0.52; 1.13]	0.84 [0.58; 1.23]	0.99 [0.66; 1.49]	1.01 [0.73; 1.39]	1.00 [0.68; 1.48]	Dulaglutide	.	.	.	.	0.84 [0.54; 1.30]	.	.	1.05 [0.66; 1.66]	.	.
***0.73 [0.55; 0.98]**	0.80 [0.61; 1.06]	0.95 [0.70; 1.29]	0.96 [0.77; 1.19]	0.96 [0.72; 1.27]	0.95 [0.68; 1.33]	Empagliflozin	.	.	.	.	.	.	***0.83 [0.72; 0.96]**	.	.
0.73 [0.52; 1.02]	0.79 [0.57; 1.10]	0.94 [0.66; 1.33]	0.95 [0.72; 1.25]	0.94 [0.67; 1.32]	0.94 [0.65; 1.37]	0.99 [0.76; 1.29]	Albiglutide	.	.	.	.	.	0.84 [0.67; 1.06]	.	.
0.71 [0.48; 1.04]	0.77 [0.53; 1.12]	0.91 [0.61; 1.36]	0.92 [0.67; 1.29]	0.92 [0.63; 1.35]	0.92 [0.61; 1.39]	0.96 [0.70; 1.33]	0.98 [0.68; 1.41]	Dapagliflozin	.	.	.	.	0.86 [0.65; 1.15]	.	.
0.70 [0.36; 1.34]	0.76 [0.40; 1.45]	0.90 [0.46; 1.74]	0.91 [0.49; 1.70]	0.91 [0.47; 1.74]	0.91 [0.46; 1.77]	0.95 [0.51; 1.76]	0.96 [0.51; 1.83]	0.99 [0.51; 1.92]	Bexagliflozin	.	.	.	0.88 [0.48; 1.60]	.	.
***0.66 [0.47; 0.94]**	0.73 [0.52; 1.02]	0.86 [0.60; 1.23]	0.87 [0.66; 1.15]	0.86 [0.61; 1.22]	0.86 [0.63; 1.18]	0.91 [0.68; 1.20]	0.92 [0.66; 1.27]	0.94 [0.65; 1.37]	0.95 [0.50; 1.82]	Efpeglenatide	.	.	0.91 [0.70; 1.19]	.	.
***0.66 [0.46; 0.94]**	0.72 [0.50; 1.02]	0.85 [0.58; 1.23]	0.86 [0.63; 1.16]	0.85 [0.60; 1.22]	0.85 [0.57; 1.26]	0.89 [0.67; 1.20]	0.90 [0.64; 1.27]	0.93 [0.63; 1.36]	0.94 [0.49; 1.81]	0.99 [0.69; 1.40]	Sotagliflozin	.	0.93 [0.72; 1.20]	.	.
***0.59 [0.39; 0.90]**	***0.65 [0.43; 0.98]**	0.76 [0.49; 1.18]	0.77 [0.53; 1.12]	0.77 [0.51; 1.17]	0.77 [0.49; 1.20]	0.80 [0.56; 1.16]	0.81 [0.54; 1.22]	0.84 [0.54; 1.30]	0.85 [0.43; 1.69]	0.89 [0.59; 1.34]	0.90 [0.59; 1.38]	Lixisenatide	0.95 [0.62; 1.46]	1.07 [0.67; 1.71]	.
***0.61 [0.48; 0.79]**	***0.67 [0.53; 0.85]**	0.79 [0.60; 1.03]	0.80 [0.68; 0.94]	0.80 [0.62; 1.02]	0.79 [0.59; 1.07]	***0.83 [0.72; 0.96]**	0.84 [0.67; 1.06]	0.86 [0.65; 1.15]	0.88 [0.48; 1.60]	0.92 [0.72; 1.17]	0.93 [0.72; 1.20]	1.03 [0.74; 1.45]	Placebo_ or_Control	0.91 [0.69; 1.20]	0.75 [0.48; 1.18]
***0.57 [0.40; 0.82]**	***0.63 [0.44; 0.89]**	0.74 [0.51; 1.07]	0.75 [0.55; 1.01]	0.75 [0.52; 1.06]	0.74 [0.50; 1.10]	0.78 [0.58; 1.05]	0.79 [0.56; 1.11]	0.81 [0.55; 1.19]	0.82 [0.43; 1.58]	0.86 [0.61; 1.22]	0.87 [0.61; 1.25]	0.97 [0.69; 1.37]	0.94 [0.73; 1.21]	Exenatide	.
***0.48 [0.29; 0.79]**	***0.52 [0.32; 0.85]**	0.61 [0.37; 1.02]	***0.62 [0.39; 0.99]**	0.62 [0.38; 1.00]	0.62 [0.37; 1.05]	0.65 [0.41; 1.02]	0.66 [0.40; 1.07]	0.67 [0.40; 1.13]	0.68 [0.33; 1.43]	0.72 [0.44; 1.17]	0.73 [0.44; 1.20]	0.81 [0.47; 1.39]	0.78 [0.51; 1.20]	0.83 [0.50; 1.37]	Oral_ semaglutide

Data present as OR [95%CIs]. Pairwise (upper-right portion) and network (lower-left portion) meta-analysis results are presented as estimate effect sizes for the outcome of safety profile (drop-out rate). Interventions are reported in order of mean ranking of safety, and outcomes are expressed as odds ratio (OR) (95% confidence intervals) (95%CIs). For the pairwise meta-analyses, OR of less than 1 indicate that the treatment specified in the row got more safety than that specified in the column. For the network meta-analysis (NMA), OR of less than 1 indicate that the treatment specified in the column got more safety than that specified in the row. Bold results marked with * indicate

statistical significance.

Abbreviation: 95%CIs: 95% confidence intervals; GLP-1 agonist: glucagon-like peptide-1 agonist; NMA: network meta-analysis; OR: odds ratio; RCT: randomized controlled trial; SGLT2 inhibitor: sodium–glucose cotransporter 2 inhibitor

Table S6: SUCRA (Surface under the cumulative ranking) of primary outcome: overall events of metastatic cancers

Treatment	Rank 1	Rank 2	Rank 3	Rank 4	Rank 5	Rank 6	Rank 7	Rank 8	Rank 9	Rank 10	Rank 11	Rank 12	Rank 13	Rank 14	Rank 15	Rank 16	SUCRA
Albiglutide	0.00375	0.0246	0.0529375	0.0739625	0.0842	0.0805125	0.0768875	0.0816625	0.0968	0.0945375	0.0904	0.0822625	0.0642875	0.0484375	0.030025	0.0147375	49.32425
Bexagliflozin	0.1290125	0.113175	0.070125	0.0496	0.03625	0.0275	0.0236125	0.023075	0.0272875	0.0295375	0.0322375	0.0380875	0.04725	0.0558125	0.08065	0.2167875	47.42083333
Canagliflozin	0.0002125	0.001175	0.00425	0.0077875	0.0127	0.01715	0.02385	0.0343	0.05235	0.076325	0.1082375	0.14685	0.175125	0.1739	0.11455	0.0512375	26.062
Dapagliflozin	0.0041	0.029075	0.0829	0.137075	0.157625	0.14245	0.115125	0.0954125	0.0779625	0.0622375	0.042375	0.027125	0.0154875	0.0075125	0.0029375	6.00E-04	63.01941667
Dulaglutide	0.0551875	0.19805	0.2269	0.16735	0.10475	0.0677	0.0457875	0.0342875	0.029625	0.0227625	0.0173875	0.0119375	0.0084625	0.0057125	0.0029375	0.0011625	77.28291667
Efpeglenatide	0.6119125	0.1984	0.076525	0.03935	0.0211625	0.012975	0.0091	0.0069625	0.0059625	0.0056375	0.0039	0.00325	0.0022	0.0013875	0.000925	0.00035	93.61208333
Empagliflozin	0.000625	0.0035125	0.0110625	0.0242875	0.039875	0.052275	0.0670625	0.0880375	0.1153625	0.1448875	0.1481375	0.1354625	0.0964	0.0475	0.019625	0.0058875	40.84183333
Ertugliflozin	0.0061375	0.0240625	0.0481125	0.071375	0.06975	0.0664375	0.0623875	0.065325	0.0776875	0.0881875	0.0929	0.088775	0.083225	0.070575	0.05135	0.0337125	45.0755
Exenatide	0.1304625	0.252025	0.2137125	0.137525	0.0878	0.0523	0.0349625	0.0271125	0.0209125	0.0169875	0.01205	0.0071625	0.0038125	0.002275	0.00075	0.00015	81.92108333
Inject_semaglutide	0.0018	0.0173375	0.0507875	0.089225	0.1221875	0.124025	0.11465	0.11465	0.106075	0.0902875	0.073475	0.04945	0.027325	0.0126	0.0049125	0.0012125	56.63691667
Liraglutide	0.0009625	0.0069375	0.0176	0.033525	0.0453375	0.0498875	0.0566875	0.0703875	0.089175	0.11045	0.1260375	0.1126625	0.103925	0.0841625	0.0606	0.0316625	38.14666667
Lixisenatide	0.0055125	0.01985	0.026575	0.0308	0.03335	0.03155	0.0311	0.0329375	0.0410625	0.051325	0.062325	0.078175	0.1041875	0.1364125	0.161575	0.1532625	28.45391667
Oral_semaglutide	0.0030125	0.008875	0.015775	0.0227125	0.02585	0.025775	0.0261	0.0297875	0.0373375	0.0503	0.0653375	0.086575	0.1099875	0.1456	0.1751625	0.1718125	24.63225
Placebo_or_Control	0	0.0002125	0.0039875	0.0240125	0.0832625	0.181075	0.250825	0.23055	0.1440625	0.060975	0.016925	0.0035625	0.00055	0	0	0	57.64158333
Sotagliflozin	0.002875	0.007525	0.01315	0.017175	0.0196125	0.0205625	0.0207625	0.0239375	0.0319875	0.0406375	0.0534	0.070175	0.090575	0.1364875	0.214325	0.2368125	20.66475
Tirzepatide	0.0444375	0.0951875	0.0856	0.0742375	0.0562875	0.047825	0.0411	0.041575	0.04635	0.054925	0.054875	0.0584875	0.0672	0.071625	0.079675	0.0806125	49.264

Table S7A: Side-splitting model inconsistency of primary outcome: overall events of metastatic cancers

Comparison	No.Studies	NMA	Direct	Indirect	Difference	Diff_95CI_lower	Diff_95CI_upper	*p* value
Albiglutide:Bexagliflozin	0	-0.0452	NA	-0.0452	NA	NA	NA	NA
Albiglutide:Canagliflozin	0	-0.4764	NA	-0.4764	NA	NA	NA	NA
Albiglutide:Dapagliflozin	0	0.09816	NA	0.09816	NA	NA	NA	NA
Albiglutide:Dulaglutide	0	0.30228	NA	0.30228	NA	NA	NA	NA
Albiglutide:Efpeglenatide	0	1.3205	NA	1.3205	NA	NA	NA	NA
Albiglutide:Empagliflozin	0	-0.3225	NA	-0.3225	NA	NA	NA	NA
Albiglutide:Ertugliflozin	0	-0.0988	NA	-0.0988	NA	NA	NA	NA
Albiglutide:Exenatide	0	0.22055	NA	0.22055	NA	NA	NA	NA
Albiglutide:Inject_semaglutide	0	0.0537	NA	0.0537	NA	NA	NA	NA
Albiglutide:Liraglutide	0	-0.1825	NA	-0.1825	NA	NA	NA	NA
Albiglutide:Lixisenatide	0	-0.5539	NA	-0.5539	NA	NA	NA	NA
Albiglutide:Oral_semaglutide	0	-0.6851	NA	-0.6851	NA	NA	NA	NA
Albiglutide:Placebo_or_Control	5	-0.0434	-0.0434	NA	NA	NA	NA	NA
Albiglutide:Sotagliflozin	0	-0.7808	NA	-0.7808	NA	NA	NA	NA
Albiglutide:Tirzepatide	0	0.11138	NA	0.11138	NA	NA	NA	NA
Bexagliflozin:Canagliflozin	0	-0.4312	NA	-0.4312	NA	NA	NA	NA
Bexagliflozin:Dapagliflozin	0	0.14335	NA	0.14335	NA	NA	NA	NA
Bexagliflozin:Dulaglutide	0	0.34747	NA	0.34747	NA	NA	NA	NA
Bexagliflozin:Efpeglenatide	0	1.36568	NA	1.36568	NA	NA	NA	NA
Bexagliflozin:Empagliflozin	0	-0.2773	NA	-0.2773	NA	NA	NA	NA

Bexagliflozin:Ertugliflozin	0	-0.0536	NA	-0.0536	NA	NA	NA	NA
Bexagliflozin:Exenatide	0	0.26574	NA	0.26574	NA	NA	NA	NA
Bexagliflozin:Inject_semaglutide	0	0.09888	NA	0.09888	NA	NA	NA	NA
Bexagliflozin:Liraglutide	0	-0.1373	NA	-0.1373	NA	NA	NA	NA
Bexagliflozin:Lixisenatide	0	-0.5087	NA	-0.5087	NA	NA	NA	NA
Bexagliflozin:Oral_semaglutide	0	-0.6399	NA	-0.6399	NA	NA	NA	NA
Bexagliflozin:Placebo_or_Control	1	0.00177	0.00177	NA	NA	NA	NA	NA
Bexagliflozin:Sotagliflozin	0	-0.7356	NA	-0.7356	NA	NA	NA	NA
Bexagliflozin:Tirzepatide	0	0.15657	NA	0.15657	NA	NA	NA	NA
Canagliflozin:Dapagliflozin	0	0.57452	NA	0.57452	NA	NA	NA	NA
Canagliflozin:Dulaglutide	0	0.77863	NA	0.77863	NA	NA	NA	NA
Canagliflozin:Efpeglenatide	0	1.79685	NA	1.79685	NA	NA	NA	NA
Canagliflozin:Empagliflozin	0	0.15386	NA	0.15386	NA	NA	NA	NA
Canagliflozin:Ertugliflozin	0	0.37752	NA	0.37752	NA	NA	NA	NA
Canagliflozin:Exenatide	0	0.6969	NA	0.6969	NA	NA	NA	NA
Canagliflozin:Inject_semaglutide	1	0.53005	1.09607	0.50621	0.58986	-2.6806	3.8603	0.72371
Canagliflozin:Liraglutide	0	0.29385	NA	0.29385	NA	NA	NA	NA
Canagliflozin:Lixisenatide	0	-0.0775	NA	-0.0775	NA	NA	NA	NA
Canagliflozin:Oral_semaglutide	0	-0.2087	NA	-0.2087	NA	NA	NA	NA
Canagliflozin:Placebo_or_Control	4	0.43293	0.41612	1.00598	-0.5899	-3.8603	2.68057	0.72371
Canagliflozin:Sotagliflozin	0	-0.3045	NA	-0.3045	NA	NA	NA	NA
Canagliflozin:Tirzepatide	0	0.58773	NA	0.58773	NA	NA	NA	NA

Dapagliflozin:Dulaglutide	0	0.20412	NA	0.20412	NA	NA	NA	NA
Dapagliflozin:Efpeglenatide	0	1.22233	NA	1.22233	NA	NA	NA	NA
Dapagliflozin:Empagliflozin	0	-0.4207	NA	-0.4207	NA	NA	NA	NA
Dapagliflozin:Ertugliflozin	0	-0.197	NA	-0.197	NA	NA	NA	NA
Dapagliflozin:Exenatide	0	0.12239	NA	0.12239	NA	NA	NA	NA
Dapagliflozin:Inject_semaglutide	0	-0.0445	NA	-0.0445	NA	NA	NA	NA
Dapagliflozin:Liraglutide	0	-0.2807	NA	-0.2807	NA	NA	NA	NA
Dapagliflozin:Lixisenatide	0	-0.652	NA	-0.652	NA	NA	NA	NA
Dapagliflozin:Oral_semaglutide	0	-0.7832	NA	-0.7832	NA	NA	NA	NA
Dapagliflozin:Placebo_or_Control	6	-0.1416	-0.1416	NA	NA	NA	NA	NA
Dapagliflozin:Sotagliflozin	0	-0.879	NA	-0.879	NA	NA	NA	NA
Dapagliflozin:Tirzepatide	0	0.01322	NA	0.01322	NA	NA	NA	NA
Dulaglutide:Efpeglenatide	1	1.01822	-0.4063	1.19661	-1.6029	-5.0012	1.79536	0.35524
Dulaglutide:Empagliflozin	0	-0.6248	NA	-0.6248	NA	NA	NA	NA
Dulaglutide:Ertugliflozin	0	-0.4011	NA	-0.4011	NA	NA	NA	NA
Dulaglutide:Exenatide	0	-0.0817	NA	-0.0817	NA	NA	NA	NA
Dulaglutide:Inject_semaglutide	1	-0.2486	-1.0953	-0.2247	-0.8706	-4.1182	2.37693	0.59928
Dulaglutide:Liraglutide	0	-0.4848	NA	-0.4848	NA	NA	NA	NA
Dulaglutide:Lixisenatide	0	-0.8561	NA	-0.8561	NA	NA	NA	NA
Dulaglutide:Oral_semaglutide	0	-0.9873	NA	-0.9873	NA	NA	NA	NA
Dulaglutide:Placebo_or_Control	2	-0.3457	-0.3092	-1.5479	1.23863	-1.1269	3.60418	0.30477
Dulaglutide:Sotagliflozin	0	-1.0831	NA	-1.0831	NA	NA	NA	NA

Dulaglutide:Tirzepatide	0	-0.1909	NA	-0.1909	NA	NA	NA	NA
Efpeglenatide:Empagliflozin	0	-1.643	NA	-1.643	NA	NA	NA	NA
Efpeglenatide:Ertugliflozin	0	-1.4193	NA	-1.4193	NA	NA	NA	NA
Efpeglenatide:Exenatide	0	-1.0999	NA	-1.0999	NA	NA	NA	NA
Efpeglenatide:Inject_semaglutide	0	-1.2668	NA	-1.2668	NA	NA	NA	NA
Efpeglenatide:Liraglutide	0	-1.503	NA	-1.503	NA	NA	NA	NA
Efpeglenatide:Lixisenatide	0	-1.8744	NA	-1.8744	NA	NA	NA	NA
Efpeglenatide:Oral_semaglutide	0	-2.0056	NA	-2.0056	NA	NA	NA	NA
Efpeglenatide:Placebo_or_Control	4	-1.3639	-1.5198	0.08311	-1.6029	-5.0012	1.79536	0.35524
Efpeglenatide:Sotagliflozin	0	-2.1013	NA	-2.1013	NA	NA	NA	NA
Efpeglenatide:Tirzepatide	0	-1.2091	NA	-1.2091	NA	NA	NA	NA
Empagliflozin:Ertugliflozin	0	0.22366	NA	0.22366	NA	NA	NA	NA
Empagliflozin:Exenatide	0	0.54304	NA	0.54304	NA	NA	NA	NA
Empagliflozin:Inject_semaglutide	0	0.37618	NA	0.37618	NA	NA	NA	NA
Empagliflozin:Liraglutide	0	0.13999	NA	0.13999	NA	NA	NA	NA
Empagliflozin:Lixisenatide	0	-0.2314	NA	-0.2314	NA	NA	NA	NA
Empagliflozin:Oral_semaglutide	0	-0.3626	NA	-0.3626	NA	NA	NA	NA
Empagliflozin:Placebo_or_Control	9	0.27907	0.27907	NA	NA	NA	NA	NA
Empagliflozin:Sotagliflozin	0	-0.4583	NA	-0.4583	NA	NA	NA	NA
Empagliflozin:Tirzepatide	0	0.43387	NA	0.43387	NA	NA	NA	NA
Ertugliflozin:Exenatide	0	0.31938	NA	0.31938	NA	NA	NA	NA
Ertugliflozin:Inject_semaglutide	0	0.15252	NA	0.15252	NA	NA	NA	NA

Ertugliflozin:Liraglutide	0	-0.0837	NA	-0.0837	NA	NA	NA	NA
Ertugliflozin:Lixisenatide	0	-0.455	NA	-0.455	NA	NA	NA	NA
Ertugliflozin:Oral_semaglutide	0	-0.5862	NA	-0.5862	NA	NA	NA	NA
Ertugliflozin:Placebo_or_Control	3	0.05541	0.05541	NA	NA	NA	NA	NA
Ertugliflozin:Sotagliflozin	0	-0.682	NA	-0.682	NA	NA	NA	NA
Ertugliflozin:Tirzepatide	0	0.21021	NA	0.21021	NA	NA	NA	NA
Exenatide:Inject_semaglutide	0	-0.1669	NA	-0.1669	NA	NA	NA	NA
Exenatide:Liraglutide	0	-0.403	NA	-0.403	NA	NA	NA	NA
Exenatide:Lixisenatide	1	-0.7744	-1.0955	-0.7258	-0.3696	-3.8079	3.06868	0.83312
Exenatide:Oral_semaglutide	0	-0.9056	NA	-0.9056	NA	NA	NA	NA
Exenatide:Placebo_or_Control	3	-0.264	-0.2545	-0.6241	0.36963	-3.0687	3.80794	0.83312
Exenatide:Sotagliflozin	0	-1.0014	NA	-1.0014	NA	NA	NA	NA
Exenatide:Tirzepatide	0	-0.1092	NA	-0.1092	NA	NA	NA	NA
Inject_semaglutide:Liraglutide	1	-0.2362	-0.8376	-0.2185	-0.6191	-3.8731	2.63495	0.70924
Inject_semaglutide:Lixisenatide	0	-0.6076	NA	-0.6076	NA	NA	NA	NA
Inject_semaglutide:Oral_semaglutide	0	-0.7388	NA	-0.7388	NA	NA	NA	NA
Inject_semaglutide:Placebo_or_Control	11	-0.0971	-0.0923	-0.1968	0.10456	-1.5836	1.79266	0.90338
Inject_semaglutide:Sotagliflozin	0	-0.8345	NA	-0.8345	NA	NA	NA	NA
Inject_semaglutide:Tirzepatide	1	0.05769	0	0.06951	-0.0695	-3.5846	3.44557	0.96908
Liraglutide:Lixisenatide	0	-0.3714	NA	-0.3714	NA	NA	NA	NA
Liraglutide:Oral_semaglutide	1	-0.5026	1.62	-0.8729	2.49286	-0.8025	5.78827	0.13817
Liraglutide:Placebo_or_Control	3	0.13908	0.0879	1.65804	-1.5701	-3.905	0.76472	0.18749

Liraglutide:Sotagliflozin	0	-0.5983	NA	-0.5983	NA	NA	NA	NA
Liraglutide:Tirzepatide	0	0.29388	NA	0.29388	NA	NA	NA	NA
Lixisenatide:Oral_semaglutide	0	-0.1312	NA	-0.1312	NA	NA	NA	NA
Lixisenatide:Placebo_or_Control	1	0.51044	0.47133	0.84096	-0.3696	-3.8079	3.06868	0.83312
Lixisenatide:Sotagliflozin	0	-0.227	NA	-0.227	NA	NA	NA	NA
Lixisenatide:Tirzepatide	0	0.66524	NA	0.66524	NA	NA	NA	NA
Oral_semaglutide:Placebo_or_Control	3	0.64164	0.97184	-1.521	2.49286	-0.8025	5.78827	0.13817
Oral_semaglutide:Sotagliflozin	0	-0.0958	NA	-0.0958	NA	NA	NA	NA
Oral_semaglutide:Tirzepatide	0	0.79644	NA	0.79644	NA	NA	NA	NA
Sotagliflozin:Placebo_or_Control	4	0.73739	0.73739	NA	NA	NA	NA	NA
Tirzepatide:Placebo_or_Control	3	-0.1548	-0.1659	-0.0964	-0.0695	-3.5846	3.44557	0.96908
Sotagliflozin:Tirzepatide	0	0.89219	NA	0.89219	NA	NA	NA	NA

Table S7B: Design-by-treatment model and loop inconsistency of primary outcome: overall events of metastatic cancers

Inconsistency model	chi^2	*p* value of Prob>chi^2
design-by-treatment	3.83	0.9225
loop inconsistency	1.31	0.9338

Abbreviation: 95%CIs: 95% confidence intervals; GLP-1 agonist: glucagon-like peptide-1 agonist; NA: not applicable; NMA: network meta-analysis; OR: odds ratio; RCT: randomized controlled trial; SGLT2 inhibitor: sodium–glucose cotransporter 2 inhibitor

Table S8: Heterogeneity of primary outcome: overall events of metastatic cancers

	Heterogeneity statistic	degrees of freedom	*p*	*I squared*	*Tau-squared*	Treatments used	
C - A	1.42	4	0.842	0.00%	0	A:	Placebo_or_Control
D - A	0.96	3	0.811	0.00%	0	B:	Liraglutide
F - A	2.29	3	0.515	0.00%	0	C:	Albiglutide
H - A	8.51	8	0.385	6.00%	0.0282	D:	Canagliflozin
I - A	0.1	2	0.95	0.00%	0	E:	Tirzepatide
K - A	2.69	5	0.747	0.00%	0	F:	Efpeglenatide
L - A	0.17	1	0.676	0.00%	0	G:	Inject_semaglutide
B - A	0.31	3	0.958	0.00%	0	H:	Empagliflozin
O - A	1.67	2	0.435	0.00%	0	I:	Ertugliflozin
J - A	0	0	.	.%	0	J:	Lixisenatide
P - A	2.37	3	0.499	0.00%	0	K:	Dapagliflozin
N - A	6.44	3	0.092	53.40%	1.8821	L:	Dulaglutide
G - A	8.37	10	0.593	0.00%	0	M:	Bexagliflozin
P - B	0	0	.	.%	0	N:	Sotagliflozin
L - F	0	0	.	.%	0	O:	Exenatide
L - G	0	0	.	.%	0	P:	Oral_semaglutide
O - J	0	0	.	.%	0		
G - B	0	0	.	.%	0		
G - D	0	0	.	.%	0		
E - A	1.93	2	0.381	0.00%	0		

G - E	0	0	.	.%	0
M - A	0	0	.	.%	0

Abbreviation: 95%CIs: 95% confidence intervals; GLP-1 agonist: glucagon-like peptide-1 agonist; NA: not applicable; NMA: network meta-analysis; OR: odds ratio; RCT: randomized controlled trial; SGLT2 inhibitor: sodium–glucose cotransporter 2 inhibitor

Table S9: GRADE of primary outcome: overall events of metastatic cancers

	Comparison	No.Studies	Direct		Indirect		NMA	
			Estimate	Rate	Estimate	Rate	Estimate	Rate
1	Albiglutide:Bexagliflozin	0			-0.045186633	⊕⊕◯◯ Low	-0.045186633	⊕⊕◯◯ Low
2	Albiglutide:Canagliflozin	0			-0.476351634	⊕⊕◯◯ Low	-0.476351634	⊕⊕◯◯ Low
3	Albiglutide:Dapagliflozin	0			0.098164951	⊕⊕◯◯ Low	0.098164951	⊕⊕◯◯ Low
4	Albiglutide:Dulaglutide	0			0.302280021	⊕⊕◯◯ Low	0.302280021	⊕⊕◯◯ Low
5	Albiglutide:Efpeglenatide	0			1.320497791	⊕⊕◯◯ Low	1.320497791	⊕⊕◯◯ Low
6	Albiglutide:Empagliflozin	0			-0.322487147	⊕⊕◯◯ Low	-0.322487147	⊕⊕◯◯ Low
7	Albiglutide:Ertugliflozin	0			-0.098828578	⊕⊕◯◯ Low	-0.098828578	⊕⊕◯◯ Low
8	Albiglutide:Exenatide	0			0.220551624	⊕⊕◯◯ Low	0.220551624	⊕⊕◯◯ Low
9	Albiglutide:Inject_semaglutide	0			0.053695238	⊕⊕◯◯ Low	0.053695238	⊕⊕◯◯ Low
10	Albiglutide:Liraglutide	0			-0.18249814	⊕⊕◯◯ Low	-0.18249814	⊕⊕◯◯ Low
11	Albiglutide:Lixisenatide	0			-0.55385891	⊕⊕◯◯ Low	-0.55385891	⊕⊕◯◯ Low
12	Albiglutide:Oral_semaglutide	0			-0.685059102	⊕⊕◯◯ Low	-0.685059102	⊕⊕◯◯ Low
13	Albiglutide:Placebo_or_Control	5	-0.043418286	⊕⊕⊕⊕ High			-0.043418286	⊕⊕⊕◯ Medium
14	Albiglutide:Sotagliflozin	0			-0.780809973	⊕⊕◯◯ Low	-0.780809973	⊕⊕◯◯ Low
15	Albiglutide:Tirzepatide	0			0.111382762	⊕⊕◯◯ Low	0.111382762	⊕⊕◯◯ Low
16	Bexagliflozin:Canagliflozin	0			-0.431165	⊕⊕◯◯ Low	-0.431165	⊕⊕◯◯ Low
17	Bexagliflozin:Dapagliflozin	0			0.143351584	⊕⊕◯◯ Low	0.143351584	⊕⊕◯◯ Low
18	Bexagliflozin:Dulaglutide	0			0.347466655	⊕⊕◯◯ Low	0.347466655	⊕⊕◯◯ Low
19	Bexagliflozin:Efpeglenatide	0			1.365684425	⊕⊕◯◯ Low	1.365684425	⊕⊕◯◯ Low

20	Bexagliflozin:Empagliflozin	0			-0.277300514	⊕⊕◯◯ Low	-0.277300514	⊕⊕◯◯ Low
21	Bexagliflozin:Ertugliflozin	0			-0.053641945	⊕⊕◯◯ Low	-0.053641945	⊕⊕◯◯ Low
22	Bexagliflozin:Exenatide	0			0.265738258	⊕⊕◯◯ Low	0.265738258	⊕⊕◯◯ Low
23	Bexagliflozin:Inject_semaglutide	0			0.098881871	⊕⊕◯◯ Low	0.098881871	⊕⊕◯◯ Low
24	Bexagliflozin:Liraglutide	0			-0.137311507	⊕⊕◯◯ Low	-0.137311507	⊕⊕◯◯ Low
25	Bexagliflozin:Lixisenatide	0			-0.508672277	⊕⊕◯◯ Low	-0.508672277	⊕⊕◯◯ Low
26	Bexagliflozin:Oral_semaglutide	0			-0.639872468	⊕⊕◯◯ Low	-0.639872468	⊕⊕◯◯ Low
27	Bexagliflozin:Placebo_or_Control	1	0.001768347	⊕⊕⊕⊕ High			0.001768347	⊕⊕⊕◯ Medium
28	Bexagliflozin:Sotagliflozin	0			-0.735623339	⊕⊕◯◯ Low	-0.735623339	⊕⊕◯◯ Low
29	Bexagliflozin:Tirzepatide	0			0.156569395	⊕⊕◯◯ Low	0.156569395	⊕⊕◯◯ Low
30	Canagliflozin:Dapagliflozin	0			0.574516584	⊕⊕◯◯ Low	0.574516584	⊕⊕◯◯ Low
31	Canagliflozin:Dulaglutide	0			0.778631655	⊕⊕◯◯ Low	0.778631655	⊕⊕◯◯ Low
32	Canagliflozin:Efpeglenatide	0			1.796849425	⊕⊕◯◯ Low	1.796849425	⊕⊕◯◯ Low
33	Canagliflozin:Empagliflozin	0			0.153864486	⊕⊕◯◯ Low	0.153864486	⊕⊕◯◯ Low
34	Canagliflozin:Ertugliflozin	0			0.377523055	⊕⊕◯◯ Low	0.377523055	⊕⊕◯◯ Low
35	Canagliflozin:Exenatide	0			0.696903258	⊕⊕◯◯ Low	0.696903258	⊕⊕◯◯ Low
36	Canagliflozin:Inject_semaglutide	1	1.096067758	⊕⊕⊕⊕ High	0.506205897	⊕⊕⊕⊕ High	0.530046871	⊕⊕⊕⊕ High
37	Canagliflozin:Liraglutide	0			0.293853493	⊕⊕◯◯ Low	0.293853493	⊕⊕◯◯ Low
38	Canagliflozin:Lixisenatide	0			-0.077507277	⊕⊕◯◯ Low	-0.077507277	⊕⊕◯◯ Low
39	Canagliflozin:Oral_semaglutide	0			-0.208707468	⊕⊕◯◯ Low	-0.208707468	⊕⊕◯◯ Low
40	Canagliflozin:Placebo_or_Control	4	0.416122559	⊕⊕⊕⊕ High	1.00598442	⊕⊕⊕⊕ High	0.432933347	⊕⊕⊕⊕ High
41	Canagliflozin:Sotagliflozin	0			-0.304458339	⊕⊕◯◯ Low	-0.304458339	⊕⊕◯◯ Low

42	Canagliflozin:Tirzepatide	0			0.587734395	⊕⊕◯◯ Low	0.587734395	⊕⊕◯◯ Low
43	Dapagliflozin:Dulaglutide	0			0.204115071	⊕⊕◯◯ Low	0.204115071	⊕⊕◯◯ Low
44	Dapagliflozin:Efpeglenatide	0			1.222332841	⊕⊕◯◯ Low	1.222332841	⊕⊕◯◯ Low
45	Dapagliflozin:Empagliflozin	0			-0.420652098	⊕⊕◯◯ Low	-0.420652098	⊕⊕◯◯ Low
46	Dapagliflozin:Ertugliflozin	0			-0.196993529	⊕⊕◯◯ Low	-0.196993529	⊕⊕◯◯ Low
47	Dapagliflozin:Exenatide	0			0.122386673	⊕⊕◯◯ Low	0.122386673	⊕⊕◯◯ Low
48	Dapagliflozin:Inject_semaglutide	0			-0.044469713	⊕⊕◯◯ Low	-0.044469713	⊕⊕◯◯ Low
49	Dapagliflozin:Liraglutide	0			-0.280663091	⊕⊕◯◯ Low	-0.280663091	⊕⊕◯◯ Low
50	Dapagliflozin:Lixisenatide	0			-0.652023861	⊕⊕◯◯ Low	-0.652023861	⊕⊕◯◯ Low
51	Dapagliflozin:Oral_semaglutide	0			-0.783224052	⊕⊕◯◯ Low	-0.783224052	⊕⊕◯◯ Low
52	Dapagliflozin:Placebo_or_Control	6	-0.141583237	⊕⊕⊕⊕ High			-0.141583237	⊕⊕⊕◯ Medium
53	Dapagliflozin:Sotagliflozin	0			-0.878974923	⊕⊕◯◯ Low	-0.878974923	⊕⊕◯◯ Low
54	Dapagliflozin:Tirzepatide	0			0.013217811	⊕⊕◯◯ Low	0.013217811	⊕⊕◯◯ Low
55	Dulaglutide:Efpeglenatide	1	-0.406291896	⊕⊕⊕⊕ High	1.19661	⊕⊕⊕◯ Medium	1.01821777	⊕⊕⊕⊕ High
56	Dulaglutide:Empagliflozin	0			-0.624767169	⊕⊕◯◯ Low	-0.624767169	⊕⊕◯◯ Low
57	Dulaglutide:Ertugliflozin	0			-0.4011086	⊕⊕◯◯ Low	-0.4011086	⊕⊕◯◯ Low
58	Dulaglutide:Exenatide	0			-0.081728397	⊕⊕◯◯ Low	-0.081728397	⊕⊕◯◯ Low
59	Dulaglutide:Inject_semaglutide	1	-1.095276172	⊕⊕⊕⊕ High	-0.224652744	⊕⊕⊕⊕ High	-0.248584784	⊕⊕⊕⊕ High
60	Dulaglutide:Liraglutide	0			-0.484778162	⊕⊕◯◯ Low	-0.484778162	⊕⊕◯◯ Low
61	Dulaglutide:Lixisenatide	0			-0.856138932	⊕⊕◯◯ Low	-0.856138932	⊕⊕◯◯ Low
62	Dulaglutide:Oral_semaglutide	0			-0.987339123	⊕⊕◯◯ Low	-0.987339123	⊕⊕◯◯ Low
63	Dulaglutide:Placebo_or_Control	2	-0.309228015	⊕⊕⊕⊕ High	-1.547862487	⊕⊕⊕⊕ High	-0.345698308	⊕⊕⊕⊕ High

64	Dulaglutide:Sotagliflozin	0			-1.083089994	⊕⊕◯◯ Low	-1.083089994	⊕⊕◯◯ Low
65	Dulaglutide:Tirzepatide	0			-0.190897259	⊕⊕◯◯ Low	-0.190897259	⊕⊕◯◯ Low
66	Efpeglenatide:Empagliflozin	0			-1.642984939	⊕⊕◯◯ Low	-1.642984939	⊕⊕◯◯ Low
67	Efpeglenatide:Ertugliflozin	0			-1.41932637	⊕⊕◯◯ Low	-1.41932637	⊕⊕◯◯ Low
68	Efpeglenatide:Exenatide	0			-1.099946167	⊕⊕◯◯ Low	-1.099946167	⊕⊕◯◯ Low
69	Efpeglenatide:Inject_semaglutide	0			-1.266802554	⊕⊕◯◯ Low	-1.266802554	⊕⊕◯◯ Low
70	Efpeglenatide:Liraglutide	0			-1.502995932	⊕⊕◯◯ Low	-1.502995932	⊕⊕◯◯ Low
71	Efpeglenatide:Lixisenatide	0			-1.874356702	⊕⊕◯◯ Low	-1.874356702	⊕⊕◯◯ Low
72	Efpeglenatide:Oral_semaglutide	0			-2.005556893	⊕⊕◯◯ Low	-2.005556893	⊕⊕◯◯ Low
73	Efpeglenatide:Placebo_or_Control	4	-1.519796316	⊕⊕⊕⊕ High	0.08310558	⊕⊕⊕◯ Medium	-1.363916078	⊕⊕⊕⊕ High
74	Efpeglenatide:Sotagliflozin	0			-2.101307764	⊕⊕◯◯ Low	-2.101307764	⊕⊕◯◯ Low
75	Efpeglenatide:Tirzepatide	0			-1.209115029	⊕⊕◯◯ Low	-1.209115029	⊕⊕◯◯ Low
76	Empagliflozin:Ertugliflozin	0			0.223658569	⊕⊕◯◯ Low	0.223658569	⊕⊕◯◯ Low
77	Empagliflozin:Exenatide	0			0.543038771	⊕⊕◯◯ Low	0.543038771	⊕⊕◯◯ Low
78	Empagliflozin:Inject_semaglutide	0			0.376182385	⊕⊕◯◯ Low	0.376182385	⊕⊕◯◯ Low
79	Empagliflozin:Liraglutide	0			0.139989007	⊕⊕◯◯ Low	0.139989007	⊕⊕◯◯ Low
80	Empagliflozin:Lixisenatide	0			-0.231371763	⊕⊕◯◯ Low	-0.231371763	⊕⊕◯◯ Low
81	Empagliflozin:Oral_semaglutide	0			-0.362571954	⊕⊕◯◯ Low	-0.362571954	⊕⊕◯◯ Low
82	Empagliflozin:Placebo_or_Control	9	0.279068861	⊕⊕⊕⊕ High			0.279068861	⊕⊕⊕◯ Medium
83	Empagliflozin:Sotagliflozin	0			-0.458322825	⊕⊕◯◯ Low	-0.458322825	⊕⊕◯◯ Low
84	Empagliflozin:Tirzepatide	0			0.433869909	⊕⊕◯◯ Low	0.433869909	⊕⊕◯◯ Low
85	Ertugliflozin:Exenatide	0			0.319380202	⊕⊕◯◯ Low	0.319380202	⊕⊕◯◯ Low

86	Ertugliflozin:Inject_semaglutide	0			0.152523816	⊕⊕◯◯ Low	0.152523816	⊕⊕◯◯ Low
87	Ertugliflozin:Liraglutide	0			-0.083669562	⊕⊕◯◯ Low	-0.083669562	⊕⊕◯◯ Low
88	Ertugliflozin:Lixisenatide	0			-0.455030332	⊕⊕◯◯ Low	-0.455030332	⊕⊕◯◯ Low
89	Ertugliflozin:Oral_semaglutide	0			-0.586230523	⊕⊕◯◯ Low	-0.586230523	⊕⊕◯◯ Low
90	Ertugliflozin:Placebo_or_Control	3	0.055410292	⊕⊕⊕⊕ High			0.055410292	⊕⊕⊕◯ Medium
91	Ertugliflozin:Sotagliflozin	0			-0.681981394	⊕⊕◯◯ Low	-0.681981394	⊕⊕◯◯ Low
92	Ertugliflozin:Tirzepatide	0			0.21021134	⊕⊕◯◯ Low	0.21021134	⊕⊕◯◯ Low
93	Exenatide:Inject_semaglutide	0			-0.166856387	⊕⊕◯◯ Low	-0.166856387	⊕⊕◯◯ Low
94	Exenatide:Liraglutide	0			-0.403049765	⊕⊕◯◯ Low	-0.403049765	⊕⊕◯◯ Low
95	Exenatide:Lixisenatide	1	-1.095457712	⊕⊕⊕⊕ High	-0.72582684	⊕⊕⊕⊕ High	-0.774410534	⊕⊕⊕⊕ High
96	Exenatide:Oral_semaglutide	0			-0.905610726	⊕⊕◯◯ Low	-0.905610726	⊕⊕◯◯ Low
97	Exenatide:Placebo_or_Control	3	-0.254500897	⊕⊕⊕⊕ High	-0.624131768	⊕⊕⊕⊕ High	-0.26396991	⊕⊕⊕⊕ High
98	Exenatide:Sotagliflozin	0			-1.001361597	⊕⊕◯◯ Low	-1.001361597	⊕⊕◯◯ Low
99	Exenatide:Tirzepatide	0			-0.109168862	⊕⊕◯◯ Low	-0.109168862	⊕⊕◯◯ Low
100	Inject_semaglutide:Liraglutide	1	-0.837589046	⊕⊕⊕⊕ High	-0.218521272	⊕⊕⊕⊕ High	-0.236193378	⊕⊕⊕⊕ High
101	Inject_semaglutide:Lixisenatide	0			-0.607554148	⊕⊕◯◯ Low	-0.607554148	⊕⊕◯◯ Low
102	Inject_semaglutide:Oral_semaglutide	0			-0.738754339	⊕⊕◯◯ Low	-0.738754339	⊕⊕◯◯ Low
103	Inject_semaglutide:Placebo_or_Control	11	-0.092267589	⊕⊕⊕⊕ High	-0.196823146	⊕⊕⊕⊕ High	-0.097113524	⊕⊕⊕⊕ High
104	Inject_semaglutide:Sotagliflozin	0			-0.83450521	⊕⊕◯◯ Low	-0.83450521	⊕⊕◯◯ Low
105	Inject_semaglutide:Tirzepatide	1	0	⊕⊕⊕⊕ High	0.069506478	⊕⊕⊕⊕ High	0.057687524	⊕⊕⊕⊕ High
106	Liraglutide:Lixisenatide	0			-0.37136077	⊕⊕◯◯ Low	-0.37136077	⊕⊕◯◯ Low
107	Liraglutide:Oral_semaglutide	1	1.620001391	⊕⊕⊕⊕ High	-0.87286253	⊕⊕⊕◯ Medium	-0.502560961	⊕⊕⊕⊕ High

108	Liraglutide:Placebo_or_Control	3	0.087904631	⊕⊕⊕⊕ High	1.658036567	⊕⊕⊕⊕ High	0.139079854	⊕⊕⊕⊕ High
109	Liraglutide:Sotagliflozin	0			-0.598311832	⊕⊕◯◯ Low	-0.598311832	⊕⊕◯◯ Low
110	Liraglutide:Tirzepatide	0			0.293880902	⊕⊕◯◯ Low	0.293880902	⊕⊕◯◯ Low
111	Lixisenatide:Oral_semaglutide	0			-0.131200191	⊕⊕◯◯ Low	-0.131200191	⊕⊕◯◯ Low
112	Lixisenatide:Placebo_or_Control	1	0.471325943	⊕⊕⊕⊕ High	0.840956815	⊕⊕⊕⊕ High	0.510440624	⊕⊕⊕⊕ High
113	Lixisenatide:Sotagliflozin	0			-0.226951062	⊕⊕◯◯ Low	-0.226951062	⊕⊕◯◯ Low
114	Lixisenatide:Tirzepatide	0			0.665241672	⊕⊕◯◯ Low	0.665241672	⊕⊕◯◯ Low
115	Oral_semaglutide:Placebo_or_Control	3	0.97183959	⊕⊕⊕⊕ High	-1.52102433	⊕⊕⊕◯ Medium	0.641640815	⊕⊕⊕⊕ High
116	Oral_semaglutide:Sotagliflozin	0			-0.095750871	⊕⊕◯◯ Low	-0.095750871	⊕⊕◯◯ Low
117	Oral_semaglutide:Tirzepatide	0			0.796441864	⊕⊕◯◯ Low	0.796441864	⊕⊕◯◯ Low
118	Sotagliflozin:Placebo_or_Control	4	0.737391686	⊕⊕⊕⊕ High			0.737391686	⊕⊕⊕◯ Medium
119	Tirzepatide:Placebo_or_Control	3	-0.16590407	⊕⊕⊕⊕ High	-0.096397593	⊕⊕⊕⊕ High	-0.154801048	⊕⊕⊕⊕ High
120	Sotagliflozin:Tirzepatide	0			0.892192735	⊕⊕◯◯ Low	0.892192735	⊕⊕◯◯ Low

Reference list of supplement tables:

1. Page, M.J.; McKenzie, J.E.; Bossuyt, P.M.; Boutron, I.; Hoffmann, T.C.; Mulrow, C.D.; Shamseer, L.; Tetzlaff, J.M.; Akl, E.A.; Brennan, S.E.; et al. The PRISMA 2020 statement: an updated guideline for reporting systematic reviews. *Bmj* **2021**, *372*, n71, doi:10.1136/bmj.n71.
2. Nauck, M.A.; Jensen, T.J.; Rosenkilde, C.; Calanna, S.; Buse, J.B.; Investigators, L.P.C.o.b.o.t.L.T. Neoplasms Reported With Liraglutide or Placebo in People With Type 2 Diabetes: Results From the LEADER Randomized Trial. *Diabetes Care* **2018**, *41*, 1663-1671, doi:10.2337/dc17-1825.
3. Popovic, D.S.; Patoulias, D.; Popovic, L.S.; Karakasis, P.; Papanas, N.; Mantzoros, C.S. Tirzepatide use and the risk of cancer among individuals with type 2 diabetes mellitus: A meta-analysis of randomized controlled trials. *Diabetes Res Clin Pract* **2024**, *213*, 111758, doi:10.1016/j.diabres.2024.111758.
4. Kuo, H.H.; Wang, K.T.; Chen, H.H.; Lai, Z.Y.; Lin, P.L.; Chuang, Y.J.; Liu, L.Y. Cardiovascular outcomes associated with SGLT2 inhibitor therapy in patients with type 2 diabetes mellitus and cancer: a systematic review and meta-analysis. *Diabetol Metab Syndr* **2024**, *16*, 108, doi:10.1186/s13098-024-01354-4.
5. Xu, B.; Kang, B.; Li, S.; Fan, S.; Zhou, J. Sodium-glucose cotransporter 2 inhibitors and cancer: a systematic review and meta-analysis. *Journal of endocrinological investigation* **2024**, *47*, 2421-2436, doi:10.1007/s40618-024-02351-0.
6. Nagendra, L.; Bg, H.; Sharma, M.; Dutta, D. Semaglutide and cancer: A systematic review and meta-analysis. *Diabetes Metab Syndr* **2023**, *17*, 102834, doi:10.1016/j.dsx.2023.102834.
7. He, L.; Wang, J.; Ping, F.; Yang, N.; Huang, J.; Li, Y.; Xu, L.; Li, W.; Zhang, H. Association of Glucagon-Like Peptide-1 Receptor Agonist Use With Risk of Gallbladder and Biliary Diseases: A Systematic Review and Meta-analysis of Randomized Clinical Trials. *JAMA Intern Med* **2022**, *182*, 513-519, doi:10.1001/jamainternmed.2022.0338.
8. Azoulay, L.; Filion, K.B.; Platt, R.W.; Dahl, M.; Dormuth, C.R.; Clemens, K.K.; Durand, M.; Juurlink, D.N.; Targownik, L.E.; Turin, T.C.; et al.

Incretin based drugs and the risk of pancreatic cancer: international multicentre cohort study. *Bmj* **2016**, *352*, i581, doi:10.1136/bmj.i581.
9. Stenlof, K.; Cefalu, W.T.; Kim, K.A.; Jodar, E.; Alba, M.; Edwards, R.; Tong, C.; Canovatchel, W.; Meininger, G. Long-term efficacy and safety of canagliflozin monotherapy in patients with type 2 diabetes inadequately controlled with diet and exercise: findings from the 52-week CANTATA-M study. *Curr Med Res Opin* **2014**, *30*, 163-175, doi:10.1185/03007995.2013.850066.
10. Lavalle-Gonzalez, F.J.; Januszewicz, A.; Davidson, J.; Tong, C.; Qiu, R.; Canovatchel, W.; Meininger, G. Efficacy and safety of canagliflozin compared with placebo and sitagliptin in patients with type 2 diabetes on background metformin monotherapy: a randomised trial. *Diabetologia* **2013**, *56*, 2582-2592, doi:10.1007/s00125-013-3039-1.
11. Charbonnel, B.; Steinberg, H.; Eymard, E.; Xu, L.; Thakkar, P.; Prabhu, V.; Davies, M.J.; Engel, S.S. Efficacy and safety over 26 weeks of an oral treatment strategy including sitagliptin compared with an injectable treatment strategy with liraglutide in patients with type 2 diabetes mellitus inadequately controlled on metformin: a randomised clinical trial. *Diabetologia* **2013**, *56*, 1503-1511, doi:10.1007/s00125-013-2905-1.
12. Schernthaner, G.; Gross, J.L.; Rosenstock, J.; Guarisco, M.; Fu, M.; Yee, J.; Kawaguchi, M.; Canovatchel, W.; Meininger, G. Canagliflozin compared with sitagliptin for patients with type 2 diabetes who do not have adequate glycemic control with metformin plus sulfonylurea: a 52-week randomized trial. *Diabetes Care* **2013**, *36*, 2508-2515, doi:10.2337/dc12-2491.
13. Wilding, J.P.; Woo, V.; Soler, N.G.; Pahor, A.; Sugg, J.; Rohwedder, K.; Parikh, S.; Dapagliflozin 006 Study, G. Long-term efficacy of dapagliflozin in patients with type 2 diabetes mellitus receiving high doses of insulin: a randomized trial. *Ann Intern Med* **2012**, *156*, 405-415, doi:10.7326/0003-4819-156-6-201203200-00003.
14. Gallwitz, B.; Bohmer, M.; Segiet, T.; Molle, A.; Milek, K.; Becker, B.; Helsberg, K.; Petto, H.; Peters, N.; Bachmann, O. Exenatide twice daily versus premixed insulin aspart 70/30 in metformin-treated patients with type 2 diabetes: a randomized 26-week study on glycemic control and hypoglycemia. *Diabetes Care* **2011**, *34*, 604-606, doi:10.2337/dc10-1900.
15. Bailey, C.J.; Gross, J.L.; Pieters, A.; Bastien, A.; List, J.F. Effect of dapagliflozin in patients with type 2 diabetes who have inadequate glycaemic control with metformin: a randomised, double-blind, placebo-controlled trial. *Lancet* **2010**, *375*, 2223-2233,

doi:10.1016/S0140-6736(10)60407-2.
16. Buse, J.B.; Rosenstock, J.; Sesti, G.; Schmidt, W.E.; Montanya, E.; Brett, J.H.; Zychma, M.; Blonde, L.; Group, L.-S. Liraglutide once a day versus exenatide twice a day for type 2 diabetes: a 26-week randomised, parallel-group, multinational, open-label trial (LEAD-6). *Lancet* **2009**, *374*, 39-47, doi:10.1016/S0140-6736(09)60659-0.
17. Nauck, M.; Frid, A.; Hermansen, K.; Shah, N.S.; Tankova, T.; Mitha, I.H.; Zdravkovic, M.; During, M.; Matthews, D.R.; Group, L.-S. Efficacy and safety comparison of liraglutide, glimepiride, and placebo, all in combination with metformin, in type 2 diabetes: the LEAD (liraglutide effect and action in diabetes)-2 study. *Diabetes Care* **2009**, *32*, 84-90, doi:10.2337/dc08-1355.
18. Garber, A.; Henry, R.; Ratner, R.; Garcia-Hernandez, P.A.; Rodriguez-Pattzi, H.; Olvera-Alvarez, I.; Hale, P.M.; Zdravkovic, M.; Bode, B.; Group, L.-S. Liraglutide versus glimepiride monotherapy for type 2 diabetes (LEAD-3 Mono): a randomised, 52-week, phase III, double-blind, parallel-treatment trial. *Lancet* **2009**, *373*, 473-481, doi:10.1016/S0140-6736(08)61246-5.
19. Kovacs, C.S.; Seshiah, V.; Merker, L.; Christiansen, A.V.; Roux, F.; Salsali, A.; Kim, G.; Stella, P.; Woerle, H.J.; Broedl, U.C.; et al. Empagliflozin as Add-on Therapy to Pioglitazone With or Without Metformin in Patients With Type 2 Diabetes Mellitus. *Clin Ther* **2015**, *37*, 1773-1788 e1771, doi:10.1016/j.clinthera.2015.05.511.
20. Blonde, L.; Jendle, J.; Gross, J.; Woo, V.; Jiang, H.; Fahrbach, J.L.; Milicevic, Z. Once-weekly dulaglutide versus bedtime insulin glargine, both in combination with prandial insulin lispro, in patients with type 2 diabetes (AWARD-4): a randomised, open-label, phase 3, non-inferiority study. *Lancet* **2015**, *385*, 2057-2066, doi:10.1016/S0140-6736(15)60936-9.
21. Weinstock, R.S.; Guerci, B.; Umpierrez, G.; Nauck, M.A.; Skrivanek, Z.; Milicevic, Z. Safety and efficacy of once-weekly dulaglutide versus sitagliptin after 2 years in metformin-treated patients with type 2 diabetes (AWARD-5): a randomized, phase III study. *Diabetes Obes Metab* **2015**, *17*, 849-858, doi:10.1111/dom.12479.
22. Cefalu, W.T.; Leiter, L.A.; de Bruin, T.W.; Gause-Nilsson, I.; Sugg, J.; Parikh, S.J. Dapagliflozin's Effects on Glycemia and Cardiovascular Risk Factors in High-Risk Patients With Type 2 Diabetes: A 24-Week, Multicenter, Randomized, Double-Blind, Placebo-Controlled Study With a 28-Week Extension. *Diabetes Care* **2015**, *38*, 1218-1227, doi:10.2337/dc14-0315.
23. Dungan, K.M.; Povedano, S.T.; Forst, T.; Gonzalez, J.G.; Atisso, C.; Sealls, W.; Fahrbach, J.L. Once-weekly dulaglutide versus once-daily

liraglutide in metformin-treated patients with type 2 diabetes (AWARD-6): a randomised, open-label, phase 3, non-inferiority trial. *Lancet* **2014**, *384*, 1349-1357, doi:10.1016/S0140-6736(14)60976-4.

24. Rosenstock, J.; Fonseca, V.A.; Gross, J.L.; Ratner, R.E.; Ahren, B.; Chow, F.C.; Yang, F.; Miller, D.; Johnson, S.L.; Stewart, M.W.; et al. Advancing basal insulin replacement in type 2 diabetes inadequately controlled with insulin glargine plus oral agents: a comparison of adding albiglutide, a weekly GLP-1 receptor agonist, versus thrice-daily prandial insulin lispro. *Diabetes Care* **2014**, *37*, 2317-2325, doi:10.2337/dc14-0001.
25. Wysham, C.; Blevins, T.; Arakaki, R.; Colon, G.; Garcia, P.; Atisso, C.; Kuhstoss, D.; Lakshmanan, M. Efficacy and safety of dulaglutide added onto pioglitazone and metformin versus exenatide in type 2 diabetes in a randomized controlled trial (AWARD-1). *Diabetes Care* **2014**, *37*, 2159-2167, doi:10.2337/dc13-2760.
26. Umpierrez, G.; Tofe Povedano, S.; Perez Manghi, F.; Shurzinske, L.; Pechtner, V. Efficacy and safety of dulaglutide monotherapy versus metformin in type 2 diabetes in a randomized controlled trial (AWARD-3). *Diabetes Care* **2014**, *37*, 2168-2176, doi:10.2337/dc13-2759.
27. Pratley, R.E.; Nauck, M.A.; Barnett, A.H.; Feinglos, M.N.; Ovalle, F.; Harman-Boehm, I.; Ye, J.; Scott, R.; Johnson, S.; Stewart, M.; et al. Once-weekly albiglutide versus once-daily liraglutide in patients with type 2 diabetes inadequately controlled on oral drugs (HARMONY 7): a randomised, open-label, multicentre, non-inferiority phase 3 study. *Lancet Diabetes Endocrinol* **2014**, *2*, 289-297, doi:10.1016/S2213-8587(13)70214-6.
28. Polidori, D.; Mari, A.; Ferrannini, E. Canagliflozin, a sodium glucose co-transporter 2 inhibitor, improves model-based indices of beta cell function in patients with type 2 diabetes. *Diabetologia* **2014**, *57*, 891-901, doi:10.1007/s00125-014-3196-x.
29. Grunberger, G.; Camp, S.; Johnson, J.; Huyck, S.; Terra, S.G.; Mancuso, J.P.; Jiang, Z.W.; Golm, G.; Engel, S.S.; Lauring, B. Ertugliflozin in Patients with Stage 3 Chronic Kidney Disease and Type 2 Diabetes Mellitus: The VERTIS RENAL Randomized Study. *Diabetes Ther* **2018**, *9*, 49-66, doi:10.1007/s13300-017-0337-5.
30. Yu, M.; Brunt, K.V.; Milicevic, Z.; Varnado, O.; Boye, K.S. Patient-reported Outcomes in Patients with Type 2 Diabetes Treated with Dulaglutide Added to Titrated Insulin Glargine (AWARD-9). *Clin Ther* **2017**, *39*, 2284-2295, doi:10.1016/j.clinthera.2017.10.002.
31. Home, P.D.; Ahren, B.; Reusch, J.E.B.; Rendell, M.; Weissman, P.N.; Cirkel, D.T.; Miller, D.; Ambery, P.; Carr, M.C.; Nauck, M.A. Three-year

data from 5 HARMONY phase 3 clinical trials of albiglutide in type 2 diabetes mellitus: Long-term efficacy with or without rescue therapy. *Diabetes Res Clin Pract* **2017**, *131*, 49-60, doi:10.1016/j.diabres.2017.06.013.

32. Meneilly, G.S.; Roy-Duval, C.; Alawi, H.; Dailey, G.; Bellido, D.; Trescoli, C.; Manrique Hurtado, H.; Guo, H.; Pilorget, V.; Perfetti, R.; et al. Lixisenatide Therapy in Older Patients With Type 2 Diabetes Inadequately Controlled on Their Current Antidiabetic Treatment: The GetGoal-O Randomized Trial. *Diabetes Care* **2017**, *40*, 485-493, doi:10.2337/dc16-2143.
33. Nauck, M.; Rizzo, M.; Johnson, A.; Bosch-Traberg, H.; Madsen, J.; Cariou, B. Once-Daily Liraglutide Versus Lixisenatide as Add-on to Metformin in Type 2 Diabetes: A 26-Week Randomized Controlled Clinical Trial. *Diabetes Care* **2016**, *39*, 1501-1509, doi:10.2337/dc15-2479.
34. Leiter, L.A.; Cefalu, W.T.; de Bruin, T.W.; Xu, J.; Parikh, S.; Johnsson, E.; Gause-Nilsson, I. Long-term maintenance of efficacy of dapagliflozin in patients with type 2 diabetes mellitus and cardiovascular disease. *Diabetes Obes Metab* **2016**, *18*, 766-774, doi:10.1111/dom.12666.
35. Dungan, K.M.; Weitgasser, R.; Perez Manghi, F.; Pintilei, E.; Fahrbach, J.L.; Jiang, H.H.; Shell, J.; Robertson, K.E. A 24-week study to evaluate the efficacy and safety of once-weekly dulaglutide added on to glimepiride in type 2 diabetes (AWARD-8). *Diabetes Obes Metab* **2016**, *18*, 475-482, doi:10.1111/dom.12634.
36. Davies, M.J.; Bain, S.C.; Atkin, S.L.; Rossing, P.; Scott, D.; Shamkhalova, M.S.; Bosch-Traberg, H.; Syren, A.; Umpierrez, G.E. Efficacy and Safety of Liraglutide Versus Placebo as Add-on to Glucose-Lowering Therapy in Patients With Type 2 Diabetes and Moderate Renal Impairment (LIRA-RENAL): A Randomized Clinical Trial. *Diabetes Care* **2016**, *39*, 222-230, doi:10.2337/dc14-2883.
37. Davies, M.J.; Bergenstal, R.; Bode, B.; Kushner, R.F.; Lewin, A.; Skjoth, T.V.; Andreasen, A.H.; Jensen, C.B.; DeFronzo, R.A.; Group, N.N.S. Efficacy of Liraglutide for Weight Loss Among Patients With Type 2 Diabetes: The SCALE Diabetes Randomized Clinical Trial. *Jama* **2015**, *314*, 687-699, doi:10.1001/jama.2015.9676.
38. Mathieu, C.; Ranetti, A.E.; Li, D.; Ekholm, E.; Cook, W.; Hirshberg, B.; Chen, H.; Hansen, L.; Iqbal, N. Randomized, Double-Blind, Phase 3 Trial of Triple Therapy With Dapagliflozin Add-on to Saxagliptin Plus Metformin in Type 2 Diabetes. *Diabetes Care* **2015**, *38*, 2009-2017, doi:10.2337/dc15-0779.

39. Rodbard, H.W.; Rosenstock, J.; Canani, L.H.; Deerochanawong, C.; Gumprecht, J.; Lindberg, S.O.; Lingvay, I.; Sondergaard, A.L.; Treppendahl, M.B.; Montanya, E.; et al. Oral Semaglutide Versus Empagliflozin in Patients With Type 2 Diabetes Uncontrolled on Metformin: The PIONEER 2 Trial. *Diabetes Care* **2019**, *42*, 2272-2281, doi:10.2337/dc19-0883.
40. Mullins, R.J.; Mustapic, M.; Chia, C.W.; Carlson, O.; Gulyani, S.; Tran, J.; Li, Y.; Mattson, M.P.; Resnick, S.; Egan, J.M.; et al. A Pilot Study of Exenatide Actions in Alzheimer's Disease. *Curr Alzheimer Res* **2019**, *16*, 741-752, doi:10.2174/1567205016666190913155950.
41. Mosenzon, O.; Blicher, T.M.; Rosenlund, S.; Eriksson, J.W.; Heller, S.; Hels, O.H.; Pratley, R.; Sathyapalan, T.; Desouza, C.; Investigators, P. Efficacy and safety of oral semaglutide in patients with type 2 diabetes and moderate renal impairment (PIONEER 5): a placebo-controlled, randomised, phase 3a trial. *Lancet Diabetes Endocrinol* **2019**, *7*, 515-527, doi:10.1016/S2213-8587(19)30192-5.
42. Aroda, V.R.; Rosenstock, J.; Terauchi, Y.; Altuntas, Y.; Lalic, N.M.; Morales Villegas, E.C.; Jeppesen, O.K.; Christiansen, E.; Hertz, C.L.; Haluzik, M.; et al. PIONEER 1: Randomized Clinical Trial of the Efficacy and Safety of Oral Semaglutide Monotherapy in Comparison With Placebo in Patients With Type 2 Diabetes. *Diabetes Care* **2019**, *42*, 1724-1732, doi:10.2337/dc19-0749.
43. Coskun, T.; Sloop, K.W.; Loghin, C.; Alsina-Fernandez, J.; Urva, S.; Bokvist, K.B.; Cui, X.; Briere, D.A.; Cabrera, O.; Roell, W.C.; et al. LY3298176, a novel dual GIP and GLP-1 receptor agonist for the treatment of type 2 diabetes mellitus: From discovery to clinical proof of concept. *Mol Metab* **2018**, *18*, 3-14, doi:10.1016/j.molmet.2018.09.009.
44. Frias, J.P.; Nauck, M.A.; Van, J.; Kutner, M.E.; Cui, X.; Benson, C.; Urva, S.; Gimeno, R.E.; Milicevic, Z.; Robins, D.; et al. Efficacy and safety of LY3298176, a novel dual GIP and GLP-1 receptor agonist, in patients with type 2 diabetes: a randomised, placebo-controlled and active comparator-controlled phase 2 trial. *Lancet* **2018**, *392*, 2180-2193, doi:10.1016/S0140-6736(18)32260-8.
45. Buse, J.B.; Garg, S.K.; Rosenstock, J.; Bailey, T.S.; Banks, P.; Bode, B.W.; Danne, T.; Kushner, J.A.; Lane, W.S.; Lapuerta, P.; et al. Sotagliflozin in Combination With Optimized Insulin Therapy in Adults With Type 1 Diabetes: The North American inTandem1 Study. *Diabetes Care* **2018**, *41*, 1970-1980, doi:10.2337/dc18-0343.
46. Tuttle, K.R.; Lakshmanan, M.C.; Rayner, B.; Busch, R.S.; Zimmermann, A.G.; Woodward, D.B.; Botros, F.T. Dulaglutide versus insulin glargine in patients with type 2 diabetes and moderate-to-severe chronic kidney disease (AWARD-7): a multicentre, open-label, randomised trial. *Lancet Diabetes Endocrinol* **2018**, *6*, 605-617, doi:10.1016/S2213-8587(18)30104-9.

47. Ludvik, B.; Frias, J.P.; Tinahones, F.J.; Wainstein, J.; Jiang, H.; Robertson, K.E.; Garcia-Perez, L.E.; Woodward, D.B.; Milicevic, Z. Dulaglutide as add-on therapy to SGLT2 inhibitors in patients with inadequately controlled type 2 diabetes (AWARD-10): a 24-week, randomised, double-blind, placebo-controlled trial. *Lancet Diabetes Endocrinol* **2018**, *6*, 370-381, doi:10.1016/S2213-8587(18)30023-8.
48. Ahmann, A.J.; Capehorn, M.; Charpentier, G.; Dotta, F.; Henkel, E.; Lingvay, I.; Holst, A.G.; Annett, M.P.; Aroda, V.R. Efficacy and Safety of Once-Weekly Semaglutide Versus Exenatide ER in Subjects With Type 2 Diabetes (SUSTAIN 3): A 56-Week, Open-Label, Randomized Clinical Trial. *Diabetes Care* **2018**, *41*, 258-266, doi:10.2337/dc17-0417.
49. Rubino, D.M.; Greenway, F.L.; Khalid, U.; O'Neil, P.M.; Rosenstock, J.; Sorrig, R.; Wadden, T.A.; Wizert, A.; Garvey, W.T.; Investigators, S. Effect of Weekly Subcutaneous Semaglutide vs Daily Liraglutide on Body Weight in Adults With Overweight or Obesity Without Diabetes: The STEP 8 Randomized Clinical Trial. *Jama* **2022**, *327*, 138-150, doi:10.1001/jama.2021.23619.
50. Rodgers, M.; Migdal, A.L.; Rodriguez, T.G.; Chen, Z.Z.; Nath, A.K.; Gerszten, R.E.; Kasid, N.; Toschi, E.; Tripaldi, J.; Heineman, B.; et al. Weight Loss Outcomes Among Early High Responders to Exenatide Treatment: A Randomized, Placebo Controlled Study in Overweight and Obese Women. *Front Endocrinol (Lausanne)* **2021**, *12*, 742873, doi:10.3389/fendo.2021.742873.
51. Kosiborod, M.N.; Esterline, R.; Furtado, R.H.M.; Oscarsson, J.; Gasparyan, S.B.; Koch, G.G.; Martinez, F.; Mukhtar, O.; Verma, S.; Chopra, V.; et al. Dapagliflozin in patients with cardiometabolic risk factors hospitalised with COVID-19 (DARE-19): a randomised, double-blind, placebo-controlled, phase 3 trial. *Lancet Diabetes Endocrinol* **2021**, *9*, 586-594, doi:10.1016/S2213-8587(21)00180-7.
52. Rosenstock, J.; Wysham, C.; Frias, J.P.; Kaneko, S.; Lee, C.J.; Fernandez Lando, L.; Mao, H.; Cui, X.; Karanikas, C.A.; Thieu, V.T. Efficacy and safety of a novel dual GIP and GLP-1 receptor agonist tirzepatide in patients with type 2 diabetes (SURPASS-1): a double-blind, randomised, phase 3 trial. *Lancet* **2021**, *398*, 143-155, doi:10.1016/S0140-6736(21)01324-6.
53. Davies, M.; Faerch, L.; Jeppesen, O.K.; Pakseresht, A.; Pedersen, S.D.; Perreault, L.; Rosenstock, J.; Shimomura, I.; Viljoen, A.; Wadden, T.A.; et al. Semaglutide 2.4 mg once a week in adults with overweight or obesity, and type 2 diabetes (STEP 2): a randomised, double-blind, double-dummy, placebo-controlled, phase 3 trial. *Lancet* **2021**, *397*, 971-984, doi:10.1016/S0140-6736(21)00213-0.
54. Wadden, T.A.; Bailey, T.S.; Billings, L.K.; Davies, M.; Frias, J.P.; Koroleva, A.; Lingvay, I.; O'Neil, P.M.; Rubino, D.M.; Skovgaard, D.; et al. Effect of Subcutaneous Semaglutide vs Placebo as an Adjunct to Intensive Behavioral Therapy on Body Weight in Adults With

Overweight or Obesity: The STEP 3 Randomized Clinical Trial. *Jama* **2021**, *325*, 1403-1413, doi:10.1001/jama.2021.1831.
55. Wilding, J.P.H.; Batterham, R.L.; Calanna, S.; Davies, M.; Van Gaal, L.F.; Lingvay, I.; McGowan, B.M.; Rosenstock, J.; Tran, M.T.D.; Wadden, T.A.; et al. Once-Weekly Semaglutide in Adults with Overweight or Obesity. *N Engl J Med* **2021**, *384*, 989-1002, doi:10.1056/NEJMoa2032183.
56. Bhatt, D.L.; Szarek, M.; Steg, P.G.; Cannon, C.P.; Leiter, L.A.; McGuire, D.K.; Lewis, J.B.; Riddle, M.C.; Voors, A.A.; Metra, M.; et al. Sotagliflozin in Patients with Diabetes and Recent Worsening Heart Failure. *N Engl J Med* **2021**, *384*, 117-128, doi:10.1056/NEJMoa2030183.
57. Stack, A.G.; Han, D.; Goldwater, R.; Johansson, S.; Dronamraju, N.; Oscarsson, J.; Johnsson, E.; Parkinson, J.; Erlandsson, F. Dapagliflozin Added to Verinurad Plus Febuxostat Further Reduces Serum Uric Acid in Hyperuricemia: The QUARTZ Study. *J Clin Endocrinol Metab* **2021**, *106*, e2347-e2356, doi:10.1210/clinem/dgaa748.
58. Wang, J.; Li, H.Q.; Xu, X.H.; Kong, X.C.; Sun, R.; Jing, T.; Ye, L.; Su, X.F.; Ma, J.H. The Effects of Once-Weekly Dulaglutide and Insulin Glargine on Glucose Fluctuation in Poorly Oral-Antidiabetic Controlled Patients with Type 2 Diabetes Mellitus. *Biomed Res Int* **2019**, *2019*, 2682657, doi:10.1155/2019/2682657.
59. Cherney, D.Z.I.; Ferrannini, E.; Umpierrez, G.E.; Peters, A.L.; Rosenstock, J.; Powell, D.R.; Davies, M.J.; Banks, P.; Agarwal, R. Efficacy and safety of sotagliflozin in patients with type 2 diabetes and stage 3 chronic kidney disease. *Diabetes Obes Metab* **2023**, *25*, 1646-1657, doi:10.1111/dom.15019.
60. Inagaki, N.; Takeuchi, M.; Oura, T.; Imaoka, T.; Seino, Y. Efficacy and safety of tirzepatide monotherapy compared with dulaglutide in Japanese patients with type 2 diabetes (SURPASS J-mono): a double-blind, multicentre, randomised, phase 3 trial. *Lancet Diabetes Endocrinol* **2022**, *10*, 623-633, doi:10.1016/S2213-8587(22)00188-7.
61. Kadowaki, T.; Chin, R.; Ozeki, A.; Imaoka, T.; Ogawa, Y. Safety and efficacy of tirzepatide as an add-on to single oral antihyperglycaemic medication in patients with type 2 diabetes in Japan (SURPASS J-combo): a multicentre, randomised, open-label, parallel-group, phase 3 trial. *Lancet Diabetes Endocrinol* **2022**, *10*, 634-644, doi:10.1016/S2213-8587(22)00187-5.
62. Wada, T.; Mori-Anai, K.; Takahashi, A.; Matsui, T.; Inagaki, M.; Iida, M.; Maruyama, K.; Tsuda, H. Effect of canagliflozin on the decline of

estimated glomerular filtration rate in chronic kidney disease patients with type 2 diabetes mellitus: A multicenter, randomized, double-blind, placebo-controlled, parallel-group, phase III study in Japan. *J Diabetes Investig* **2022**, *13*, 1981-1989, doi:10.1111/jdi.13888.
63. Tuttle, K.R.; Levin, A.; Nangaku, M.; Kadowaki, T.; Agarwal, R.; Hauske, S.J.; Elsasser, A.; Ritter, I.; Steubl, D.; Wanner, C.; et al. Safety of Empagliflozin in Patients With Type 2 Diabetes and Chronic Kidney Disease: Pooled Analysis of Placebo-Controlled Clinical Trials. *Diabetes Care* **2022**, *45*, 1445-1452, doi:10.2337/dc21-2034.
64. Heise, T.; Mari, A.; DeVries, J.H.; Urva, S.; Li, J.; Pratt, E.J.; Coskun, T.; Thomas, M.K.; Mather, K.J.; Haupt, A.; et al. Effects of subcutaneous tirzepatide versus placebo or semaglutide on pancreatic islet function and insulin sensitivity in adults with type 2 diabetes: a multicentre, randomised, double-blind, parallel-arm, phase 1 clinical trial. *Lancet Diabetes Endocrinol* **2022**, *10*, 418-429, doi:10.1016/S2213-8587(22)00085-7.
65. Fox, C.K.; Clark, J.M.; Rudser, K.D.; Ryder, J.R.; Gross, A.C.; Nathan, B.M.; Sunni, M.; Dengel, D.R.; Billington, C.J.; Bensignor, M.O.; et al. Exenatide for weight-loss maintenance in adolescents with severe obesity: A randomized, placebo-controlled trial. *Obesity (Silver Spring)* **2022**, *30*, 1105-1115, doi:10.1002/oby.23395.
66. Spertus, J.A.; Birmingham, M.C.; Nassif, M.; Damaraju, C.V.; Abbate, A.; Butler, J.; Lanfear, D.E.; Lingvay, I.; Kosiborod, M.N.; Januzzi, J.L. The SGLT2 inhibitor canagliflozin in heart failure: the CHIEF-HF remote, patient-centered randomized trial. *Nat Med* **2022**, *28*, 809-813, doi:10.1038/s41591-022-01703-8.
67. Dahl, D.; Onishi, Y.; Norwood, P.; Huh, R.; Bray, R.; Patel, H.; Rodriguez, A. Effect of Subcutaneous Tirzepatide vs Placebo Added to Titrated Insulin Glargine on Glycemic Control in Patients With Type 2 Diabetes: The SURPASS-5 Randomized Clinical Trial. *Jama* **2022**, *327*, 534-545, doi:10.1001/jama.2022.0078.
68. Kadowaki, T.; Isendahl, J.; Khalid, U.; Lee, S.Y.; Nishida, T.; Ogawa, W.; Tobe, K.; Yamauchi, T.; Lim, S.; investigators, S. Semaglutide once a week in adults with overweight or obesity, with or without type 2 diabetes in an east Asian population (STEP 6): a randomised, double-blind, double-dummy, placebo-controlled, phase 3a trial. *Lancet Diabetes Endocrinol* **2022**, *10*, 193-206, doi:10.1016/S2213-8587(22)00008-0.
69. Mu, Y.; Bao, X.; Eliaschewitz, F.G.; Hansen, M.R.; Kim, B.T.; Koroleva, A.; Ma, R.C.W.; Yang, T.; Zu, N.; Liu, M.; et al. Efficacy and safety of

once weekly semaglutide 2.4 mg for weight management in a predominantly east Asian population with overweight or obesity (STEP 7): a double-blind, multicentre, randomised controlled trial. *Lancet Diabetes Endocrinol* **2024**, *12*, 184-195, doi:10.1016/S2213-8587(23)00388-1.
70. Lee, B.W.; Cho, Y.M.; Kim, S.G.; Ko, S.H.; Lim, S.; Dahaoui, A.; Jeong, J.S.; Lim, H.J.; Yu, J.M. Efficacy and Safety of Once-Weekly Semaglutide Versus Once-Daily Sitagliptin as Metformin Add-on in a Korean Population with Type 2 Diabetes. *Diabetes Ther* **2024**, *15*, 547-563, doi:10.1007/s13300-023-01515-0.
71. Tuttle, K.R.; Hauske, S.J.; Canziani, M.E.; Caramori, M.L.; Cherney, D.; Cronin, L.; Heerspink, H.J.L.; Hugo, C.; Nangaku, M.; Rotter, R.C.; et al. Efficacy and safety of aldosterone synthase inhibition with and without empagliflozin for chronic kidney disease: a randomised, controlled, phase 2 trial. *Lancet* **2024**, *403*, 379-390, doi:10.1016/S0140-6736(23)02408-X.
72. Aronne, L.J.; Sattar, N.; Horn, D.B.; Bays, H.E.; Wharton, S.; Lin, W.Y.; Ahmad, N.N.; Zhang, S.; Liao, R.; Bunck, M.C.; et al. Continued Treatment With Tirzepatide for Maintenance of Weight Reduction in Adults With Obesity: The SURMOUNT-4 Randomized Clinical Trial. *Jama* **2024**, *331*, 38-48, doi:10.1001/jama.2023.24945.
73. Wadden, T.A.; Chao, A.M.; Machineni, S.; Kushner, R.; Ard, J.; Srivastava, G.; Halpern, B.; Zhang, S.; Chen, J.; Bunck, M.C.; et al. Tirzepatide after intensive lifestyle intervention in adults with overweight or obesity: the SURMOUNT-3 phase 3 trial. *Nat Med* **2023**, *29*, 2909-2918, doi:10.1038/s41591-023-02597-w.
74. Rosenstock, J.; Frias, J.P.; Rodbard, H.W.; Tofe, S.; Sears, E.; Huh, R.; Fernandez Lando, L.; Patel, H. Tirzepatide vs Insulin Lispro Added to Basal Insulin in Type 2 Diabetes: The SURPASS-6 Randomized Clinical Trial. *Jama* **2023**, *330*, 1631-1640, doi:10.1001/jama.2023.20294.
75. Garvey, W.T.; Frias, J.P.; Jastreboff, A.M.; le Roux, C.W.; Sattar, N.; Aizenberg, D.; Mao, H.; Zhang, S.; Ahmad, N.N.; Bunck, M.C.; et al. Tirzepatide once weekly for the treatment of obesity in people with type 2 diabetes (SURMOUNT-2): a double-blind, randomised, multicentre, placebo-controlled, phase 3 trial. *Lancet* **2023**, *402*, 613-626, doi:10.1016/S0140-6736(23)01200-X.
76. Frias, J.P.; Hsia, S.; Eyde, S.; Liu, R.; Ma, X.; Konig, M.; Kazda, C.; Mather, K.J.; Haupt, A.; Pratt, E.; et al. Efficacy and safety of oral orforglipron in patients with type 2 diabetes: a multicentre, randomised, dose-response, phase 2 study. *Lancet* **2023**, *402*, 472-483, doi:10.1016/S0140-6736(23)01302-8.

77. Feng, P.; Sheng, X.; Ji, Y.; Urva, S.; Wang, F.; Miller, S.; Qian, C.; An, Z.; Cui, Y. A Phase 1 Multiple Dose Study of Tirzepatide in Chinese Patients with Type 2 Diabetes. *Adv Ther* **2023**, *40*, 3434-3445, doi:10.1007/s12325-023-02536-8.
78. Gao, L.; Lee, B.W.; Chawla, M.; Kim, J.; Huo, L.; Du, L.; Huang, Y.; Ji, L. Tirzepatide versus insulin glargine as second-line or third-line therapy in type 2 diabetes in the Asia-Pacific region: the SURPASS-AP-Combo trial. *Nat Med* **2023**, *29*, 1500-1510, doi:10.1038/s41591-023-02344-1.
79. Zhao, L.; Cheng, Z.; Lu, Y.; Liu, M.; Chen, H.; Zhang, M.; Wang, R.; Yuan, Y.; Li, X. Tirzepatide for Weight Reduction in Chinese Adults With Obesity: The SURMOUNT-CN Randomized Clinical Trial. *Jama* **2024**, *332*, 551-560, doi:10.1001/jama.2024.9217.
80. Natale, P.; Tunnicliffe, D.J.; Toyama, T.; Palmer, S.C.; Saglimbene, V.M.; Ruospo, M.; Gargano, L.; Stallone, G.; Gesualdo, L.; Strippoli, G.F. Sodium-glucose co-transporter protein 2 (SGLT2) inhibitors for people with chronic kidney disease and diabetes. *The Cochrane database of systematic reviews* **2024**, *5*, CD015588, doi:10.1002/14651858.CD015588.pub2.
81. Dei Cas, A.; Micheli, M.M.; Aldigeri, R.; Gardini, S.; Ferrari-Pellegrini, F.; Perini, M.; Messa, G.; Antonini, M.; Spigoni, V.; Cinquegrani, G.; et al. Long-acting exenatide does not prevent cognitive decline in mild cognitive impairment: a proof-of-concept clinical trial. *Journal of endocrinological investigation* **2024**, *47*, 2339-2349, doi:10.1007/s40618-024-02320-7.
82. SURMOUNT-J. A Study of Tirzepatide (LY3298176) in Participants With Obesity Disease (SURMOUNT-J). Available online: https://clinicaltrials.gov/study/NCT04844918?cond=NCT04844918&rank=1 (accessed on 2024/10/28).
83. Leipold, G.; Toth, R.; Harsfalvi, P.; Loczi, L.; Torok, M.; Keszthelyi, A.; Acs, N.; Lintner, B.; Varbiro, S.; Keszthelyi, M. Comprehensive Evaluation of a Levonorgestrel Intrauterine Device (LNG-IUD), Metformin, and Liraglutide for Fertility Preservation in Endometrial Cancer: Protocol for a Randomized Clinical Trial. *Life (Basel)* **2024**, *14*, doi:10.3390/life14070835.
84. Bliddal, H.; Bays, H.; Czernichow, S.; Udden Hemmingsson, J.; Hjelmesaeth, J.; Hoffmann Morville, T.; Koroleva, A.; Skov Neergaard, J.; Velez Sanchez, P.; Wharton, S.; et al. Once-Weekly Semaglutide in Persons with Obesity and Knee Osteoarthritis. *N Engl J Med* **2024**, *391*, 1573-1583, doi:10.1056/NEJMoa2403664.
85. Aroda, V.R.; Frias, J.P.; Ji, L.; Niemoeller, E.; Nguyen-Pascal, M.L.; Denkel, K.; Espinasse, M.; Guo, H.; Baek, S.; Choi, J.; et al. Efficacy and safety of once-weekly efpeglenatide in people with suboptimally controlled type 2 diabetes: The AMPLITUDE-D, AMPLITUDE-L and

AMPLITUDE-S randomized controlled trials. *Diabetes Obes Metab* **2023**, *25*, 2084-2095, doi:10.1111/dom.15079.
86. Buse, J.B.; Nordahl Christensen, H.; Harty, B.J.; Mitchell, J.; Soule, B.P.; Zacherle, E.; Cziraky, M.; Willey, V.J. Study design and baseline profile for adults with type 2 diabetes in the once-weekly subcutaneous SEmaglutide randomized PRAgmatic (SEPRA) trial. *BMJ Open Diabetes Res Care* **2023**, *11*, doi:10.1136/bmjdrc-2022-003206.
87. The, E.-K.C.G.; Herrington, W.G.; Staplin, N.; Wanner, C.; Green, J.B.; Hauske, S.J.; Emberson, J.R.; Preiss, D.; Judge, P.; Mayne, K.J.; et al. Empagliflozin in Patients with Chronic Kidney Disease. *N Engl J Med* **2023**, *388*, 117-127, doi:10.1056/NEJMoa2204233.
88. Ji, L.; Lu, Y.; Li, Q.; Fu, L.; Luo, Y.; Lei, T.; Li, L.; Ye, S.; Shi, B.; Li, X.; et al. Efficacy and safety of empagliflozin in combination with insulin in Chinese patients with type 2 diabetes and insufficient glycaemic control: A phase III, randomized, double-blind, placebo-controlled, parallel study. *Diabetes Obes Metab* **2023**, *25*, 1839-1848, doi:10.1111/dom.15041.
89. Lincoff, A.M.; Brown-Frandsen, K.; Colhoun, H.M.; Deanfield, J.; Emerson, S.S.; Esbjerg, S.; Hardt-Lindberg, S.; Hovingh, G.K.; Kahn, S.E.; Kushner, R.F.; et al. Semaglutide and Cardiovascular Outcomes in Obesity without Diabetes. *N Engl J Med* **2023**, *389*, 2221-2232, doi:10.1056/NEJMoa2307563.
90. Frias, J.P.; Choi, J.; Rosenstock, J.; Popescu, L.; Niemoeller, E.; Muehlen-Bartmer, I.; Baek, S. Efficacy and Safety of Once-Weekly Efpeglenatide Monotherapy Versus Placebo in Type 2 Diabetes: The AMPLITUDE-M Randomized Controlled Trial. *Diabetes Care* **2022**, *45*, 1592-1600, doi:10.2337/dc21-2656.
91. Garvey, W.T.; Batterham, R.L.; Bhatta, M.; Buscemi, S.; Christensen, L.N.; Frias, J.P.; Jodar, E.; Kandler, K.; Rigas, G.; Wadden, T.A.; et al. Two-year effects of semaglutide in adults with overweight or obesity: the STEP 5 trial. *Nat Med* **2022**, *28*, 2083-2091, doi:10.1038/s41591-022-02026-4.
92. Jastreboff, A.M.; Aronne, L.J.; Ahmad, N.N.; Wharton, S.; Connery, L.; Alves, B.; Kiyosue, A.; Zhang, S.; Liu, B.; Bunck, M.C.; et al. Tirzepatide Once Weekly for the Treatment of Obesity. *N Engl J Med* **2022**, *387*, 205-216, doi:10.1056/NEJMoa2206038.
93. Kellerer, M.; Kaltoft, M.S.; Lawson, J.; Nielsen, L.L.; Strojek, K.; Tabak, O.; Jacob, S. Effect of once-weekly semaglutide versus thrice-daily insulin aspart, both as add-on to metformin and optimized insulin glargine treatment in participants with type 2 diabetes (SUSTAIN 11): A randomized, open-label, multinational, phase 3b trial. *Diabetes Obes Metab* **2022**, *24*, 1788-1799, doi:10.1111/dom.14765.

94. Solomon, S.D.; McMurray, J.J.V.; Claggett, B.; de Boer, R.A.; DeMets, D.; Hernandez, A.F.; Inzucchi, S.E.; Kosiborod, M.N.; Lam, C.S.P.; Martinez, F.; et al. Dapagliflozin in Heart Failure with Mildly Reduced or Preserved Ejection Fraction. *N Engl J Med* **2022**, *387*, 1089-1098, doi:10.1056/NEJMoa2206286.
95. Voors, A.A.; Angermann, C.E.; Teerlink, J.R.; Collins, S.P.; Kosiborod, M.; Biegus, J.; Ferreira, J.P.; Nassif, M.E.; Psotka, M.A.; Tromp, J.; et al. The SGLT2 inhibitor empagliflozin in patients hospitalized for acute heart failure: a multinational randomized trial. *Nat Med* **2022**, *28*, 568-574, doi:10.1038/s41591-021-01659-1.
96. Anker, S.D.; Butler, J.; Filippatos, G.; Ferreira, J.P.; Bocchi, E.; Bohm, M.; Brunner-La Rocca, H.P.; Choi, D.J.; Chopra, V.; Chuquiure-Valenzuela, E.; et al. Empagliflozin in Heart Failure with a Preserved Ejection Fraction. *N Engl J Med* **2021**, *385*, 1451-1461, doi:10.1056/NEJMoa2107038.
97. Bhatt, D.L.; Szarek, M.; Pitt, B.; Cannon, C.P.; Leiter, L.A.; McGuire, D.K.; Lewis, J.B.; Riddle, M.C.; Inzucchi, S.E.; Kosiborod, M.N.; et al. Sotagliflozin in Patients with Diabetes and Chronic Kidney Disease. *N Engl J Med* **2021**, *384*, 129-139, doi:10.1056/NEJMoa2030186.
98. Del Prato, S.; Kahn, S.E.; Pavo, I.; Weerakkody, G.J.; Yang, Z.; Doupis, J.; Aizenberg, D.; Wynne, A.G.; Riesmeyer, J.S.; Heine, R.J.; et al. Tirzepatide versus insulin glargine in type 2 diabetes and increased cardiovascular risk (SURPASS-4): a randomised, open-label, parallel-group, multicentre, phase 3 trial. *Lancet* **2021**, *398*, 1811-1824, doi:10.1016/S0140-6736(21)02188-7.
99. Frias, J.P.; Davies, M.J.; Rosenstock, J.; Perez Manghi, F.C.; Fernandez Lando, L.; Bergman, B.K.; Liu, B.; Cui, X.; Brown, K.; Investigators, S.-. Tirzepatide versus Semaglutide Once Weekly in Patients with Type 2 Diabetes. *N Engl J Med* **2021**, *385*, 503-515, doi:10.1056/NEJMoa2107519.
100. Gerstein, H.C.; Sattar, N.; Rosenstock, J.; Ramasundarahettige, C.; Pratley, R.; Lopes, R.D.; Lam, C.S.P.; Khurmi, N.S.; Heenan, L.; Del Prato, S.; et al. Cardiovascular and Renal Outcomes with Efpeglenatide in Type 2 Diabetes. *N Engl J Med* **2021**, *385*, 896-907, doi:10.1056/NEJMoa2108269.
101. Lock, J.P. Bexagliflozin Efficacy and Safety Trial (BEST). Available online: https://clinicaltrials.gov/study/NCT02558296?cond=NCT02558296&rank=1 (accessed on 2024/10/28).
102. Ludvik, B.; Giorgino, F.; Jodar, E.; Frias, J.P.; Fernandez Lando, L.; Brown, K.; Bray, R.; Rodriguez, A. Once-weekly tirzepatide versus once-

daily insulin degludec as add-on to metformin with or without SGLT2 inhibitors in patients with type 2 diabetes (SURPASS-3): a randomised, open-label, parallel-group, phase 3 trial. *Lancet* **2021**, *398*, 583-598, doi:10.1016/S0140-6736(21)01443-4.
103. Rubino, D.; Abrahamsson, N.; Davies, M.; Hesse, D.; Greenway, F.L.; Jensen, C.; Lingvay, I.; Mosenzon, O.; Rosenstock, J.; Rubio, M.A.; et al. Effect of Continued Weekly Subcutaneous Semaglutide vs Placebo on Weight Loss Maintenance in Adults With Overweight or Obesity: The STEP 4 Randomized Clinical Trial. *Jama* **2021**, *325*, 1414-1425, doi:10.1001/jama.2021.3224.
104. Wason, S. Efficacy and Bone Safety of Sotagliflozin 400 and 200 mg Versus Placebo in Participants With Type 2 Diabetes Mellitus Who Have Inadequate Glycemic Control (SOTA-BONE). Available online: https://clinicaltrials.gov/study/NCT03386344?cond=NCT03386344&rank=1 (accessed on 2024/10/28).
105. Wason, S. Efficacy and Safety of Sotagliflozin Versus Placebo in Participants With Type 2 Diabetes Mellitus Who Have Inadequate Glycemic Control While Taking Insulin Alone or With Other Oral Antidiabetic Agents (SOTA-INS). Available online: https://clinicaltrials.gov/study/NCT03285594?cond=NCT03285594&rank=1 (accessed on 2024/10/28).
106. Cannon, C.P.; Pratley, R.; Dagogo-Jack, S.; Mancuso, J.; Huyck, S.; Masiukiewicz, U.; Charbonnel, B.; Frederich, R.; Gallo, S.; Cosentino, F.; et al. Cardiovascular Outcomes with Ertugliflozin in Type 2 Diabetes. *N Engl J Med* **2020**, *383*, 1425-1435, doi:10.1056/NEJMoa2004967.
107. Heerspink, H.J.L.; Stefansson, B.V.; Correa-Rotter, R.; Chertow, G.M.; Greene, T.; Hou, F.F.; Mann, J.F.E.; McMurray, J.J.V.; Lindberg, M.; Rossing, P.; et al. Dapagliflozin in Patients with Chronic Kidney Disease. *N Engl J Med* **2020**, *383*, 1436-1446, doi:10.1056/NEJMoa2024816.
108. Packer, M.; Anker, S.D.; Butler, J.; Filippatos, G.; Pocock, S.J.; Carson, P.; Januzzi, J.; Verma, S.; Tsutsui, H.; Brueckmann, M.; et al. Cardiovascular and Renal Outcomes with Empagliflozin in Heart Failure. *N Engl J Med* **2020**, *383*, 1413-1424, doi:10.1056/NEJMoa2022190.
109. Gallo, S.; Charbonnel, B.; Goldman, A.; Shi, H.; Huyck, S.; Darekar, A.; Lauring, B.; Terra, S.G. Long-term efficacy and safety of ertugliflozin in patients with type 2 diabetes mellitus inadequately controlled with metformin monotherapy: 104-week VERTIS MET trial. *Diabetes Obes Metab* **2019**, *21*, 1027-1036, doi:10.1111/dom.13631.
110. Gerstein, H.C.; Colhoun, H.M.; Dagenais, G.R.; Diaz, R.; Lakshmanan, M.; Pais, P.; Probstfield, J.; Riesmeyer, J.S.; Riddle, M.C.; Ryden, L.;

et al. Dulaglutide and cardiovascular outcomes in type 2 diabetes (REWIND): a double-blind, randomised placebo-controlled trial. *Lancet* **2019**, *394*, 121-130, doi:10.1016/S0140-6736(19)31149-3.

111. Husain, M.; Birkenfeld, A.L.; Donsmark, M.; Dungan, K.; Eliaschewitz, F.G.; Franco, D.R.; Jeppesen, O.K.; Lingvay, I.; Mosenzon, O.; Pedersen, S.D.; et al. Oral Semaglutide and Cardiovascular Outcomes in Patients with Type 2 Diabetes. *N Engl J Med* **2019**, *381*, 841-851, doi:10.1056/NEJMoa1901118.

112. Lingvay, I.; Catarig, A.M.; Frias, J.P.; Kumar, H.; Lausvig, N.L.; le Roux, C.W.; Thielke, D.; Viljoen, A.; McCrimmon, R.J. Efficacy and safety of once-weekly semaglutide versus daily canagliflozin as add-on to metformin in patients with type 2 diabetes (SUSTAIN 8): a double-blind, phase 3b, randomised controlled trial. *Lancet Diabetes Endocrinol* **2019**, *7*, 834-844, doi:10.1016/S2213-8587(19)30311-0.

113. McMurray, J.J.V.; Solomon, S.D.; Inzucchi, S.E.; Kober, L.; Kosiborod, M.N.; Martinez, F.A.; Ponikowski, P.; Sabatine, M.S.; Anand, I.S.; Belohlavek, J.; et al. Dapagliflozin in Patients with Heart Failure and Reduced Ejection Fraction. *N Engl J Med* **2019**, *381*, 1995-2008, doi:10.1056/NEJMoa1911303.

114. Perkovic, V.; Jardine, M.J.; Neal, B.; Bompoint, S.; Heerspink, H.J.L.; Charytan, D.M.; Edwards, R.; Agarwal, R.; Bakris, G.; Bull, S.; et al. Canagliflozin and Renal Outcomes in Type 2 Diabetes and Nephropathy. *N Engl J Med* **2019**, *380*, 2295-2306, doi:10.1056/NEJMoa1811744.

115. Pieber, T.R.; Bode, B.; Mertens, A.; Cho, Y.M.; Christiansen, E.; Hertz, C.L.; Wallenstein, S.O.R.; Buse, J.B.; investigators, P. Efficacy and safety of oral semaglutide with flexible dose adjustment versus sitagliptin in type 2 diabetes (PIONEER 7): a multicentre, open-label, randomised, phase 3a trial. *Lancet Diabetes Endocrinol* **2019**, *7*, 528-539, doi:10.1016/S2213-8587(19)30194-9.

116. Pratley, R.; Amod, A.; Hoff, S.T.; Kadowaki, T.; Lingvay, I.; Nauck, M.; Pedersen, K.B.; Saugstrup, T.; Meier, J.J.; investigators, P. Oral semaglutide versus subcutaneous liraglutide and placebo in type 2 diabetes (PIONEER 4): a randomised, double-blind, phase 3a trial. *Lancet* **2019**, *394*, 39-50, doi:10.1016/S0140-6736(19)31271-1.

117. Rosenstock, J.; Allison, D.; Birkenfeld, A.L.; Blicher, T.M.; Deenadayalan, S.; Jacobsen, J.B.; Serusclat, P.; Violante, R.; Watada, H.; Davies, M.; et al. Effect of Additional Oral Semaglutide vs Sitagliptin on Glycated Hemoglobin in Adults With Type 2 Diabetes Uncontrolled With Metformin Alone or With Sulfonylurea: The PIONEER 3 Randomized Clinical Trial. *Jama* **2019**, *321*, 1466-1480,

doi:10.1001/jama.2019.2942.
118. Wiviott, S.D.; Raz, I.; Bonaca, M.P.; Mosenzon, O.; Kato, E.T.; Cahn, A.; Silverman, M.G.; Zelniker, T.A.; Kuder, J.F.; Murphy, S.A.; et al. Dapagliflozin and Cardiovascular Outcomes in Type 2 Diabetes. *N Engl J Med* **2019**, *380*, 347-357, doi:10.1056/NEJMoa1812389.
119. Aronson, R.; Frias, J.; Goldman, A.; Darekar, A.; Lauring, B.; Terra, S.G. Long-term efficacy and safety of ertugliflozin monotherapy in patients with inadequately controlled T2DM despite diet and exercise: VERTIS MONO extension study. *Diabetes Obes Metab* **2018**, *20*, 1453-1460, doi:10.1111/dom.13251.
120. Danne, T.; Cariou, B.; Banks, P.; Brandle, M.; Brath, H.; Franek, E.; Kushner, J.A.; Lapuerta, P.; McGuire, D.K.; Peters, A.L.; et al. HbA(1c) and Hypoglycemia Reductions at 24 and 52 Weeks With Sotagliflozin in Combination With Insulin in Adults With Type 1 Diabetes: The European inTandem2 Study. *Diabetes Care* **2018**, *41*, 1981-1990, doi:10.2337/dc18-0342.
121. Hernandez, A.F.; Green, J.B.; Janmohamed, S.; D'Agostino, R.B., Sr.; Granger, C.B.; Jones, N.P.; Leiter, L.A.; Rosenberg, A.E.; Sigmon, K.N.; Somerville, M.C.; et al. Albiglutide and cardiovascular outcomes in patients with type 2 diabetes and cardiovascular disease (Harmony Outcomes): a double-blind, randomised placebo-controlled trial. *Lancet* **2018**, *392*, 1519-1529, doi:10.1016/S0140-6736(18)32261-X.
122. Kaku, K.; Yamada, Y.; Watada, H.; Abiko, A.; Nishida, T.; Zacho, J.; Kiyosue, A. Safety and efficacy of once-weekly semaglutide vs additional oral antidiabetic drugs in Japanese people with inadequately controlled type 2 diabetes: A randomized trial. *Diabetes Obes Metab* **2018**, *20*, 1202-1212, doi:10.1111/dom.13218.
123. O'Neil, P.M.; Birkenfeld, A.L.; McGowan, B.; Mosenzon, O.; Pedersen, S.D.; Wharton, S.; Carson, C.G.; Jepsen, C.H.; Kabisch, M.; Wilding, J.P.H. Efficacy and safety of semaglutide compared with liraglutide and placebo for weight loss in patients with obesity: a randomised, double-blind, placebo and active controlled, dose-ranging, phase 2 trial. *Lancet* **2018**, *392*, 637-649, doi:10.1016/S0140-6736(18)31773-2.
124. Pratley, R.E.; Aroda, V.R.; Lingvay, I.; Ludemann, J.; Andreassen, C.; Navarria, A.; Viljoen, A.; investigators, S. Semaglutide versus dulaglutide once weekly in patients with type 2 diabetes (SUSTAIN 7): a randomised, open-label, phase 3b trial. *Lancet Diabetes Endocrinol* **2018**, *6*, 275-286, doi:10.1016/S2213-8587(18)30024-X.
125. Ahren, B.; Masmiquel, L.; Kumar, H.; Sargin, M.; Karsbol, J.D.; Jacobsen, S.H.; Chow, F. Efficacy and safety of once-weekly semaglutide

versus once-daily sitagliptin as an add-on to metformin, thiazolidinediones, or both, in patients with type 2 diabetes (SUSTAIN 2): a 56-week, double-blind, phase 3a, randomised trial. *Lancet Diabetes Endocrinol* **2017**, *5*, 341-354, doi:10.1016/S2213-8587(17)30092-X.
126. Aroda, V.R.; Bain, S.C.; Cariou, B.; Piletic, M.; Rose, L.; Axelsen, M.; Rowe, E.; DeVries, J.H. Efficacy and safety of once-weekly semaglutide versus once-daily insulin glargine as add-on to metformin (with or without sulfonylureas) in insulin-naive patients with type 2 diabetes (SUSTAIN 4): a randomised, open-label, parallel-group, multicentre, multinational, phase 3a trial. *Lancet Diabetes Endocrinol* **2017**, *5*, 355-366, doi:10.1016/S2213-8587(17)30085-2.
127. Holman, R.R.; Bethel, M.A.; Mentz, R.J.; Thompson, V.P.; Lokhnygina, Y.; Buse, J.B.; Chan, J.C.; Choi, J.; Gustavson, S.M.; Iqbal, N.; et al. Effects of Once-Weekly Exenatide on Cardiovascular Outcomes in Type 2 Diabetes. *N Engl J Med* **2017**, *377*, 1228-1239, doi:10.1056/NEJMoa1612917.
128. Januzzi, J.L., Jr.; Butler, J.; Jarolim, P.; Sattar, N.; Vijapurkar, U.; Desai, M.; Davies, M.J. Effects of Canagliflozin on Cardiovascular Biomarkers in Older Adults With Type 2 Diabetes. *J Am Coll Cardiol* **2017**, *70*, 704-712, doi:10.1016/j.jacc.2017.06.016.
129. Neal, B.; Perkovic, V.; Mahaffey, K.W.; de Zeeuw, D.; Fulcher, G.; Erondu, N.; Shaw, W.; Law, G.; Desai, M.; Matthews, D.R.; et al. Canagliflozin and Cardiovascular and Renal Events in Type 2 Diabetes. *N Engl J Med* **2017**, *377*, 644-657, doi:10.1056/NEJMoa1611925.
130. Investigators, F.-S.T. Glucose Variability in a 26-Week Randomized Comparison of Mealtime Treatment With Rapid-Acting Insulin Versus GLP-1 Agonist in Participants With Type 2 Diabetes at High Cardiovascular Risk. *Diabetes Care* **2016**, *39*, 973-981, doi:10.2337/dc15-2782.
131. Marso, S.P.; Daniels, G.H.; Brown-Frandsen, K.; Kristensen, P.; Mann, J.F.; Nauck, M.A.; Nissen, S.E.; Pocock, S.; Poulter, N.R.; Ravn, L.S.; et al. Liraglutide and Cardiovascular Outcomes in Type 2 Diabetes. *N Engl J Med* **2016**, *375*, 311-322, doi:10.1056/NEJMoa1603827.
132. Marso, S.P.; Bain, S.C.; Consoli, A.; Eliaschewitz, F.G.; Jodar, E.; Leiter, L.A.; Lingvay, I.; Rosenstock, J.; Seufert, J.; Warren, M.L.; et al. Semaglutide and Cardiovascular Outcomes in Patients with Type 2 Diabetes. *N Engl J Med* **2016**, *375*, 1834-1844, doi:10.1056/NEJMoa1607141.
133. Mellander, A.; Billger, M.; Johnsson, E.; Traff, A.K.; Yoshida, S.; Johnsson, K. Hypersensitivity Events, Including Potentially Hypersensitivity-Related Skin Events, with Dapagliflozin in Patients with Type 2 Diabetes Mellitus: A Pooled Analysis. *Clinical drug*

investigation **2016**, *36*, 925-933, doi:10.1007/s40261-016-0438-3.
134. Nauck, M.A.; Stewart, M.W.; Perkins, C.; Jones-Leone, A.; Yang, F.; Perry, C.; Reinhardt, R.R.; Rendell, M. Efficacy and safety of once-weekly GLP-1 receptor agonist albiglutide (HARMONY 2): 52 week primary endpoint results from a randomised, placebo-controlled trial in patients with type 2 diabetes mellitus inadequately controlled with diet and exercise. *Diabetologia* **2016**, *59*, 266-274, doi:10.1007/s00125-015-3795-1.
135. Giorgino, F.; Benroubi, M.; Sun, J.H.; Zimmermann, A.G.; Pechtner, V. Efficacy and Safety of Once-Weekly Dulaglutide Versus Insulin Glargine in Patients With Type 2 Diabetes on Metformin and Glimepiride (AWARD-2). *Diabetes Care* **2015**, *38*, 2241-2249, doi:10.2337/dc14-1625.
136. Pfeffer, M.A.; Claggett, B.; Diaz, R.; Dickstein, K.; Gerstein, H.C.; Kober, L.V.; Lawson, F.C.; Ping, L.; Wei, X.; Lewis, E.F.; et al. Lixisenatide in Patients with Type 2 Diabetes and Acute Coronary Syndrome. *N Engl J Med* **2015**, *373*, 2247-2257, doi:10.1056/NEJMoa1509225.
137. Pi-Sunyer, X.; Astrup, A.; Fujioka, K.; Greenway, F.; Halpern, A.; Krempf, M.; Lau, D.C.; le Roux, C.W.; Violante Ortiz, R.; Jensen, C.B.; et al. A Randomized, Controlled Trial of 3.0 mg of Liraglutide in Weight Management. *N Engl J Med* **2015**, *373*, 11-22, doi:10.1056/NEJMoa1411892.
138. Zinman, B.; Wanner, C.; Lachin, J.M.; Fitchett, D.; Bluhmki, E.; Hantel, S.; Mattheus, M.; Devins, T.; Johansen, O.E.; Woerle, H.J.; et al. Empagliflozin, Cardiovascular Outcomes, and Mortality in Type 2 Diabetes. *N Engl J Med* **2015**, *373*, 2117-2128, doi:10.1056/NEJMoa1504720.
139. Barnett, A.H.; Mithal, A.; Manassie, J.; Jones, R.; Rattunde, H.; Woerle, H.J.; Broedl, U.C.; investigators, E.-R.R.t. Efficacy and safety of empagliflozin added to existing antidiabetes treatment in patients with type 2 diabetes and chronic kidney disease: a randomised, double-blind, placebo-controlled trial. *Lancet Diabetes Endocrinol* **2014**, *2*, 369-384, doi:10.1016/S2213-8587(13)70208-0.
140. Ridderstrale, M.; Andersen, K.R.; Zeller, C.; Kim, G.; Woerle, H.J.; Broedl, U.C.; investigators, E.-R.H.H.S.t. Comparison of empagliflozin and glimepiride as add-on to metformin in patients with type 2 diabetes: a 104-week randomised, active-controlled, double-blind, phase 3 trial. *Lancet Diabetes Endocrinol* **2014**, *2*, 691-700, doi:10.1016/S2213-8587(14)70120-2.
141. Weissman, P.N.; Carr, M.C.; Ye, J.; Cirkel, D.T.; Stewart, M.; Perry, C.; Pratley, R. HARMONY 4: randomised clinical trial comparing once-

weekly albiglutide and insulin glargine in patients with type 2 diabetes inadequately controlled with metformin with or without sulfonylurea. *Diabetologia* **2014**, *57*, 2475-2484, doi:10.1007/s00125-014-3360-3.
142. Roden, M.; Weng, J.; Eilbracht, J.; Delafont, B.; Kim, G.; Woerle, H.J.; Broedl, U.C.; investigators, E.-R.M.t. Empagliflozin monotherapy with sitagliptin as an active comparator in patients with type 2 diabetes: a randomised, double-blind, placebo-controlled, phase 3 trial. *Lancet Diabetes Endocrinol* **2013**, *1*, 208-219, doi:10.1016/S2213-8587(13)70084-6.
143. Rosenstock, J.; Raccah, D.; Koranyi, L.; Maffei, L.; Boka, G.; Miossec, P.; Gerich, J.E. Efficacy and safety of lixisenatide once daily versus exenatide twice daily in type 2 diabetes inadequately controlled on metformin: a 24-week, randomized, open-label, active-controlled study (GetGoal-X). *Diabetes Care* **2013**, *36*, 2945-2951, doi:10.2337/dc12-2709.
144. Gallwitz, B.; Guzman, J.; Dotta, F.; Guerci, B.; Simo, R.; Basson, B.R.; Festa, A.; Kiljanski, J.; Sapin, H.; Trautmann, M.; et al. Exenatide twice daily versus glimepiride for prevention of glycaemic deterioration in patients with type 2 diabetes with metformin failure (EUREXA): an open-label, randomised controlled trial. *Lancet* **2012**, *379*, 2270-2278, doi:10.1016/S0140-6736(12)60479-6.
